# Supplementary figures and images for: Exploring the Influence of Chemical Exposures in Breast Cancer Disparities: High-Throughput Transcriptomic Analysis in Normal Breast Cells from Diverse Donors
Source: bioRxiv. 2026 Feb 24:2026.02.23.707203. Preprint. [Version 1] doi: 10.64898/2026.02.23.707203 (PMC13160138; doi:10.64898/2026.02.23.707203)

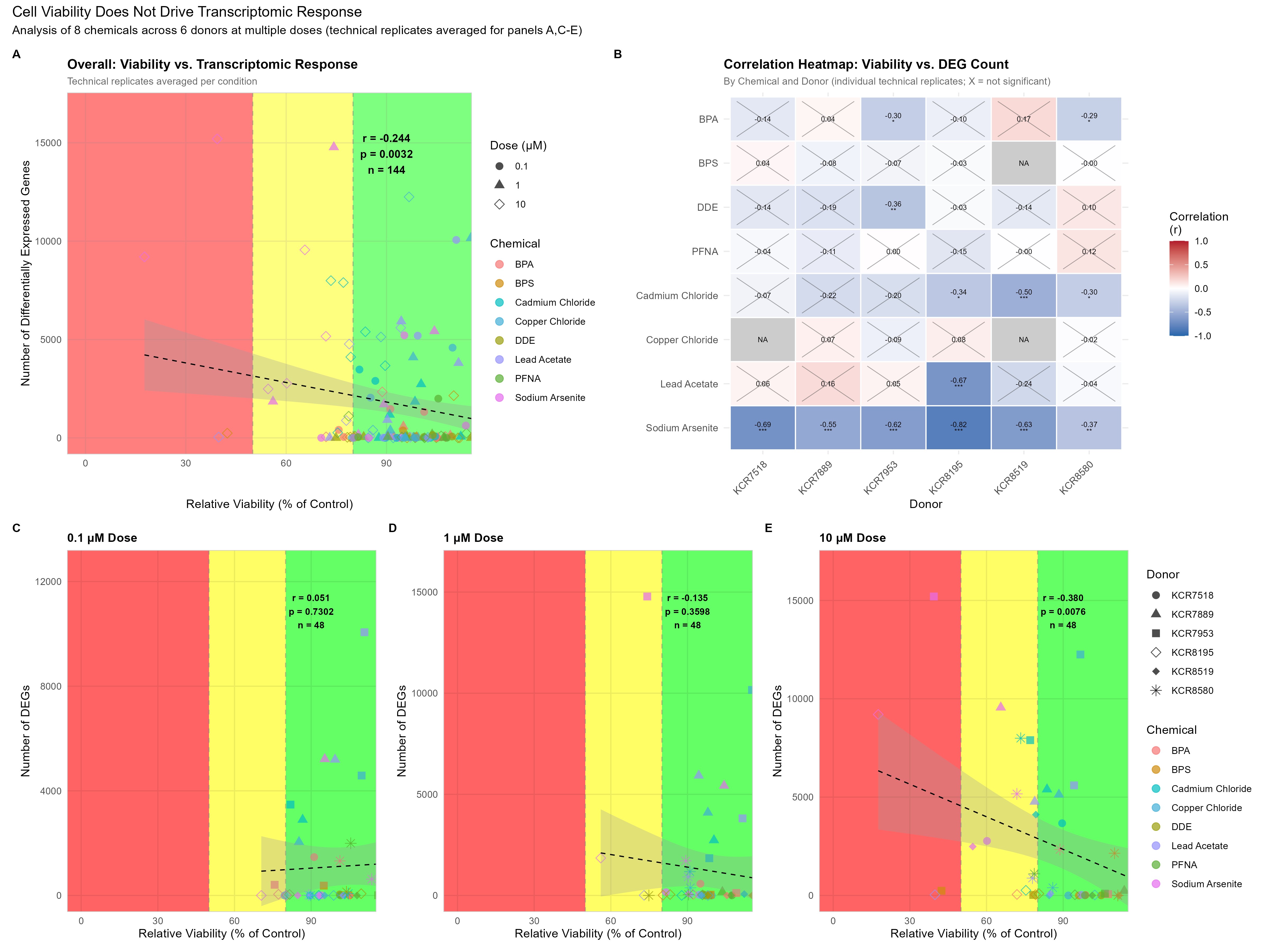

Supplement: Supplement 1 — (A) Scatterplot of relative cell viability (% of vehicle control) versus number of differentially expressed genes (DEGs) across all treatment conditions (n = 144; 6 donors × 8 chemicals × 3 doses). Each point represents technical replicates averaged per condition. Background shading indicates viability ranges: green (≥80%), yellow (50–80%), red (<50%). Dashed line shows linear regression fit. Pearson correlation: r = −0.244, p = 0.0032. (B) Heatmap of correlation coefficients between viability and DEG count for each chemical × donor combination. Correlations calculated using individual technical replicates per combination. Diagonal cross (×) indicates non-significant correlations (p ≥ 0.05). Numbers indicate correlation coefficient with significance level (*, p < 0.05; **, p < 0.01; ***, p < 0.001). Gray cells (NA) indicate no variance for correlation calculation. (C-E) Scatterplots of viability versus DEG count stratified by dose: 0.1 μM (C), 1 μM (D), and 10 μM (E). Each point represents technical replicates averaged per condition. Points colored by chemical and shaped by donor. Dashed line shows linear regression fit with Pearson correlation coefficient and p-value reported for each dose. [file media-1.jpg]

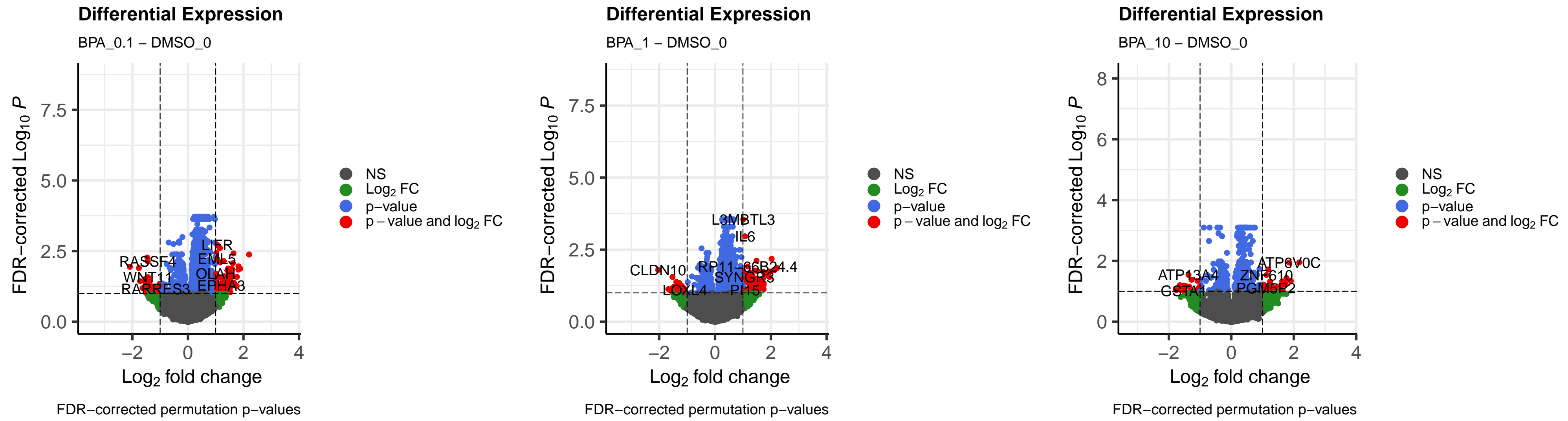

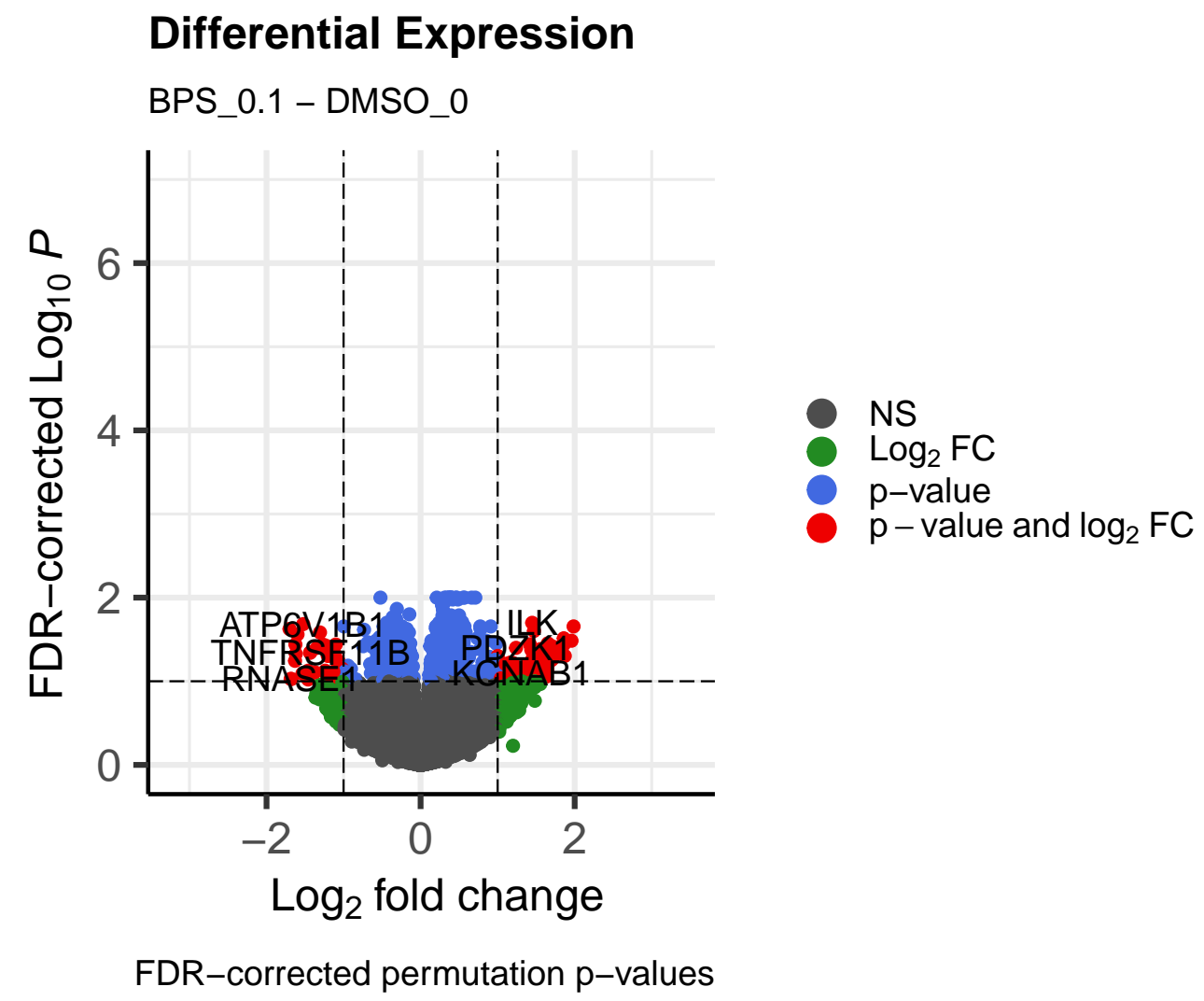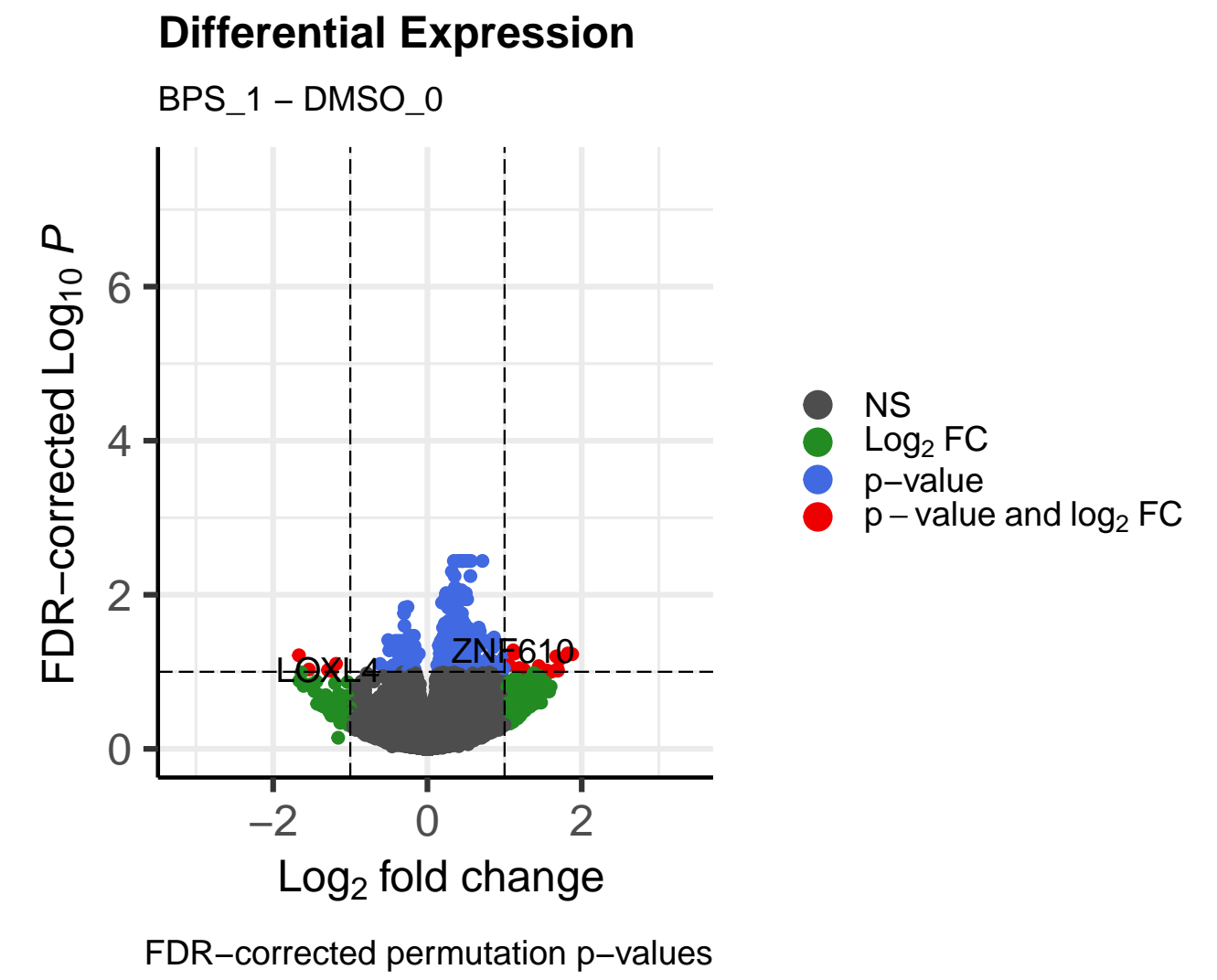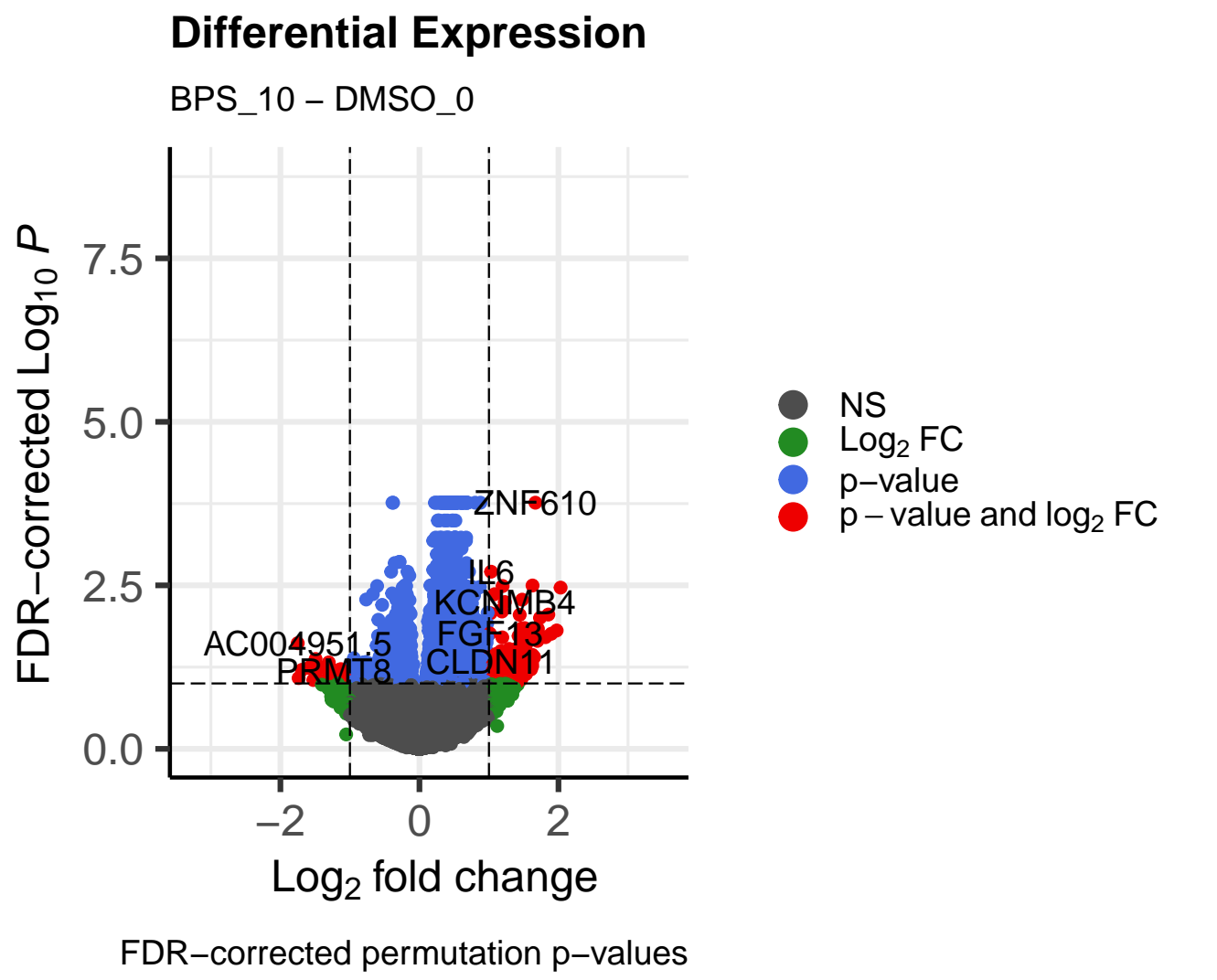

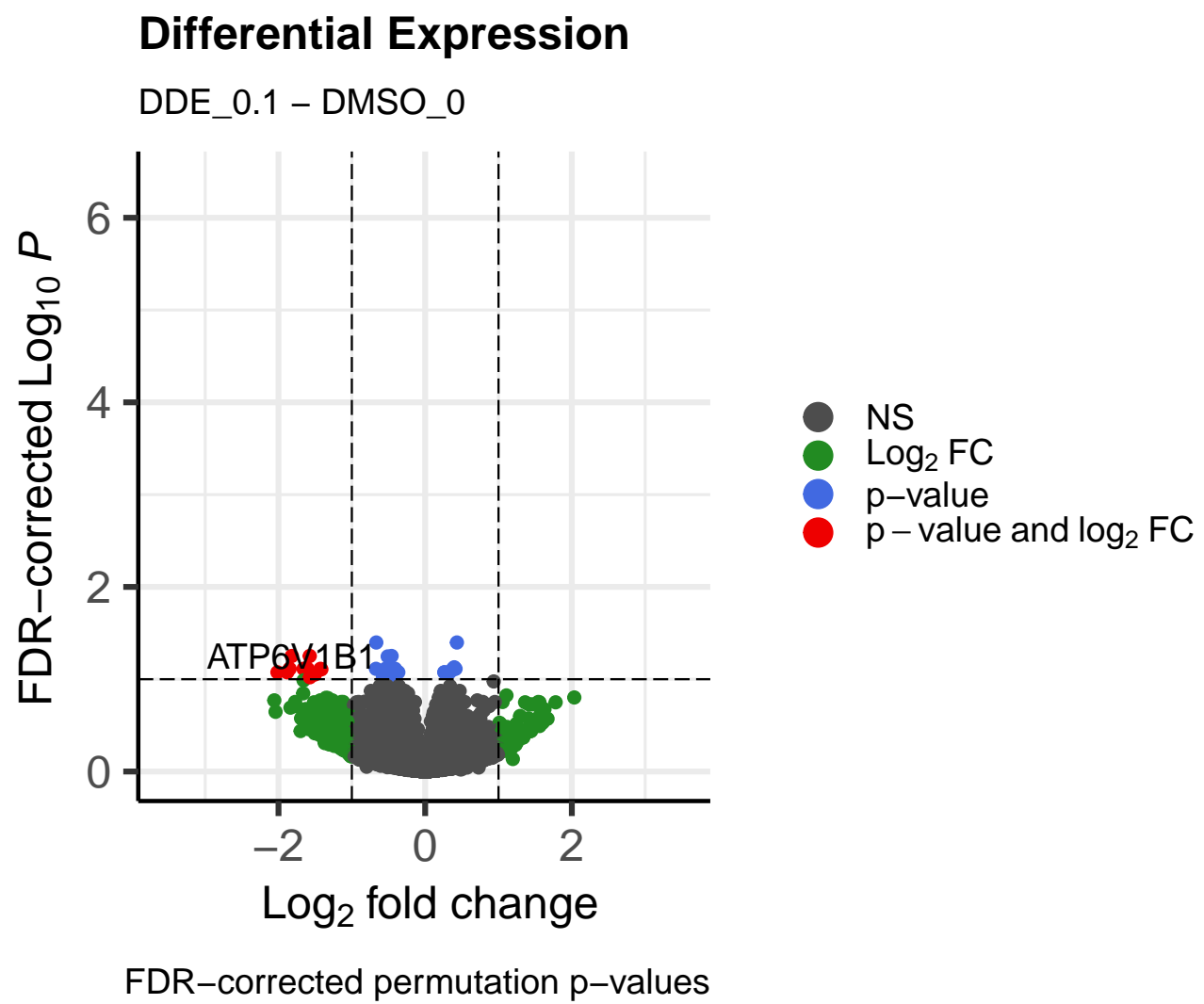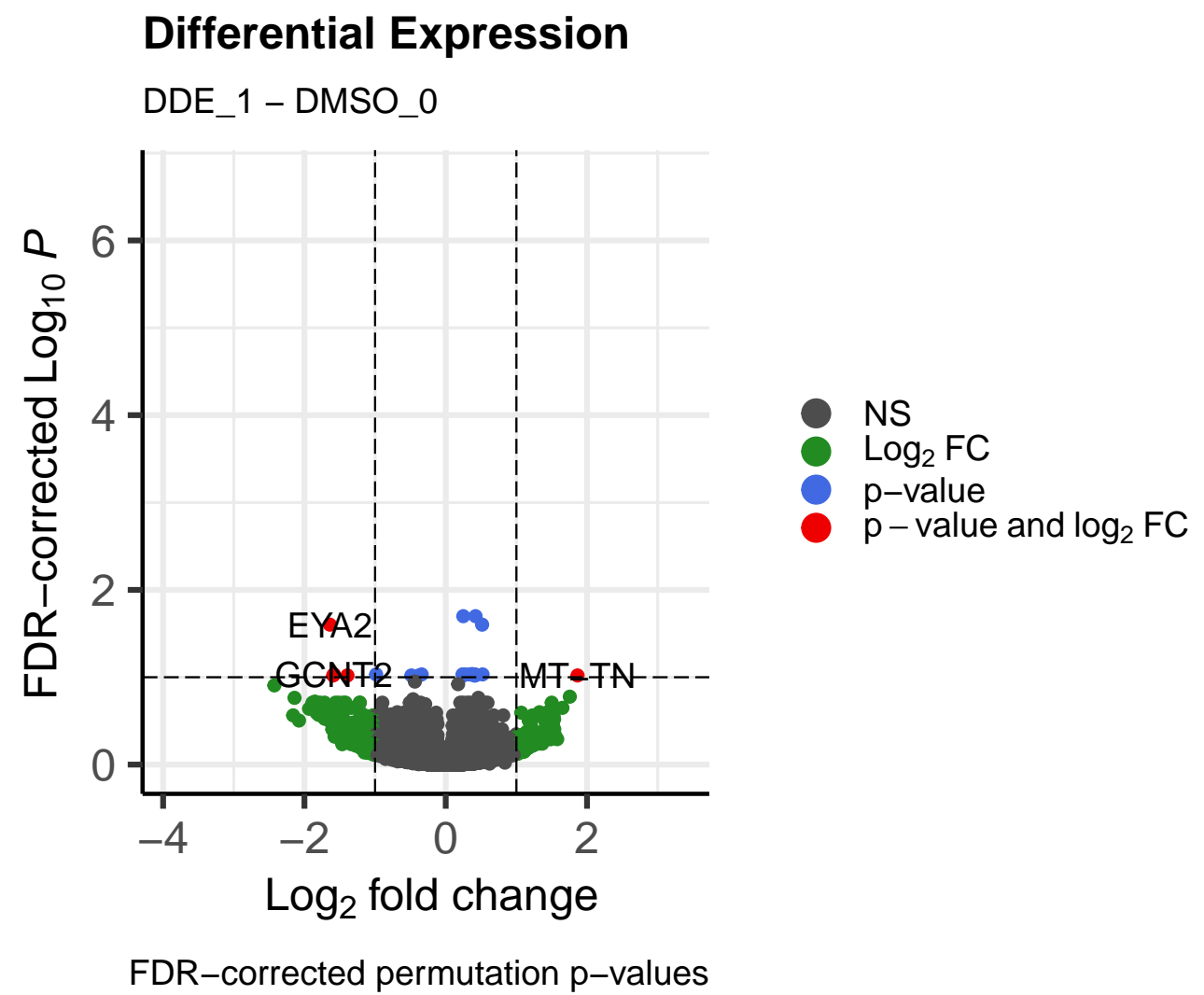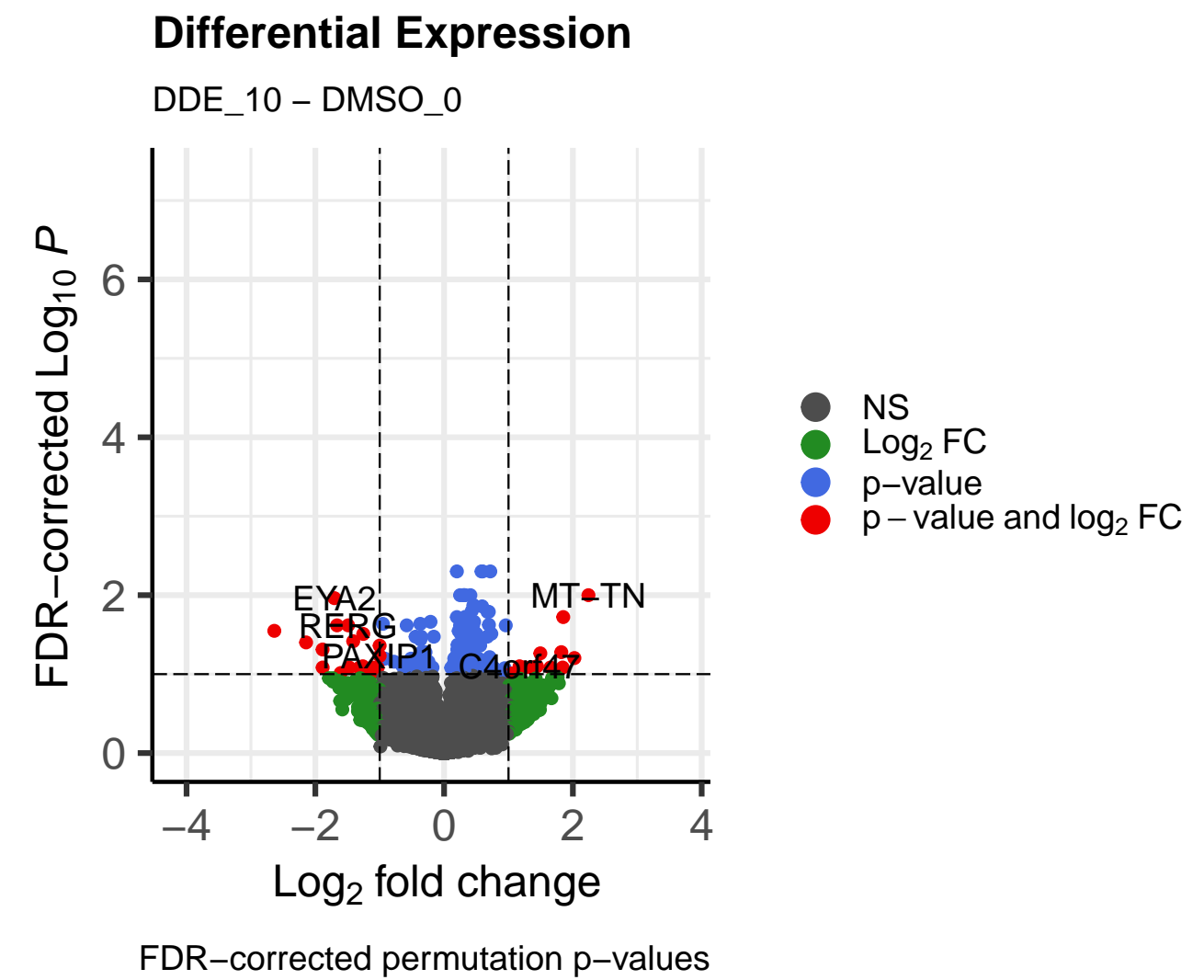

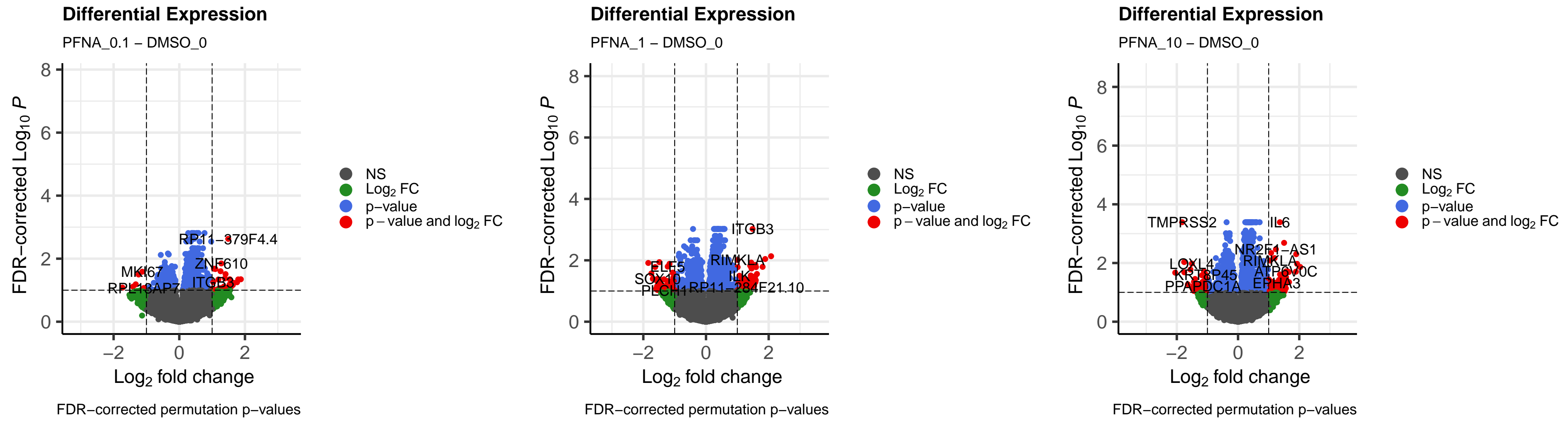

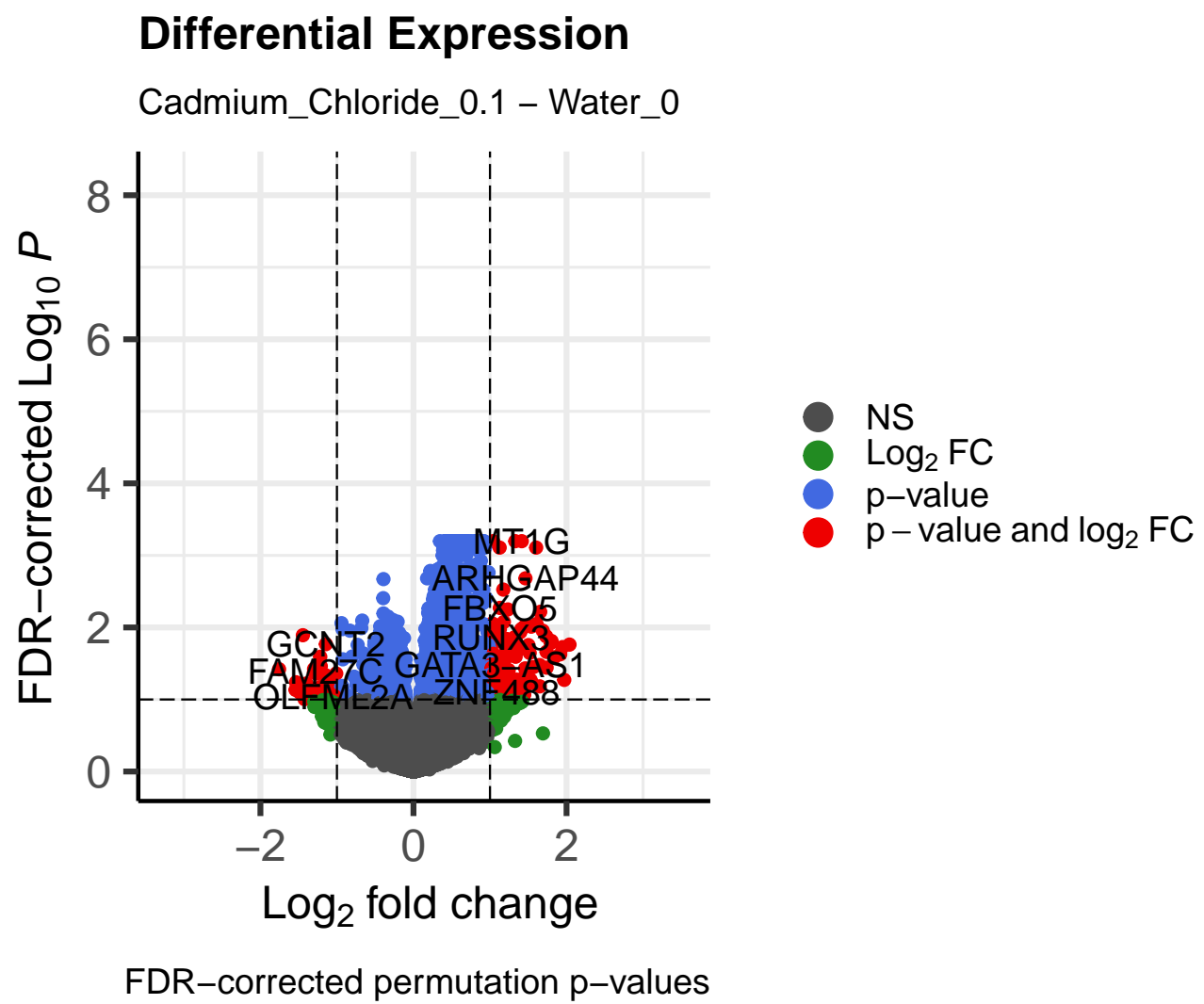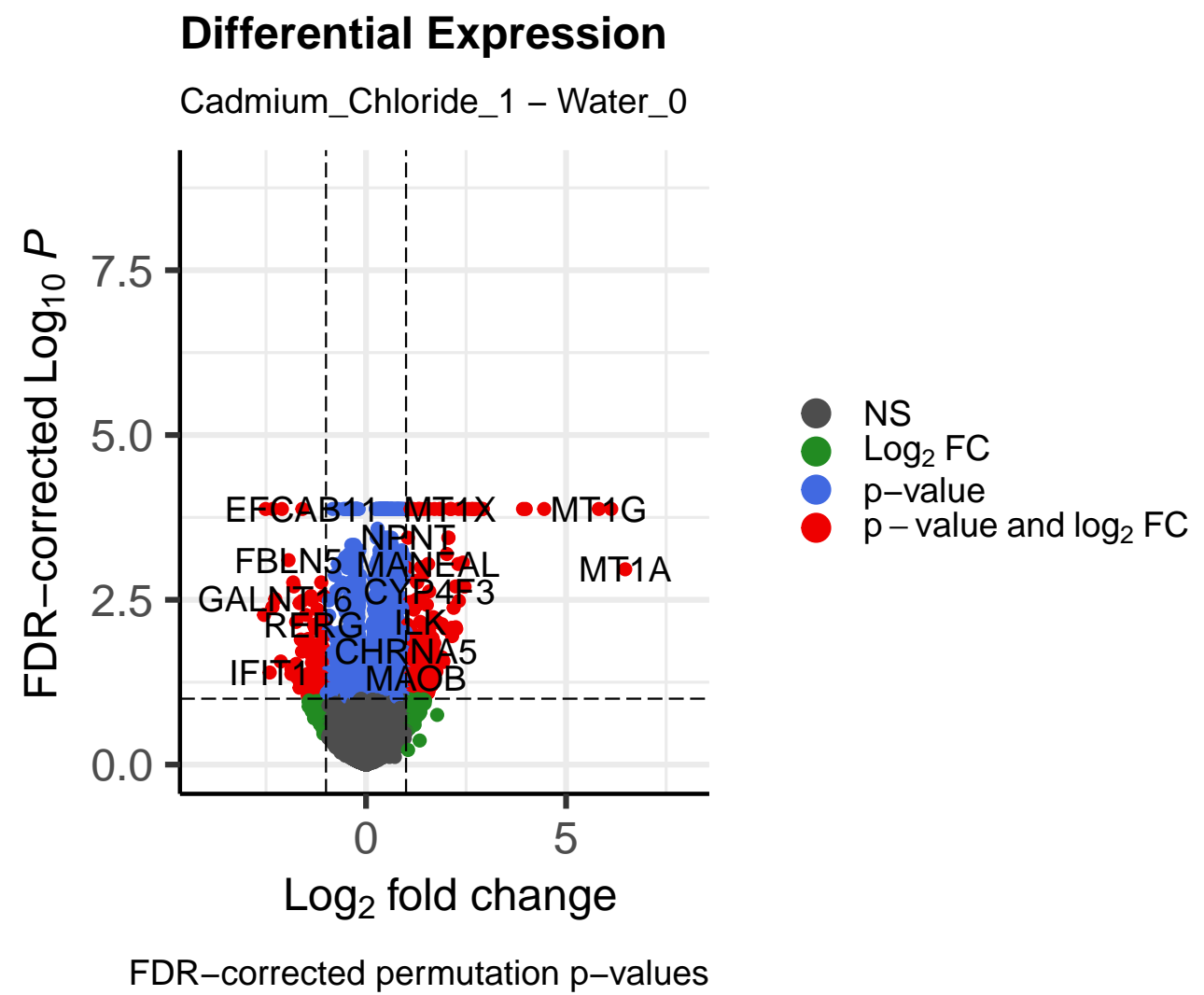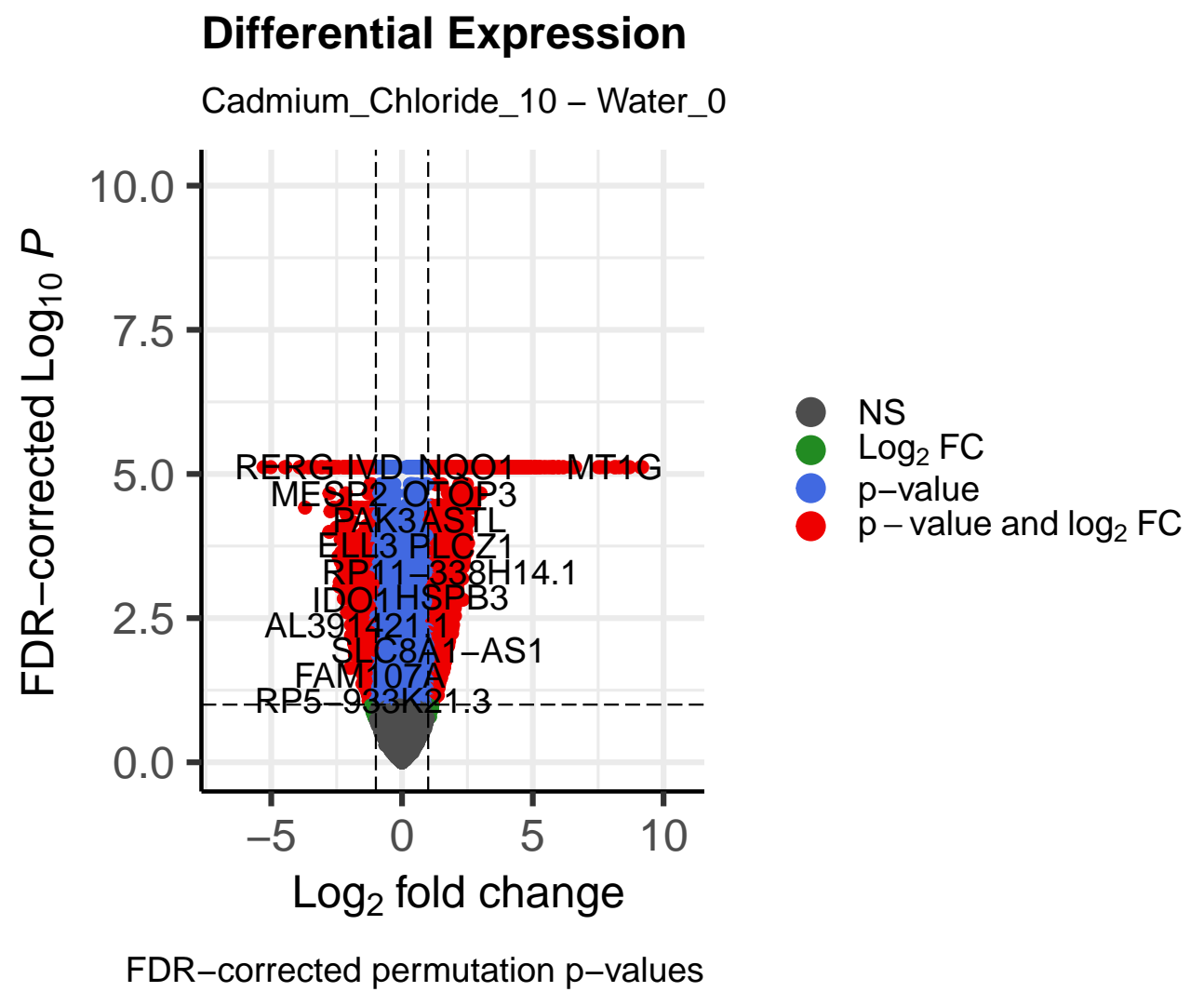

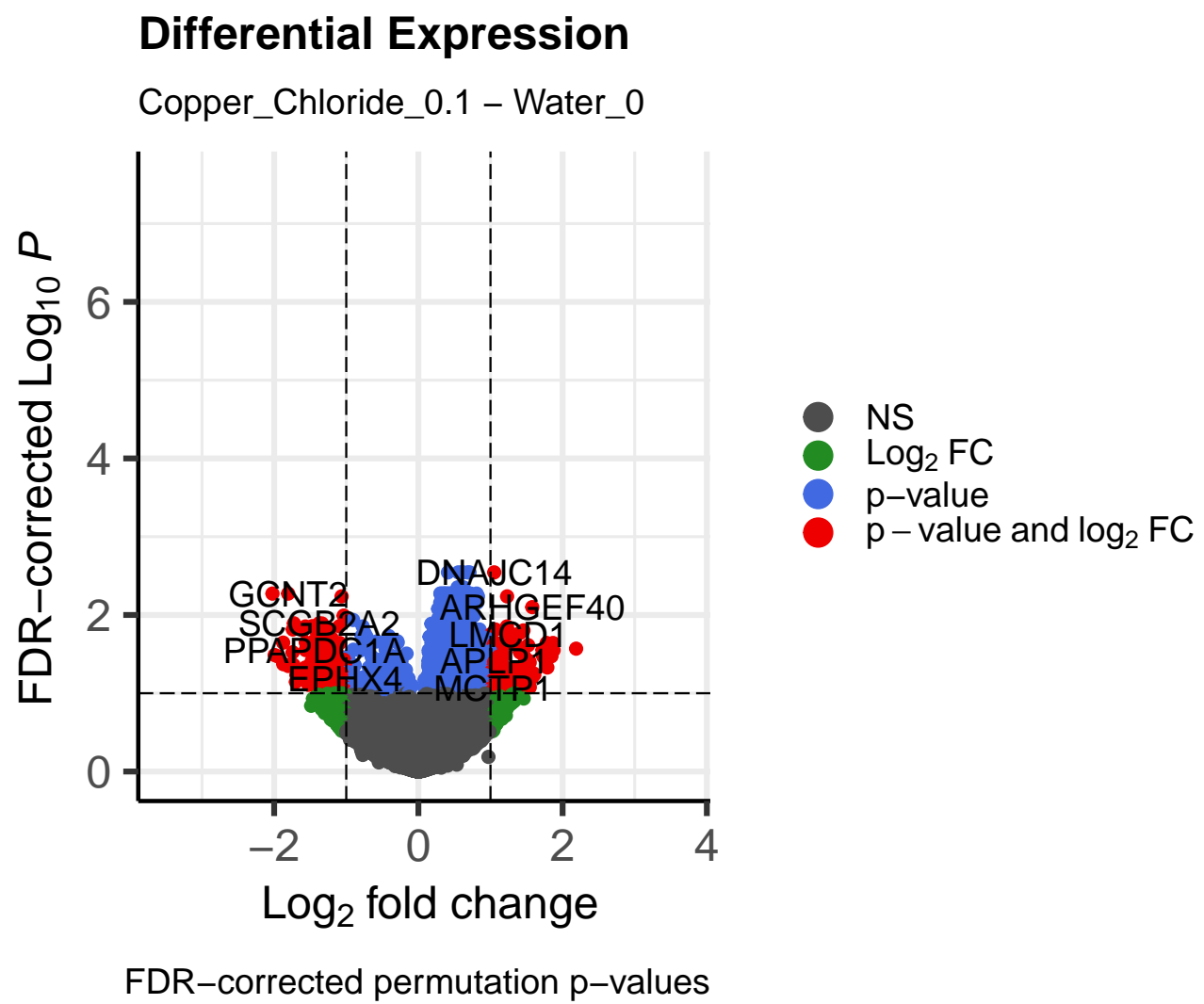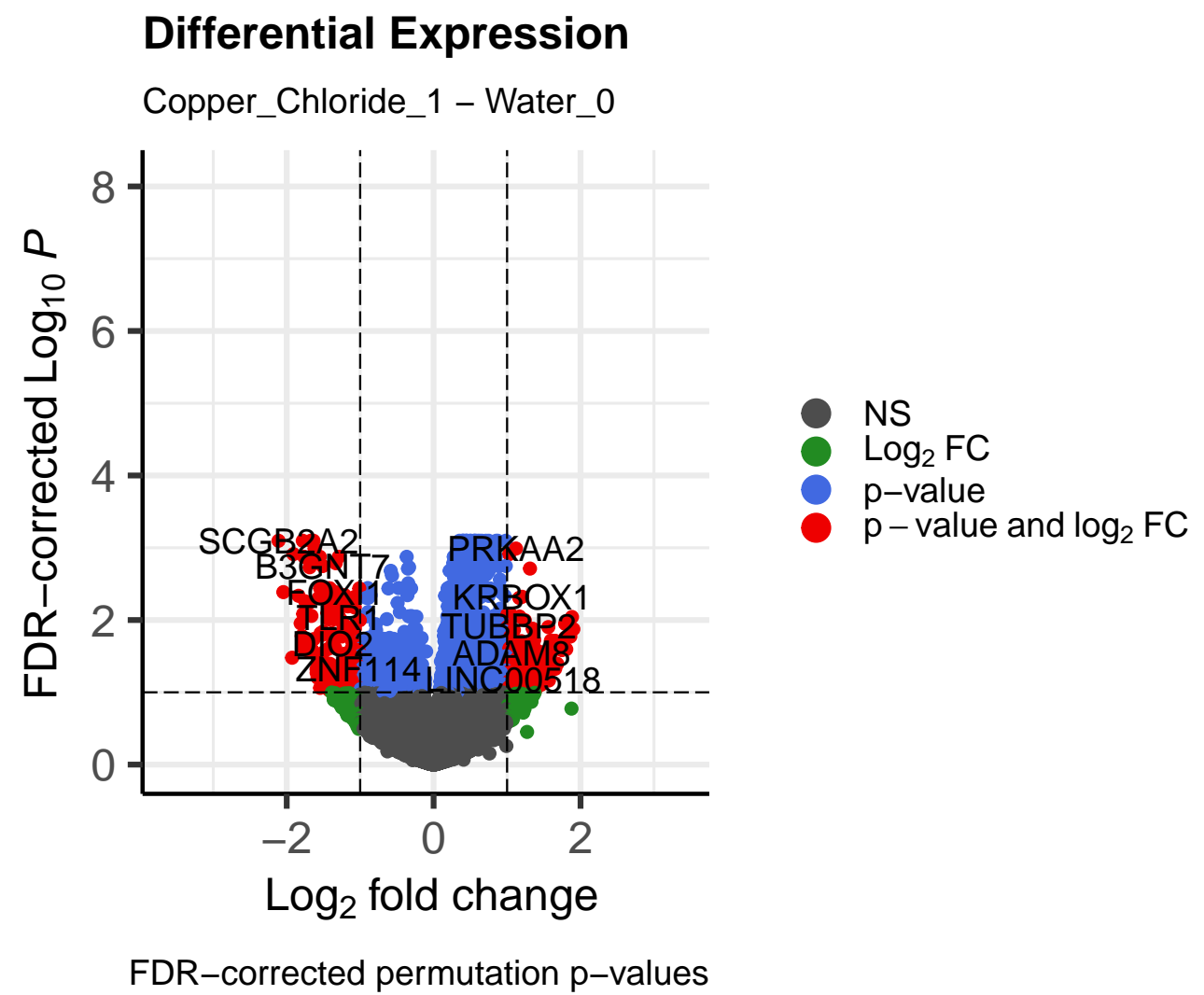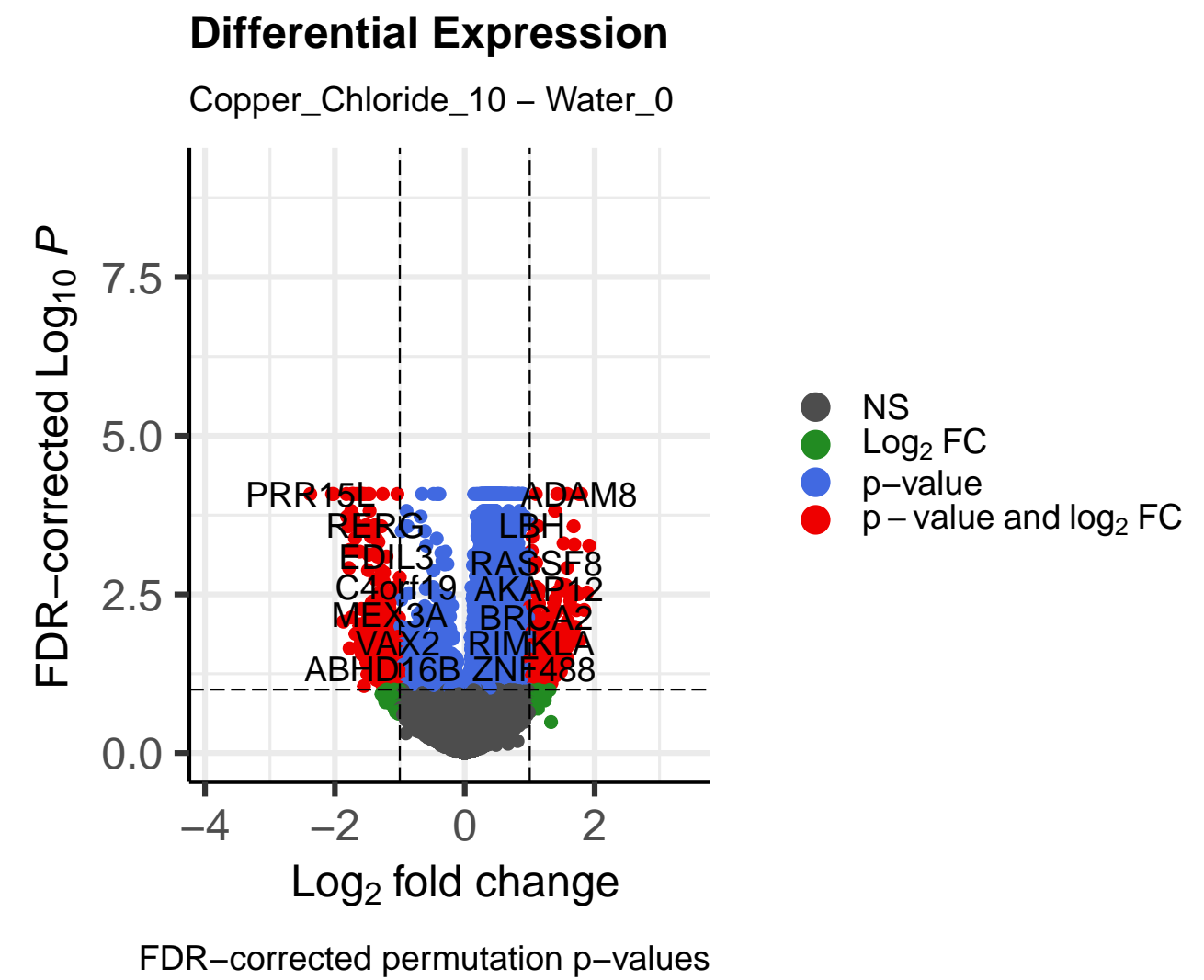

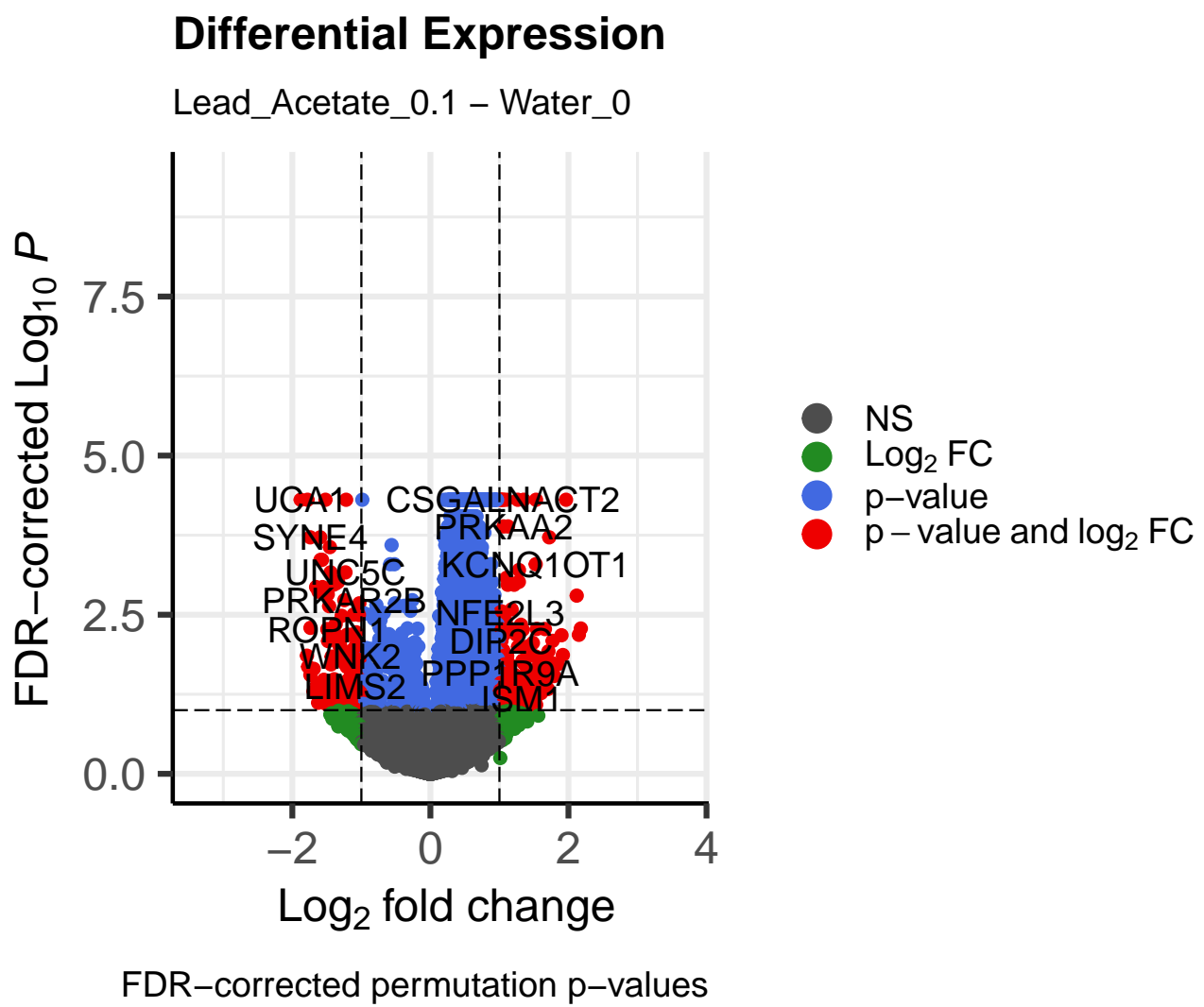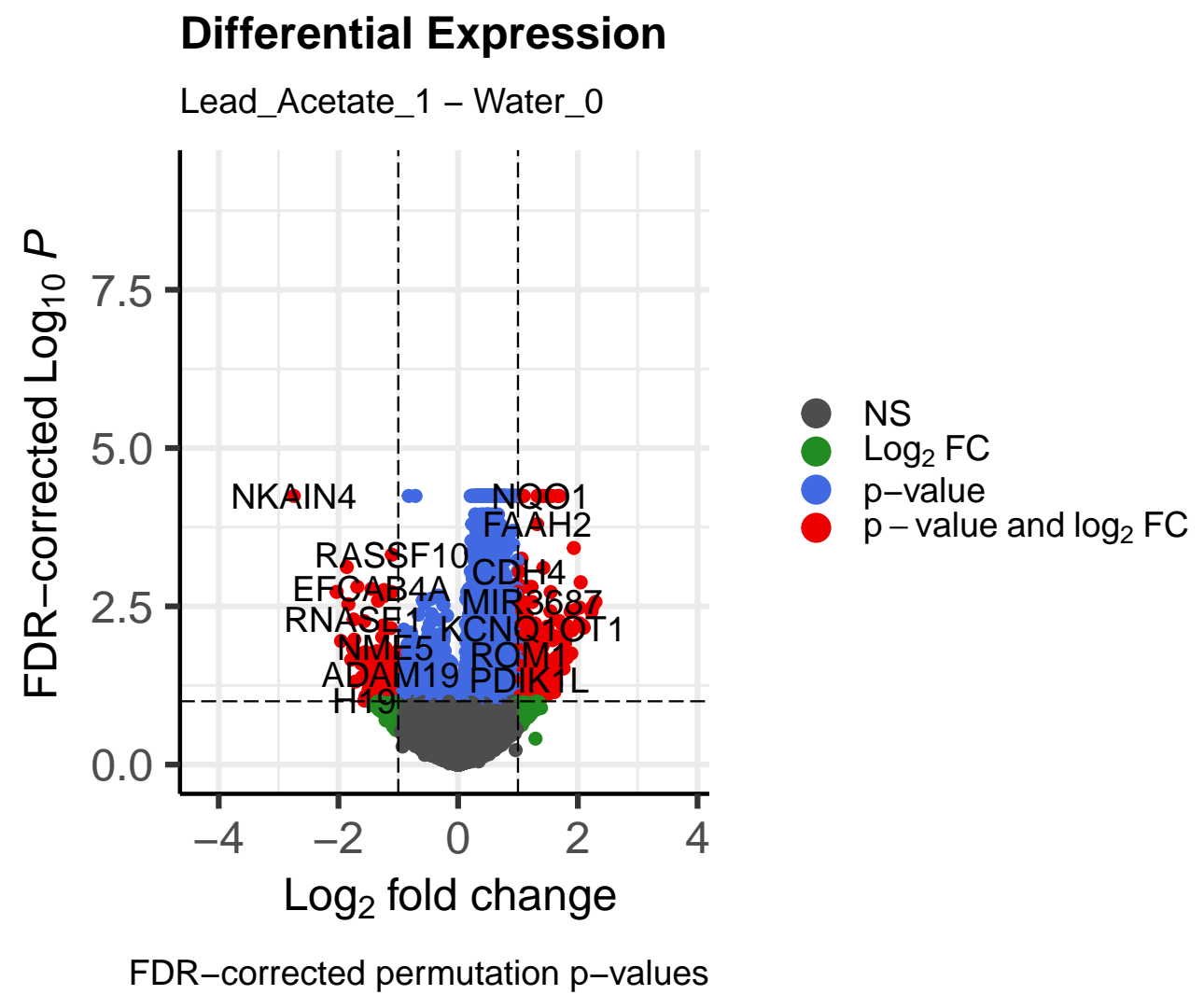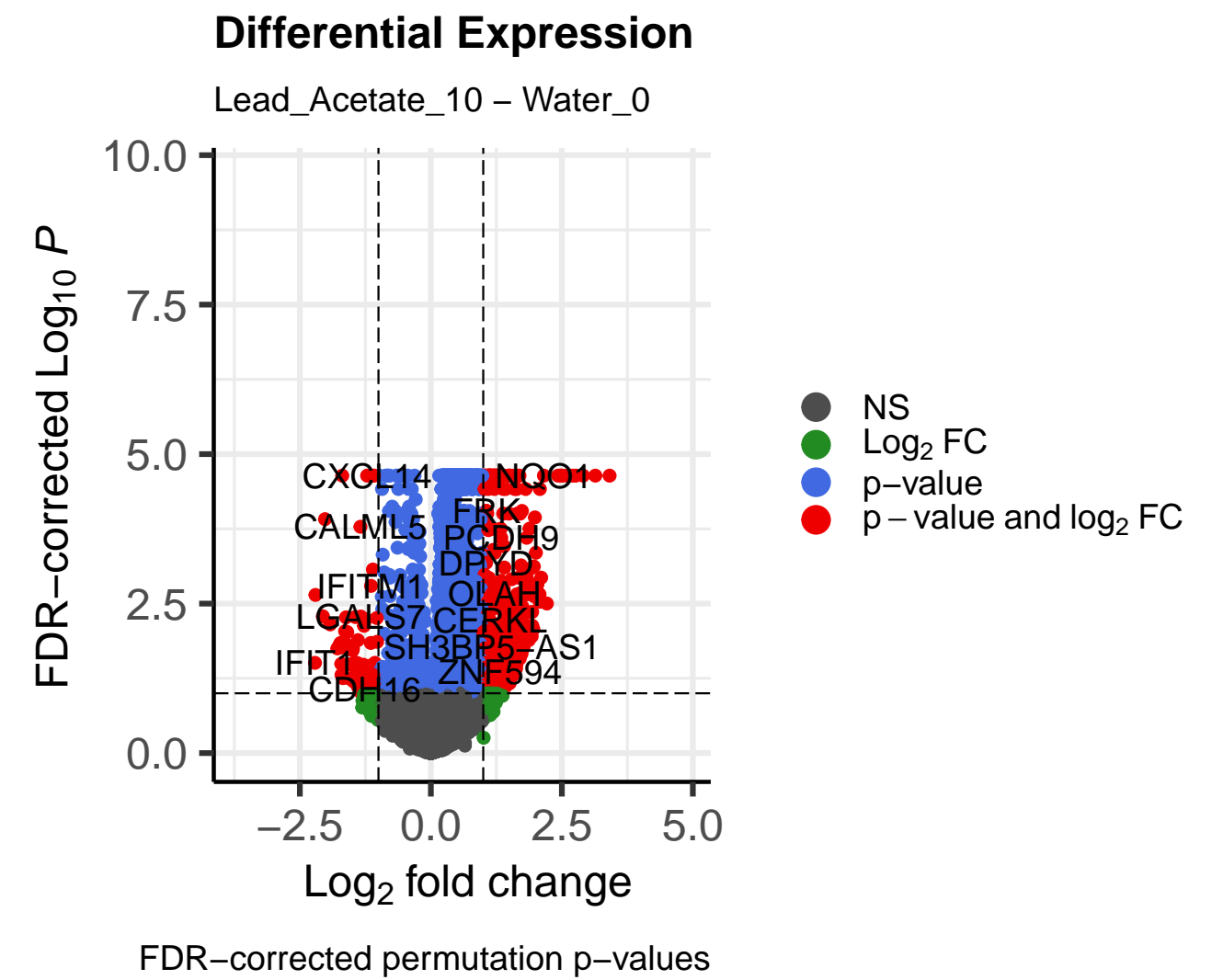

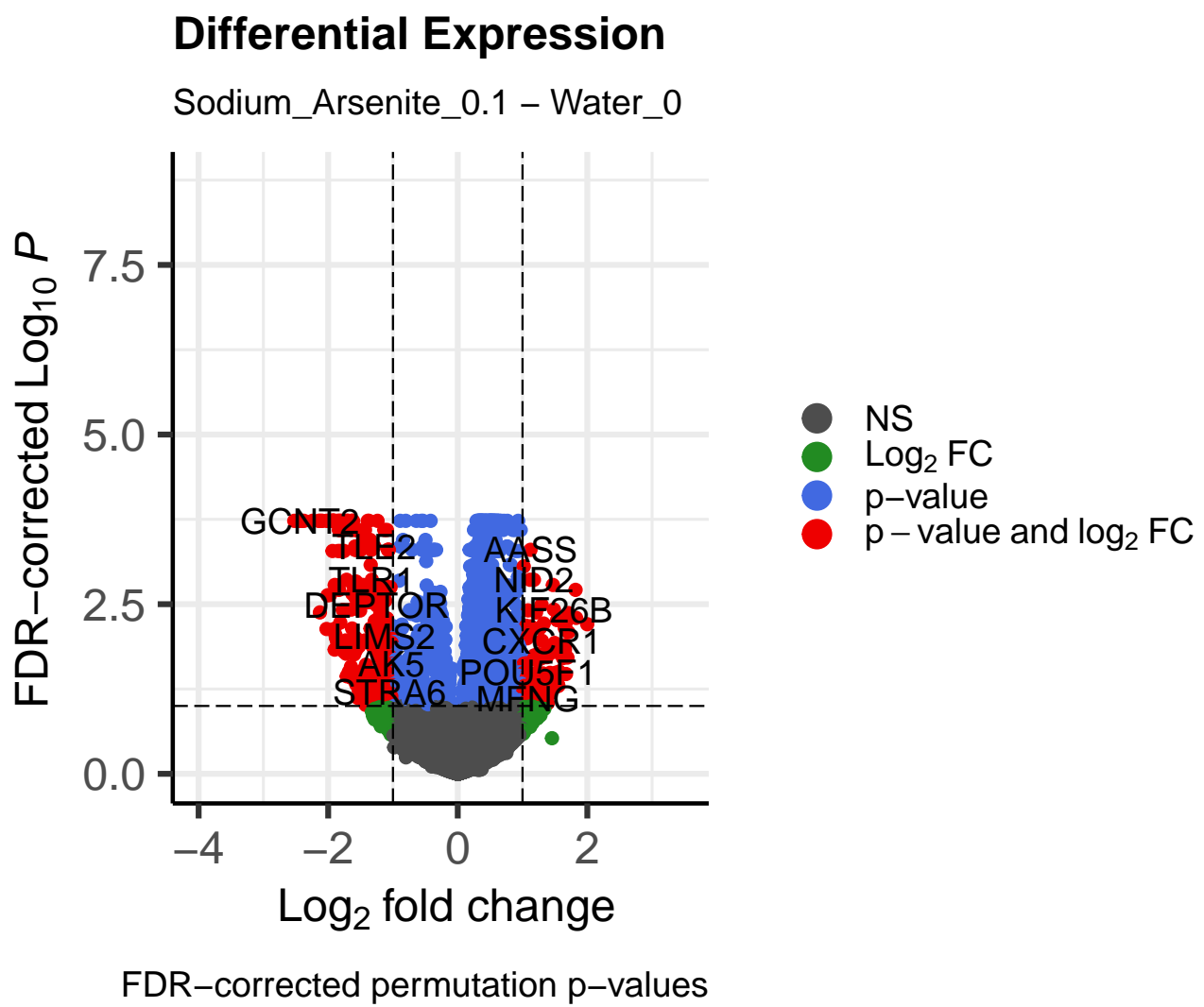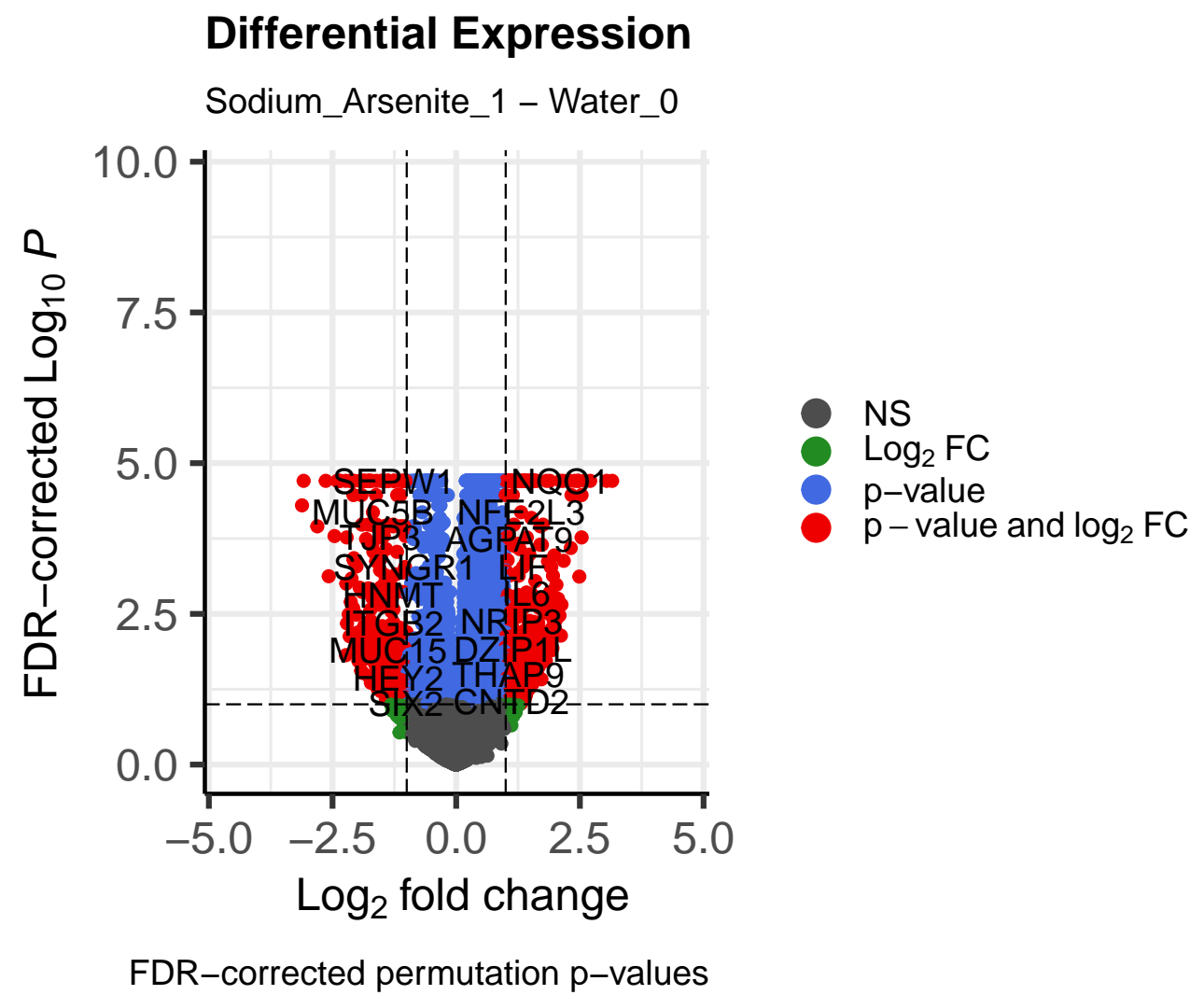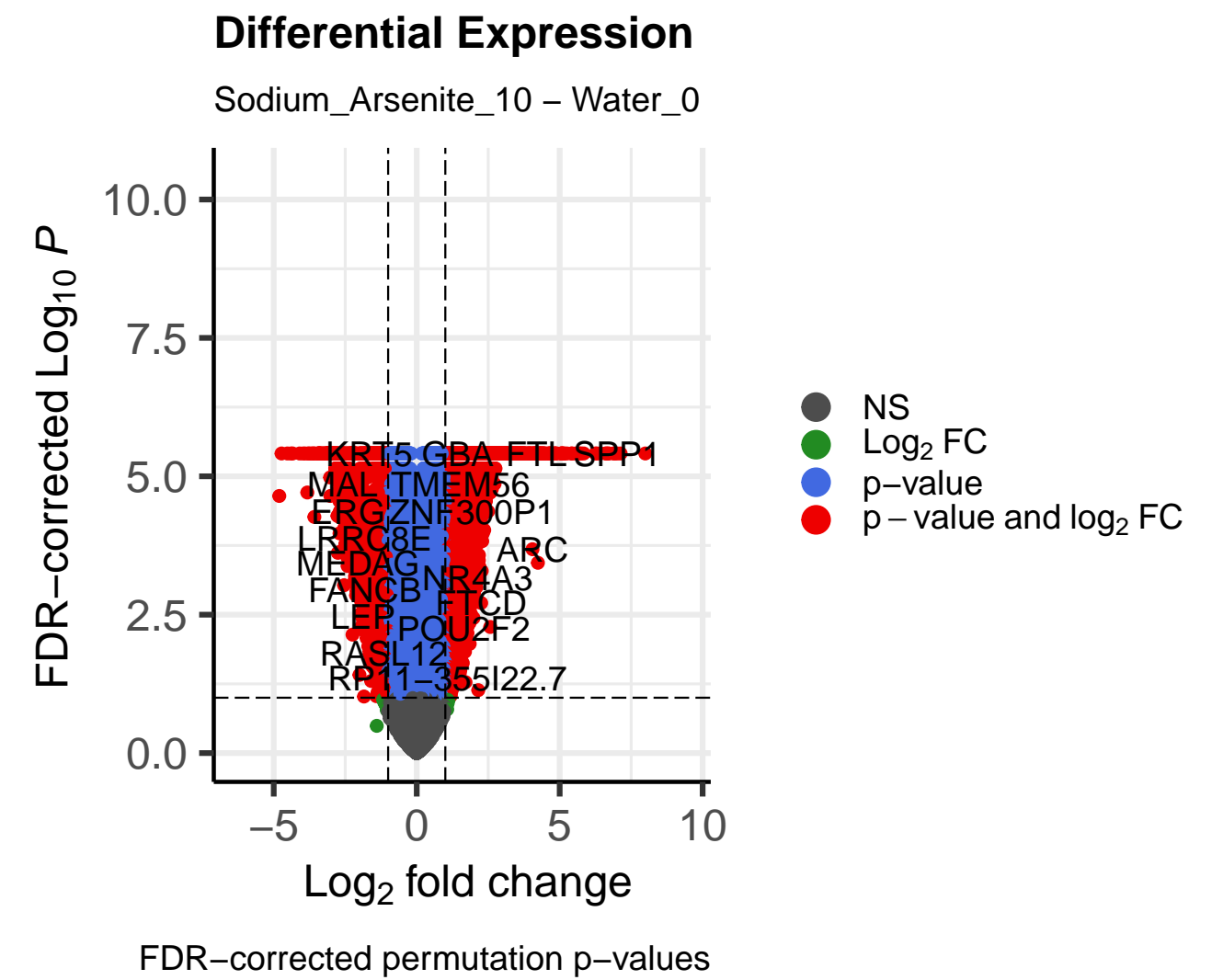

Supplement: Supplement 2 — Differential gene expression between each aggregated chemical dose and control was calculated using limma-Voom and empirical Bayes quality and precision weighted generalized linear modeling. Vertical dotted-black lines mark a log2 fold-change cutoff of >|2|. Horizontal dotted-black lines mark a false discovery rate (FDR) adjusted p-value of ≤0.05. [file media-2.pdf]

Luminal Progenitor

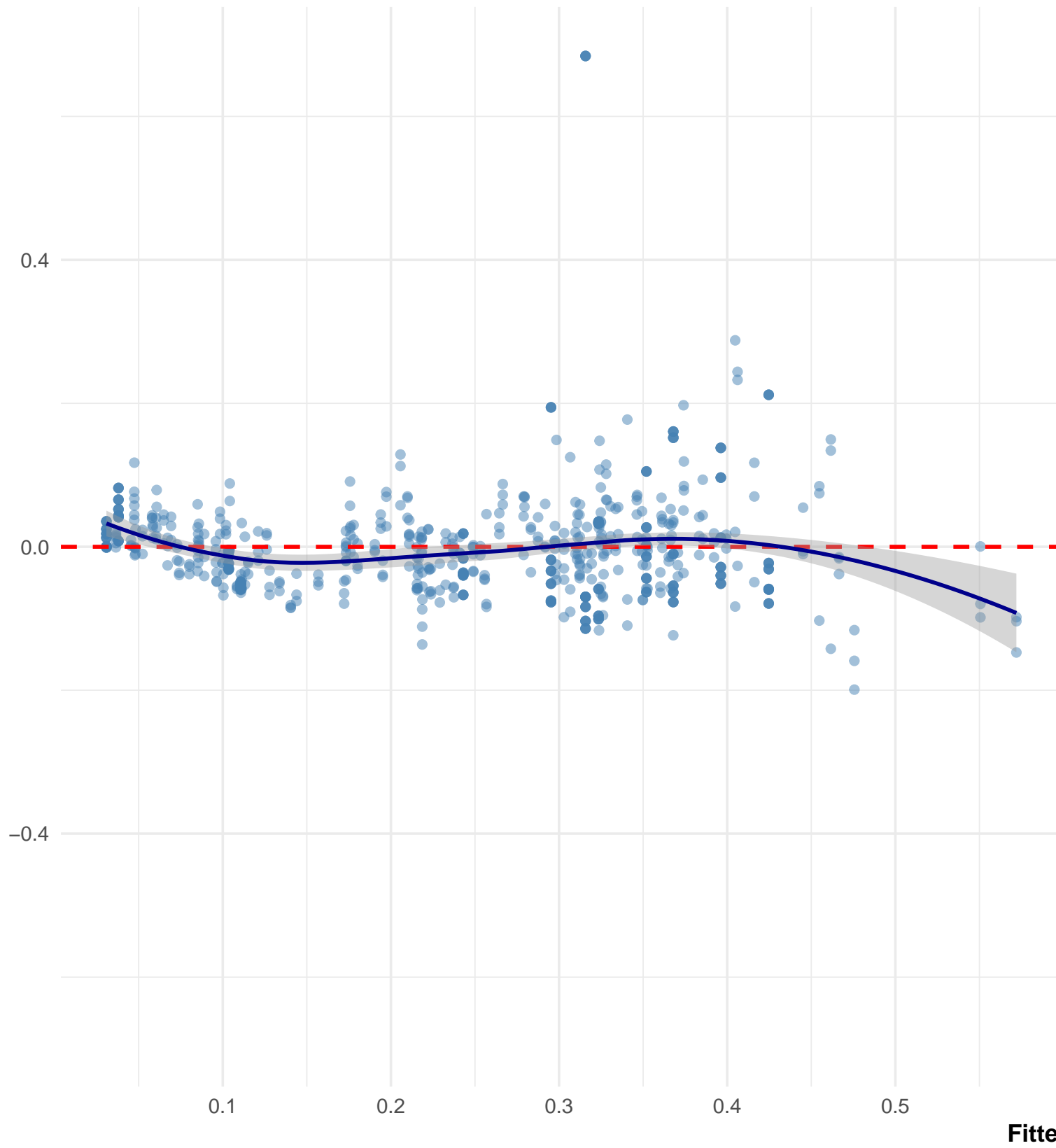

Myoepithelial

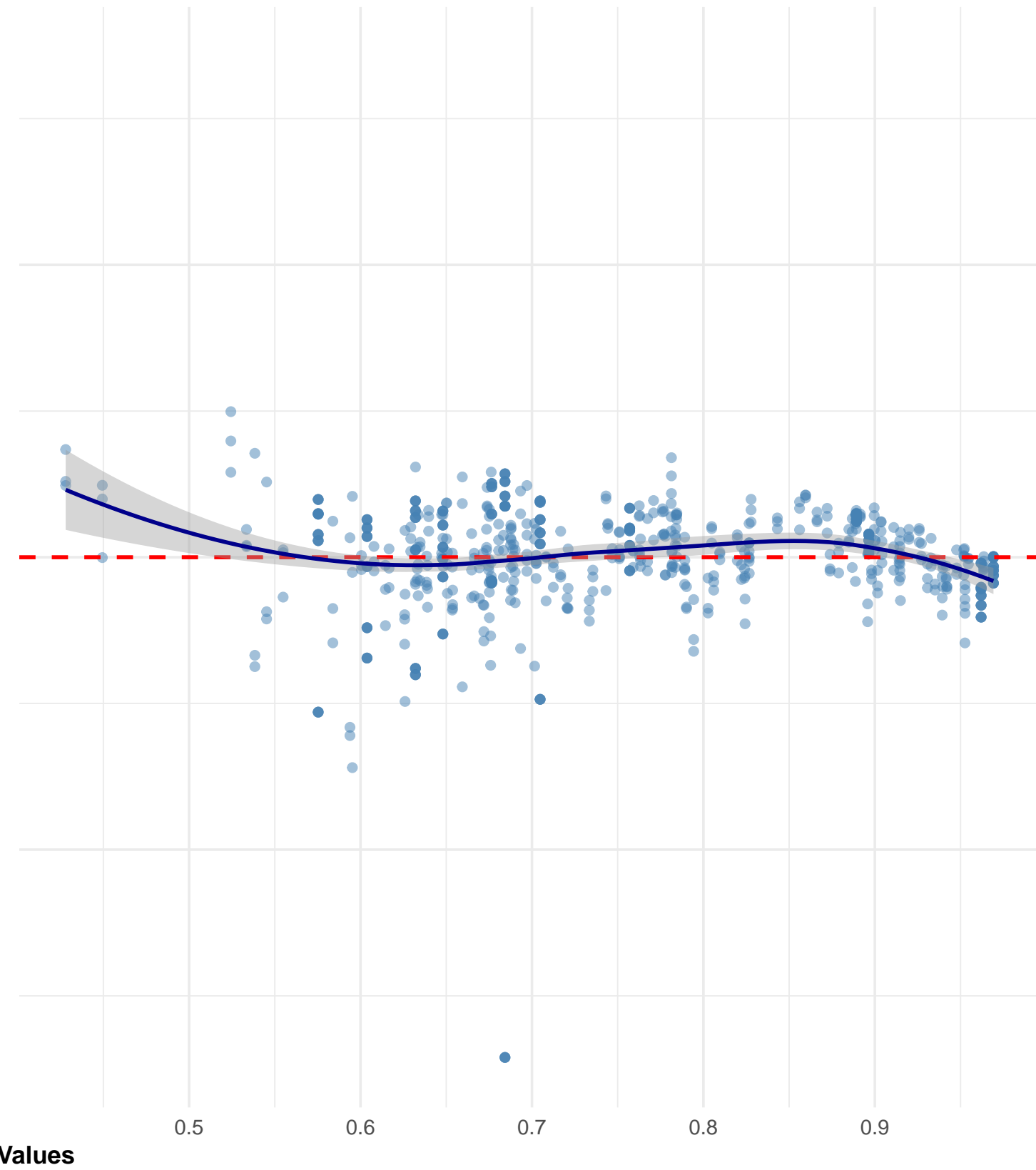

Supplement: Supplement 4 — Residuals versus fitted values for luminal progenitor (left) and myoepithelial (right) cell-type proportion models. Points represent individual observations colored by treatment. Horizontal dashed line at zero. Blue curve shows LOESS smoothing of residuals. Cell-type proportions sum to 1.0. [file media-4.pdf]
