# Supplementary material for: Exploring the Influence of Chemical Exposures in Breast Cancer Disparities: High-Throughput Transcriptomic Analysis in Normal Breast Cells from Diverse Donors
Source: bioRxiv. 2026 Feb 24:2026.02.23.707203. Preprint. [Version 1] doi: 10.64898/2026.02.23.707203 (PMC13160138; doi:10.64898/2026.02.23.707203)
Supplement: Supplement 3 — Differential gene expression between each sample-specific chemical dose and control was calculated using limma-Voom and empirical Bayes quality and precision weighted generalized linear modeling. Vertical dotted-black lines mark a log2 fold-change cutoff of >|2|. Horizontal dotted-black lines mark a FDR adjusted p-value of ≤0.05. [file media-3.pdf]

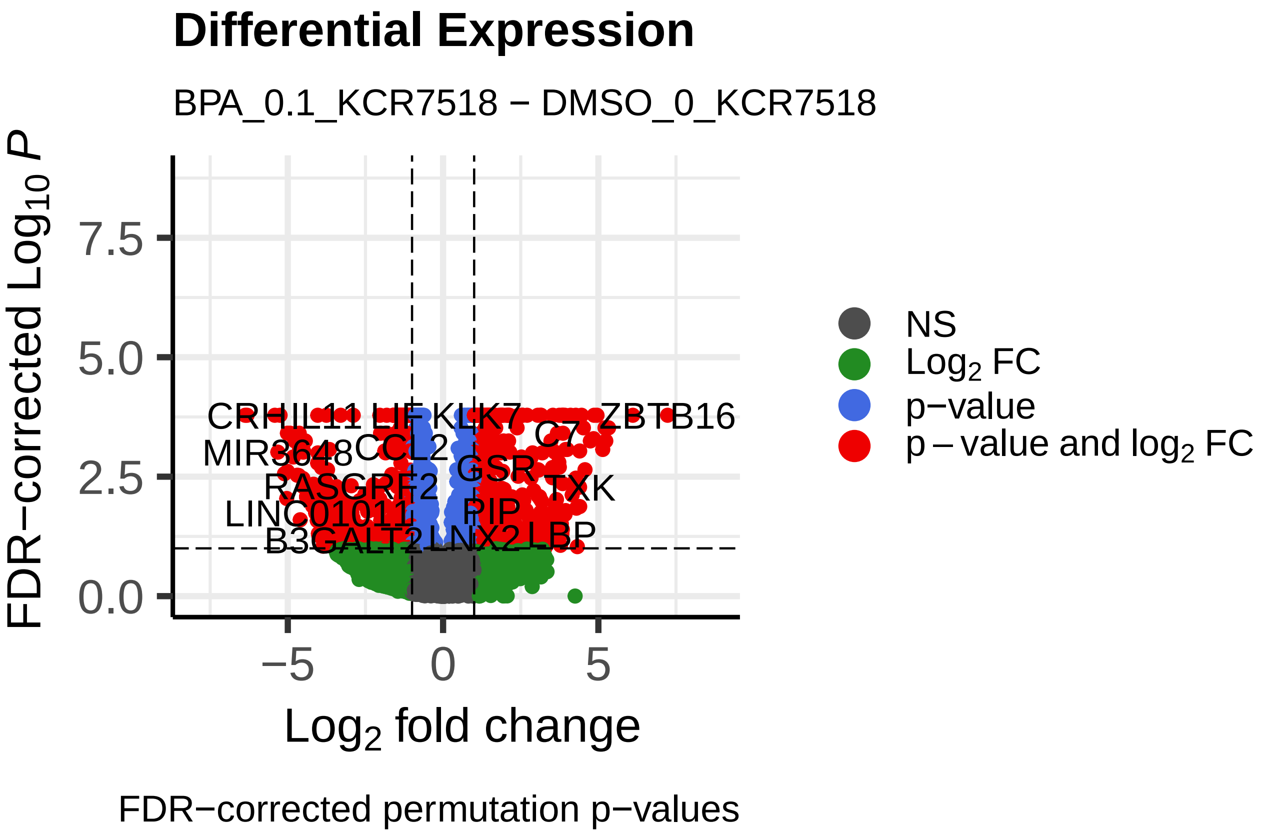
Differential Expression
BPA_0.1_KCR7518 − DMSO_0_KCR7518
FDR−corrected $Log_{10}$ $P$
Log₂ fold change
NS
$Log_2$ FC
p−value
p − value and $log_2$ FC
CRHR1
IL11
LIF
KLK7
ZBTB16
MIR3648
CCL2
C7
GSR
RASGRF2
TXK
LINC01011
PIP
B3GALT2
LNX2
LBP
FDR−corrected permutation p−values

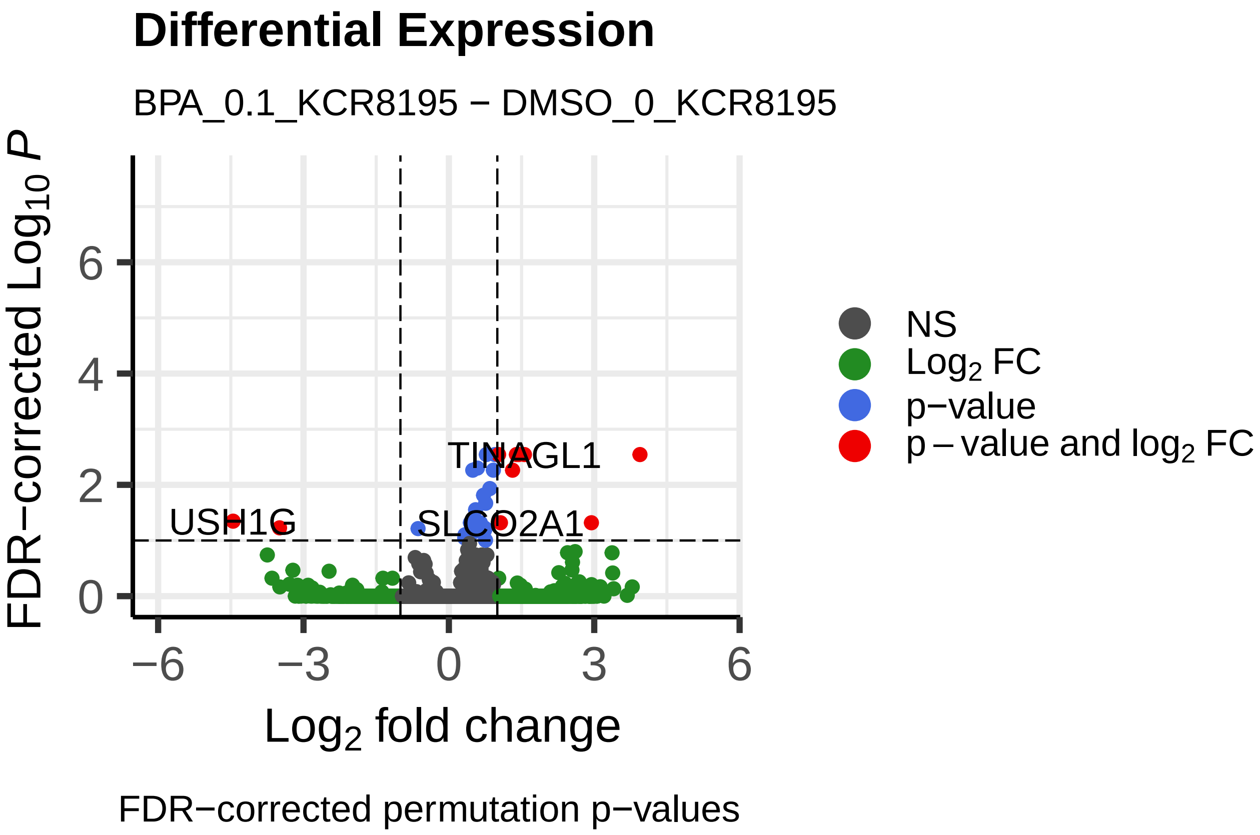
Differential Expression
BPA_0.1_KCR8195 − DMSO_0_KCR8195
FDR−corrected $Log_{10}$ $P$
$Log_2$ fold change
NS
$Log_2$ FC
p−value
p − value and $log_2$ FC
TINAGL1
USH1G
SLCO2A1
FDR−corrected permutation p−values

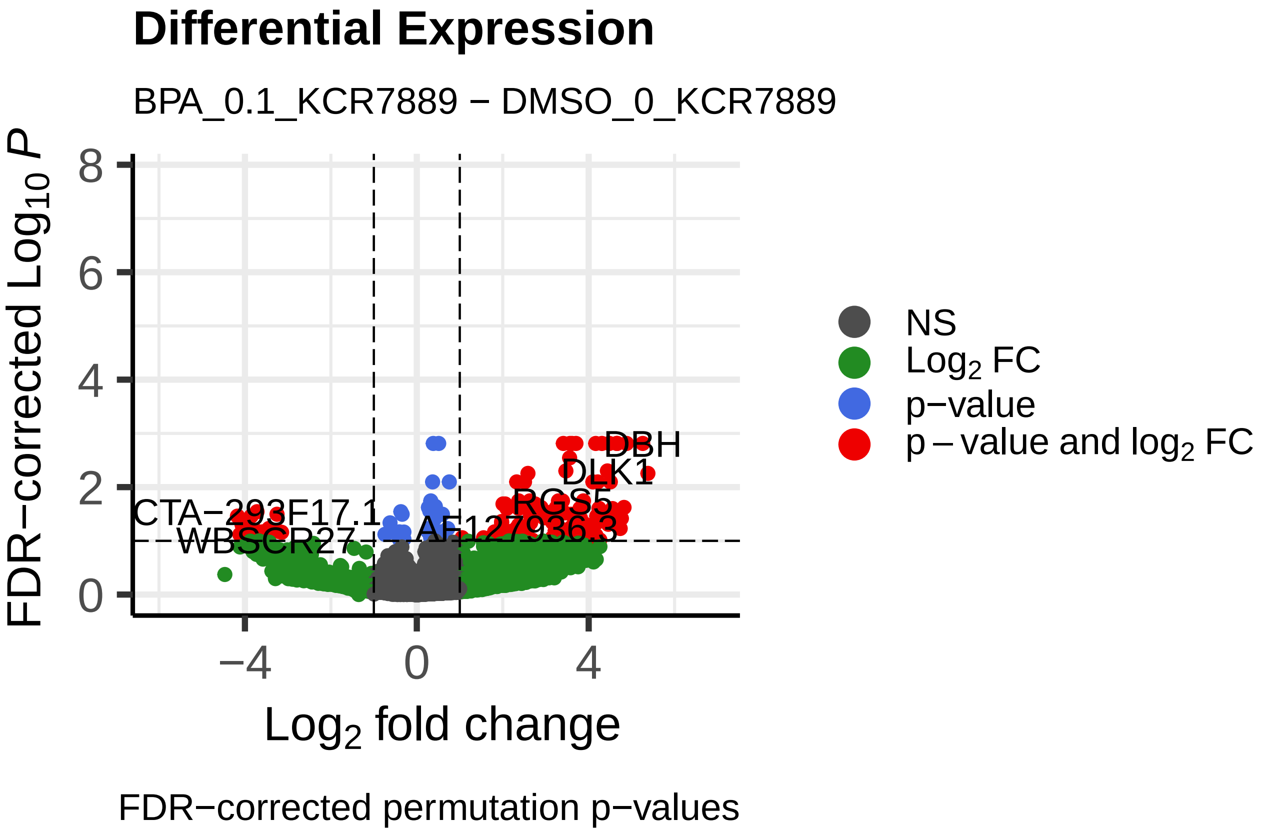
Differential Expression
BPA_0.1_KCR7889 − DMSO_0_KCR7889
FDR−corrected $Log_{10}$ $P$
$Log_2$ fold change
NS
$Log_2$ FC
p−value
p − value and $log_2$ FC
DBH
DLK1
RGS5
CTA−293F17.1
WBSCR27
AF127936.3
FDR−corrected permutation p−values

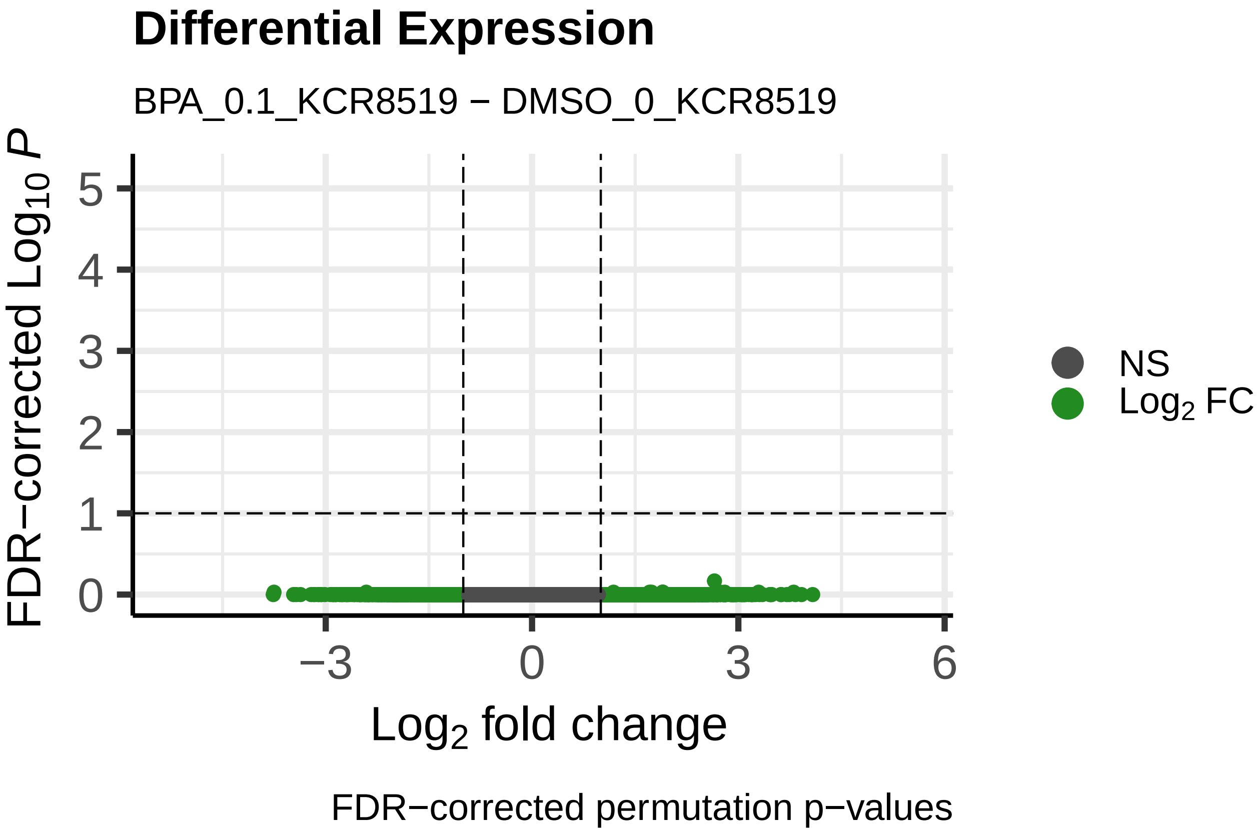
Differential Expression
BPA_0.1_KCR8519 − DMSO_0_KCR8519
FDR−corrected $Log_{10}$ $P$
$Log_2$ fold change
NS
$Log_2$ FC
FDR−corrected permutation p−values

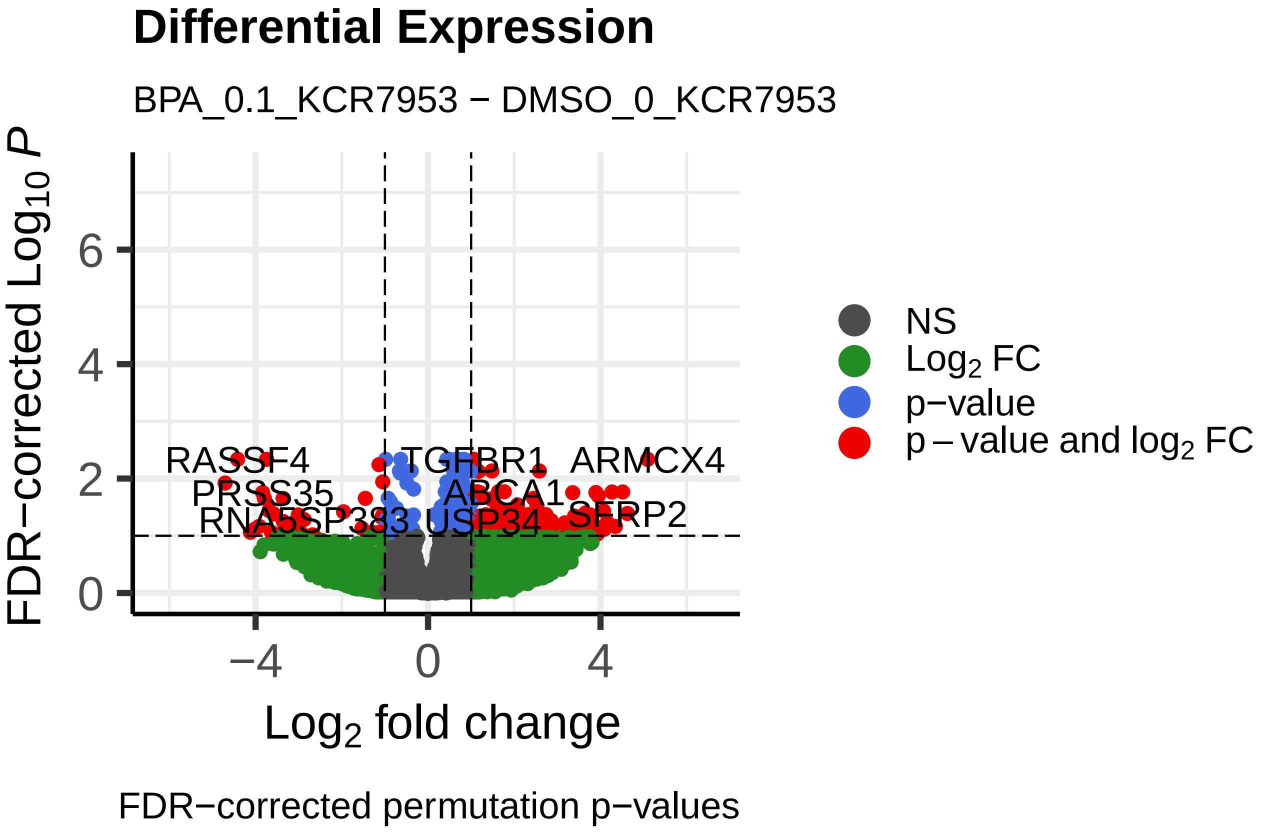
Differential Expression
BPA_0.1_KCR7953 − DMSO_0_KCR7953
FDR−corrected $Log_{10}$ $P$
$Log_2$ fold change
NS
$Log_2$ FC
p−value
p − value and $log_2$ FC
RASSF4
TGFBR1
ARMCX4
PRSS35
ABCA1
USP34
SFRP2
FDR−corrected permutation p−values

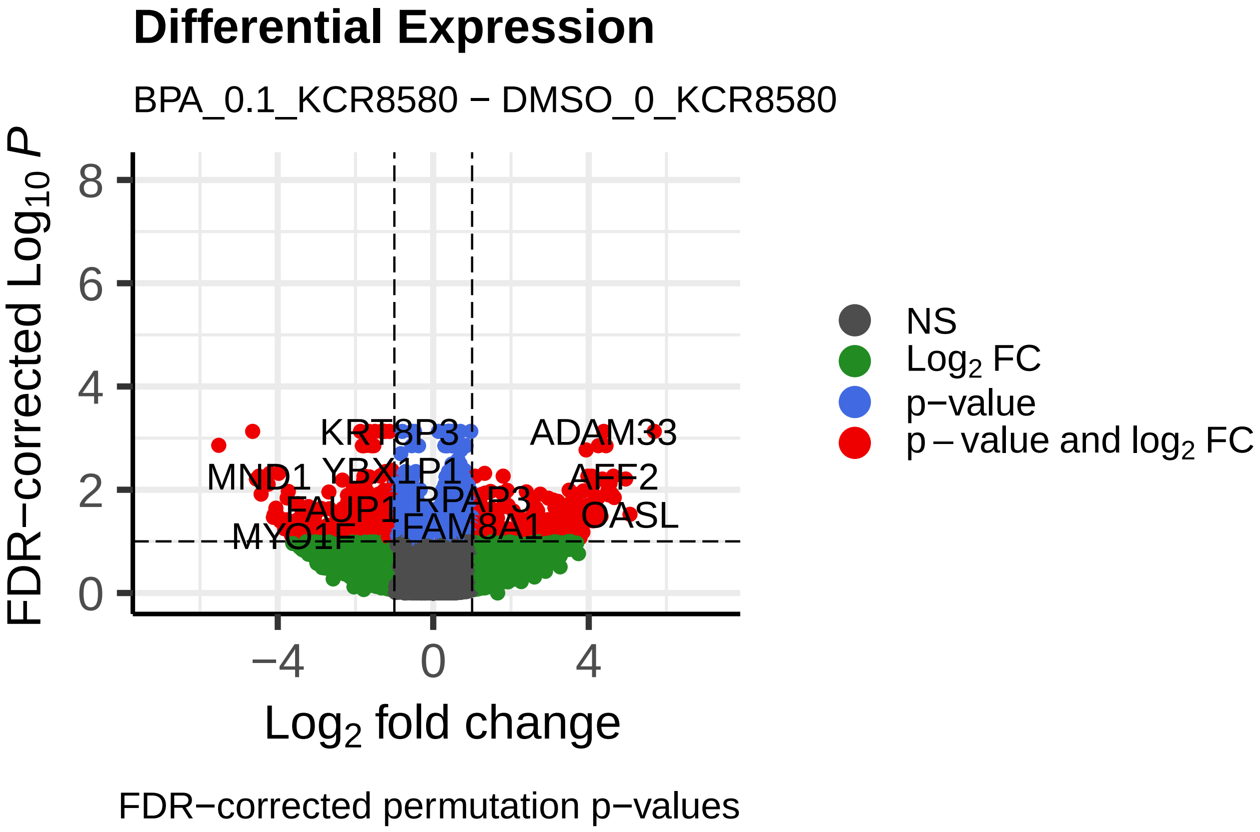
Differential Expression
BPA_0.1_KCR8580 − DMSO_0_KCR8580
FDR−corrected $Log_{10}$ $P$
$Log_2$ fold change
NS
$Log_2$ FC
p−value
p − value and $log_2$ FC
ADAM33
MND1
AFF2
RPAP3
OASL
MYO1F
FAM8A1
FDR−corrected permutation p−values

## Differential Expression

BPA_1_KCR7518 − DMSO_0_KCR7518

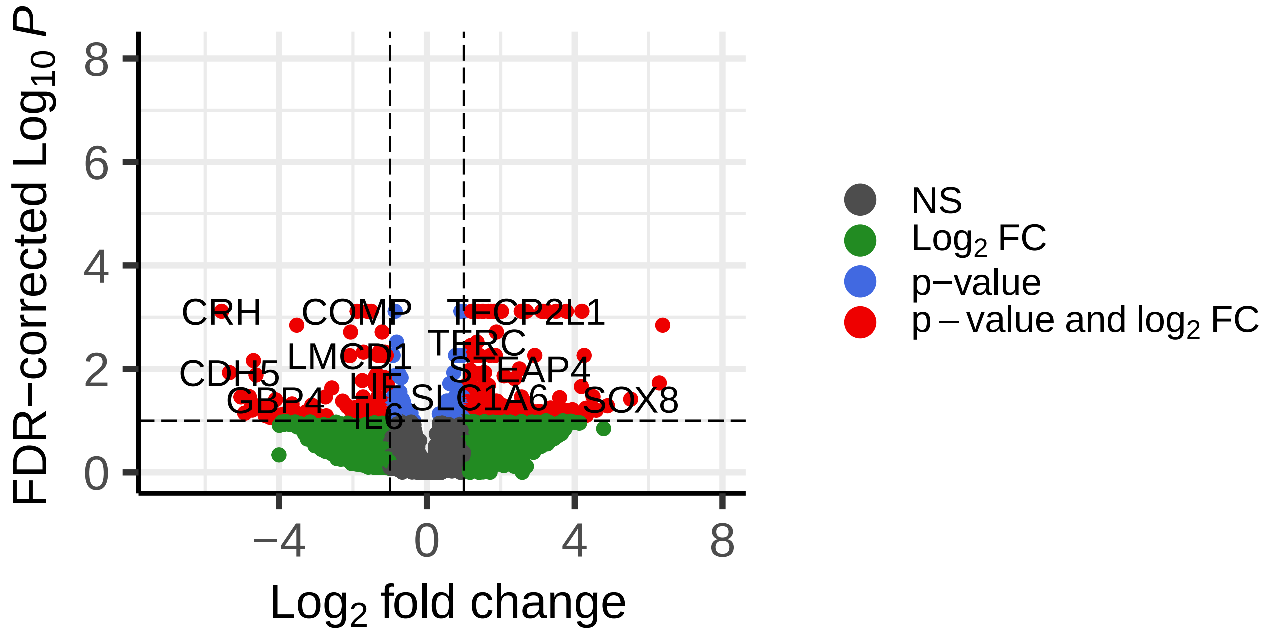


FDR−corrected permutation p−values

## Differential Expression

BPA_1_KCR8195 − DMSO_0_KCR8195

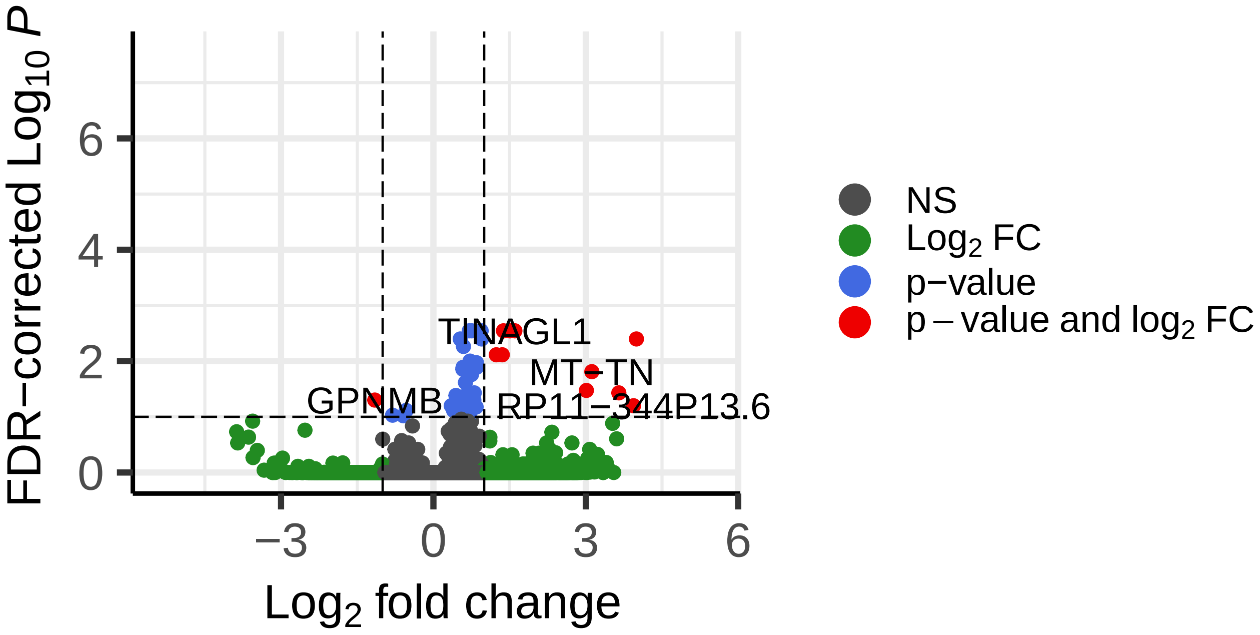


FDR−corrected permutation p−values

## Differential Expression

BPA_1_KCR7889 − DMSO_0_KCR7889

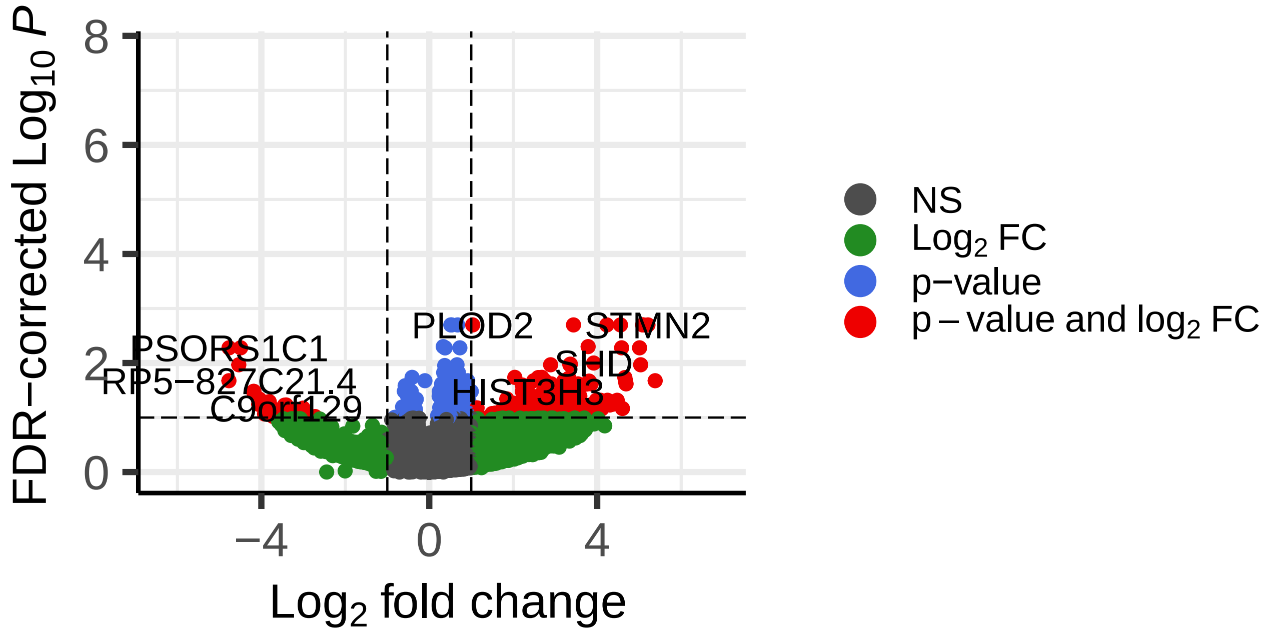


FDR−corrected permutation p−values

## Differential Expression

BPA_1_KCR8519 − DMSO_0_KCR8519

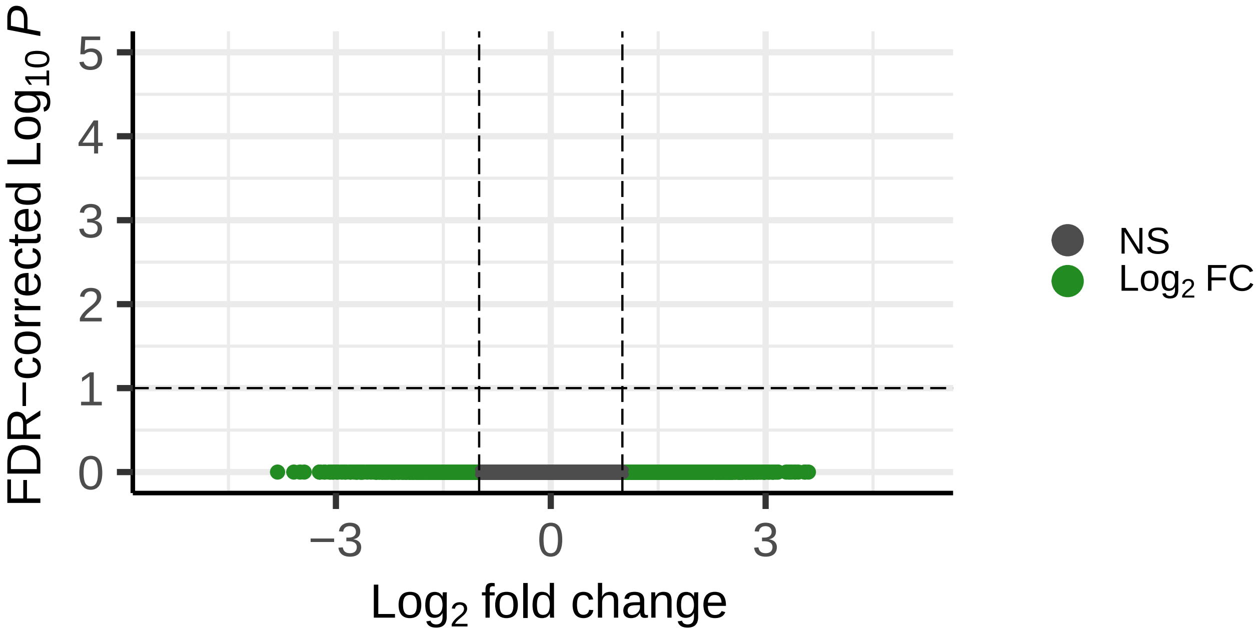


FDR−corrected permutation p−values

## Differential Expression

BPA_1_KCR7953 − DMSO_0_KCR7953

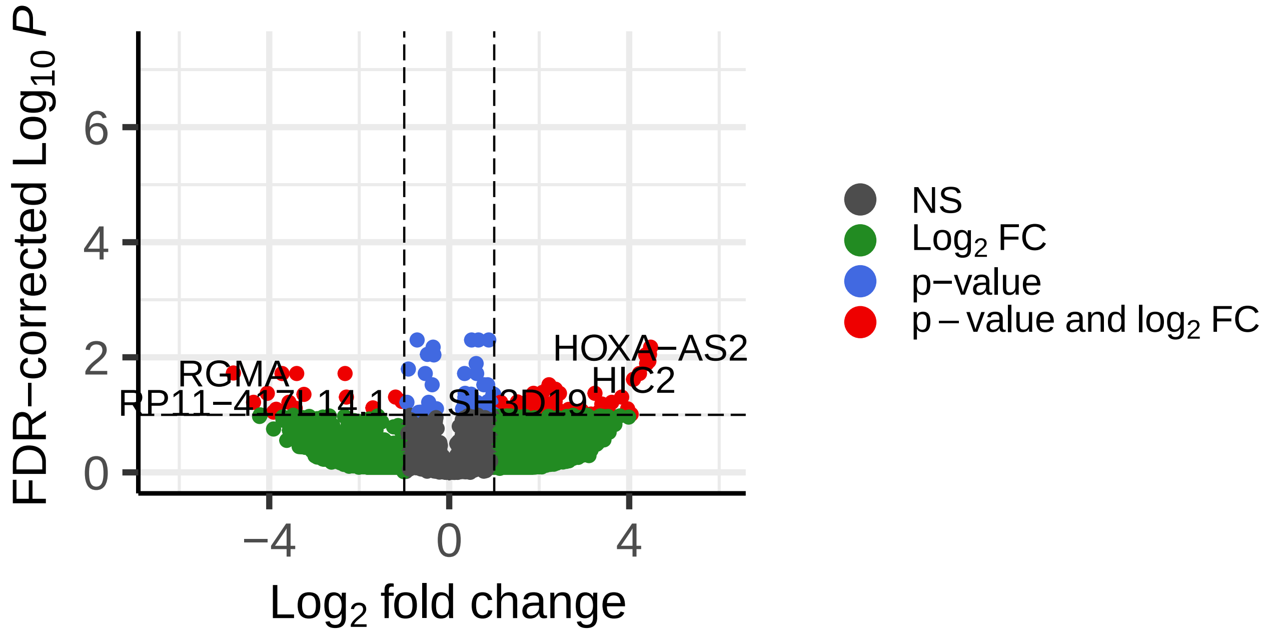


FDR−corrected permutation p−values

## Differential Expression

BPA_1_KCR8580 − DMSO_0_KCR8580

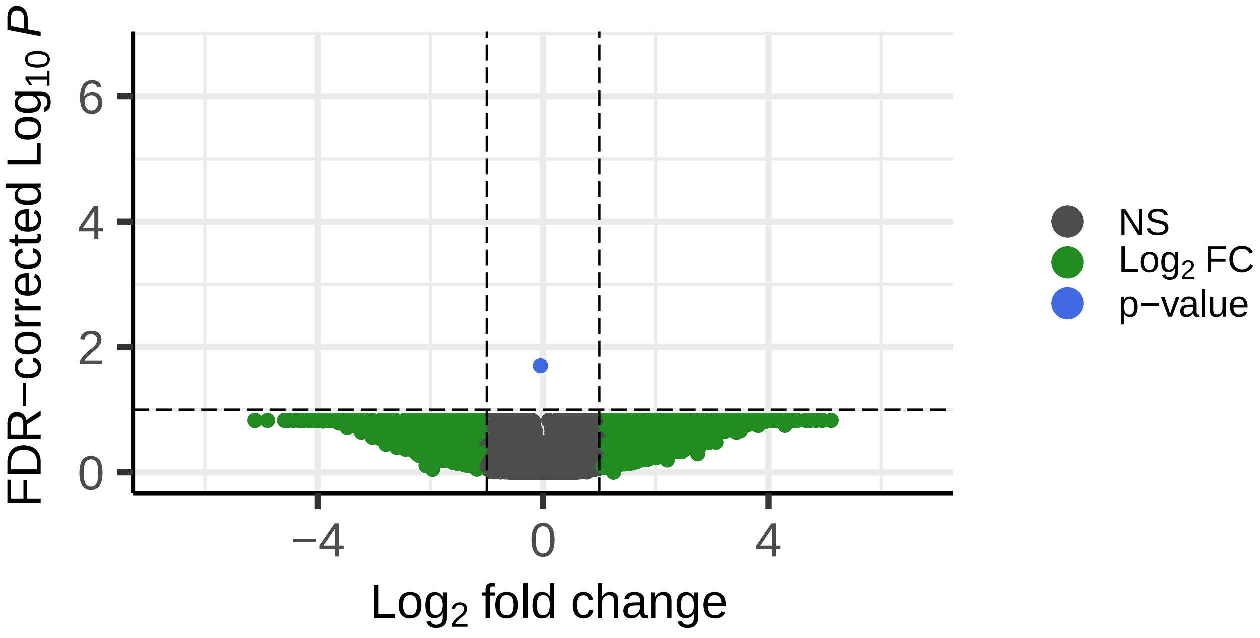


FDR−corrected permutation p−values

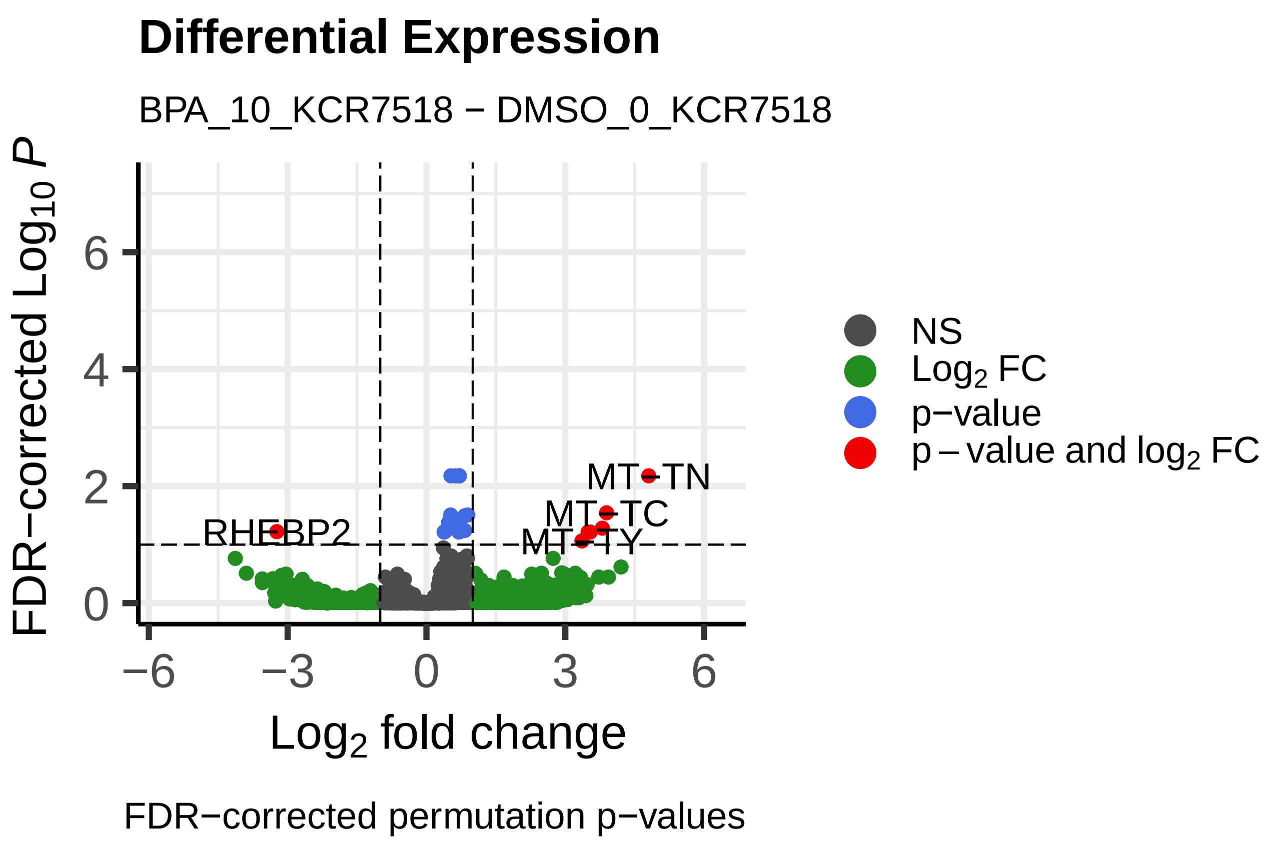
Differential Expression
BPA_10_KCR7518 − DMSO_0_KCR7518
FDR−corrected Log10 P
Log2 fold change
NS
Log2 FC
p−value
p − value and log2 FC
MT−TN
MT−TC
MT−TY
RHEBP2
FDR−corrected permutation p−values

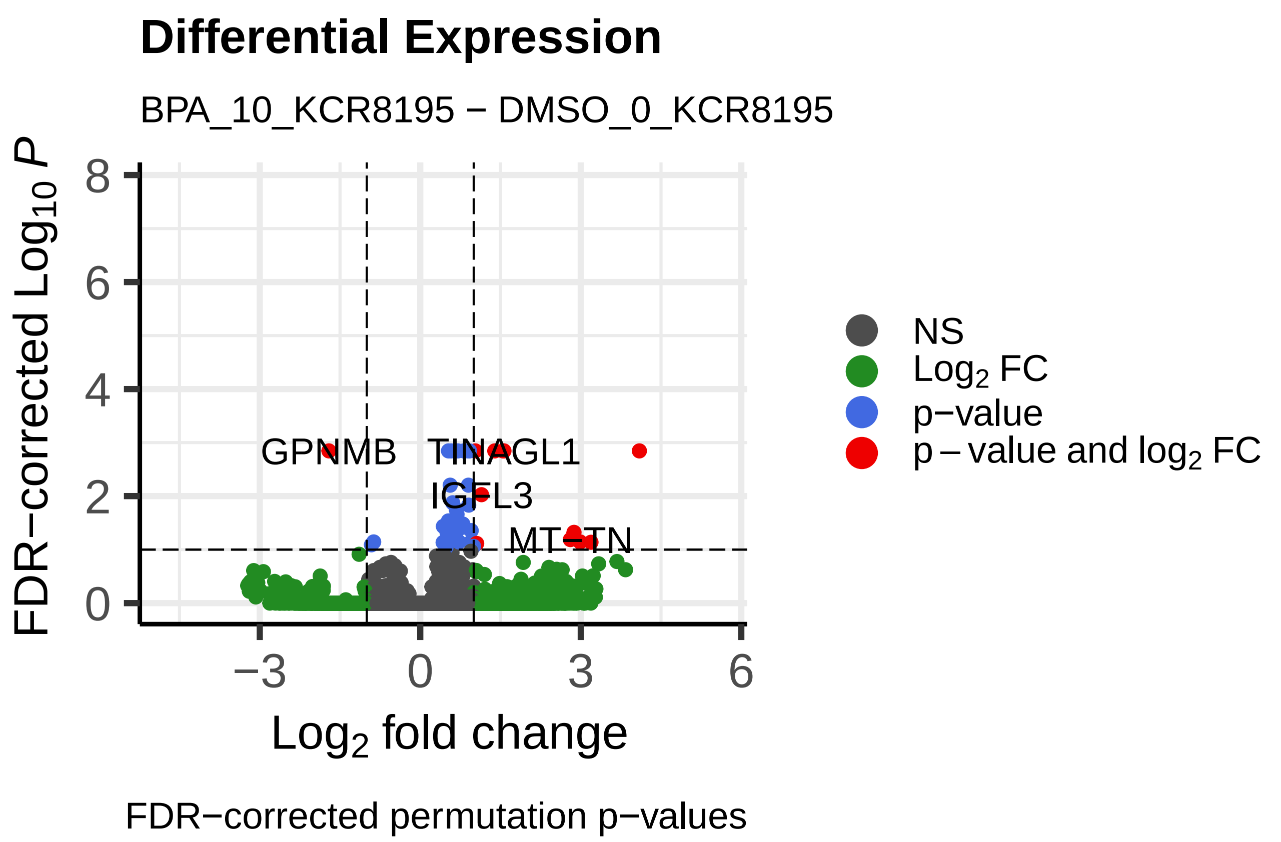
Differential Expression
BPA_10_KCR8195 − DMSO_0_KCR8195
FDR−corrected Log10 P
Log2 fold change
NS
Log2 FC
p−value
p − value and log2 FC
GPNMB
TINAGL1
IGFL3
MT−TN
FDR−corrected permutation p−values

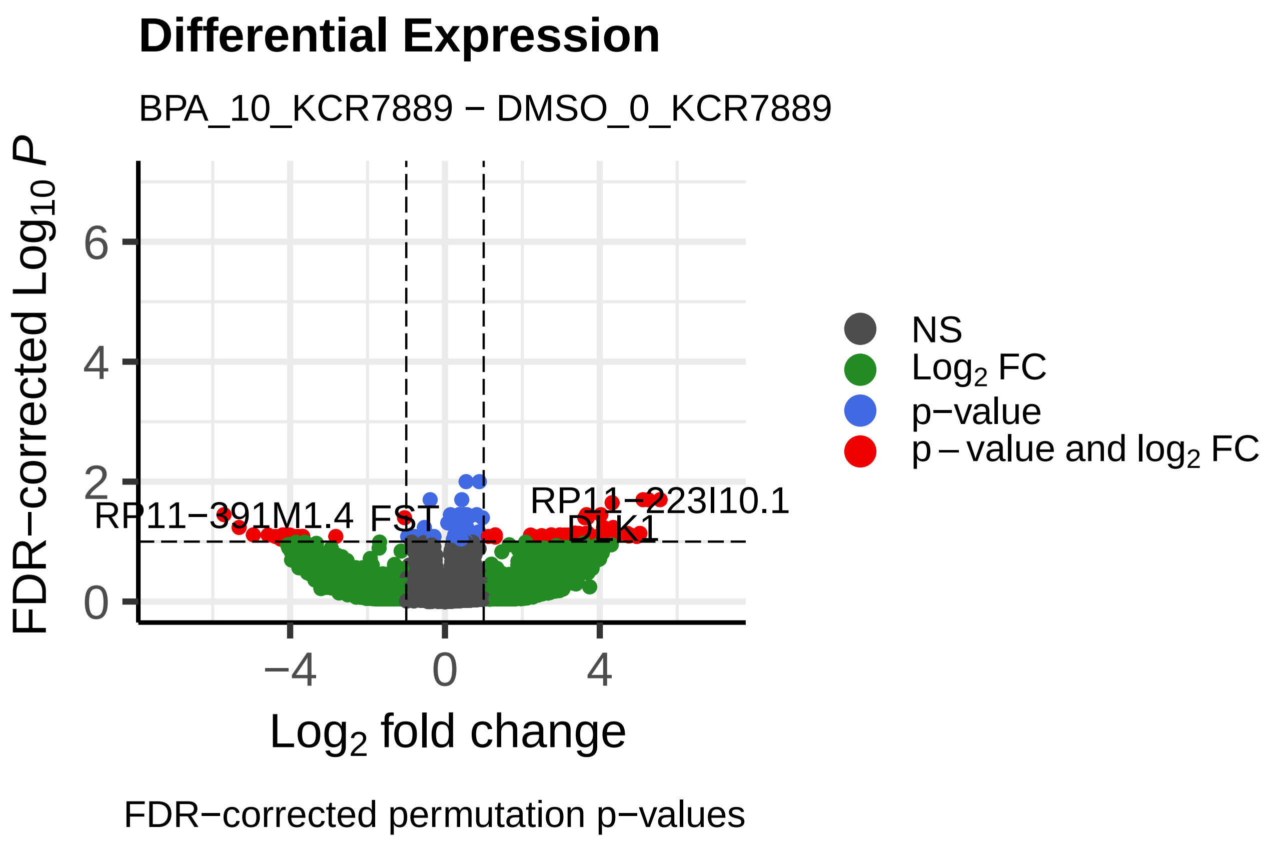
Differential Expression
BPA_10_KCR7889 − DMSO_0_KCR7889
FDR−corrected Log10 P
Log2 fold change
NS
Log2 FC
p−value
p − value and log2 FC
RP11−391M1.4
FST
RP11−223I10.1
DLK1
FDR−corrected permutation p−values

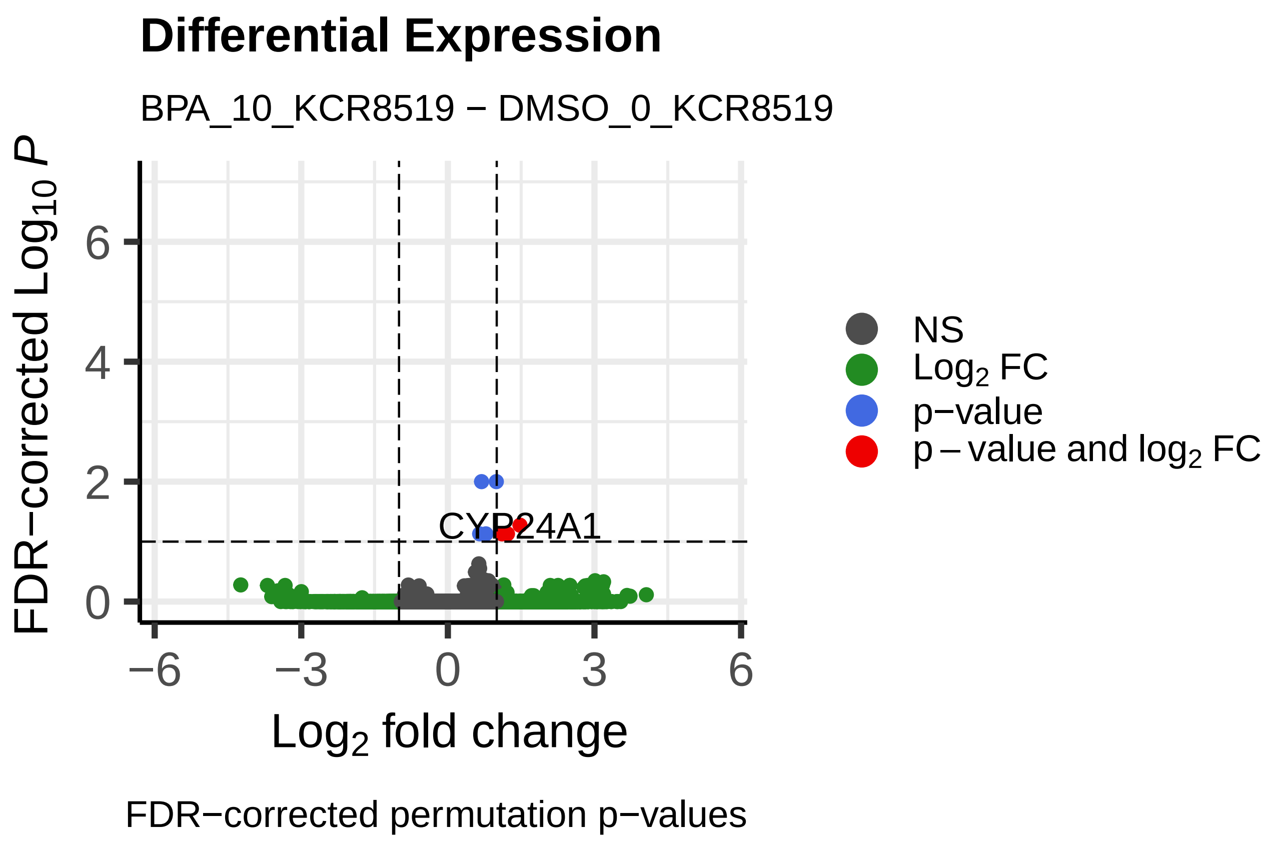
Differential Expression
BPA_10_KCR8519 − DMSO_0_KCR8519
FDR−corrected Log10 P
Log2 fold change
NS
Log2 FC
p−value
p − value and log2 FC
CYP24A1
FDR−corrected permutation p−values

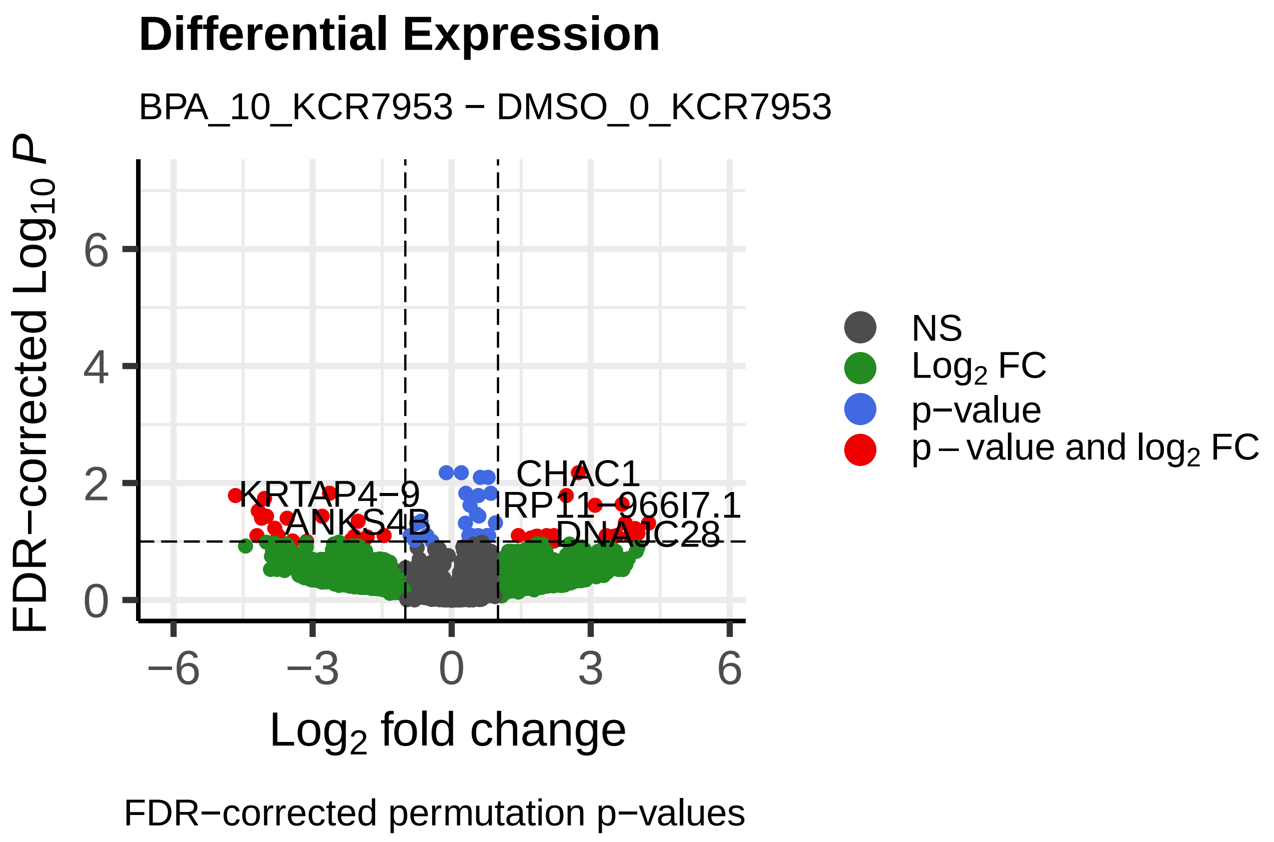
Differential Expression
BPA_10_KCR7953 − DMSO_0_KCR7953
FDR−corrected Log10 P
Log2 fold change
NS
Log2 FC
p−value
p − value and log2 FC
CHAC1
KRTAP4−9
RP11−966I7.1
ANKS4B
DNAJC28
FDR−corrected permutation p−values

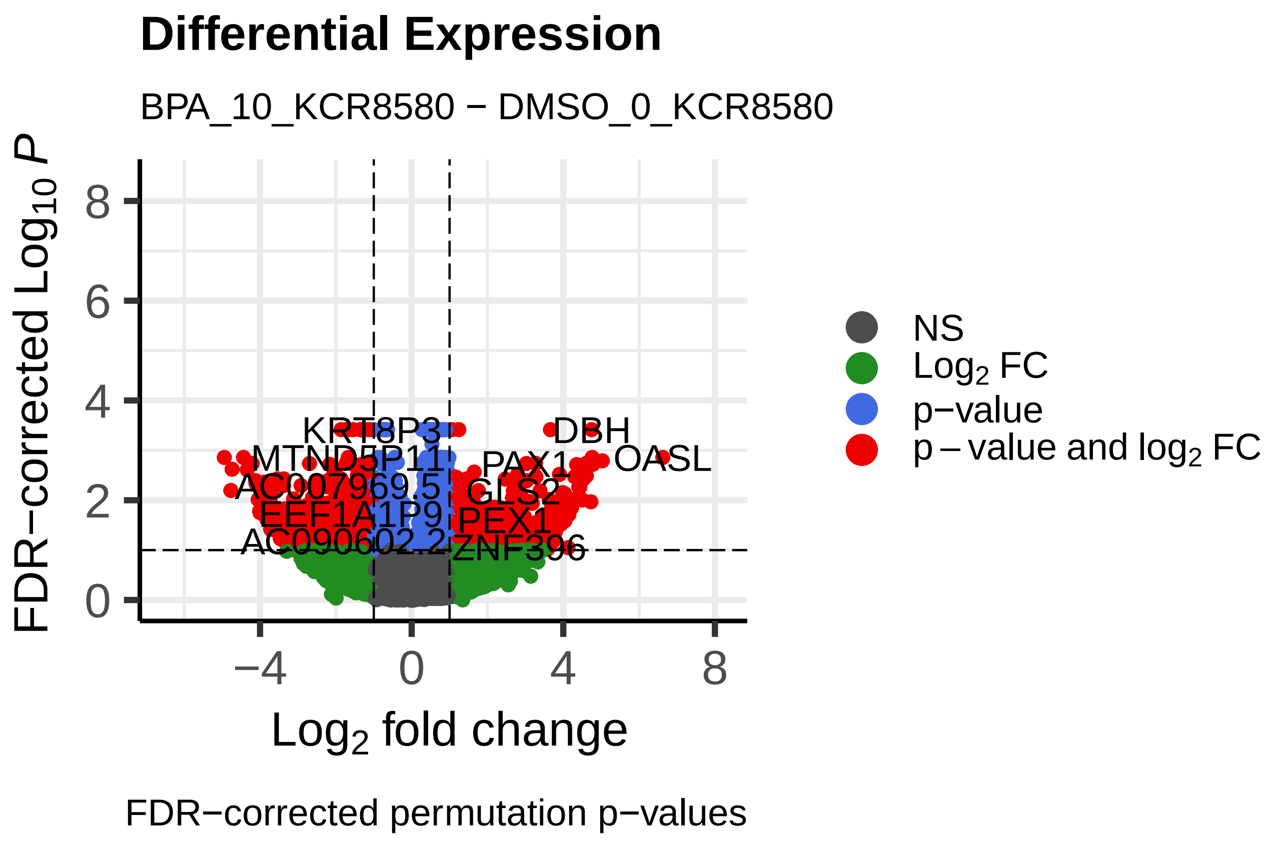
Differential Expression
BPA_10_KCR8580 − DMSO_0_KCR8580
FDR−corrected Log10 P
Log2 fold change
NS
Log2 FC
p−value
p − value and log2 FC
DBH
OASL
PAX1
PEX1
ZNF396
FDR−corrected permutation p−values

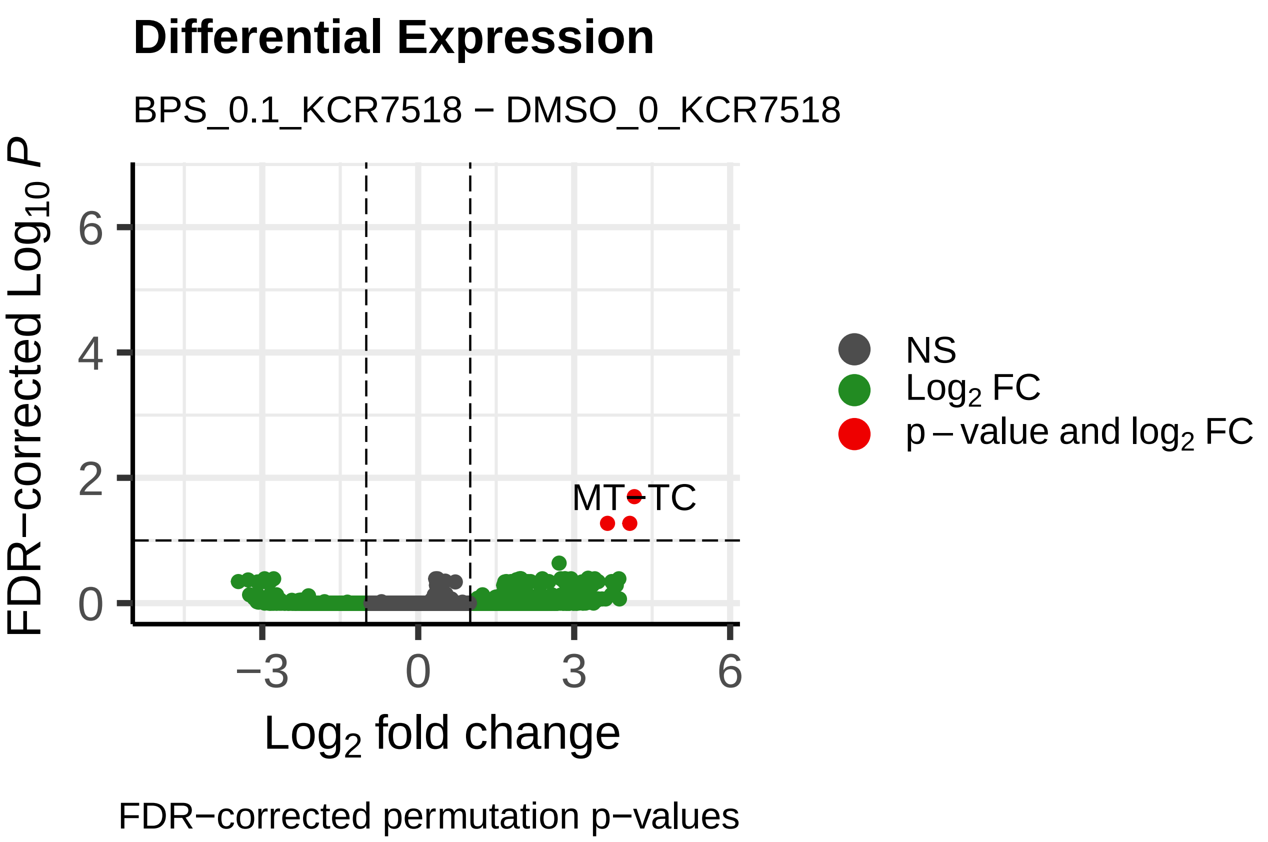
Differential Expression
BPS_0.1_KCR7518 − DMSO_0_KCR7518
FDR−corrected Log10 P
Log2 fold change
MT−TC
NS
Log2 FC
p − value and log2 FC
FDR−corrected permutation p−values

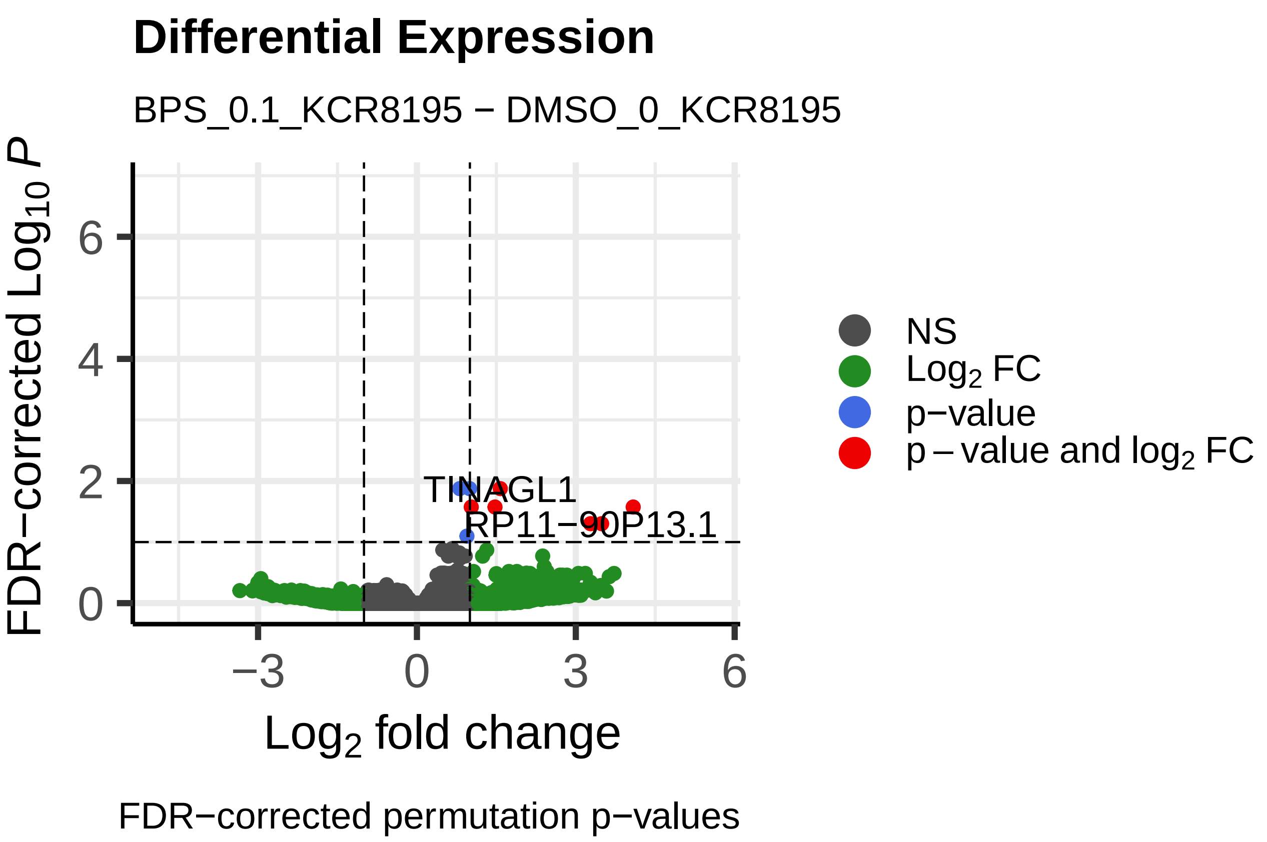
Differential Expression
BPS_0.1_KCR8195 − DMSO_0_KCR8195
FDR−corrected Log10 P
Log2 fold change
TINAGL1
RP11−90P13.1
NS
Log2 FC
p−value
p − value and log2 FC
FDR−corrected permutation p−values

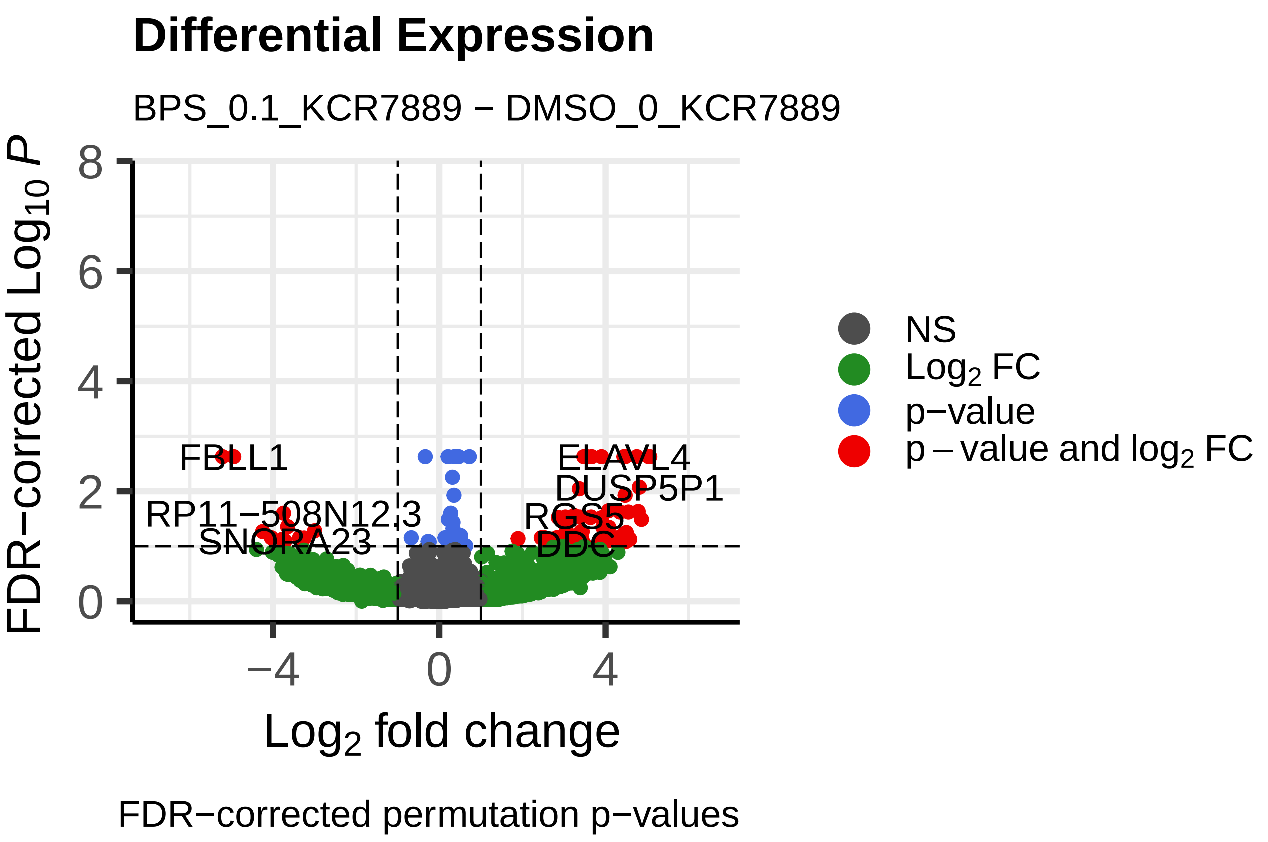
Differential Expression
BPS_0.1_KCR7889 − DMSO_0_KCR7889
FDR−corrected Log10 P
Log2 fold change
FBLL1
RP11−508N12.3
SNORA23
ELAVL4
DUSP5P1
RGS5
DDC
NS
Log2 FC
p−value
p − value and log2 FC
FDR−corrected permutation p−values

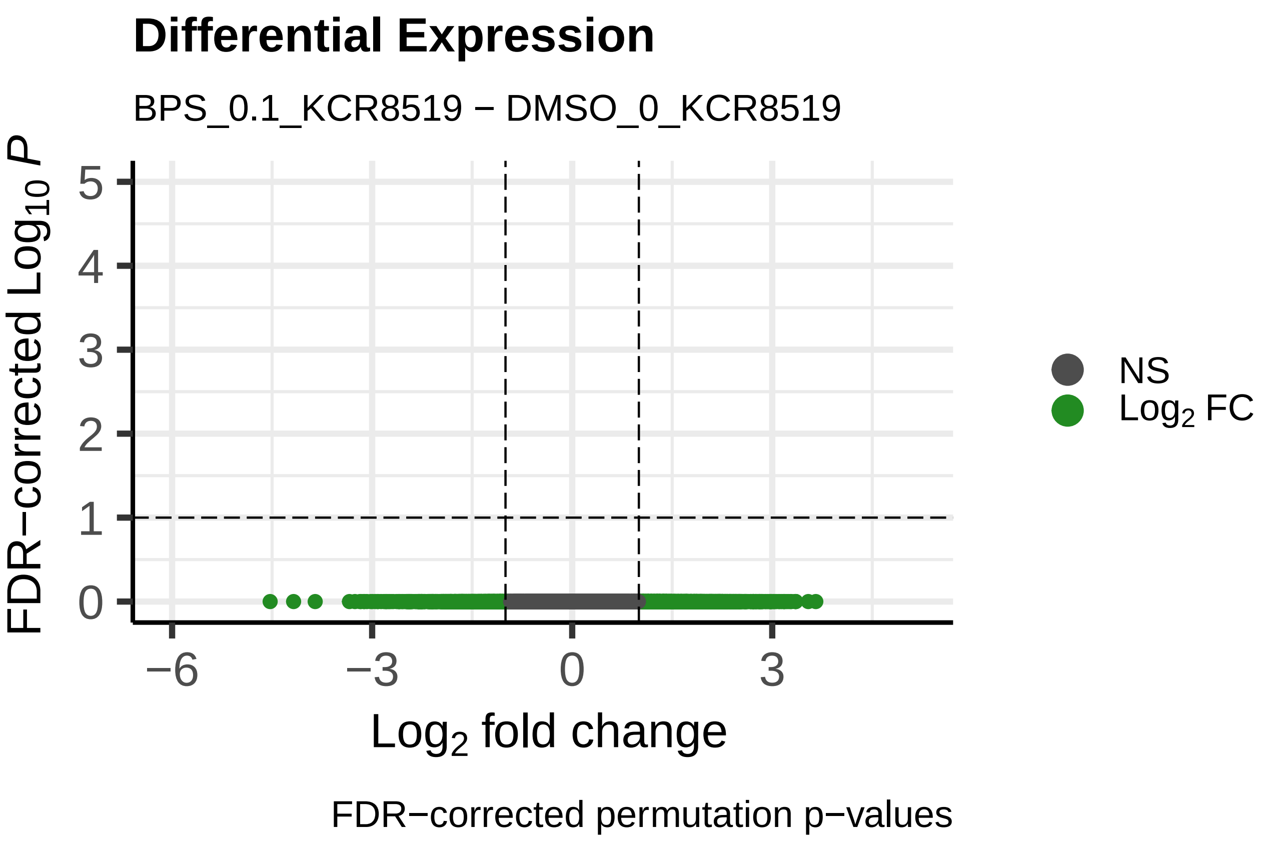
Differential Expression
BPS_0.1_KCR8519 − DMSO_0_KCR8519
FDR−corrected Log10 P
Log2 fold change
NS
Log2 FC
FDR−corrected permutation p−values

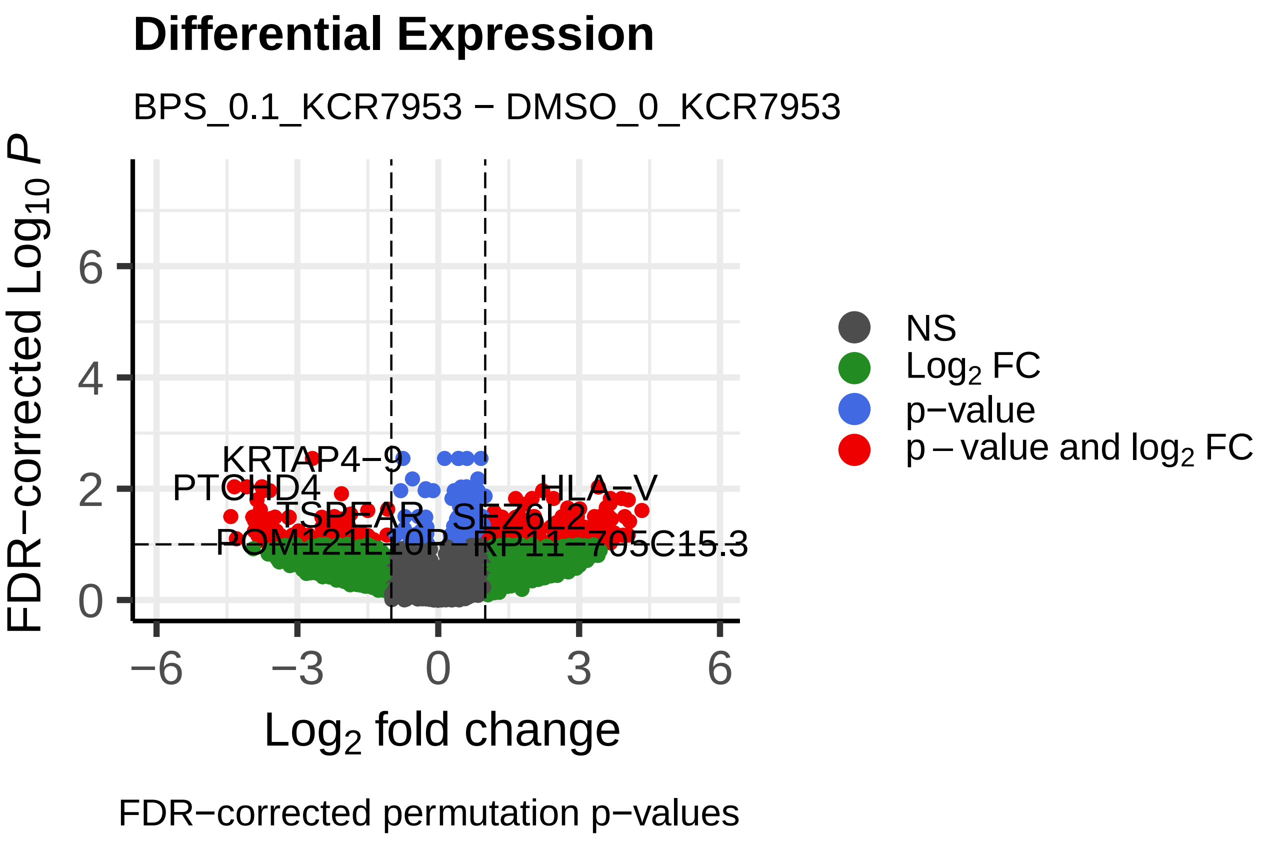
Differential Expression
BPS_0.1_KCR7953 − DMSO_0_KCR7953
FDR−corrected Log10 P
Log2 fold change
KRTAP4−9
PTCHD4
TSPEAR
SEZ6L2
HLA−V
POM121L10P
RP11−705C15.3
NS
Log2 FC
p−value
p − value and log2 FC
FDR−corrected permutation p−values

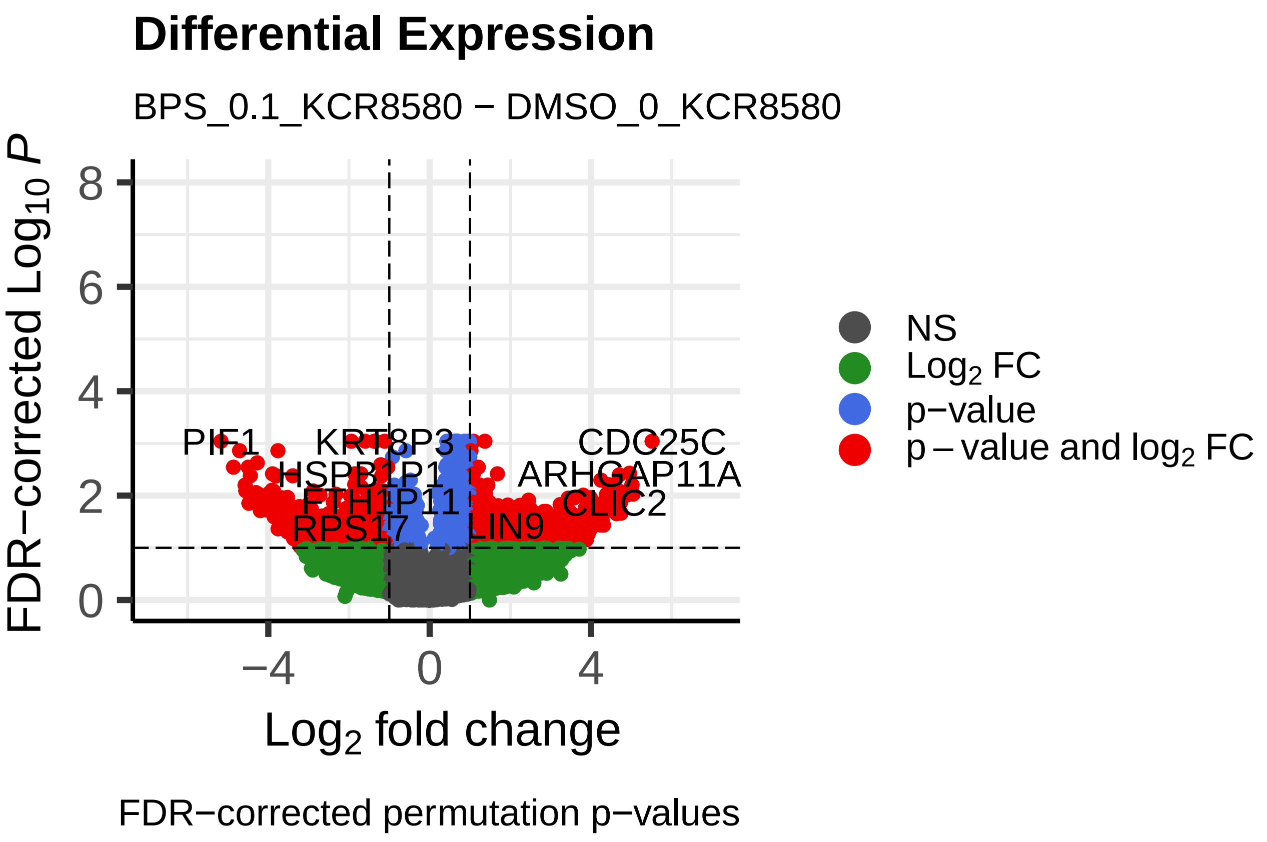
Differential Expression
BPS_0.1_KCR8580 − DMSO_0_KCR8580
FDR−corrected Log10 P
Log2 fold change
PIF1
KRT8P3
CDC25C
ARHGAP11A
CLIC2
FTH1P11
RPS17
LIN9
NS
Log2 FC
p−value
p − value and log2 FC
FDR−corrected permutation p−values

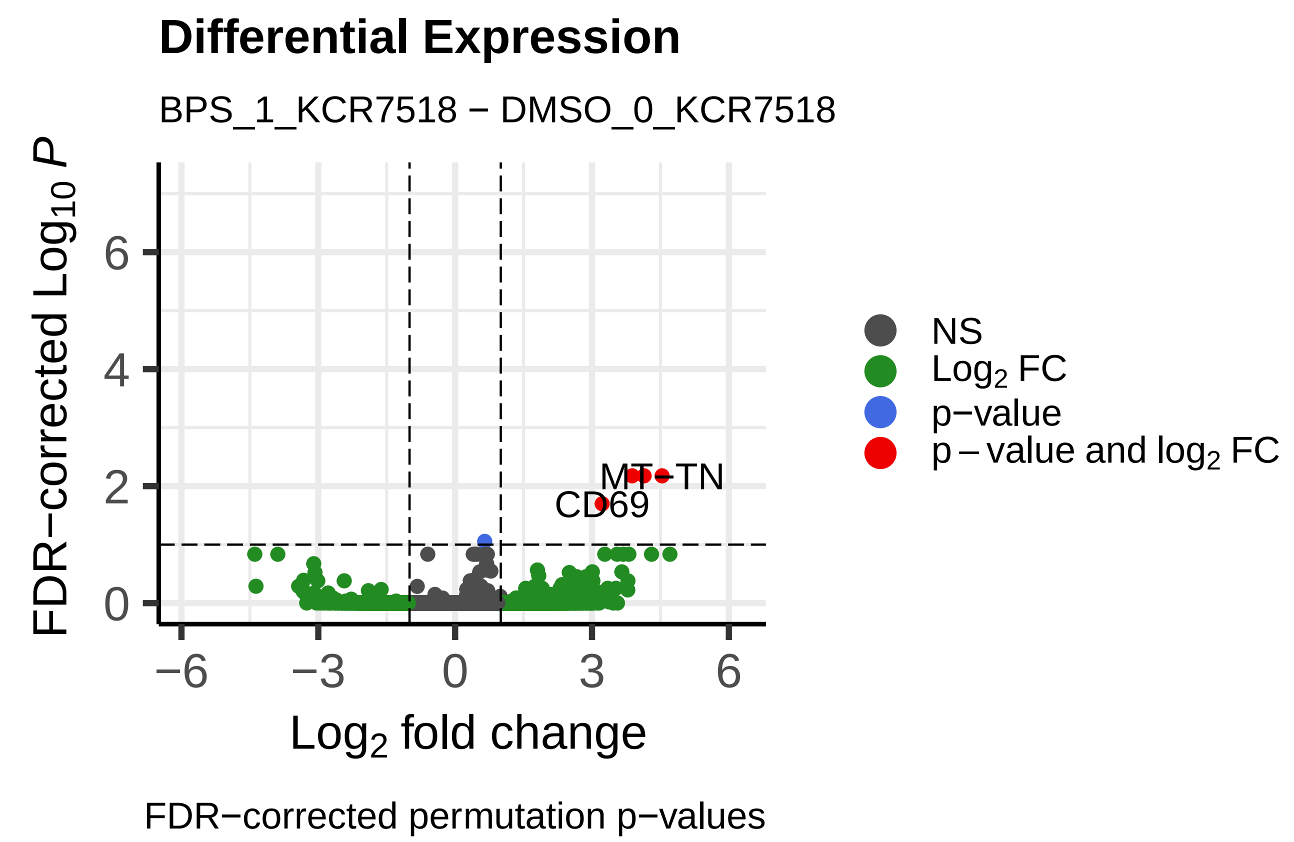
Differential Expression
BPS_1_KCR7518 − DMSO_0_KCR7518
FDR−corrected $Log_{10}$ $P$
Log$_2$ fold change
MT−TN
CD69
NS
Log$_2$ FC
p−value
p − value and log$_2$ FC
FDR−corrected permutation p−values

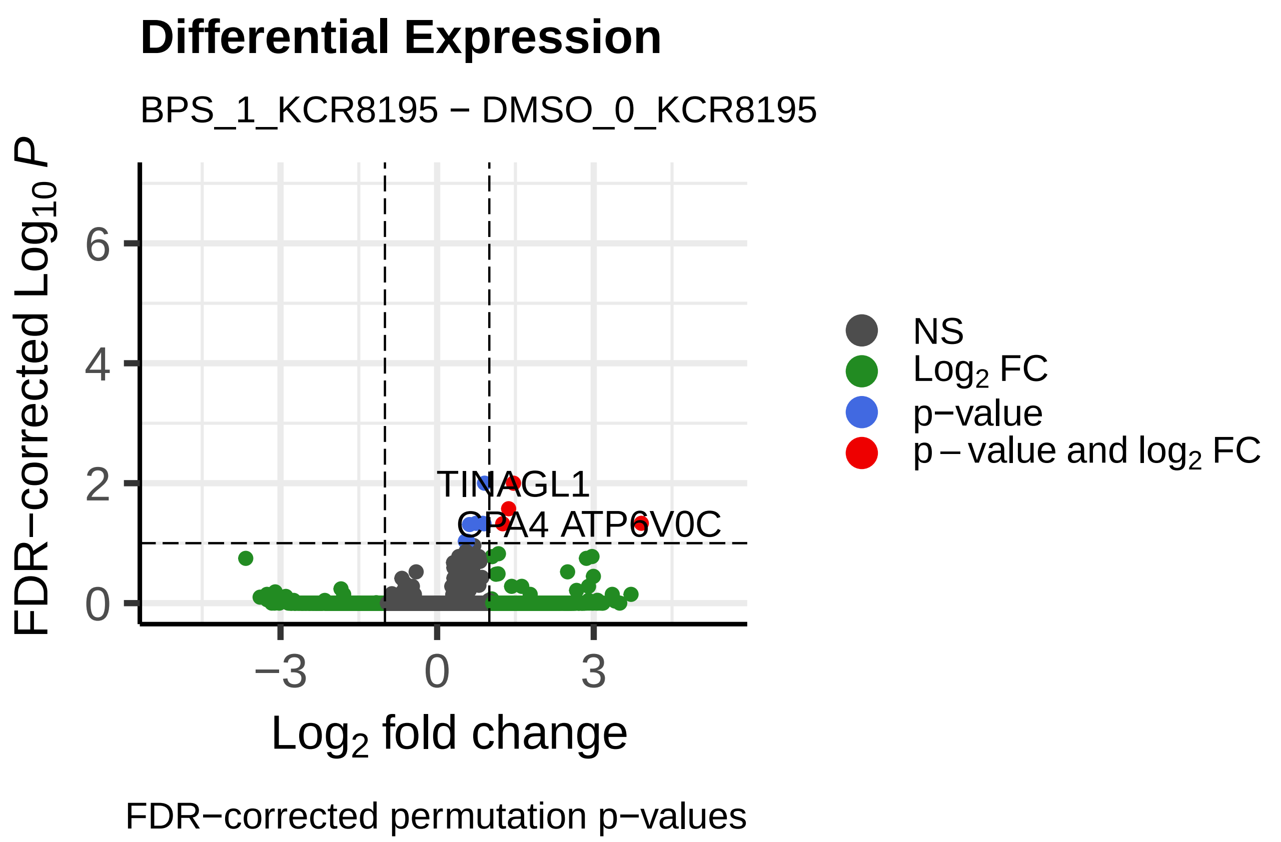
Differential Expression
BPS_1_KCR8195 − DMSO_0_KCR8195
FDR−corrected $Log_{10}$ $P$
Log$_2$ fold change
TINAGL1
CPA4 ATP6V0C
NS
Log$_2$ FC
p−value
p − value and log$_2$ FC
FDR−corrected permutation p−values

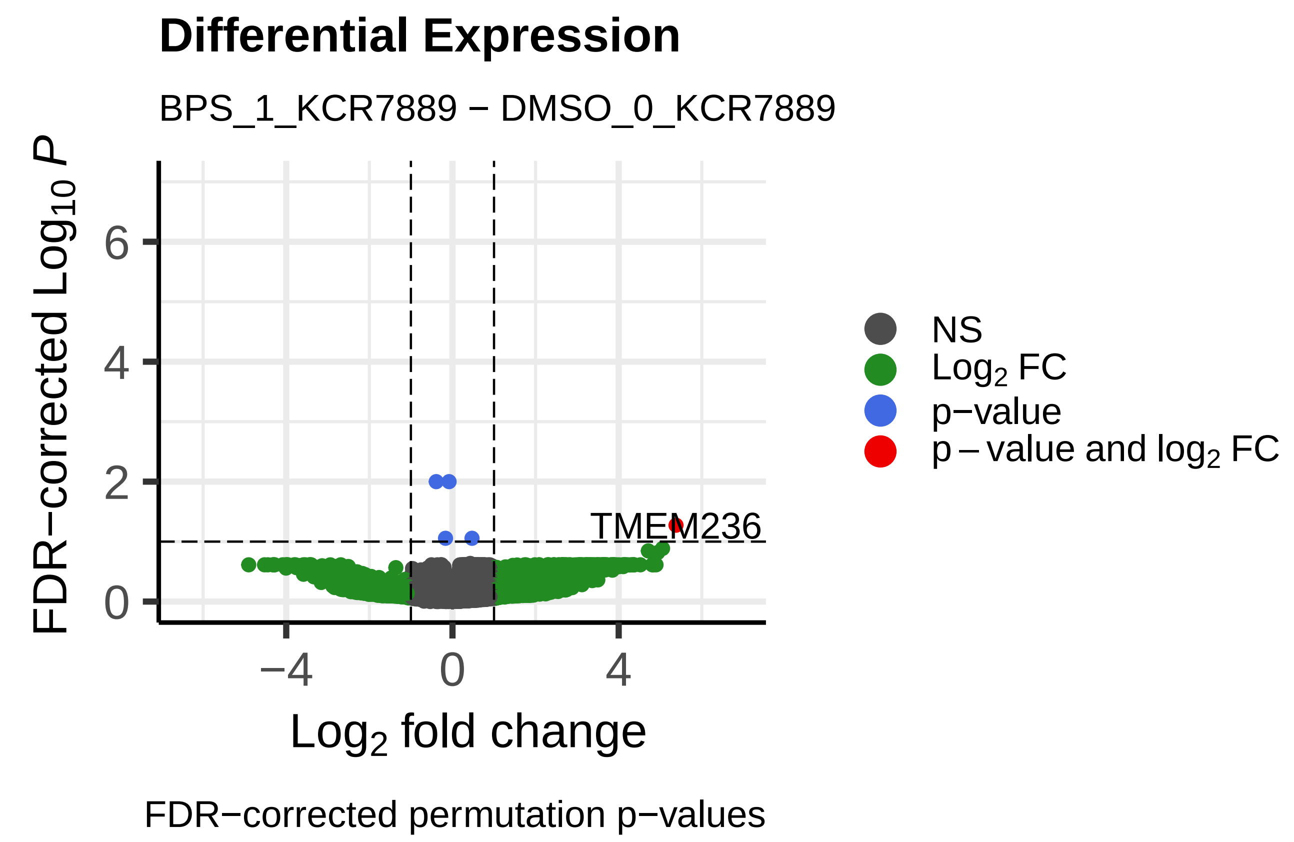
Differential Expression
BPS_1_KCR7889 − DMSO_0_KCR7889
FDR−corrected $Log_{10}$ $P$
Log$_2$ fold change
TMEM236
NS
Log$_2$ FC
p−value
p − value and log$_2$ FC
FDR−corrected permutation p−values

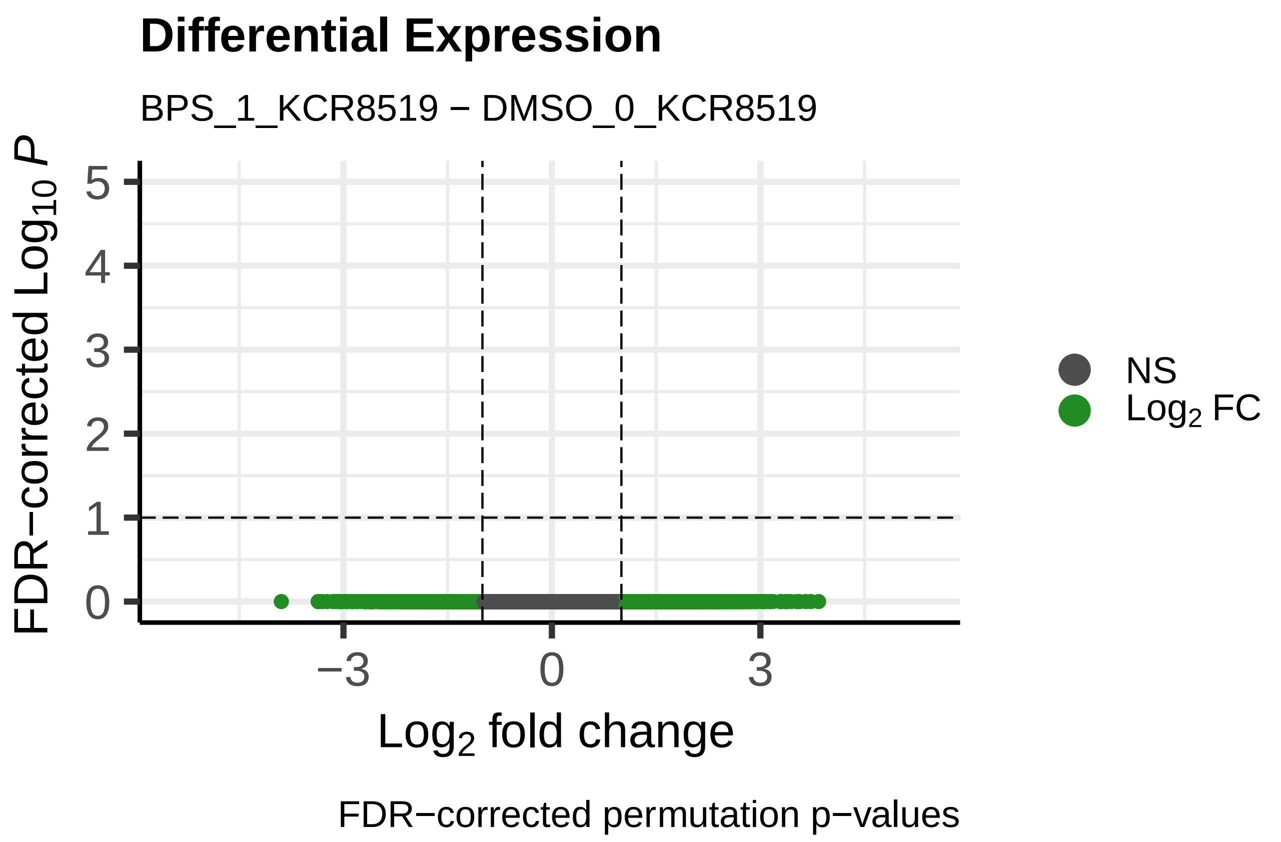
Differential Expression
BPS_1_KCR8519 − DMSO_0_KCR8519
FDR−corrected $Log_{10}$ $P$
Log$_2$ fold change
NS
Log$_2$ FC
FDR−corrected permutation p−values

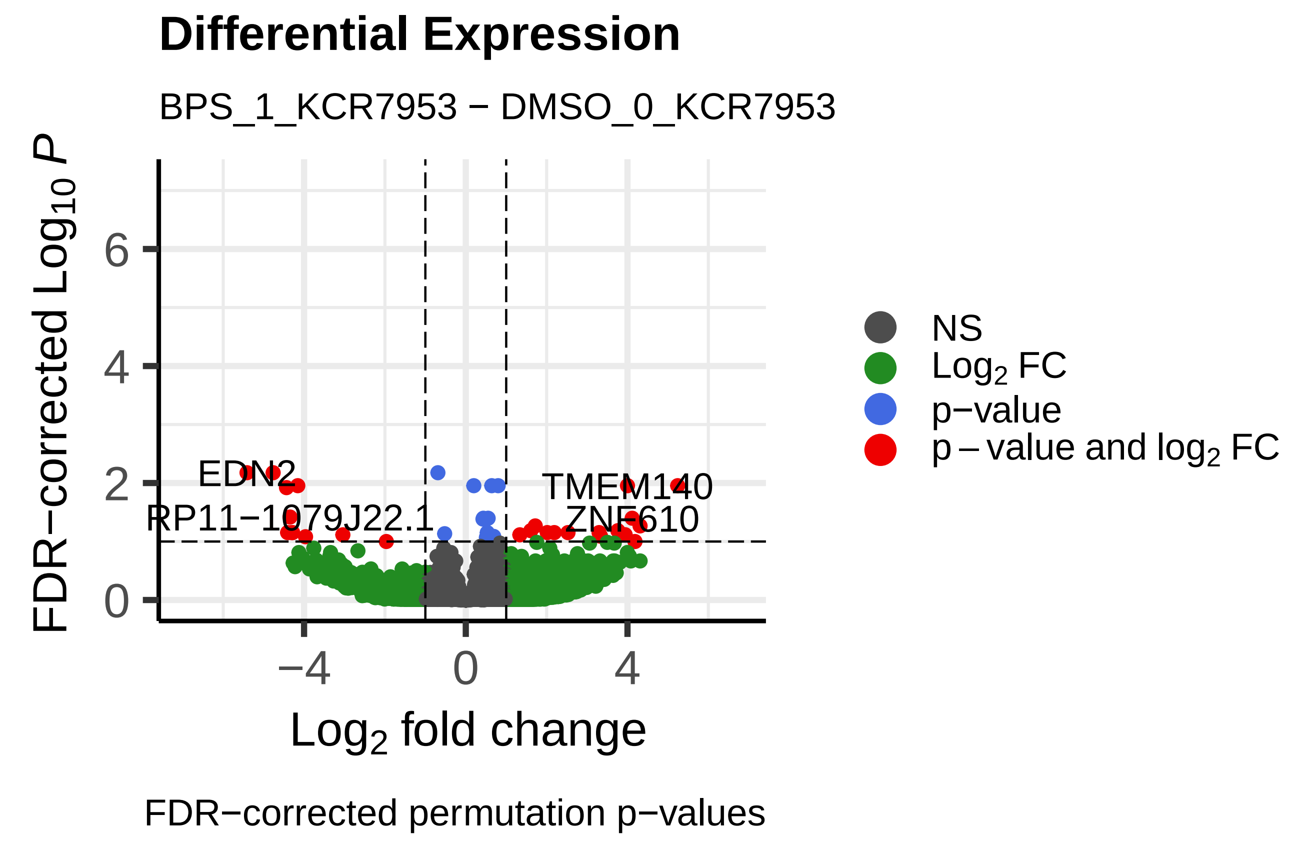
Differential Expression
BPS_1_KCR7953 − DMSO_0_KCR7953
FDR−corrected $Log_{10}$ $P$
Log$_2$ fold change
EDN2
RP11−1079J22.1
TMEM140
ZNF610
NS
Log$_2$ FC
p−value
p − value and log$_2$ FC
FDR−corrected permutation p−values

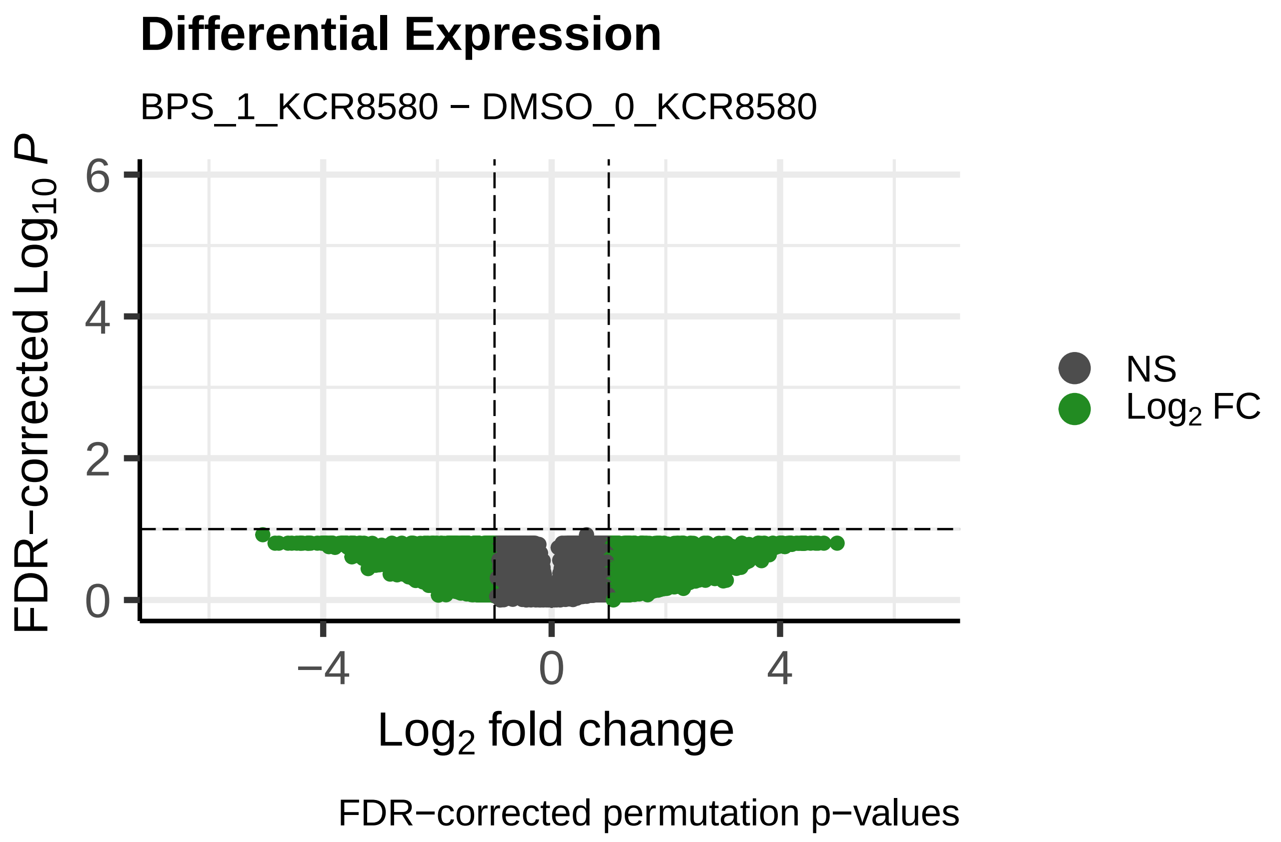
Differential Expression
BPS_1_KCR8580 − DMSO_0_KCR8580
FDR−corrected $Log_{10}$ $P$
Log$_2$ fold change
NS
Log$_2$ FC
FDR−corrected permutation p−values

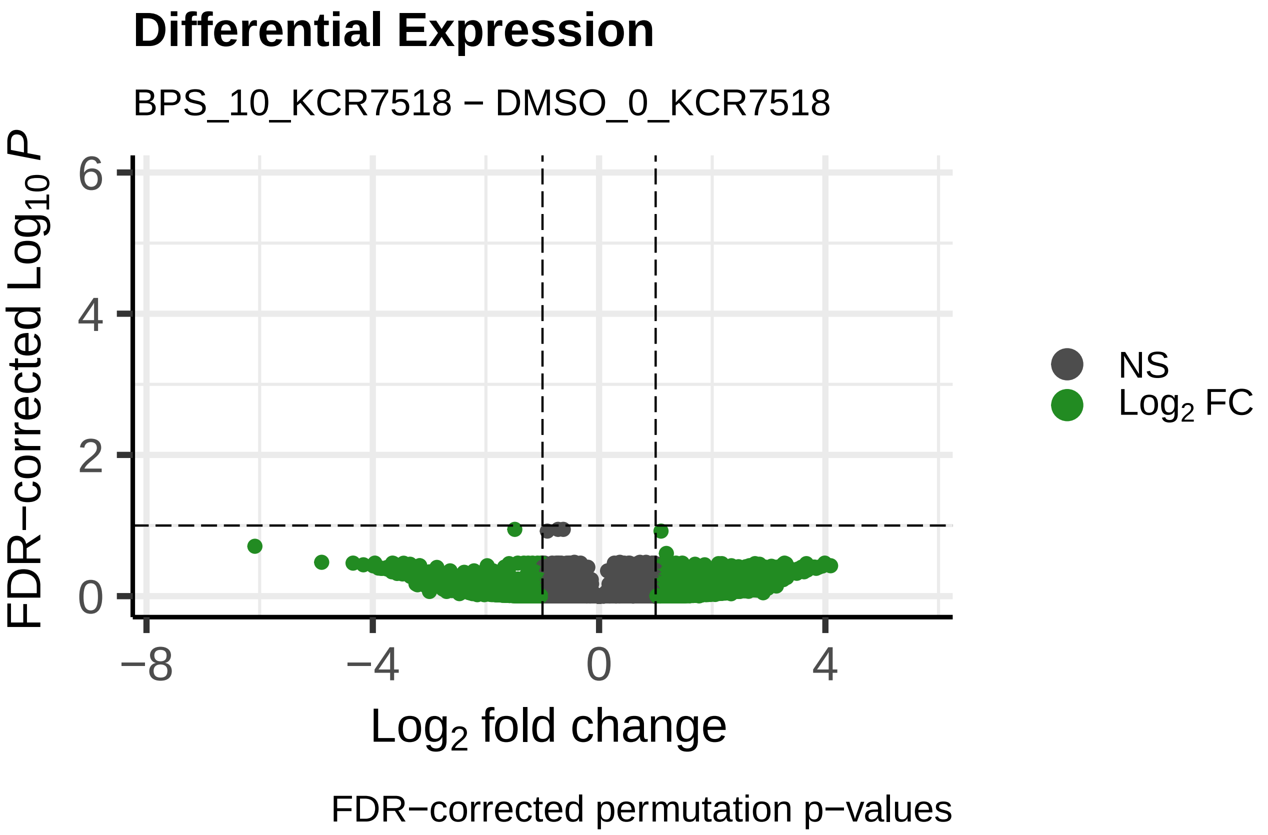
Differential Expression
BPS_10_KCR7518 − DMSO_0_KCR7518
FDR−corrected Log10 P
Log2 fold change
NS
Log2 FC
FDR−corrected permutation p−values

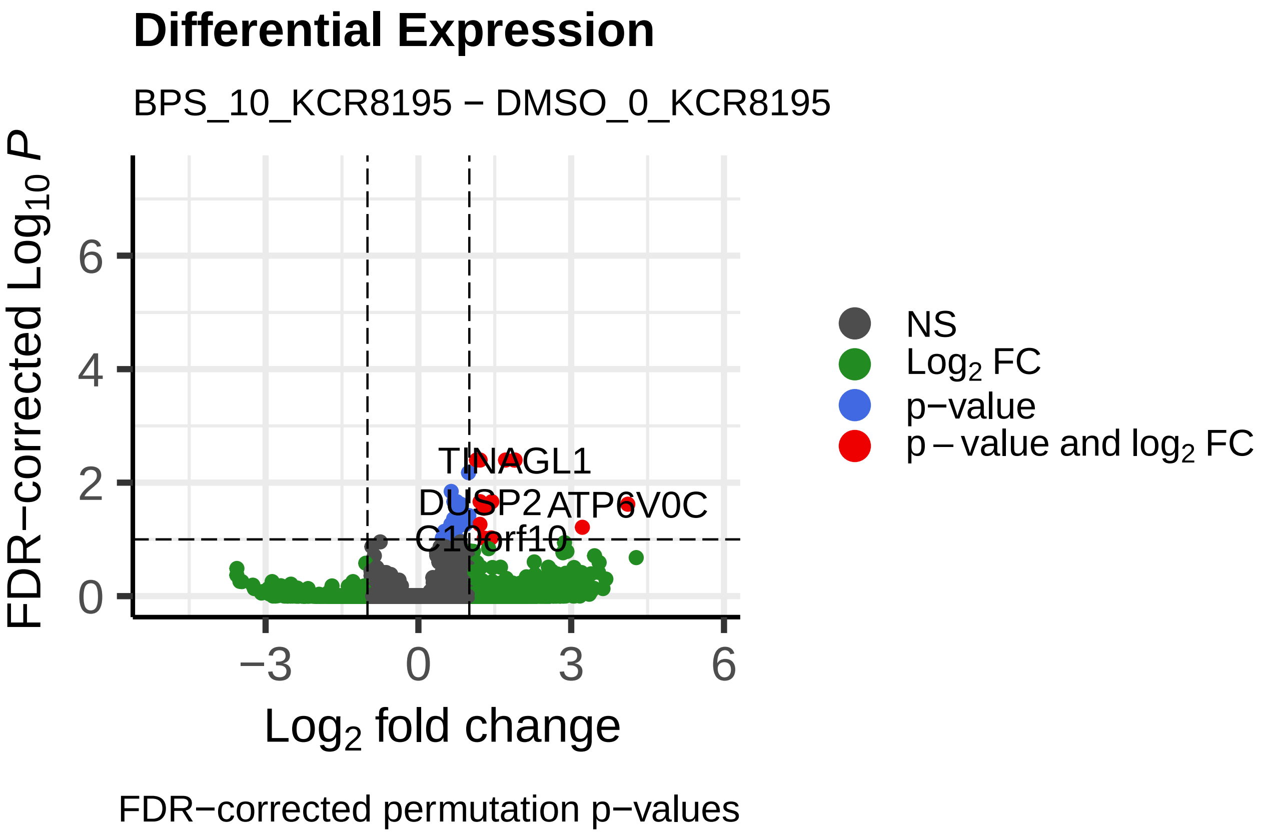
Differential Expression
BPS_10_KCR8195 − DMSO_0_KCR8195
TINAGL1
DUSP2
ATP6V0C
C10orf10
FDR−corrected Log10 P
Log2 fold change
NS
Log2 FC
p−value
p − value and log2 FC
FDR−corrected permutation p−values

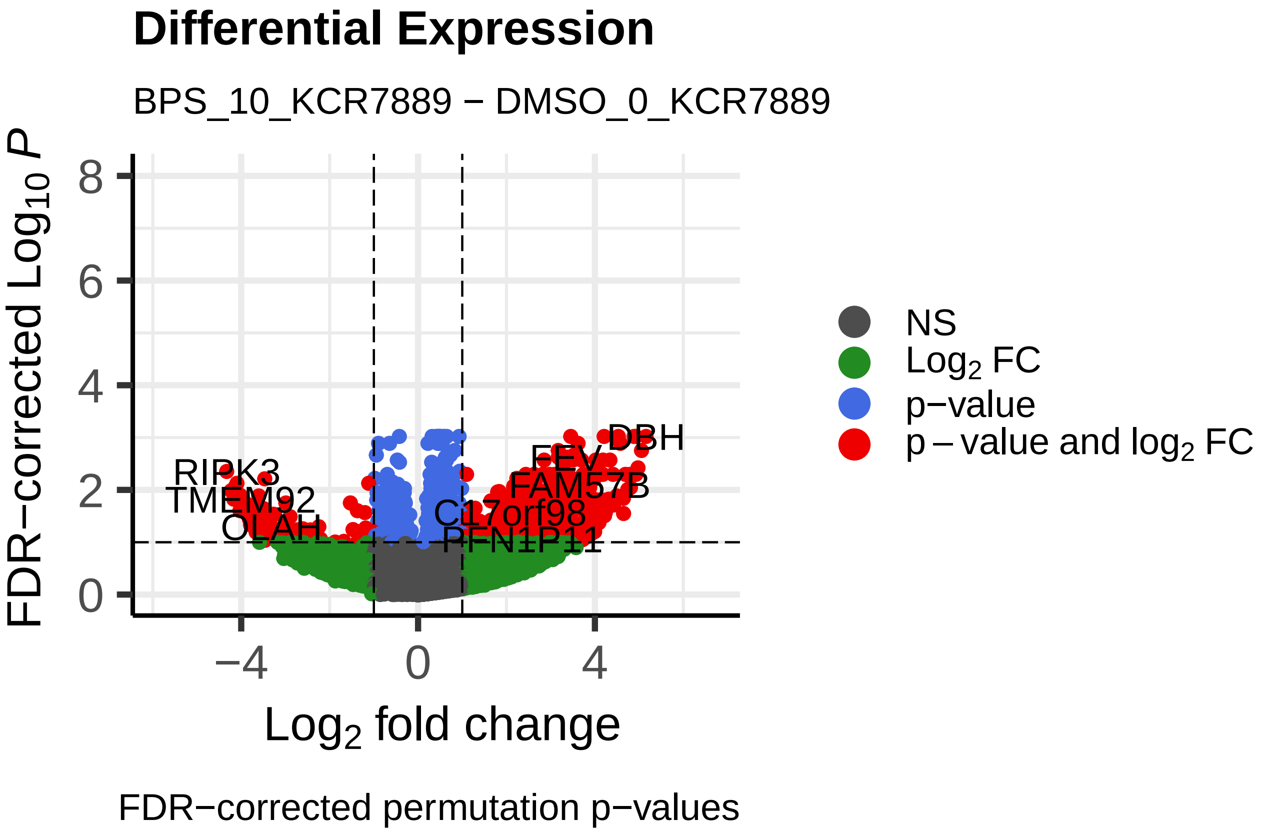
Differential Expression
BPS_10_KCR7889 − DMSO_0_KCR7889
RIPK3
TMEM92
OLAH
DBH
FEV
FAM57B
C17orf98
FDR−corrected Log10 P
Log2 fold change
NS
Log2 FC
p−value
p − value and log2 FC
FDR−corrected permutation p−values

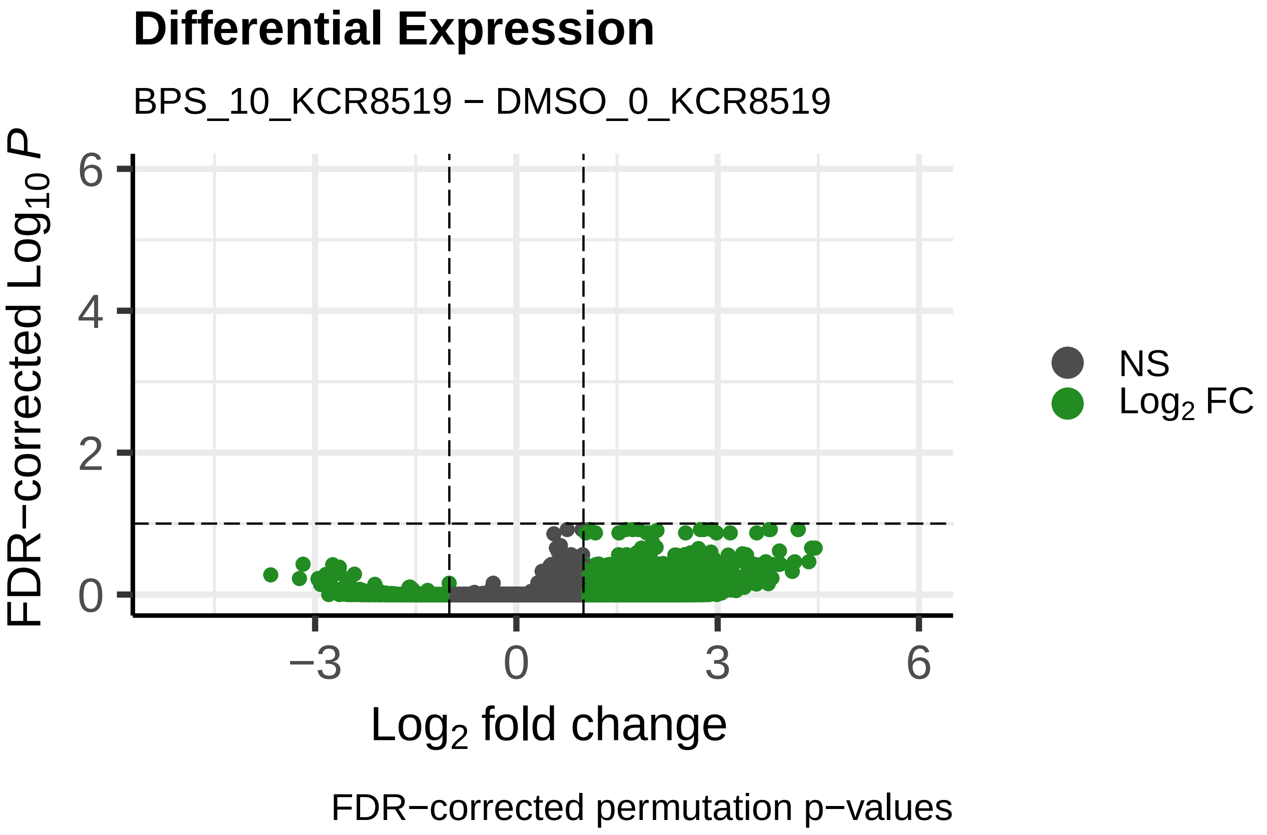
Differential Expression
BPS_10_KCR8519 − DMSO_0_KCR8519
FDR−corrected Log10 P
Log2 fold change
NS
Log2 FC
FDR−corrected permutation p−values

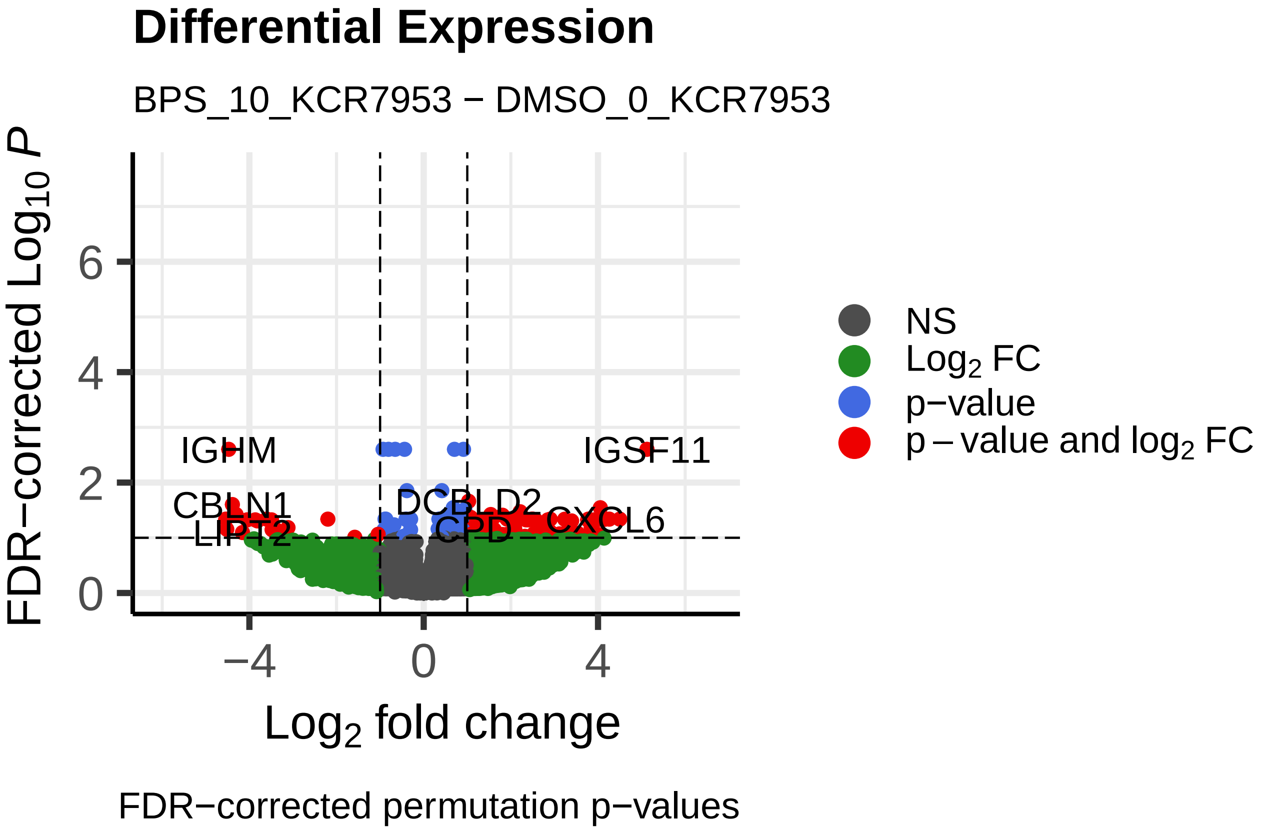
Differential Expression
BPS_10_KCR7953 − DMSO_0_KCR7953
IGHM
IGSF11
CBLN1
LIPT2
DCBLD2
CPD
CXCL6
FDR−corrected Log10 P
Log2 fold change
NS
Log2 FC
p−value
p − value and log2 FC
FDR−corrected permutation p−values

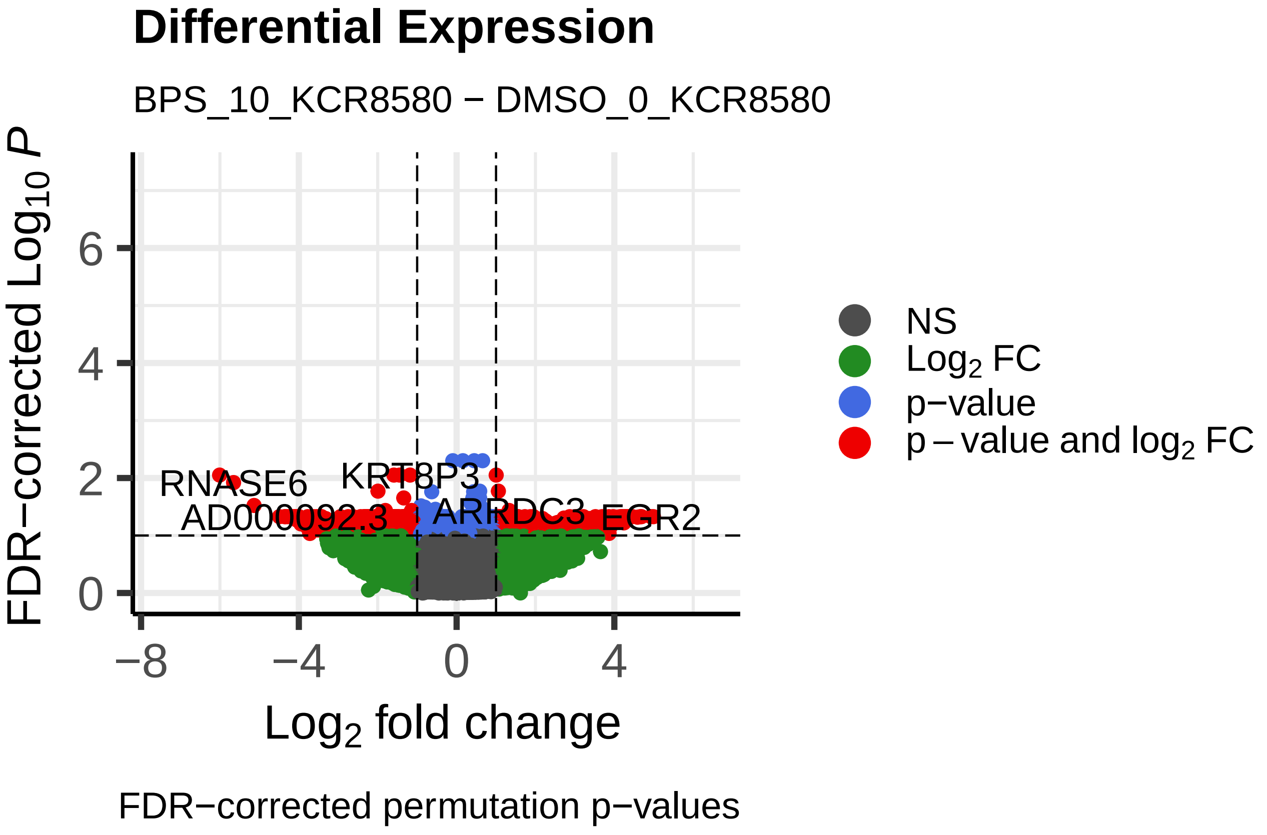
Differential Expression
BPS_10_KCR8580 − DMSO_0_KCR8580
RNASE6
KRT8P3
AD000092.3
ARRDC3
EGR2
FDR−corrected Log10 P
Log2 fold change
NS
Log2 FC
p−value
p − value and log2 FC
FDR−corrected permutation p−values

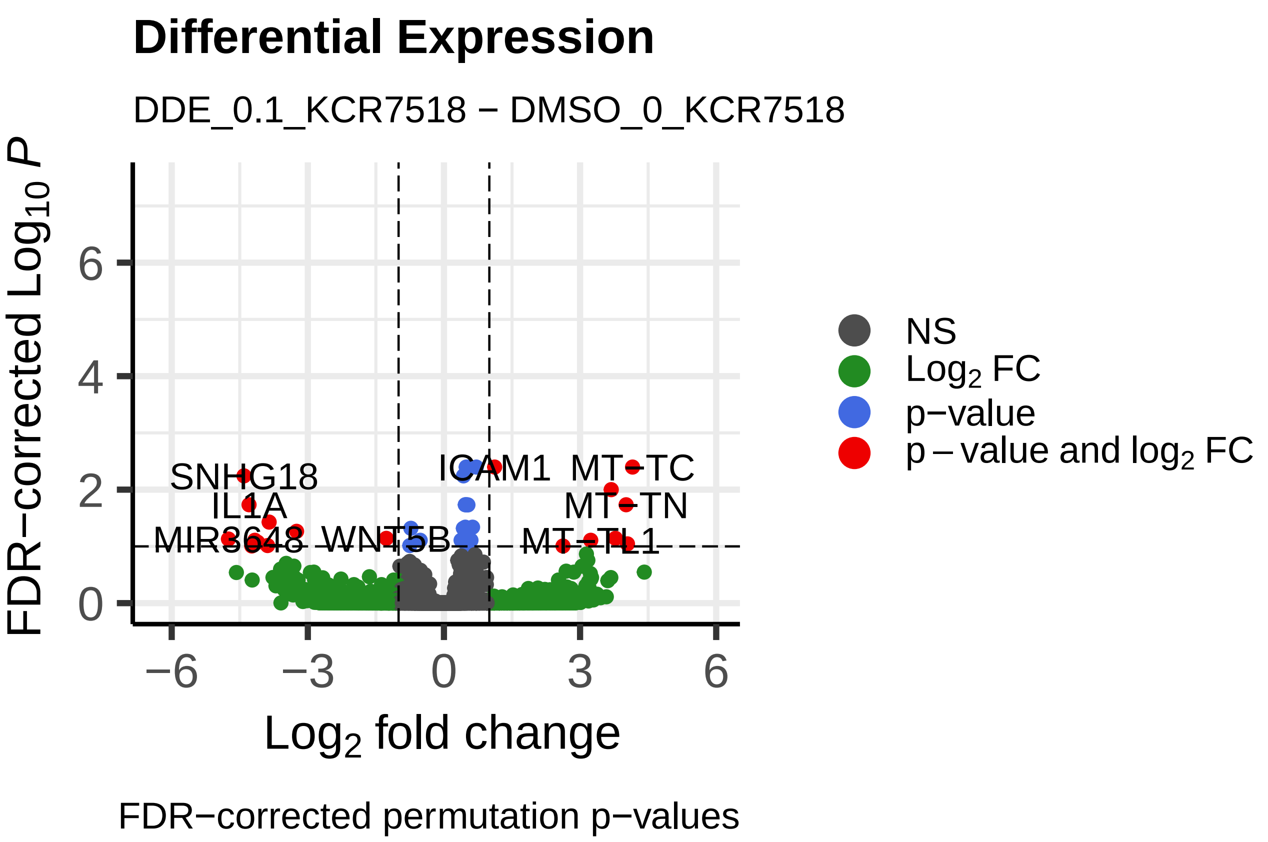
Differential Expression
DDE_0.1_KCR7518 − DMSO_0_KCR7518
FDR−corrected $Log_{10}$ P
Log2 fold change
NS
Log2 FC
p−value
p − value and log2 FC
SNHG18
IL1A
MIR3648
WNT5B
ICAM1
MT−TC
MT−TN
MT−TL1
FDR−corrected permutation p−values

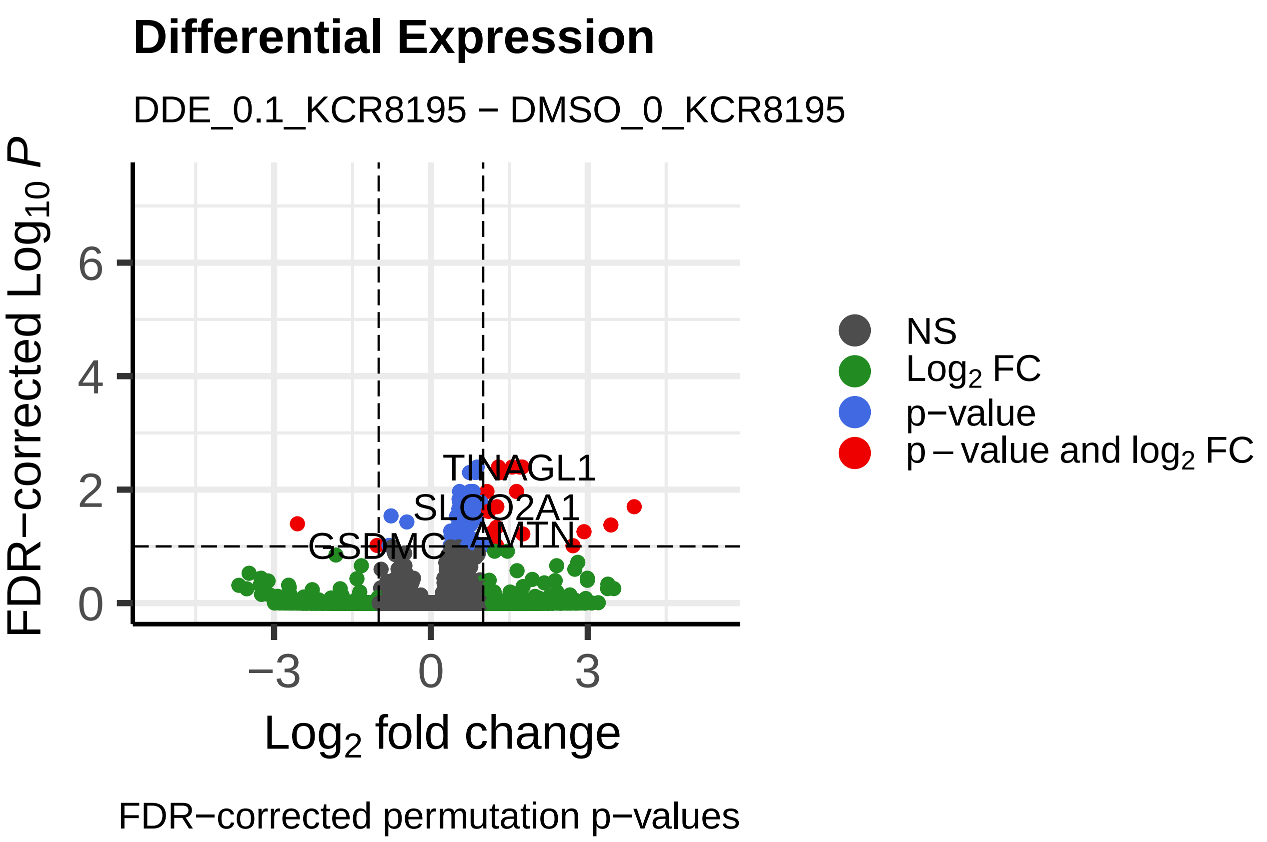
Differential Expression
DDE_0.1_KCR8195 − DMSO_0_KCR8195
FDR−corrected $Log_{10}$ P
Log2 fold change
NS
Log2 FC
p−value
p − value and log2 FC
TINAGL1
SLCO2A1
GSDMC
AMTN
FDR−corrected permutation p−values

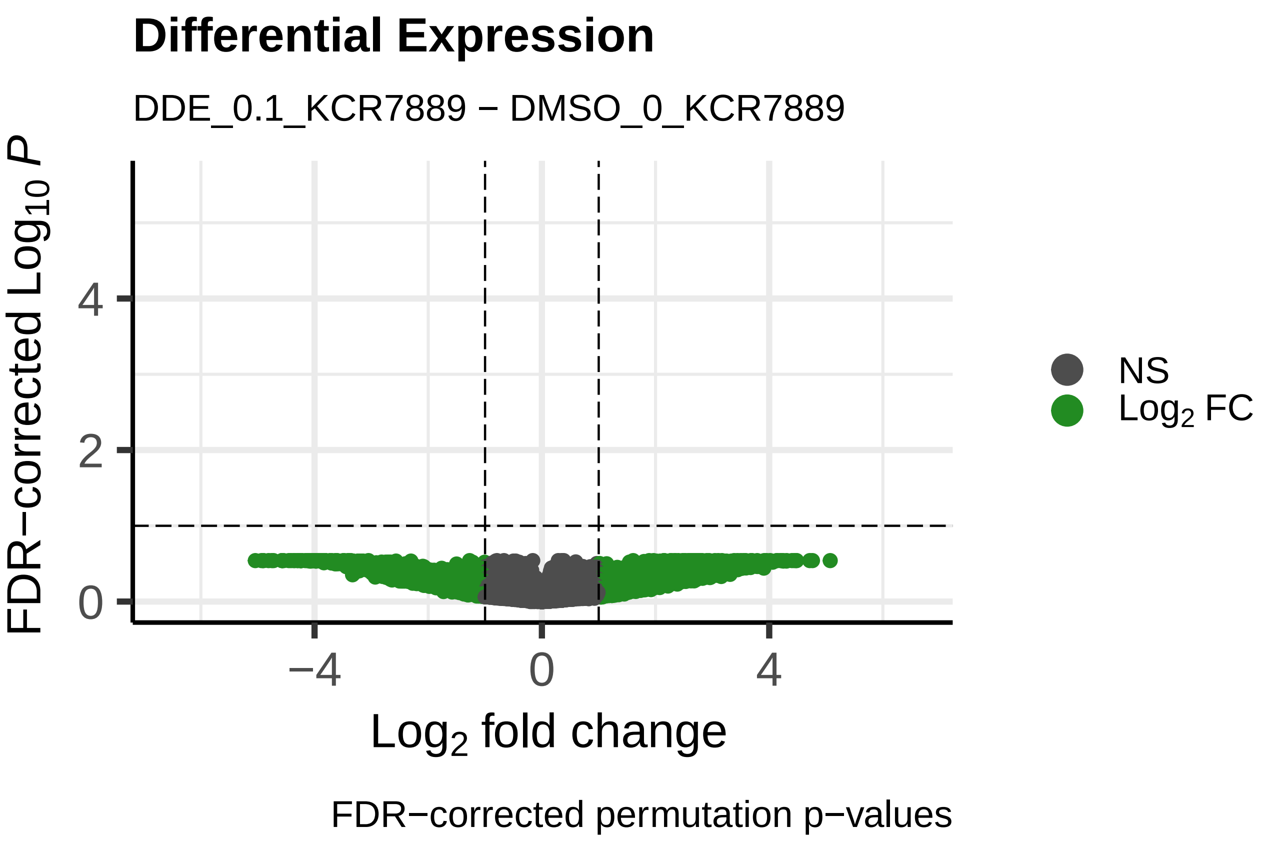
Differential Expression
DDE_0.1_KCR7889 − DMSO_0_KCR7889
FDR−corrected $Log_{10}$ P
Log2 fold change
NS
Log2 FC
FDR−corrected permutation p−values

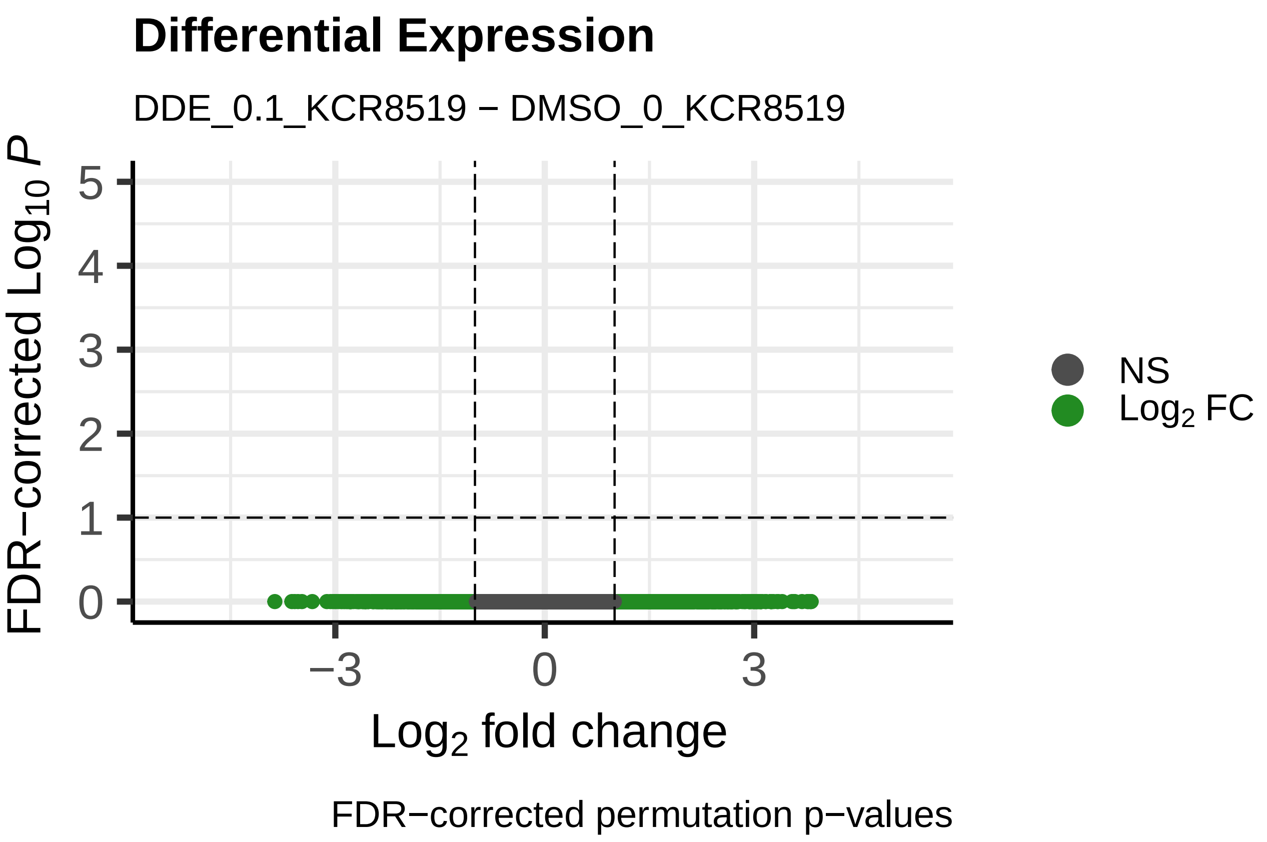
Differential Expression
DDE_0.1_KCR8519 − DMSO_0_KCR8519
FDR−corrected $Log_{10}$ P
Log2 fold change
NS
Log2 FC
FDR−corrected permutation p−values

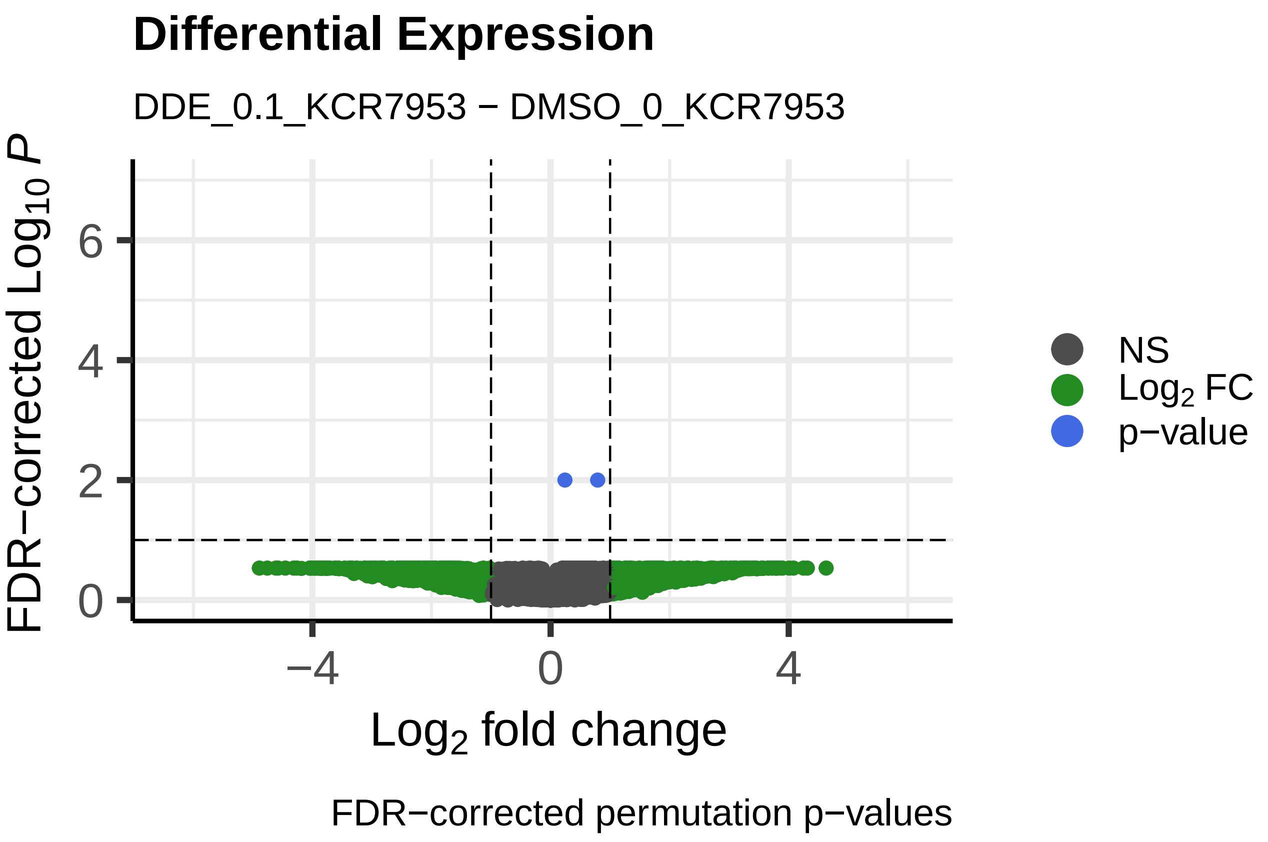
Differential Expression
DDE_0.1_KCR7953 − DMSO_0_KCR7953
FDR−corrected $Log_{10}$ P
Log2 fold change
NS
Log2 FC
p−value
FDR−corrected permutation p−values

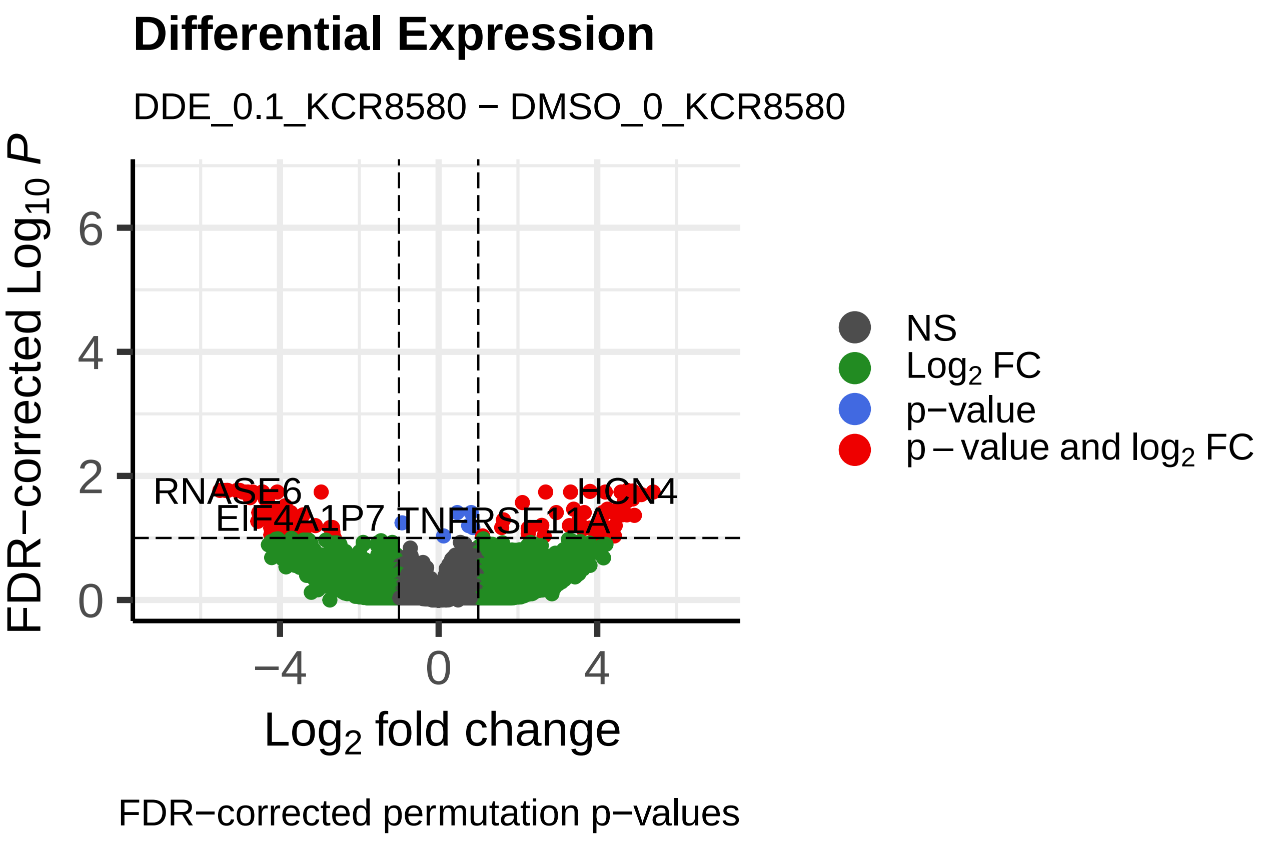
Differential Expression
DDE_0.1_KCR8580 − DMSO_0_KCR8580
FDR−corrected $Log_{10}$ P
Log2 fold change
NS
Log2 FC
p−value
p − value and log2 FC
RNASE6
HCN4
EIF4A1P7
TNFRSF11A
FDR−corrected permutation p−values

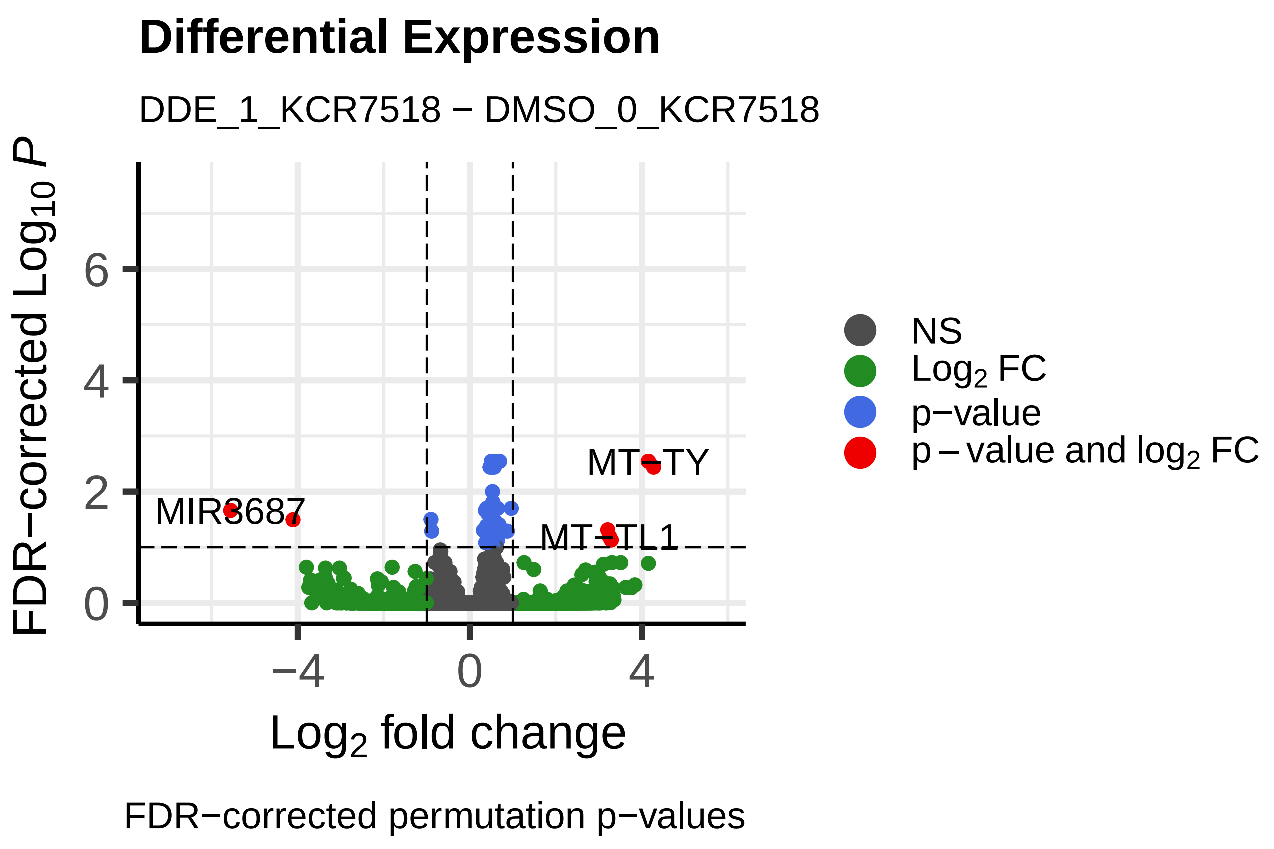
Differential Expression
DDE_1_KCR7518 − DMSO_0_KCR7518
FDR−corrected $Log_{10}$ $P$
0
2
4
6
−4
0
4
$Log_2$ fold change
MIR3687
MT−TY
MT−TL1
NS
$Log_2$ FC
p−value
p − value and $log_2$ FC
FDR−corrected permutation p−values

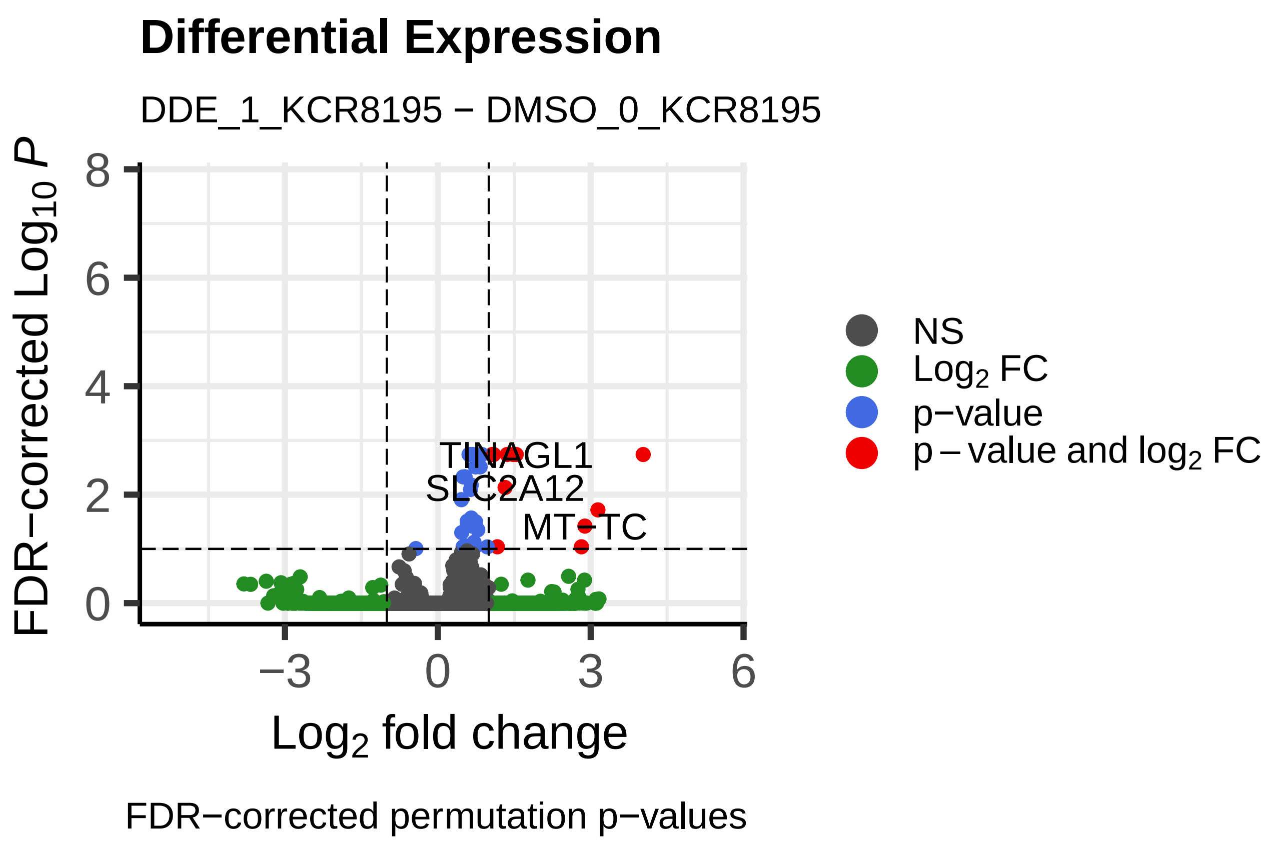
Differential Expression
DDE_1_KCR8195 − DMSO_0_KCR8195
FDR−corrected $Log_{10}$ $P$
0
2
4
6
8
−3
0
3
6
$Log_2$ fold change
TINAGL1
SLC2A12
MT−TC
NS
$Log_2$ FC
p−value
p − value and $log_2$ FC
FDR−corrected permutation p−values

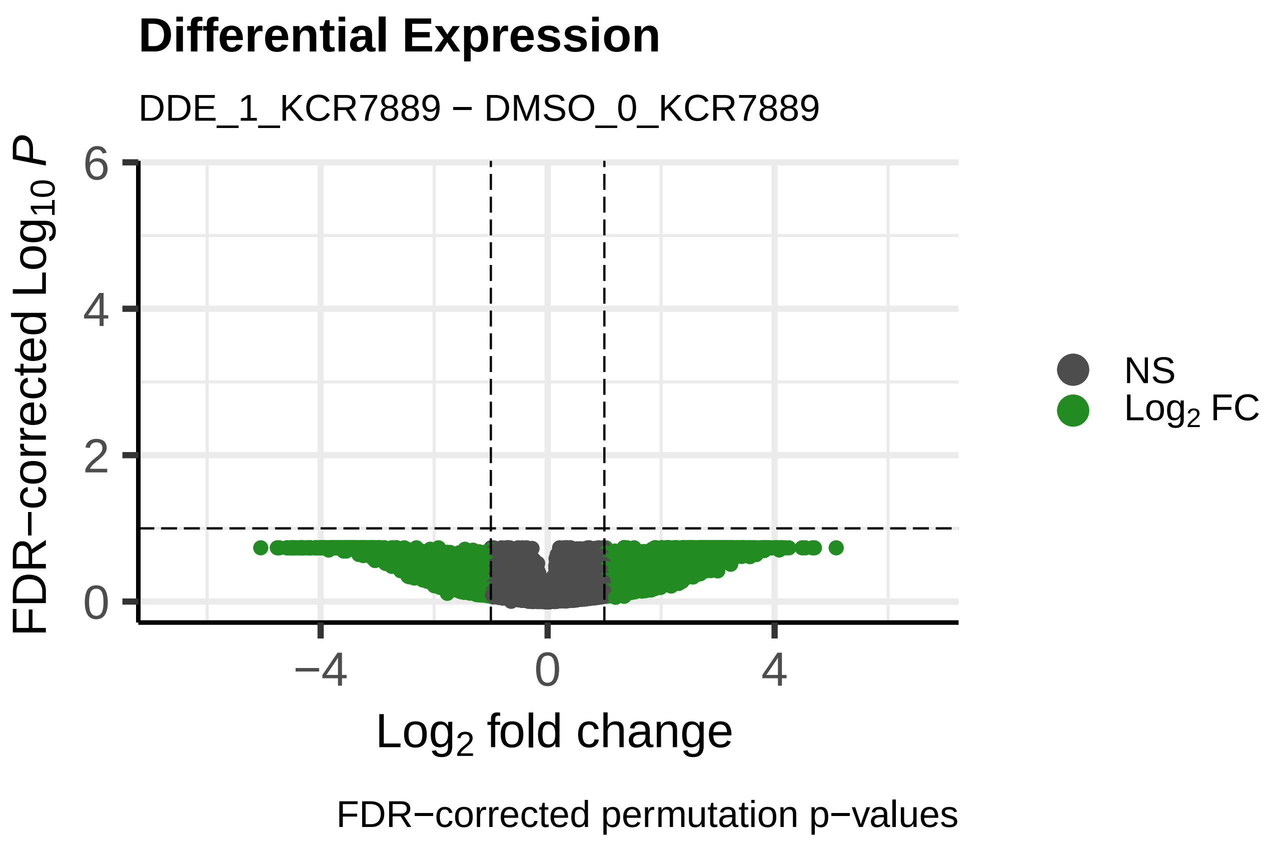
Differential Expression
DDE_1_KCR7889 − DMSO_0_KCR7889
FDR−corrected $Log_{10}$ $P$
0
2
4
6
−4
0
4
$Log_2$ fold change
NS
$Log_2$ FC
FDR−corrected permutation p−values

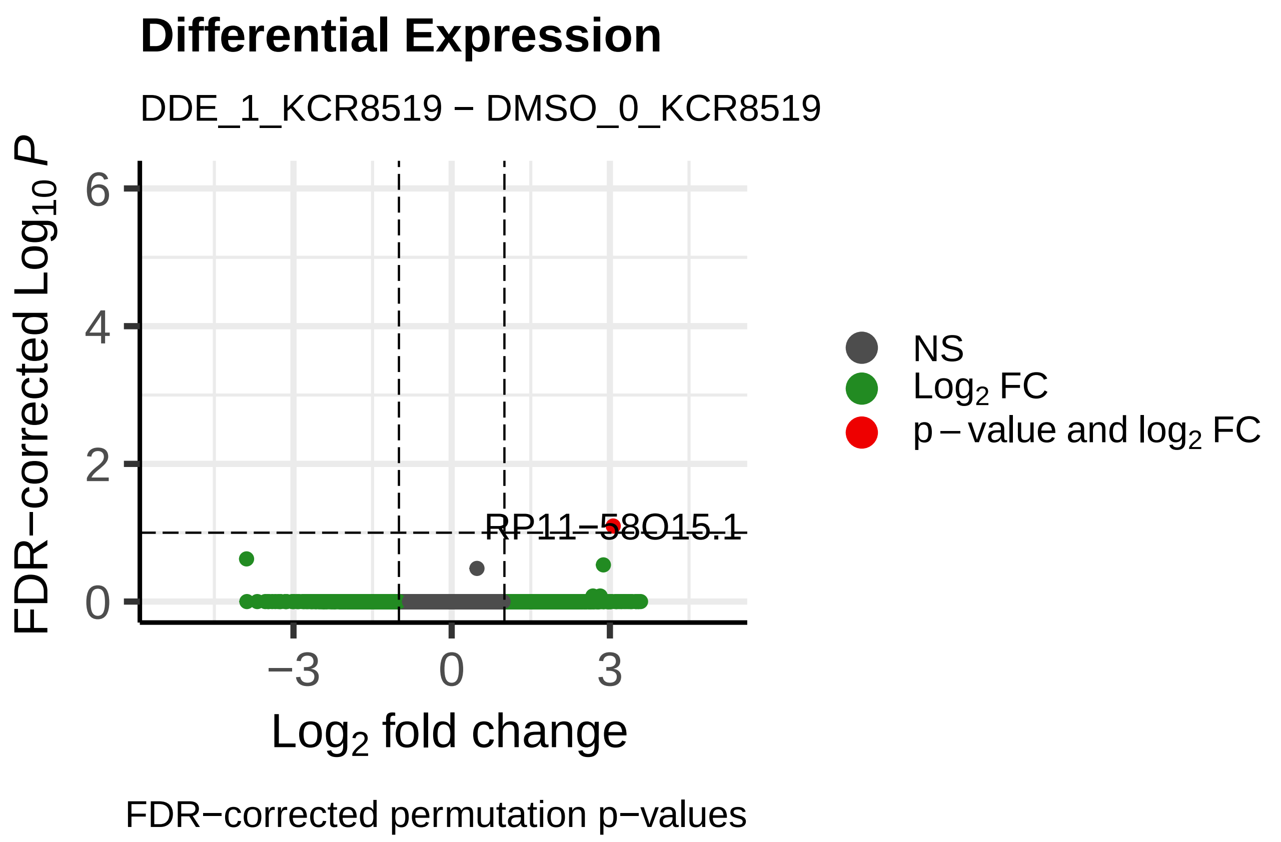
Differential Expression
DDE_1_KCR8519 − DMSO_0_KCR8519
FDR−corrected $Log_{10}$ $P$
0
2
4
6
−3
0
3
$Log_2$ fold change
RP11−58O15.1
NS
$Log_2$ FC
p − value and $log_2$ FC
FDR−corrected permutation p−values

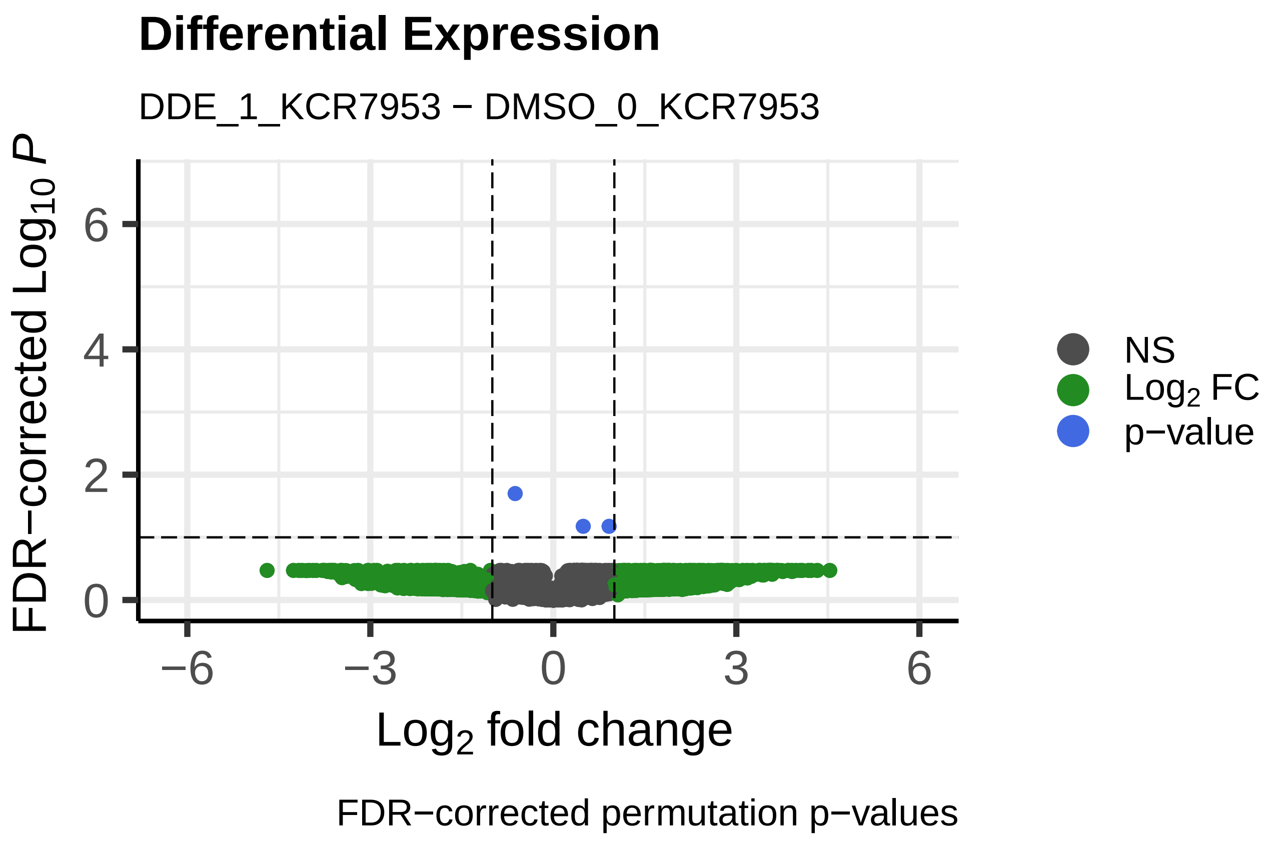
Differential Expression
DDE_1_KCR7953 − DMSO_0_KCR7953
FDR−corrected $Log_{10}$ $P$
0
2
4
6
−6
−3
0
3
6
$Log_2$ fold change
NS
$Log_2$ FC
p−value
FDR−corrected permutation p−values

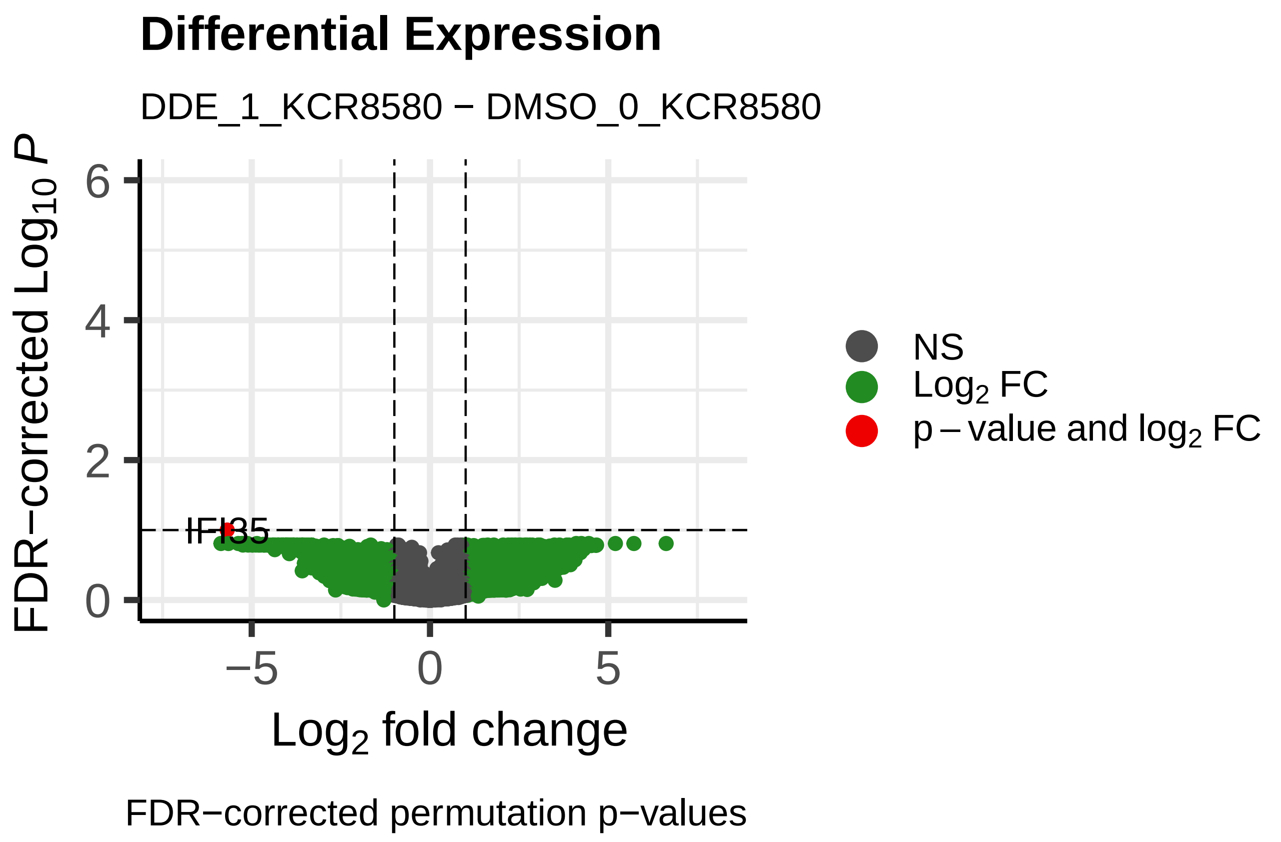
Differential Expression
DDE_1_KCR8580 − DMSO_0_KCR8580
FDR−corrected $Log_{10}$ $P$
0
2
4
6
−5
0
5
$Log_2$ fold change
IFI35
NS
$Log_2$ FC
p − value and $log_2$ FC
FDR−corrected permutation p−values

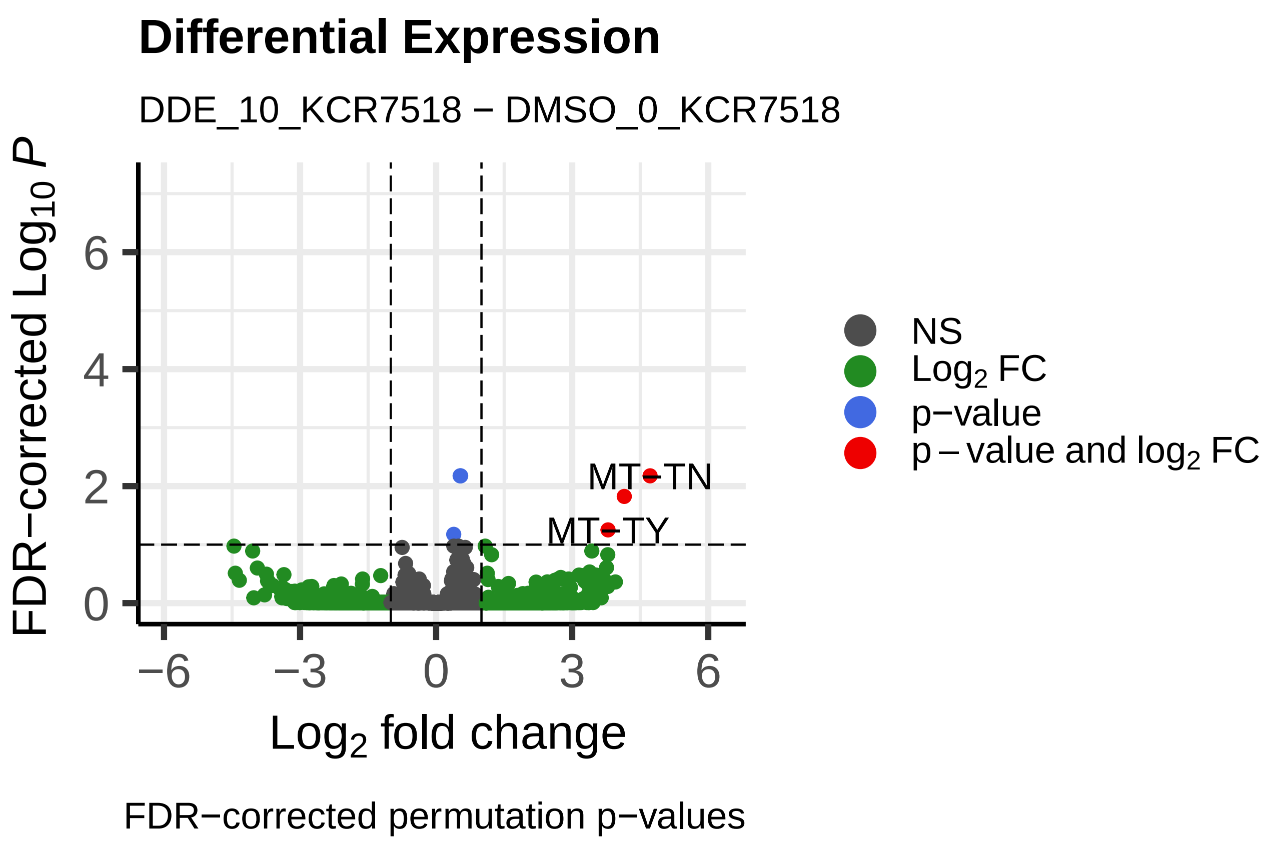
Differential Expression
DDE_10_KCR7518 − DMSO_0_KCR7518
FDR−corrected Log10 P
Log2 fold change
MT−TN
MT−TY
NS
Log2 FC
p−value
p − value and log2 FC
FDR−corrected permutation p−values

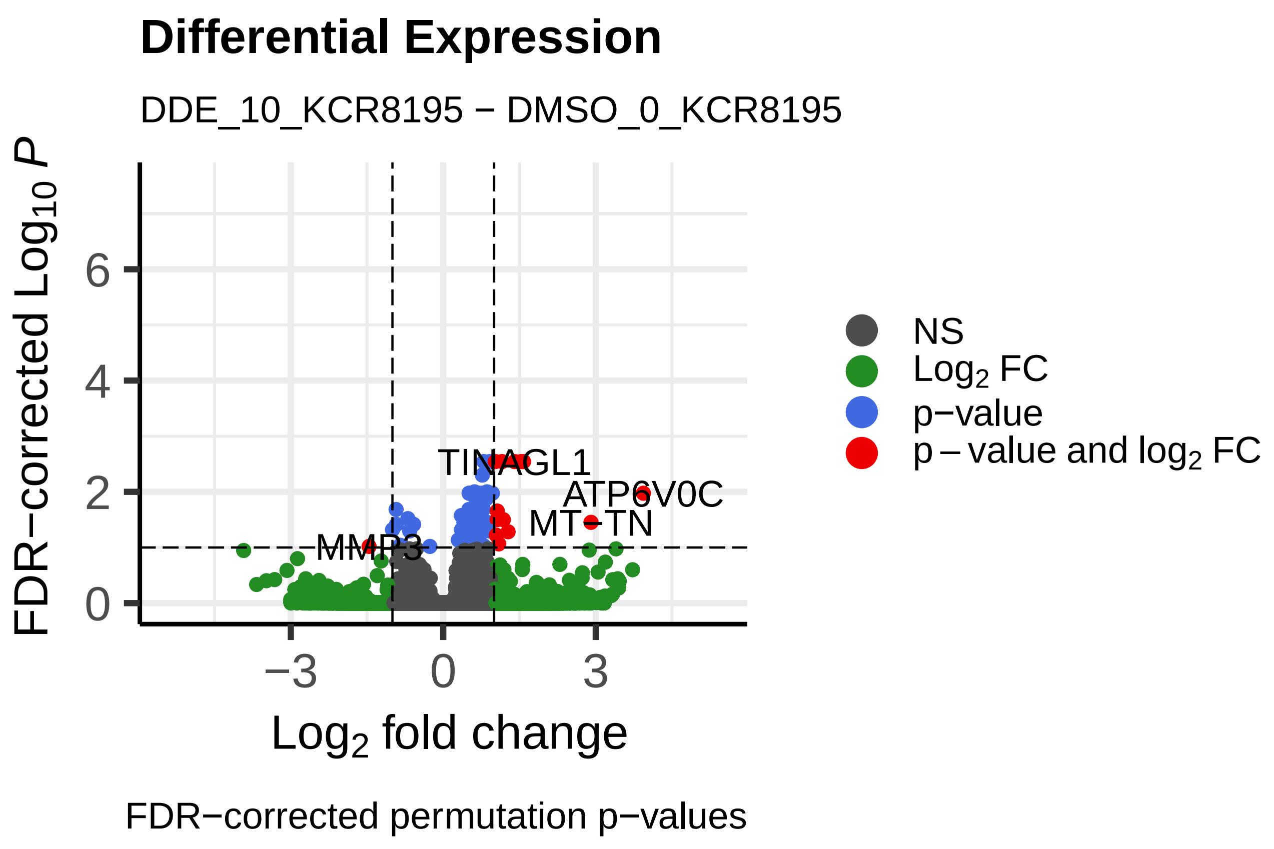
Differential Expression
DDE_10_KCR8195 − DMSO_0_KCR8195
FDR−corrected Log10 P
Log2 fold change
TINAGL1
ATP6V0C
MT−TN
MMP3
NS
Log2 FC
p−value
p − value and log2 FC
FDR−corrected permutation p−values

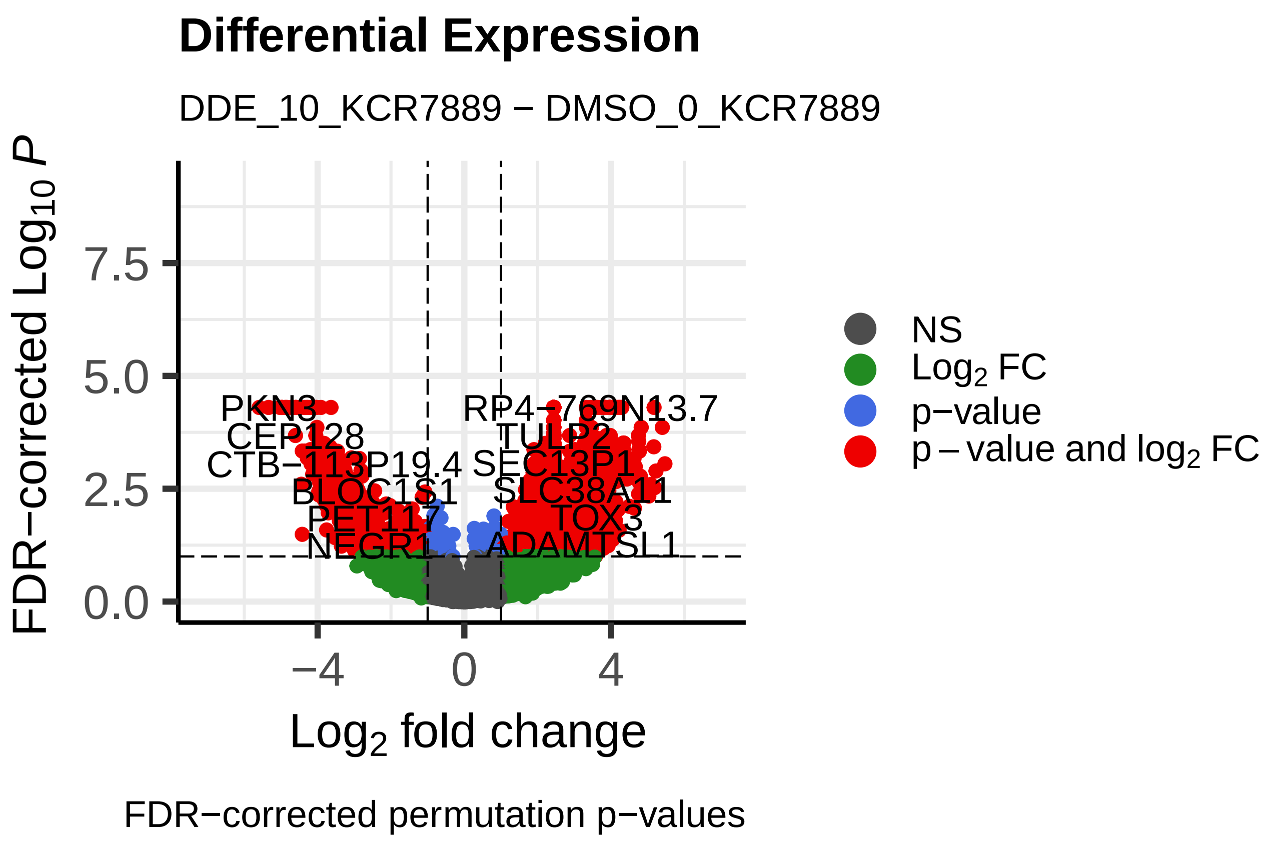
Differential Expression
DDE_10_KCR7889 − DMSO_0_KCR7889
FDR−corrected Log10 P
Log2 fold change
PKN3
CEP128
CTB−113P19.4
BLOC1S1
PET117
NEGR1
RP4−769N13.7
TULP2
SEC13P1
SLC38A11
TOX3
ADAMTSL1
NS
Log2 FC
p−value
p − value and log2 FC
FDR−corrected permutation p−values

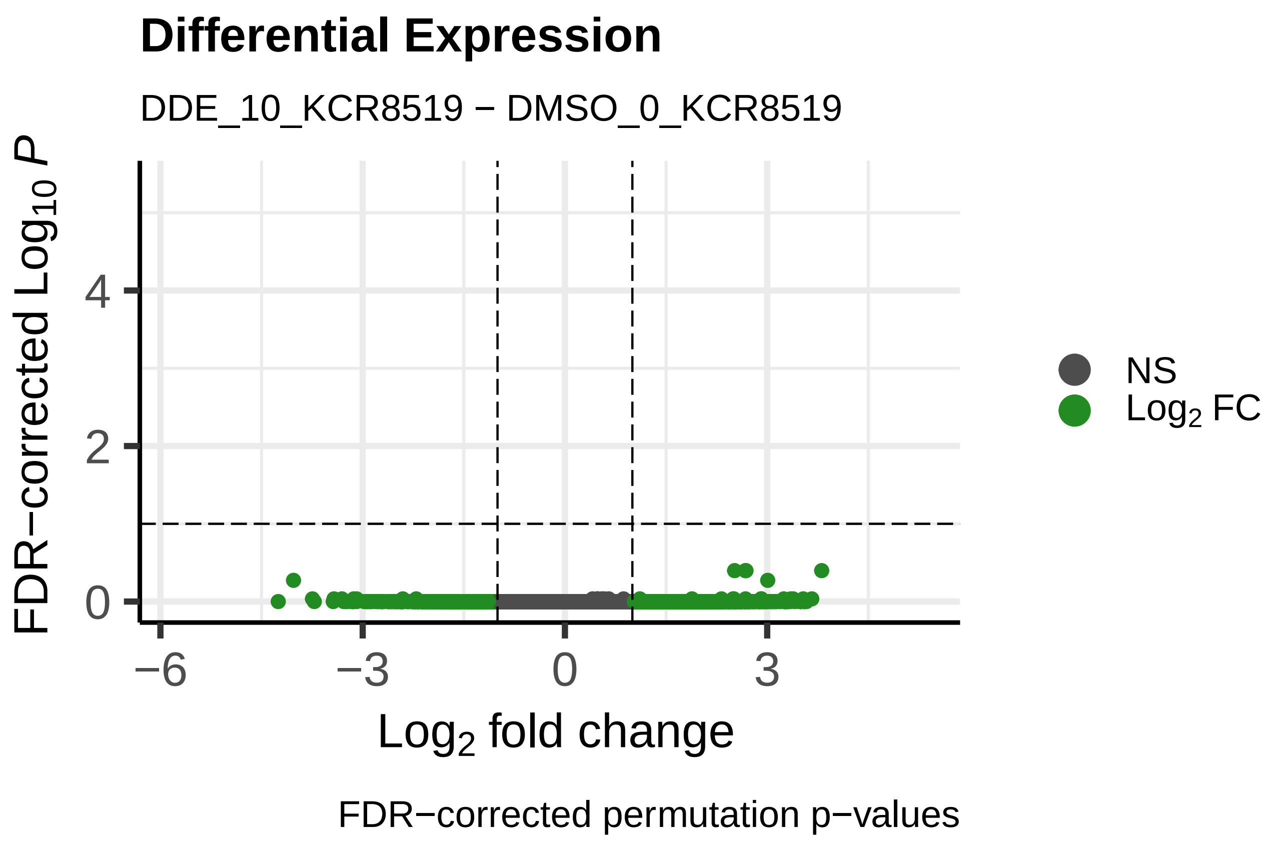
Differential Expression
DDE_10_KCR8519 − DMSO_0_KCR8519
FDR−corrected Log10 P
Log2 fold change
NS
Log2 FC
FDR−corrected permutation p−values

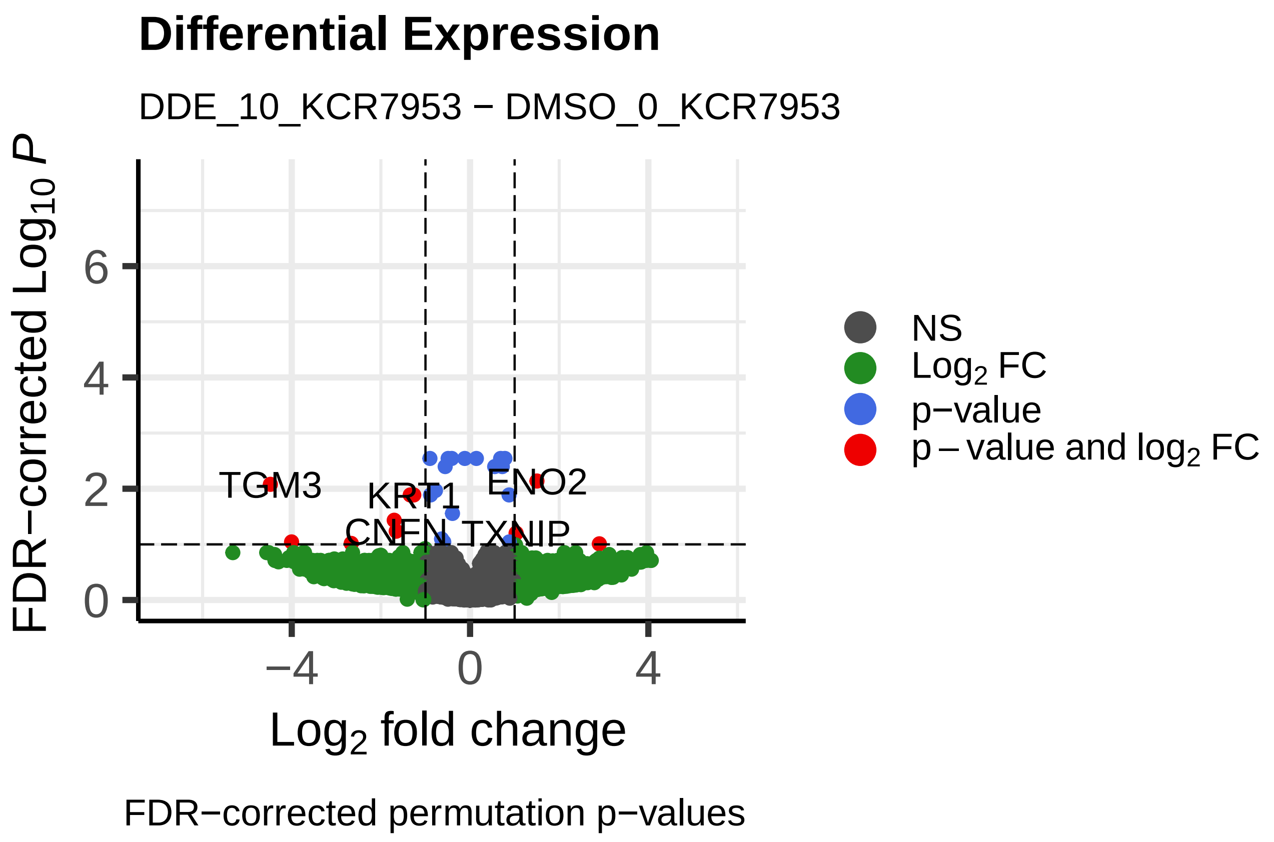
Differential Expression
DDE_10_KCR7953 − DMSO_0_KCR7953
FDR−corrected Log10 P
Log2 fold change
TGM3
KRT1
ENO2
CNFN
TXNIP
NS
Log2 FC
p−value
p − value and log2 FC
FDR−corrected permutation p−values

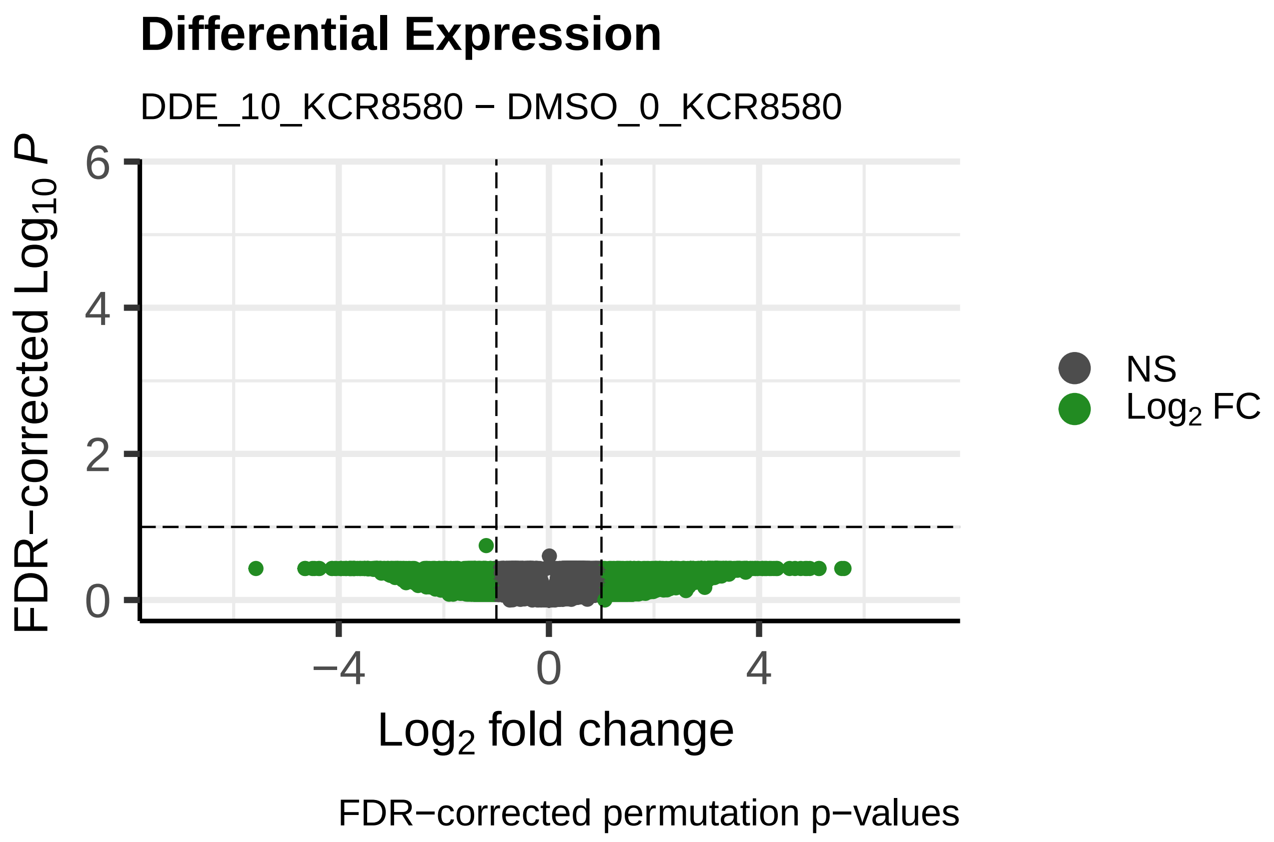
Differential Expression
DDE_10_KCR8580 − DMSO_0_KCR8580
FDR−corrected Log10 P
Log2 fold change
NS
Log2 FC
FDR−corrected permutation p−values

**Differential Expression**

PFNA_0.1_KCR7518 − DMSO_0_KCR7518

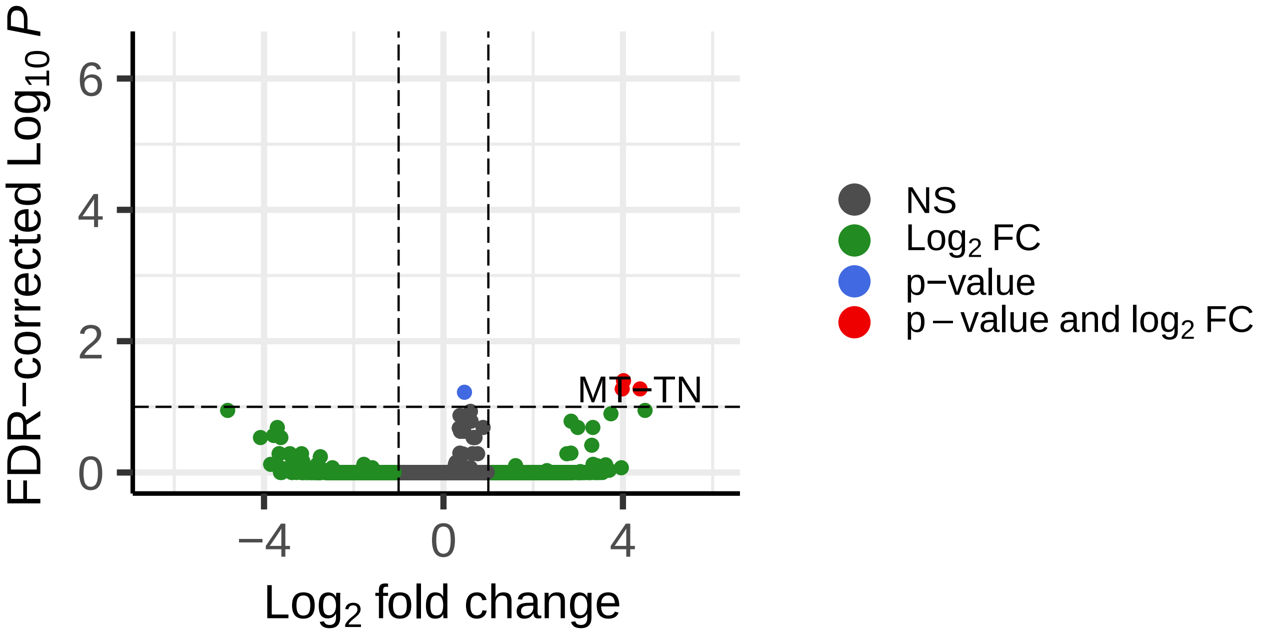


FDR−corrected permutation p−values

**Differential Expression**

PFNA_0.1_KCR8195 − DMSO_0_KCR8195

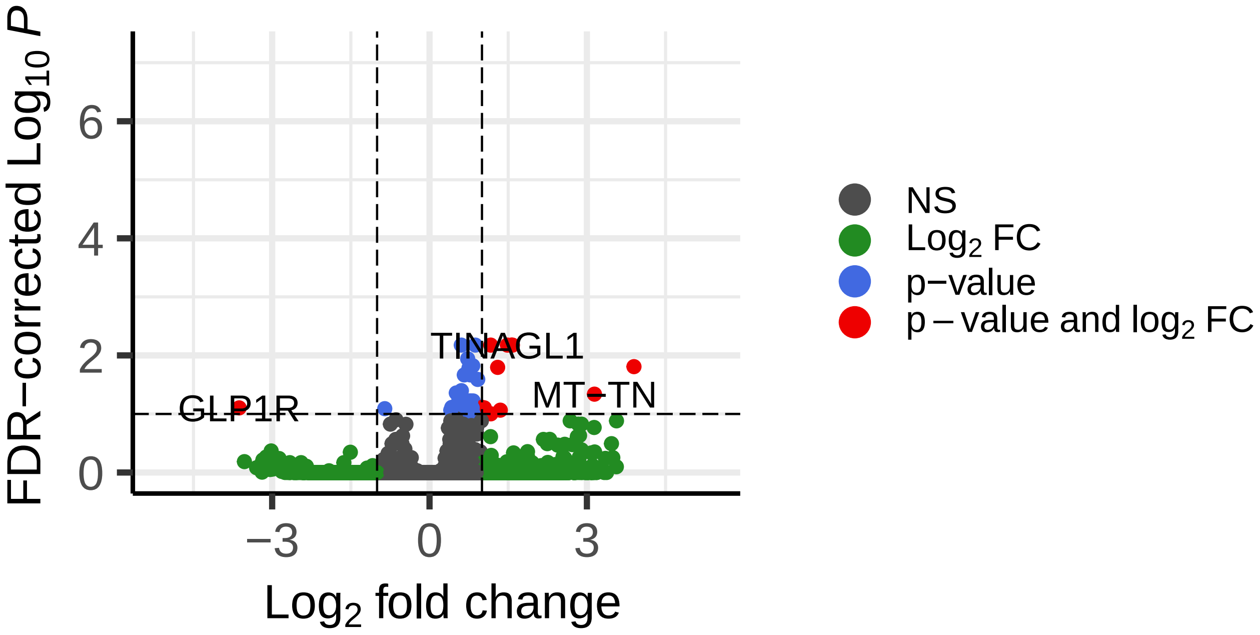


FDR−corrected permutation p−values

**Differential Expression**

PFNA_0.1_KCR7889 − DMSO_0_KCR7889

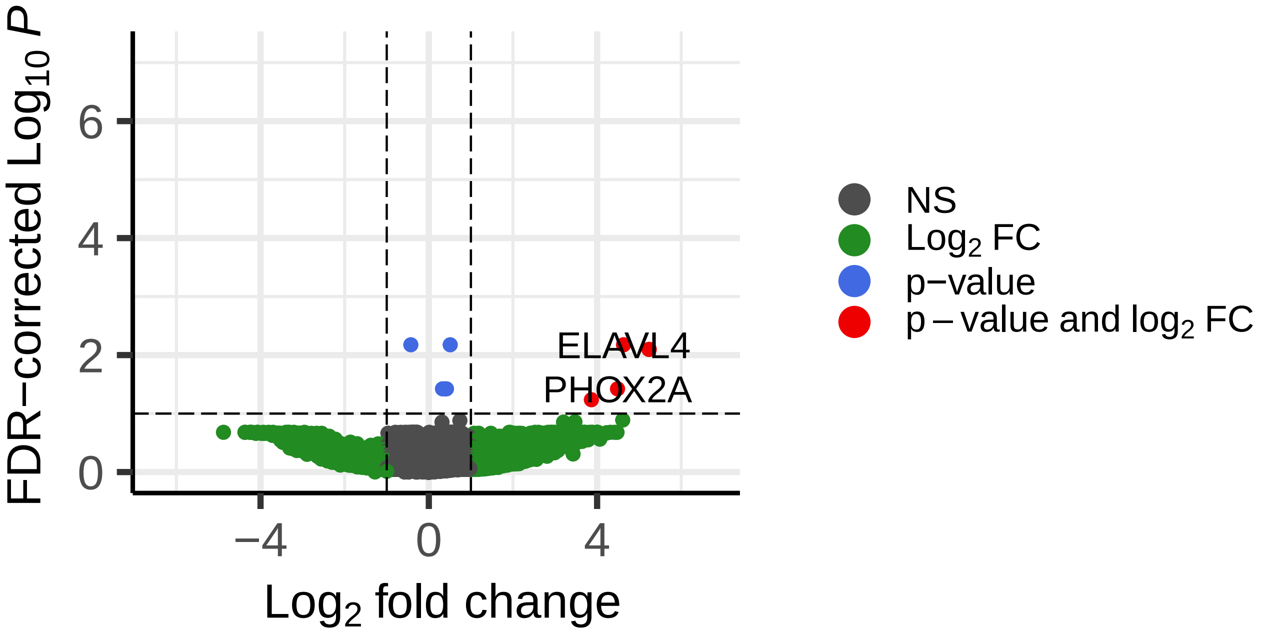


FDR−corrected permutation p−values

**Differential Expression**

PFNA_0.1_KCR8519 − DMSO_0_KCR8519

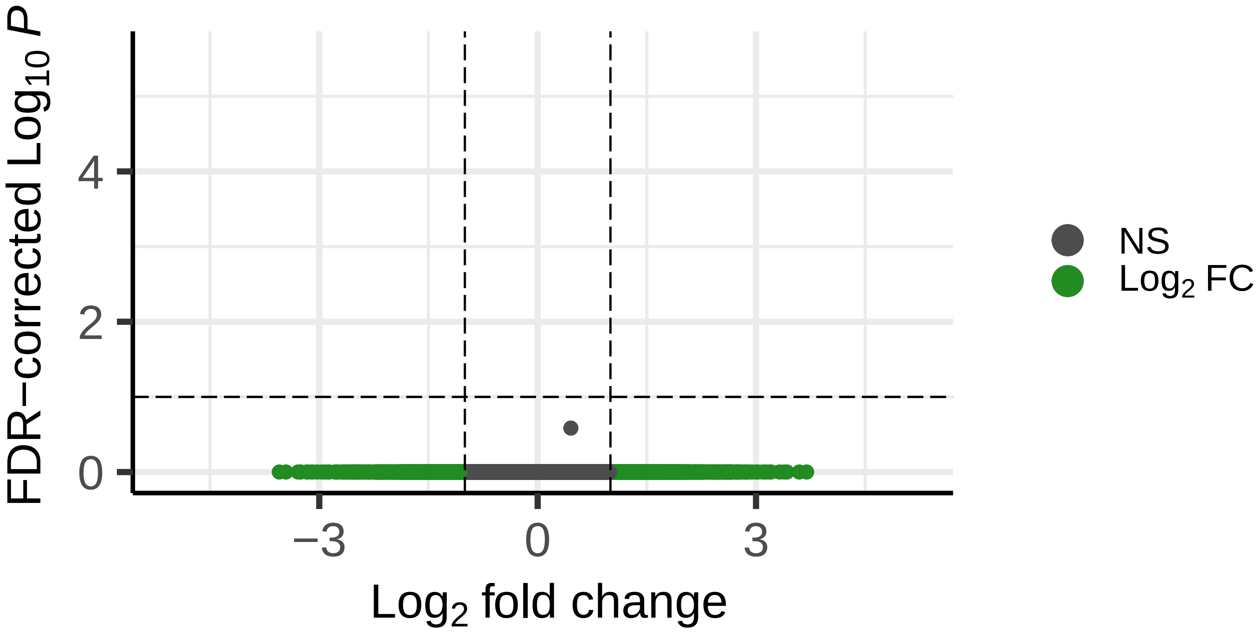


FDR−corrected permutation p−values

**Differential Expression**

PFNA_0.1_KCR7953 − DMSO_0_KCR7953

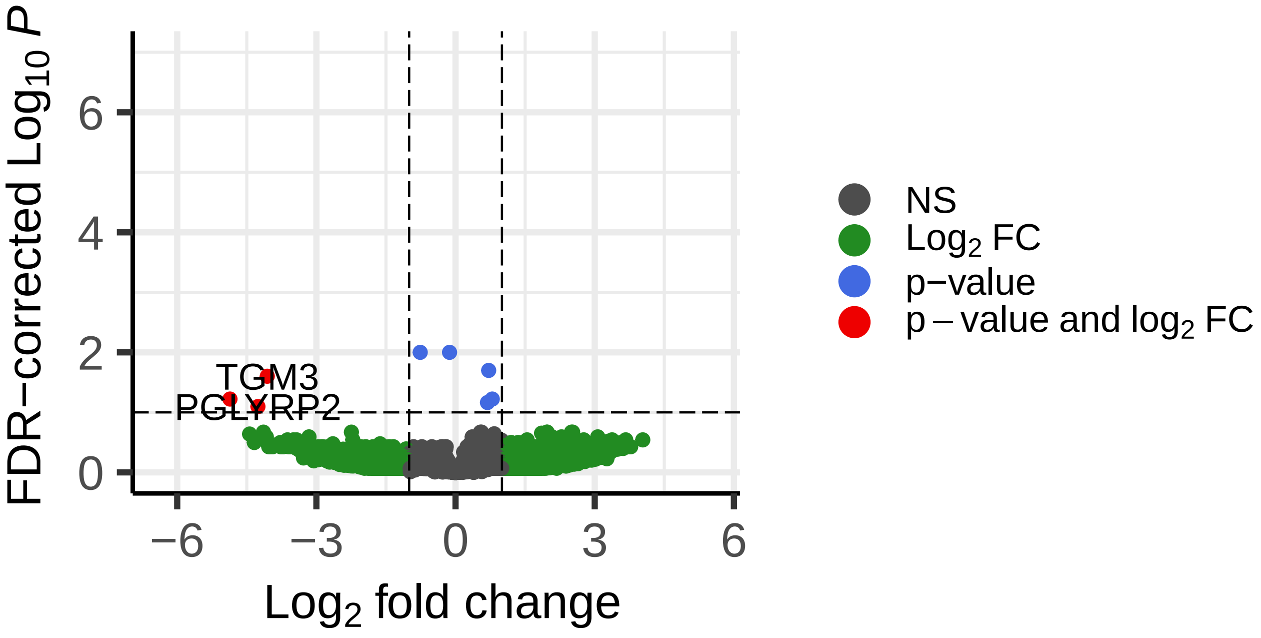


FDR−corrected permutation p−values

**Differential Expression**

PFNA_0.1_KCR8580 − DMSO_0_KCR8580

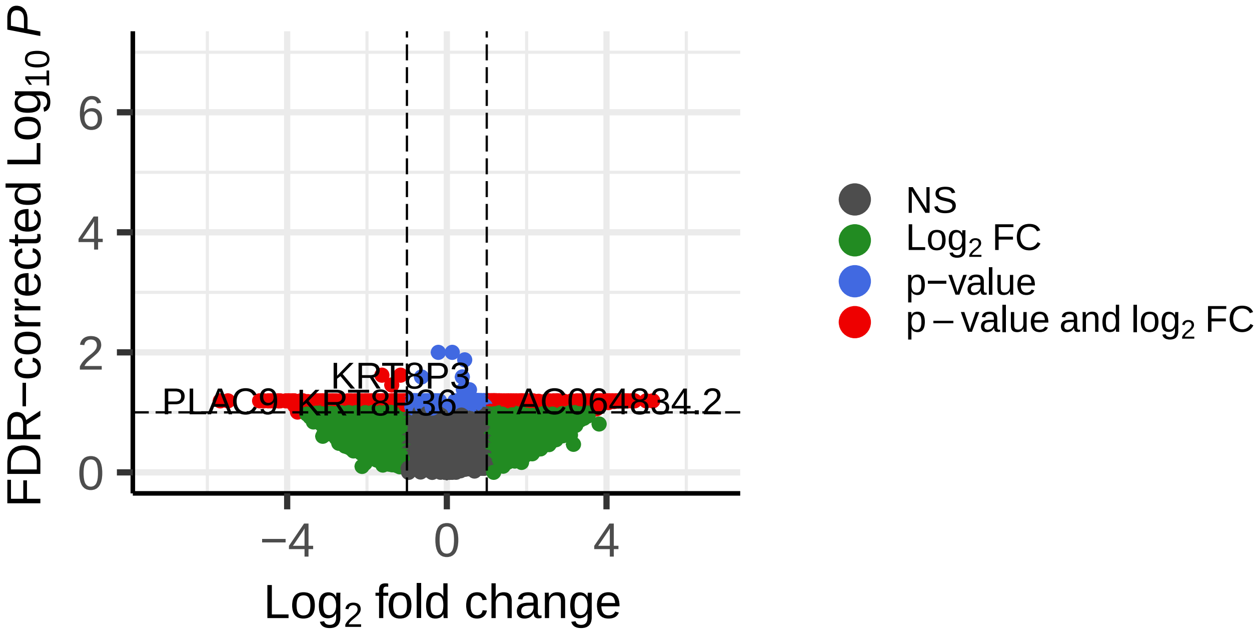


FDR−corrected permutation p−values

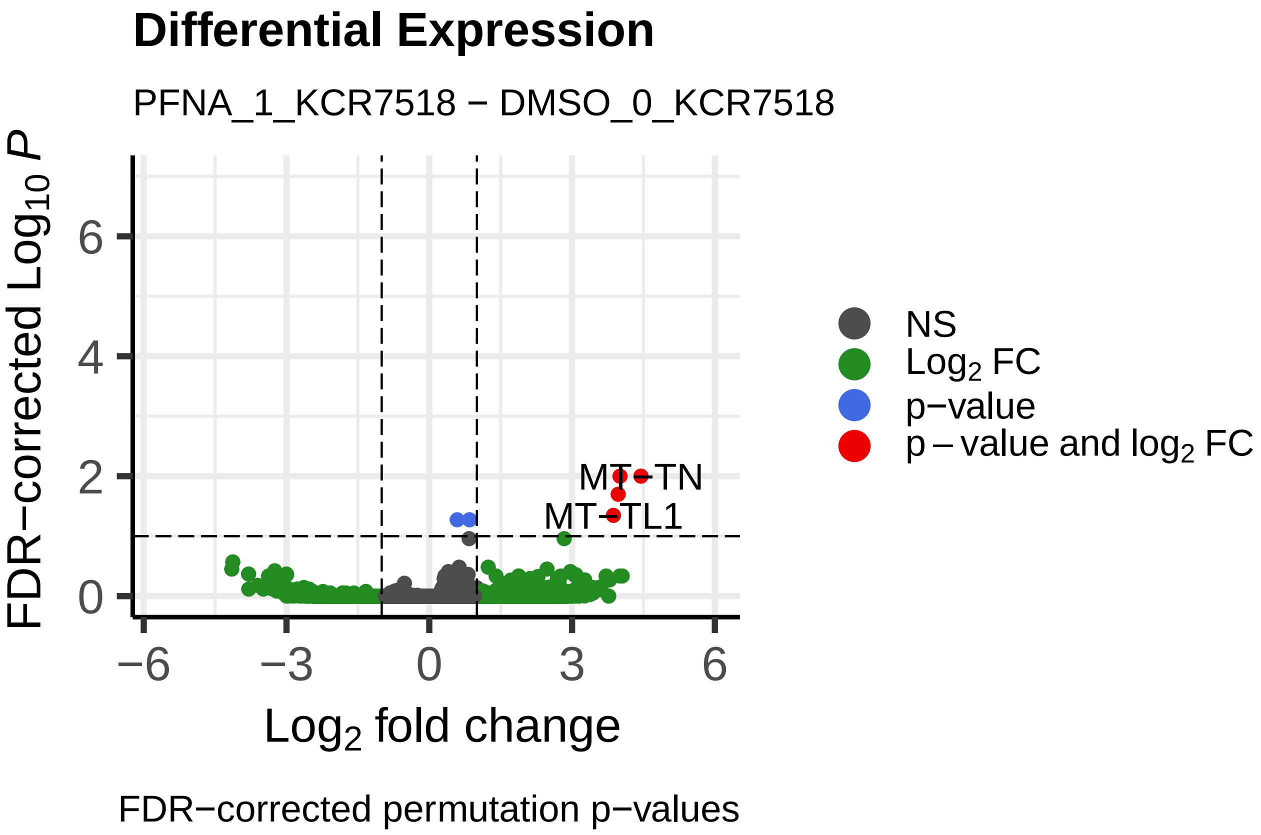
Differential Expression
PFNA_1_KCR7518 − DMSO_0_KCR7518
FDR−corrected $Log_{10}$ $P$
Log2 fold change
MT−TN
MT−TL1
NS
Log2 FC
p−value
p − value and log2 FC
FDR−corrected permutation p−values

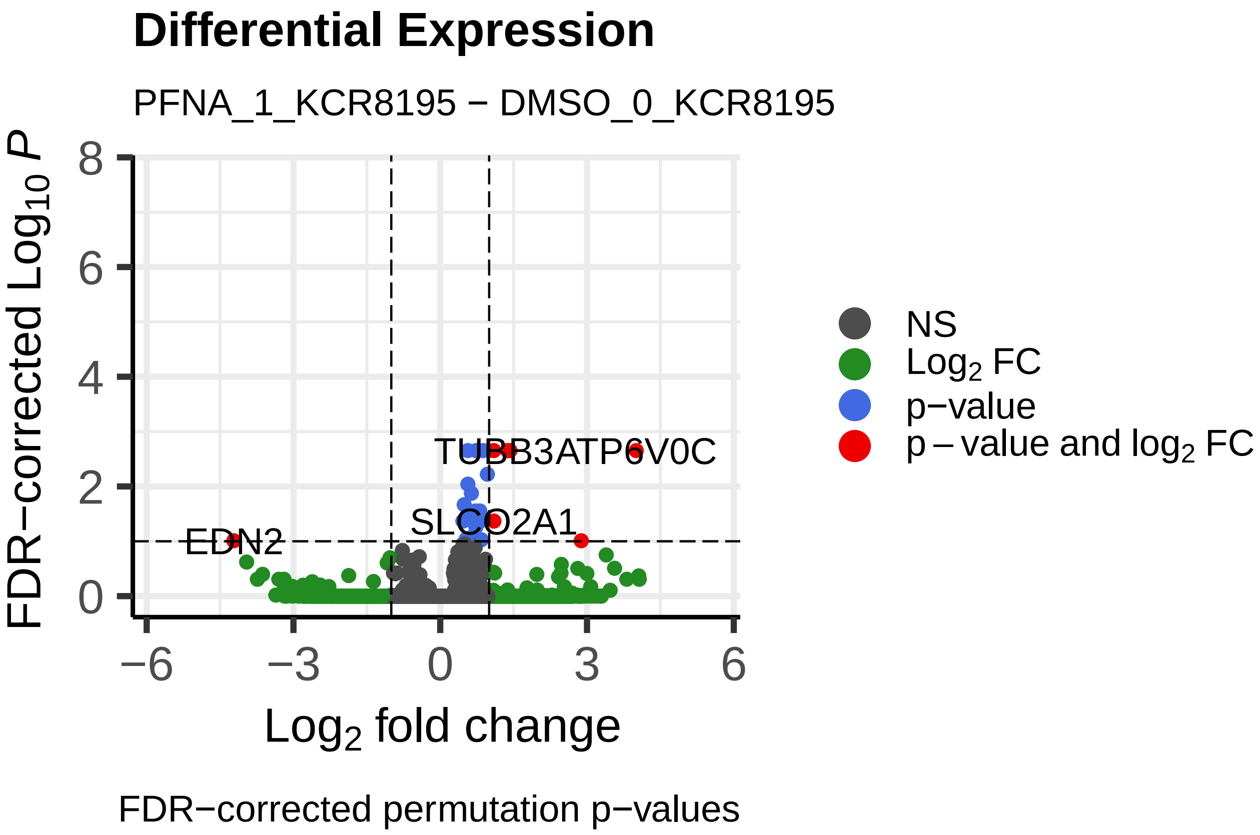
Differential Expression
PFNA_1_KCR8195 − DMSO_0_KCR8195
FDR−corrected $Log_{10}$ $P$
Log2 fold change
TUBB3
ATP6V0C
SLCO2A1
EDN2
NS
Log2 FC
p−value
p − value and log2 FC
FDR−corrected permutation p−values

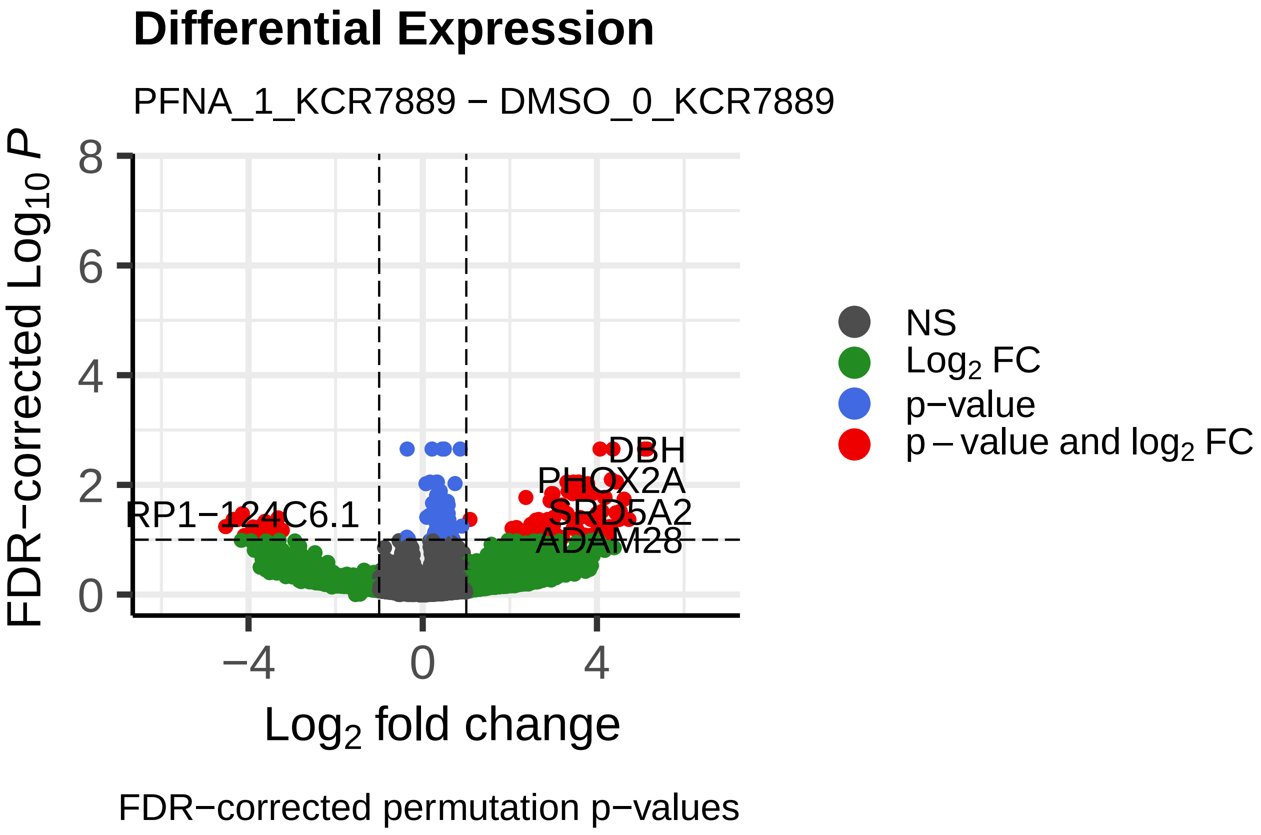
Differential Expression
PFNA_1_KCR7889 − DMSO_0_KCR7889
FDR−corrected $Log_{10}$ $P$
Log2 fold change
DBH
PHOX2A
SRD5A2
ADAM28
RP1−124C6.1
NS
Log2 FC
p−value
p − value and log2 FC
FDR−corrected permutation p−values

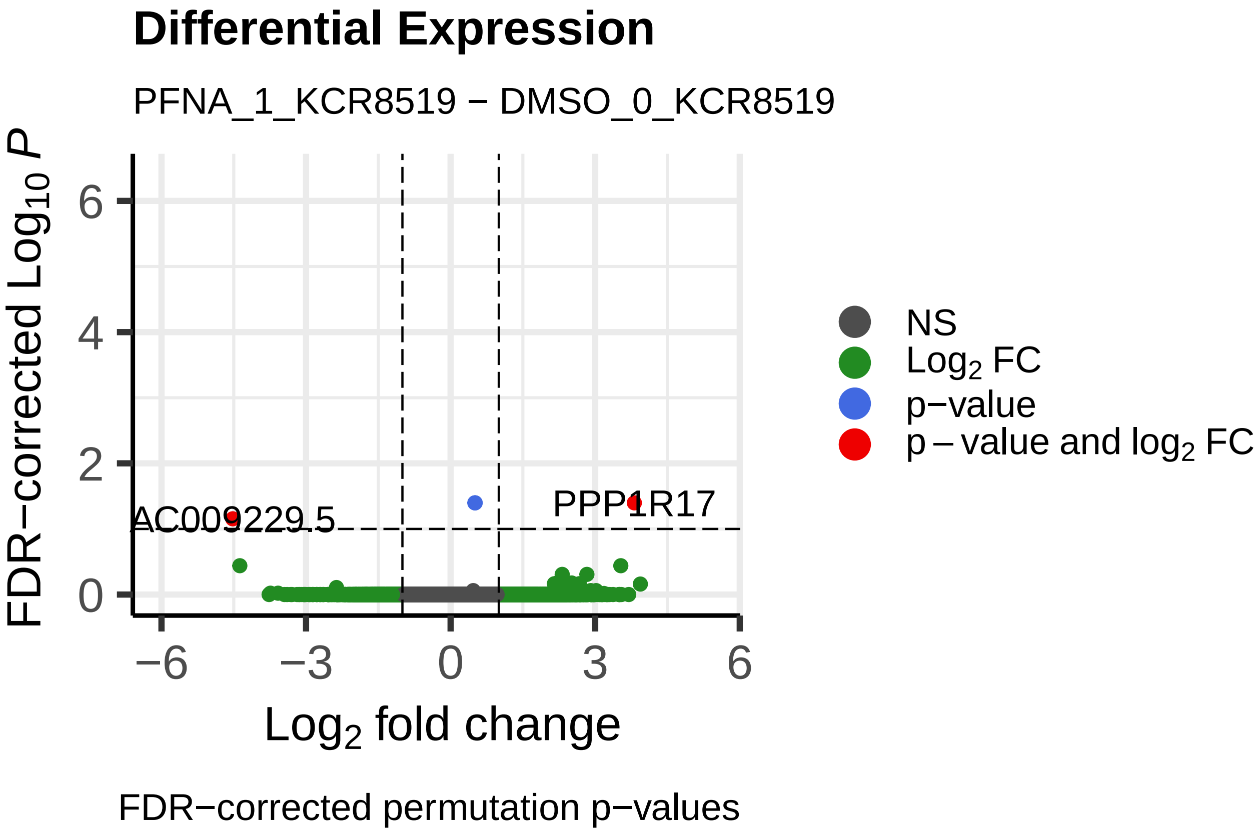
Differential Expression
PFNA_1_KCR8519 − DMSO_0_KCR8519
FDR−corrected $Log_{10}$ $P$
Log2 fold change
PPP1R17
AC009229.5
NS
Log2 FC
p−value
p − value and log2 FC
FDR−corrected permutation p−values

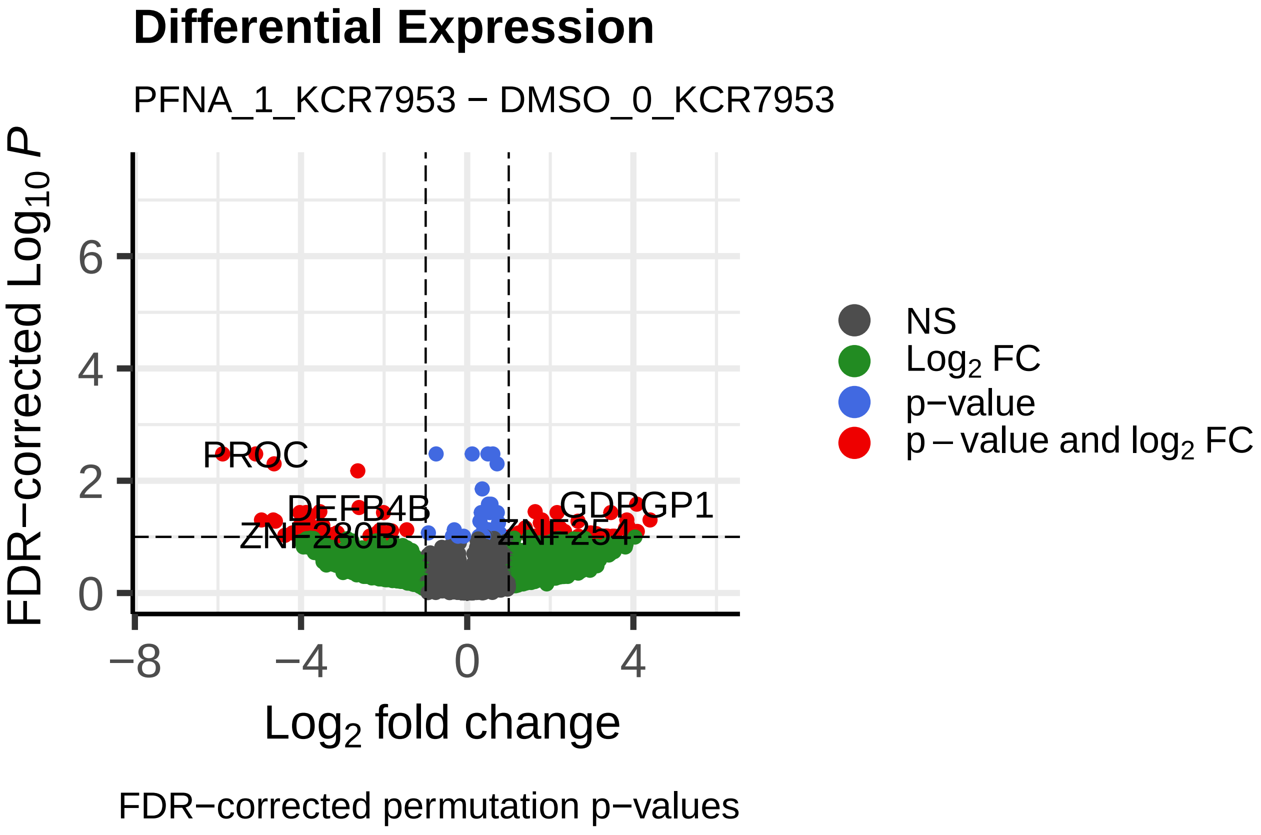
Differential Expression
PFNA_1_KCR7953 − DMSO_0_KCR7953
FDR−corrected $Log_{10}$ $P$
Log2 fold change
PROC
DEFB4B
ZNF280B
GDPGP1
ZNF254
NS
Log2 FC
p−value
p − value and log2 FC
FDR−corrected permutation p−values

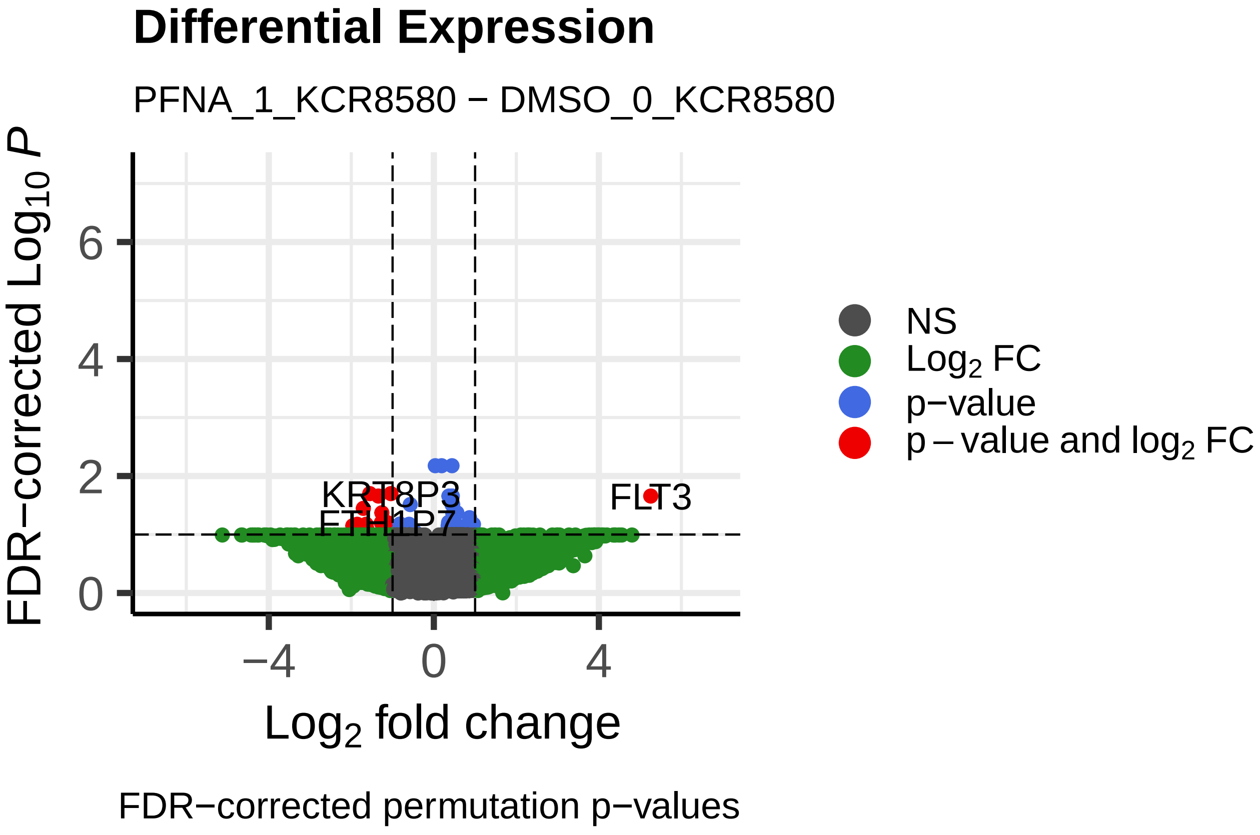
Differential Expression
PFNA_1_KCR8580 − DMSO_0_KCR8580
FDR−corrected $Log_{10}$ $P$
Log2 fold change
KRT8P3
FLT3
NS
Log2 FC
p−value
p − value and log2 FC
FDR−corrected permutation p−values

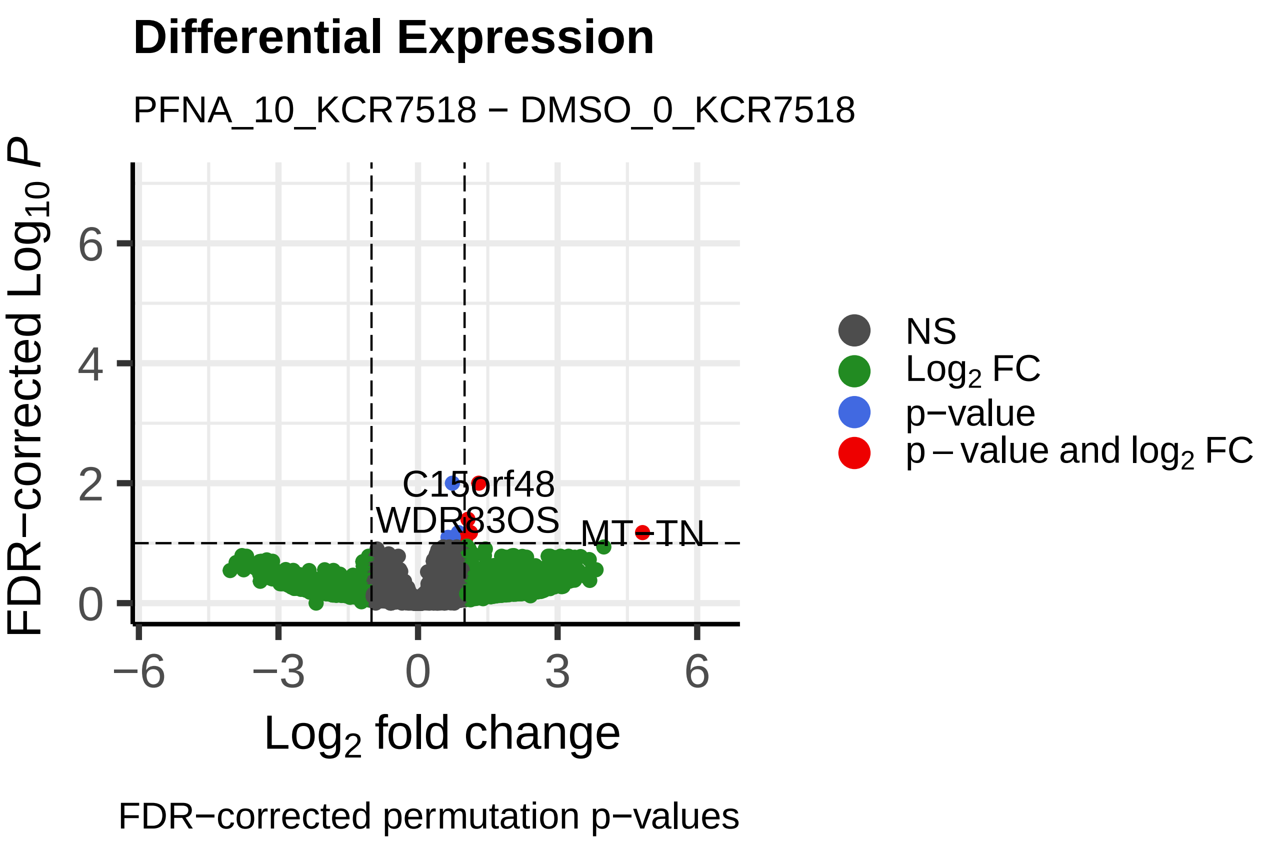
Differential Expression
PFNA_10_KCR7518 − DMSO_0_KCR7518
FDR−corrected $Log_{10}$ P
Log2 fold change
C15orf48
WDR83OS
MT−TN
NS
Log2 FC
p−value
p − value and log2 FC
FDR−corrected permutation p−values

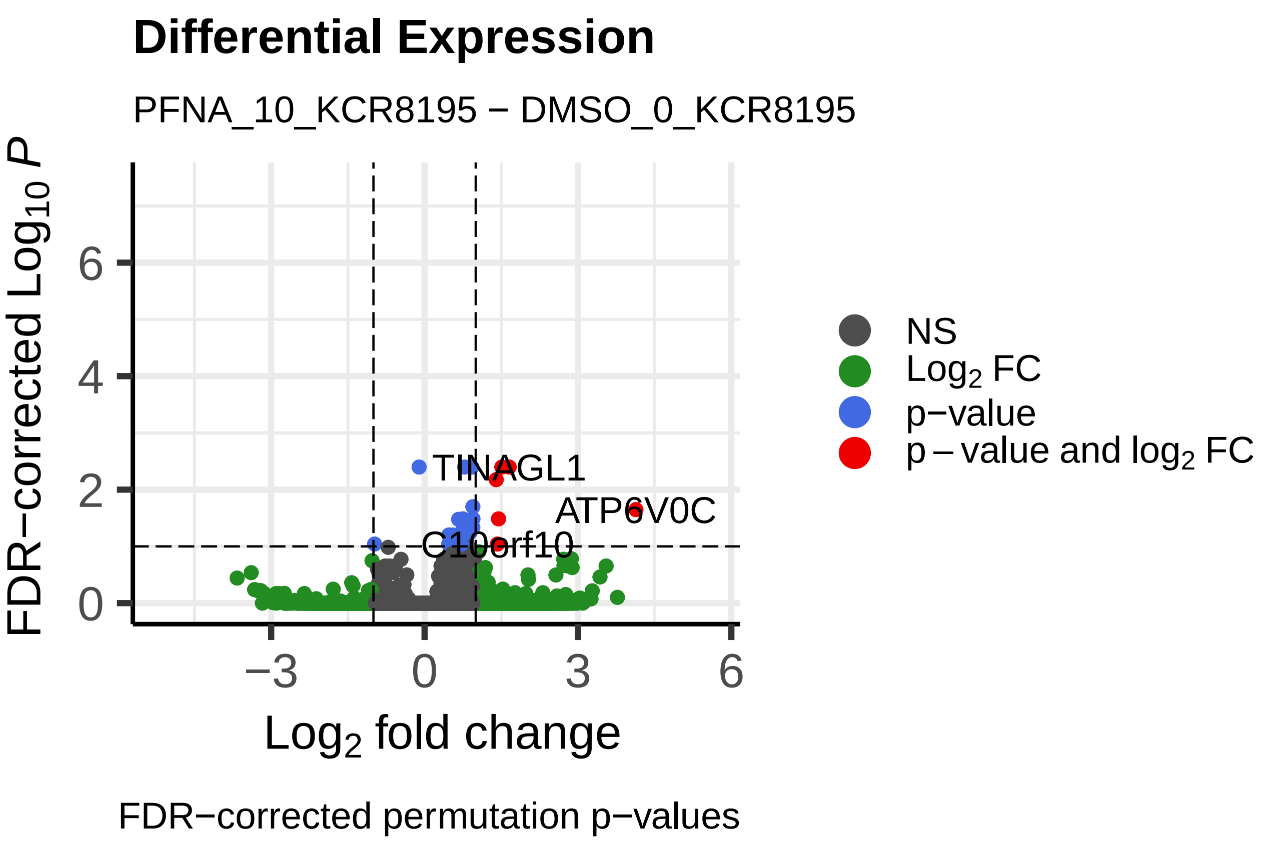
Differential Expression
PFNA_10_KCR8195 − DMSO_0_KCR8195
FDR−corrected $Log_{10}$ P
Log2 fold change
TINAGL1
ATP6V0C
C10orf10
NS
Log2 FC
p−value
p − value and log2 FC
FDR−corrected permutation p−values

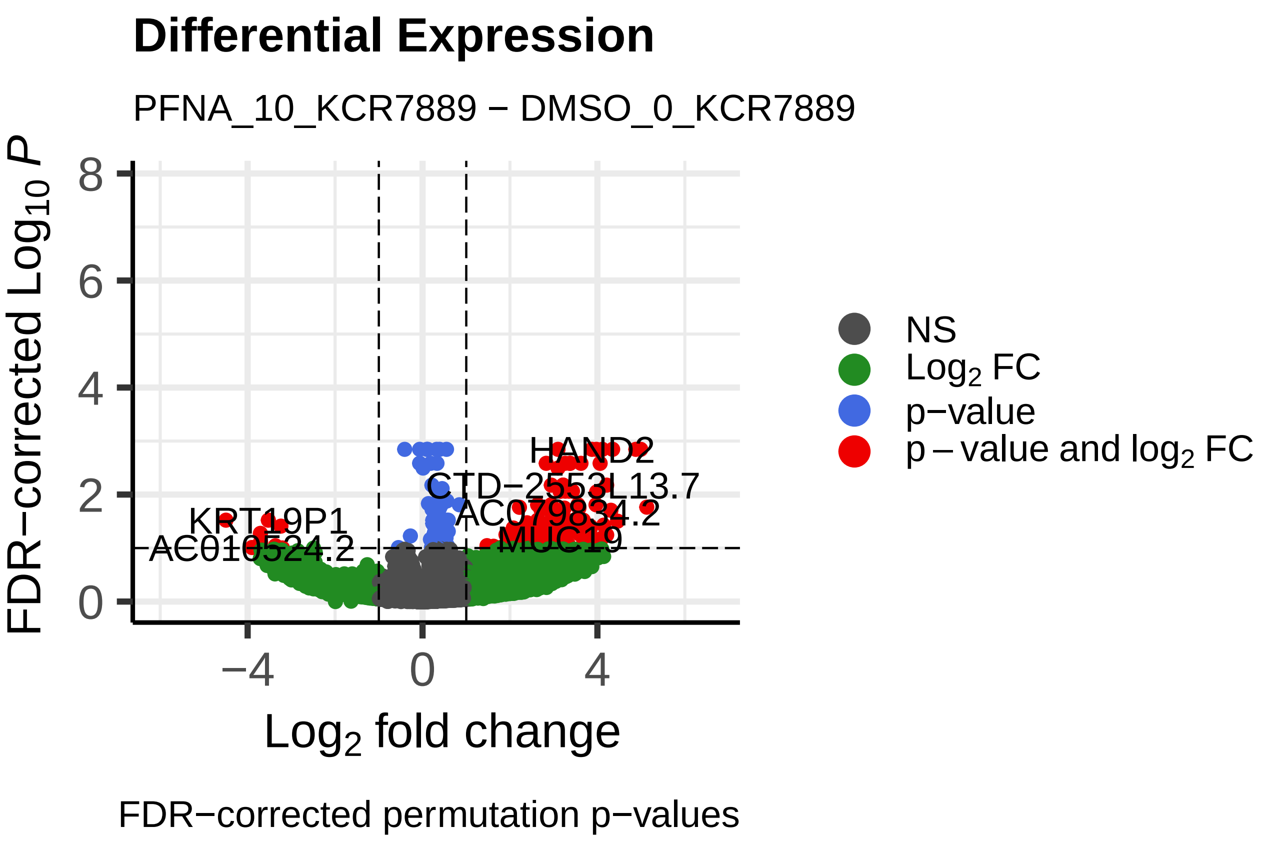
Differential Expression
PFNA_10_KCR7889 − DMSO_0_KCR7889
FDR−corrected $Log_{10}$ P
Log2 fold change
HAND2
CTD−2553L13.7
KRT19P1
AC010524.2
MUC19
NS
Log2 FC
p−value
p − value and log2 FC
FDR−corrected permutation p−values

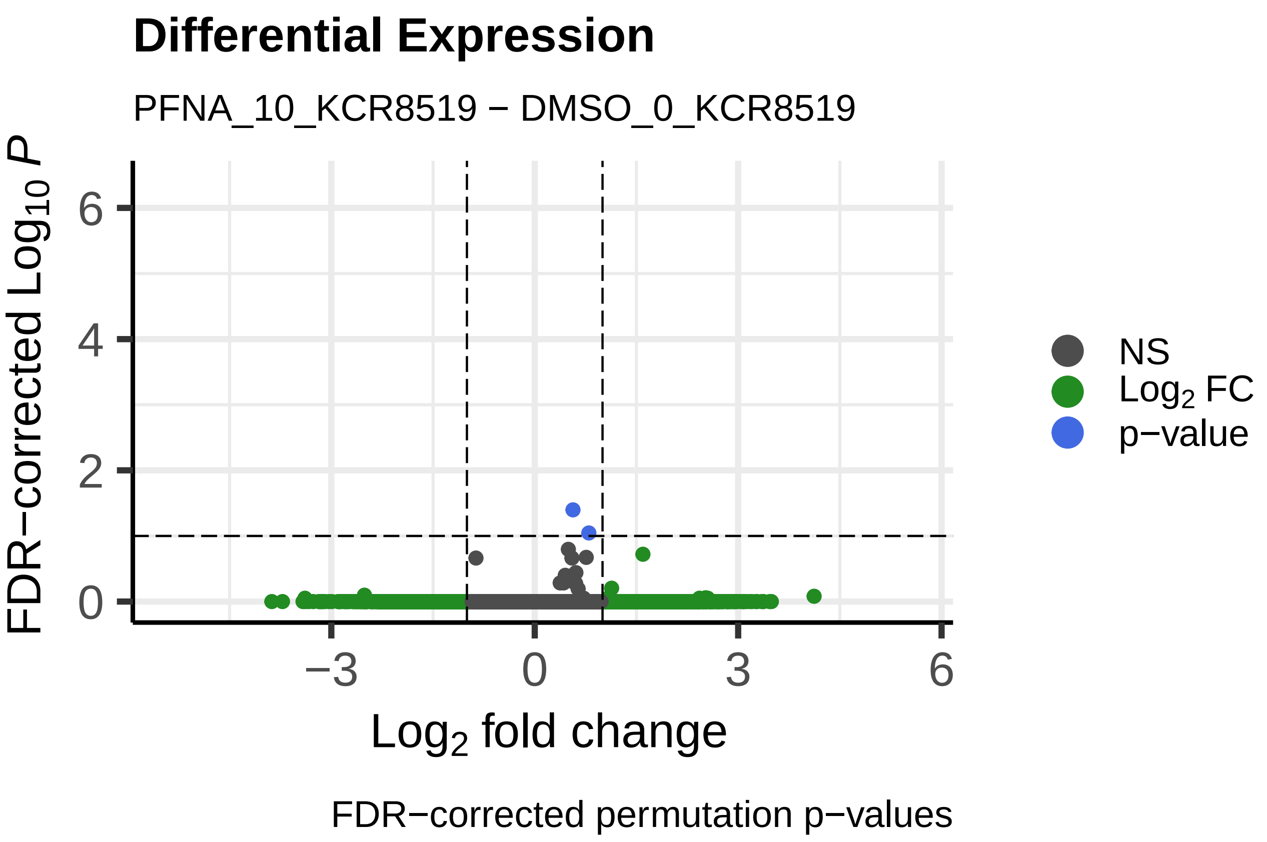
Differential Expression
PFNA_10_KCR8519 − DMSO_0_KCR8519
FDR−corrected $Log_{10}$ P
Log2 fold change
NS
Log2 FC
p−value
FDR−corrected permutation p−values

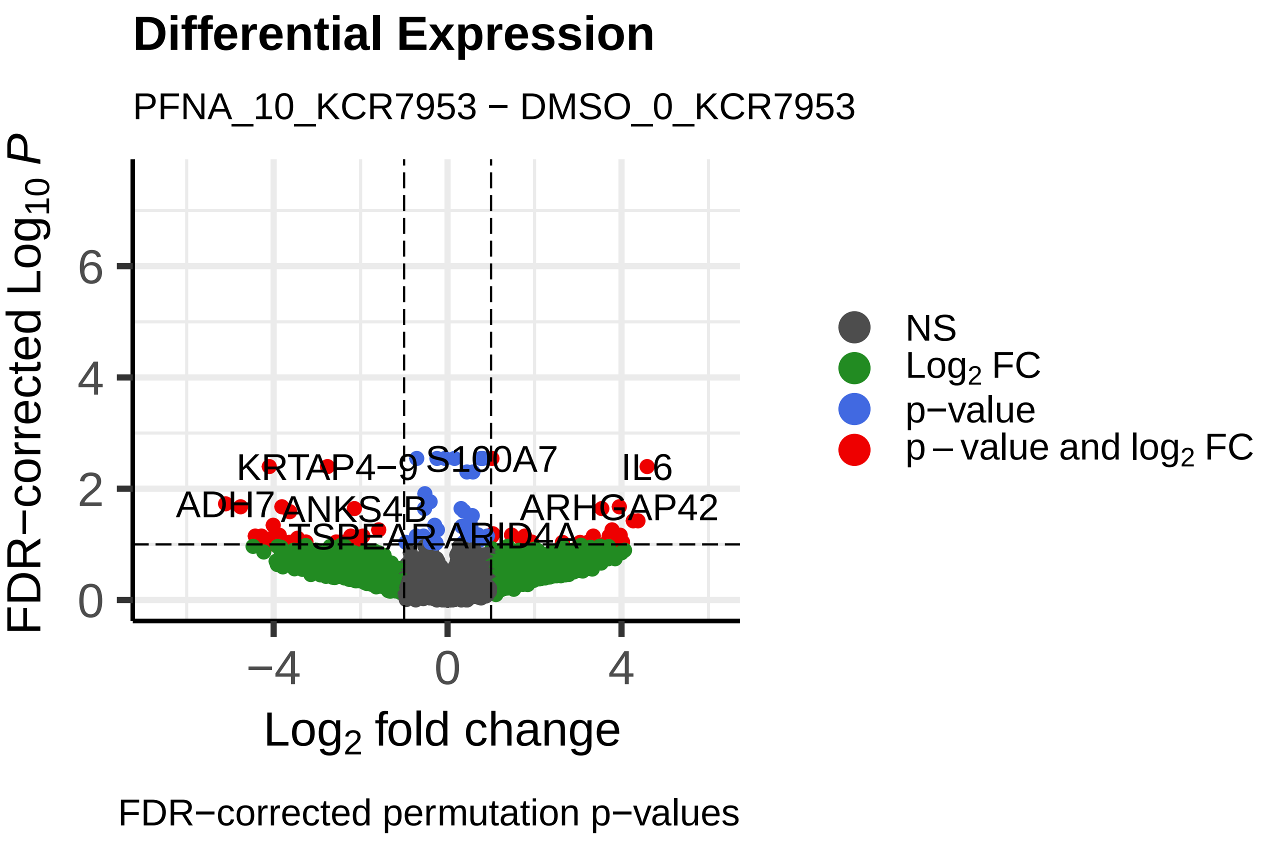
Differential Expression
PFNA_10_KCR7953 − DMSO_0_KCR7953
FDR−corrected $Log_{10}$ P
Log2 fold change
KRTAP4−9
S100A7
IL6
ADH7
ANKS4B
ARHGAP42
ARID4A
NS
Log2 FC
p−value
p − value and log2 FC
FDR−corrected permutation p−values

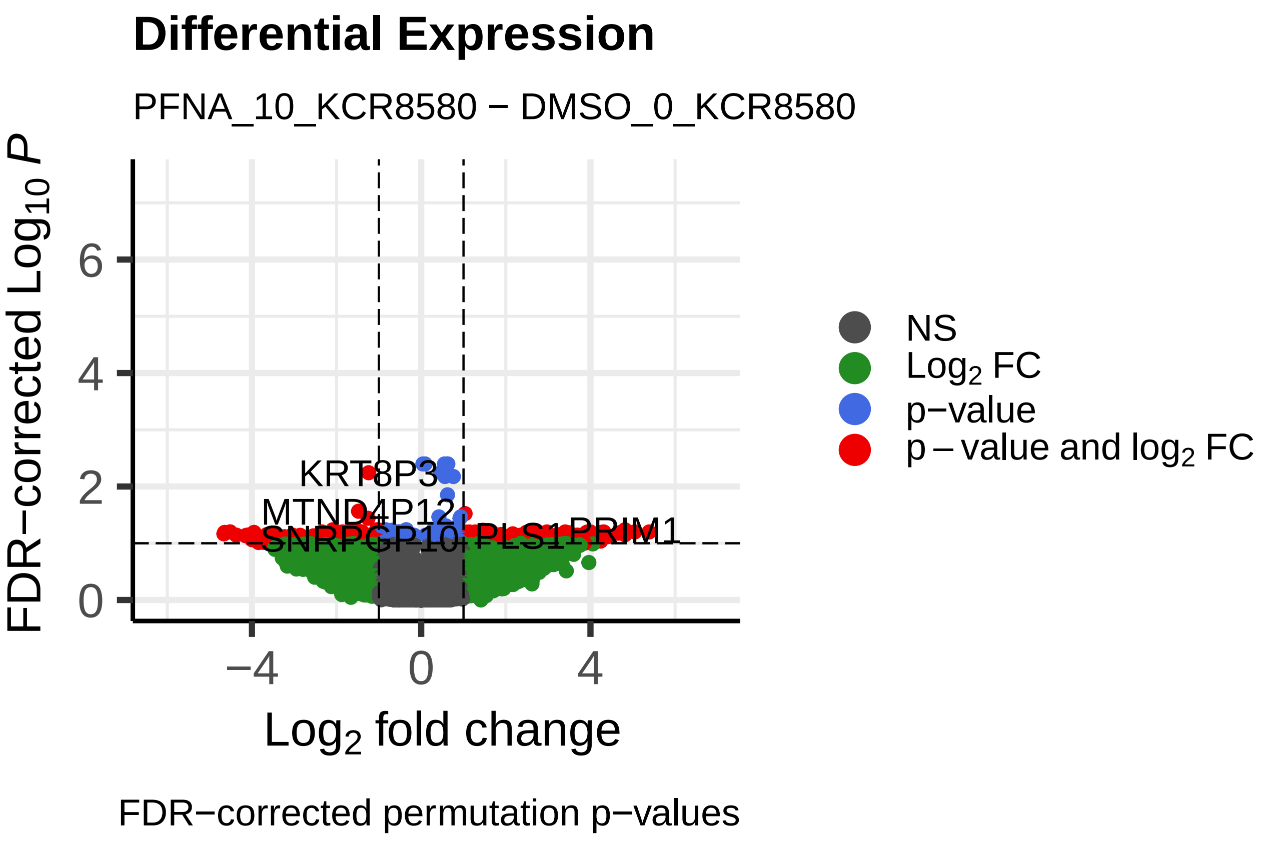
Differential Expression
PFNA_10_KCR8580 − DMSO_0_KCR8580
FDR−corrected $Log_{10}$ P
Log2 fold change
KRT8P3
MTND4P12
PLS1
NS
Log2 FC
p−value
p − value and log2 FC
FDR−corrected permutation p−values

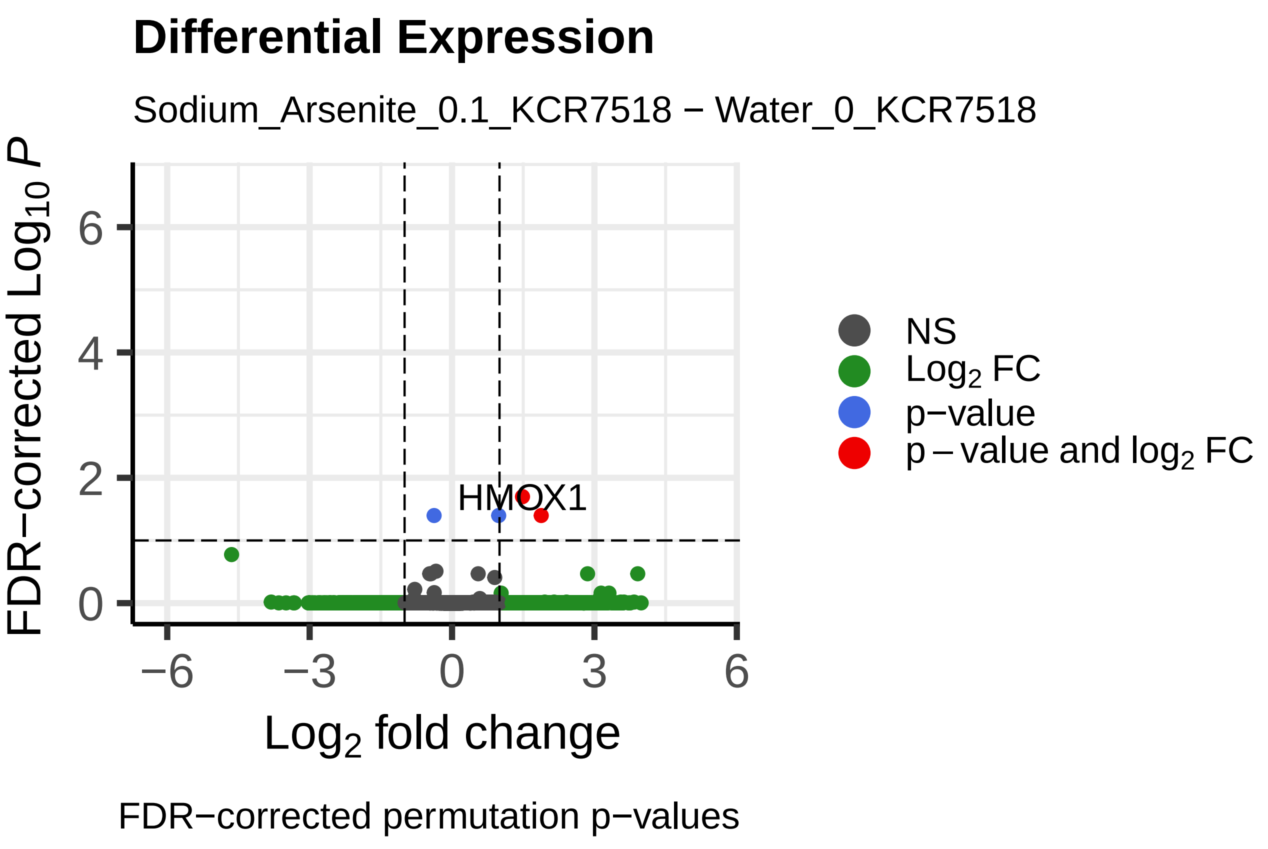

Differential Expression
Sodium_Arsenite_0.1_KCR7518 − Water_0_KCR7518
FDR−corrected Log10 P
Log2 fold change
HMOX1
NS
Log2 FC
p−value
p − value and log2 FC
FDR−corrected permutation p−values


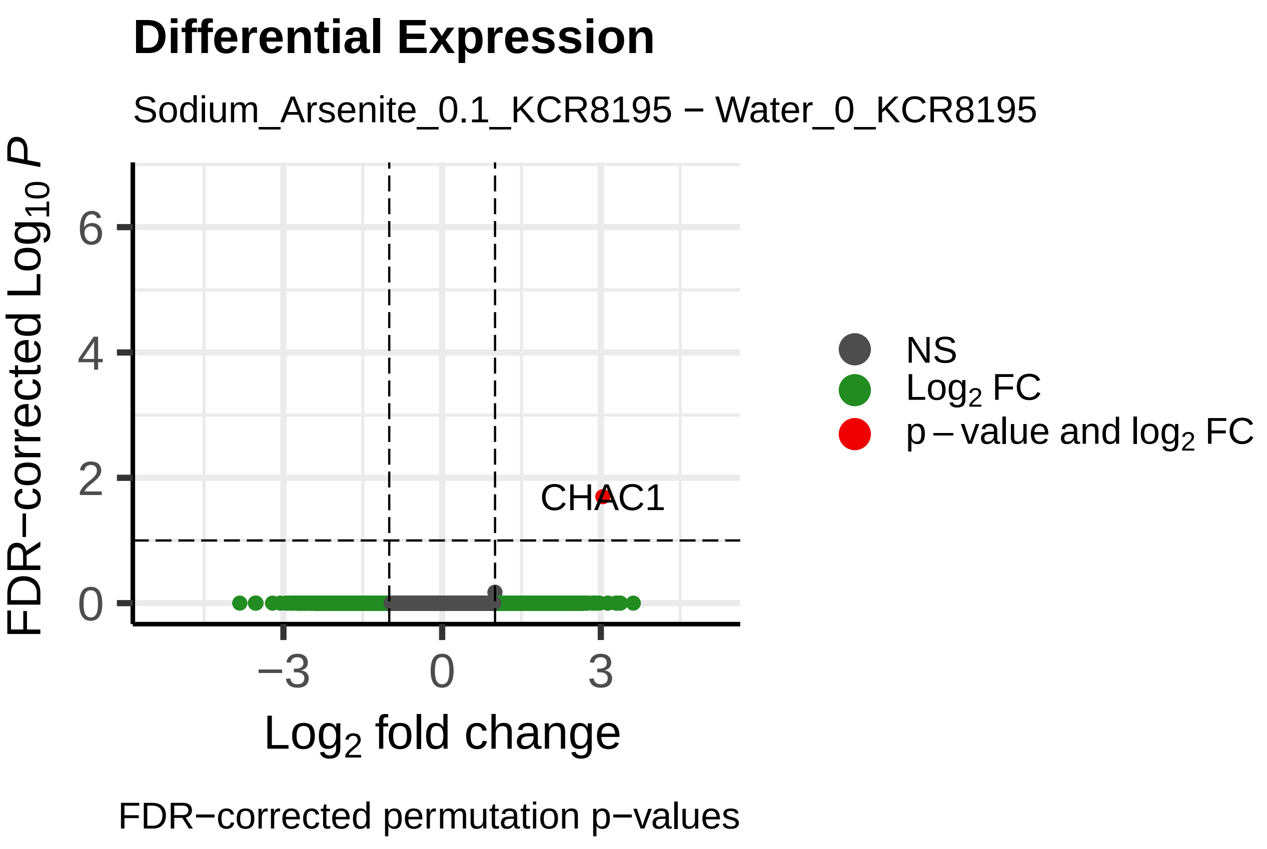

Differential Expression
Sodium_Arsenite_0.1_KCR8195 − Water_0_KCR8195
FDR−corrected Log10 P
Log2 fold change
CHAC1
NS
Log2 FC
p − value and log2 FC
FDR−corrected permutation p−values


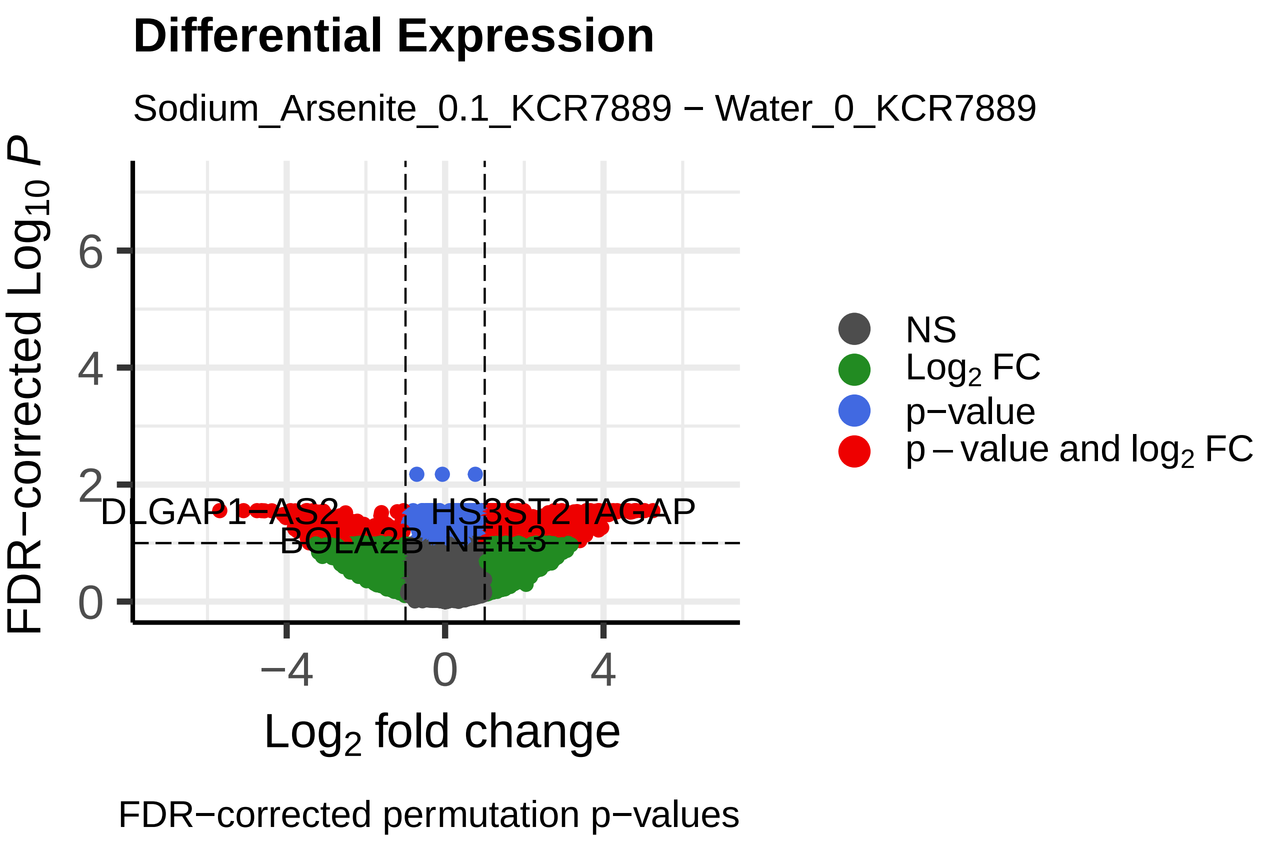

Differential Expression
Sodium_Arsenite_0.1_KCR7889 − Water_0_KCR7889
FDR−corrected Log10 P
Log2 fold change
DLGAP1−AS2
BOLA2B
NEIL3
HS3ST2
TAGAP
NS
Log2 FC
p−value
p − value and log2 FC
FDR−corrected permutation p−values


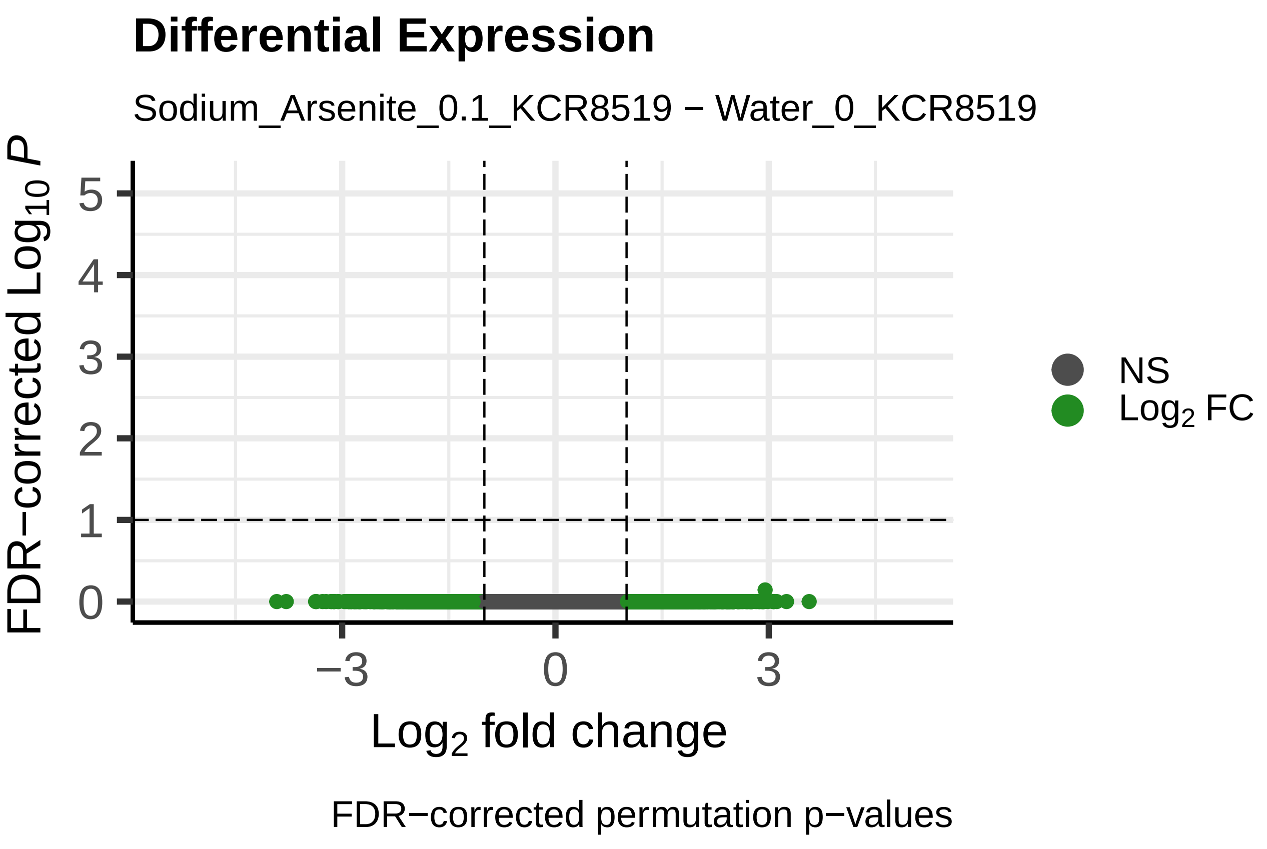

Differential Expression
Sodium_Arsenite_0.1_KCR8519 − Water_0_KCR8519
FDR−corrected Log10 P
Log2 fold change
NS
Log2 FC
FDR−corrected permutation p−values


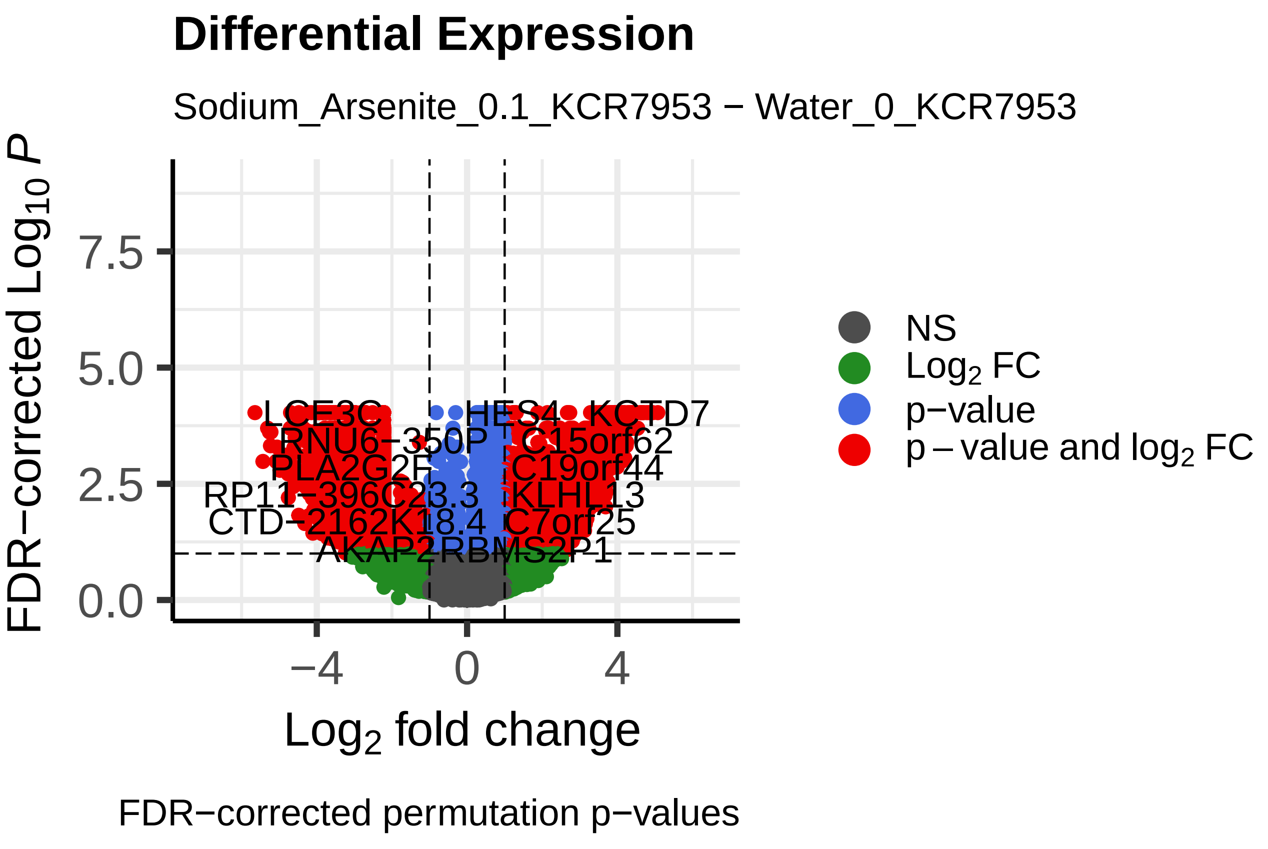

Differential Expression
Sodium_Arsenite_0.1_KCR7953 − Water_0_KCR7953
FDR−corrected Log10 P
Log2 fold change
LCE3C
RNU6−350P
PLA2G2F
RP11−396C23.3
CTD−2162K18.4
AKAP2
HES4
KCTD7
C15orf62
C19orf44
KLHL13
C7orf25
RBMS2P1
NS
Log2 FC
p−value
p − value and log2 FC
FDR−corrected permutation p−values


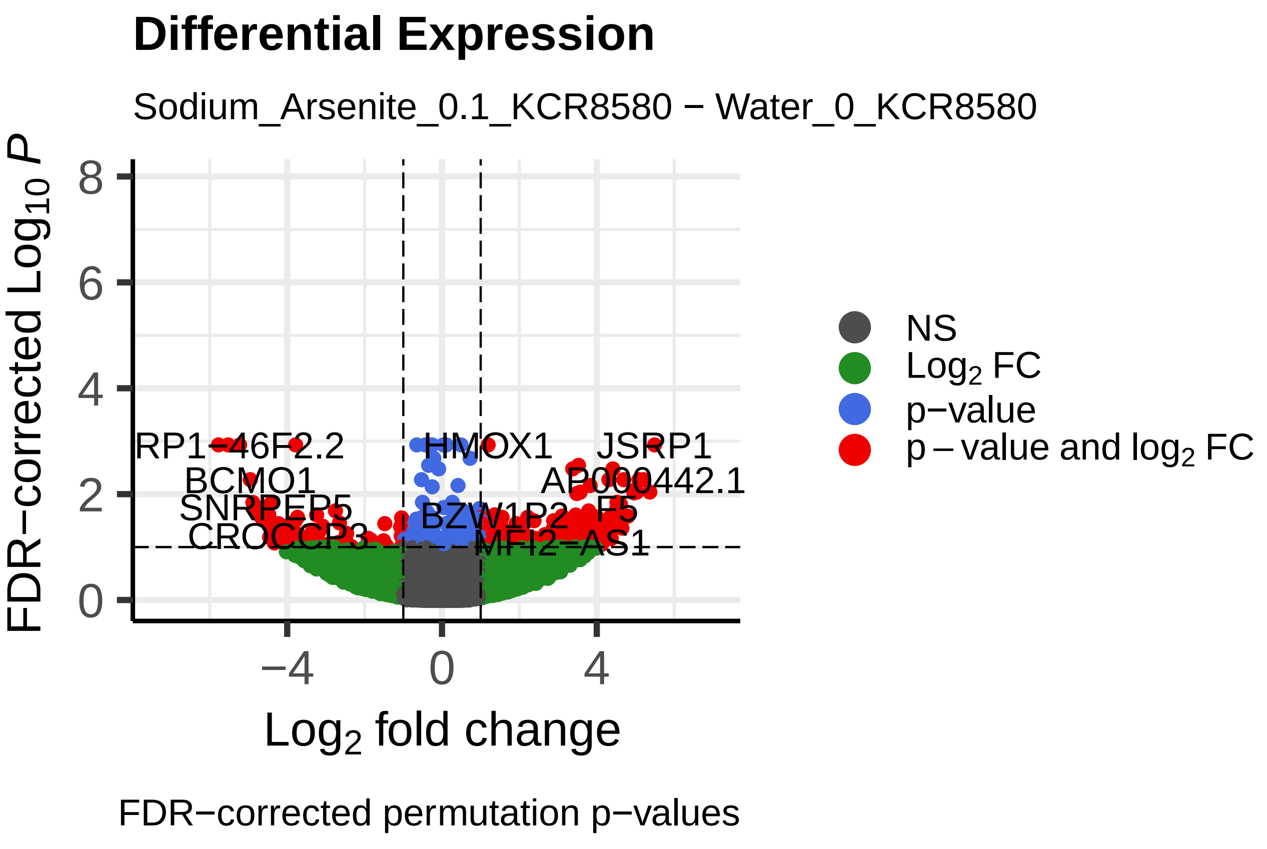

Differential Expression
Sodium_Arsenite_0.1_KCR8580 − Water_0_KCR8580
FDR−corrected Log10 P
Log2 fold change
RP1−46F2.2
BCMO1
HMOX1
JSRP1
AP000442.1
MFI2−AS1
NS
Log2 FC
p−value
p − value and log2 FC
FDR−corrected permutation p−values

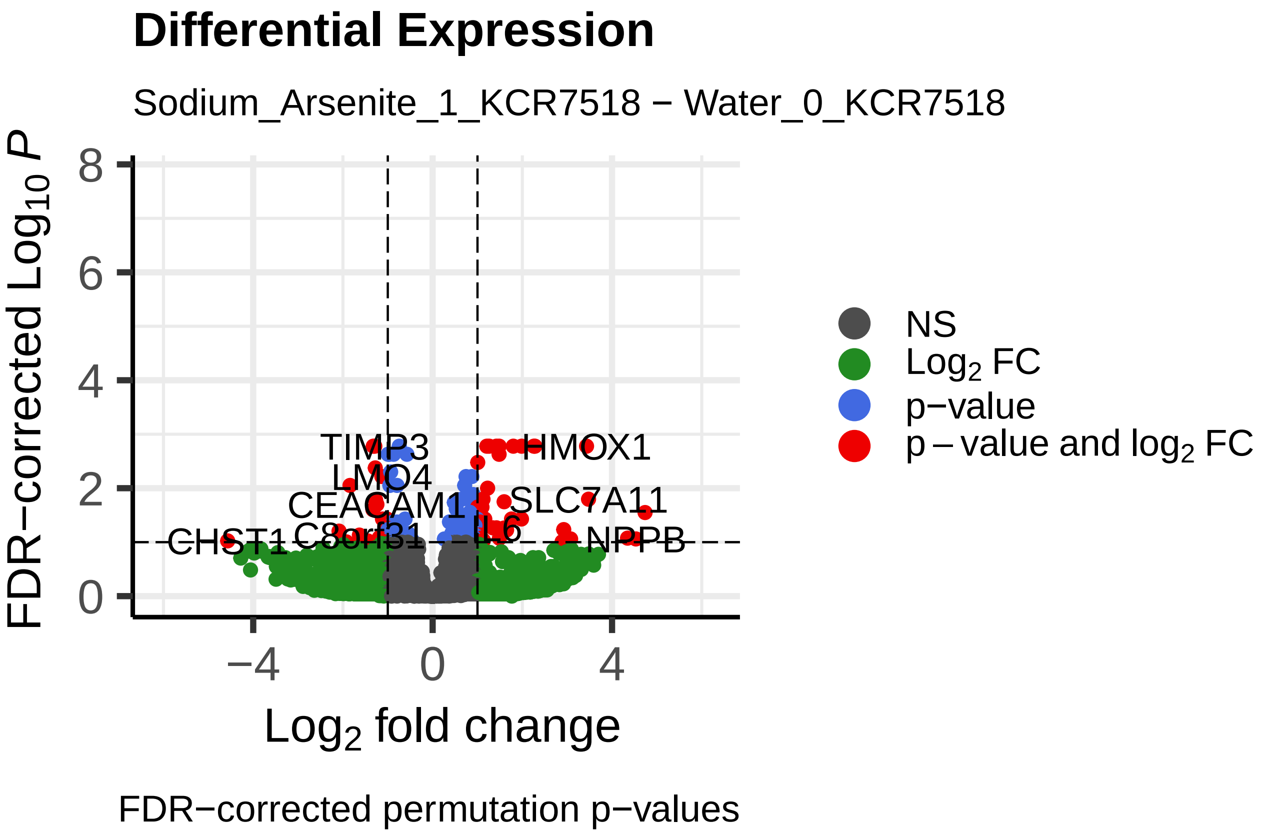
Differential Expression
Sodium_Arsenite_1_KCR7518 − Water_0_KCR7518
FDR−corrected Log10 P
Log2 fold change
TIMP3
LMO4
CEACAM1
CHST1
C8orf31
IL6
HMOX1
SLC7A11
NPPB
NS
Log2 FC
p−value
p − value and log2 FC
FDR−corrected permutation p−values

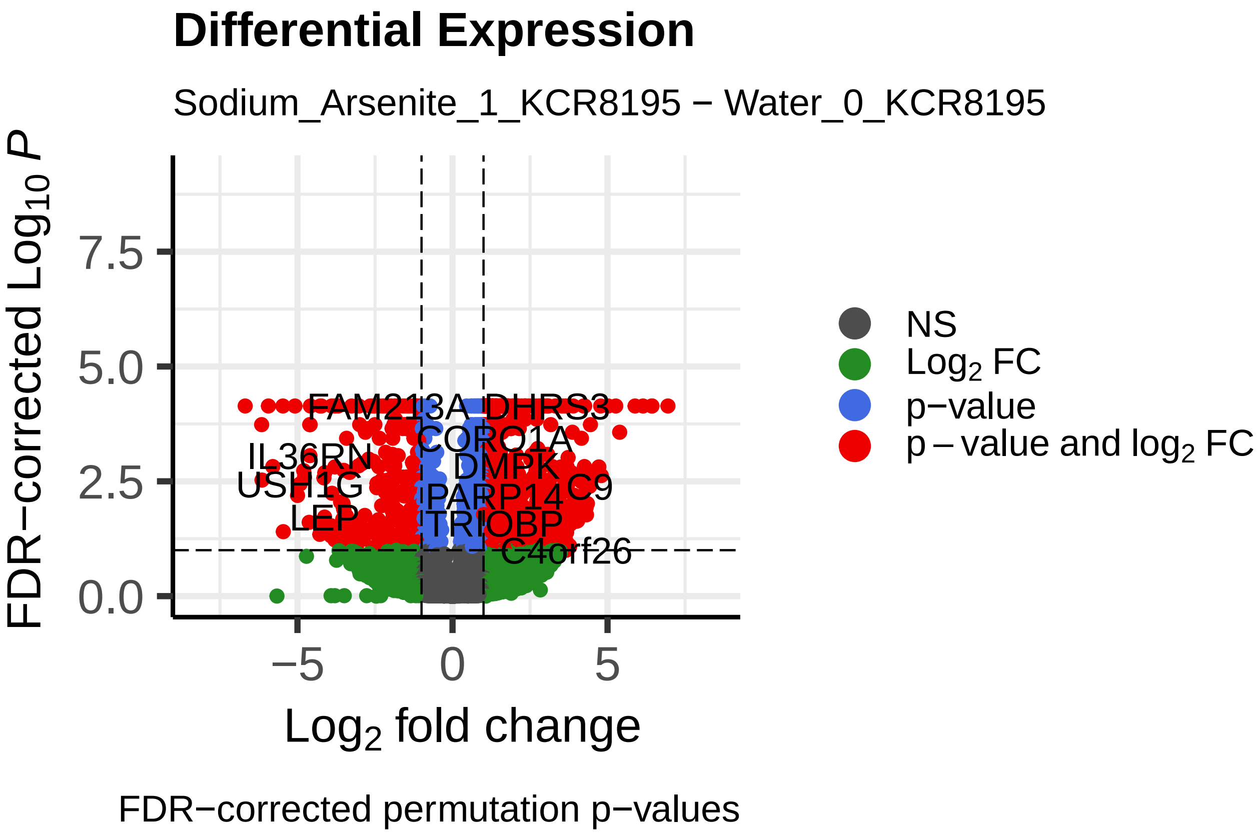
Differential Expression
Sodium_Arsenite_1_KCR8195 − Water_0_KCR8195
FDR−corrected Log10 P
Log2 fold change
FAM213A
DHRS3
CORO1A
IL36RN
DMPK
USH1G
PARP14
C9
LEP
TRIOBP
C4orf26
NS
Log2 FC
p−value
p − value and log2 FC
FDR−corrected permutation p−values

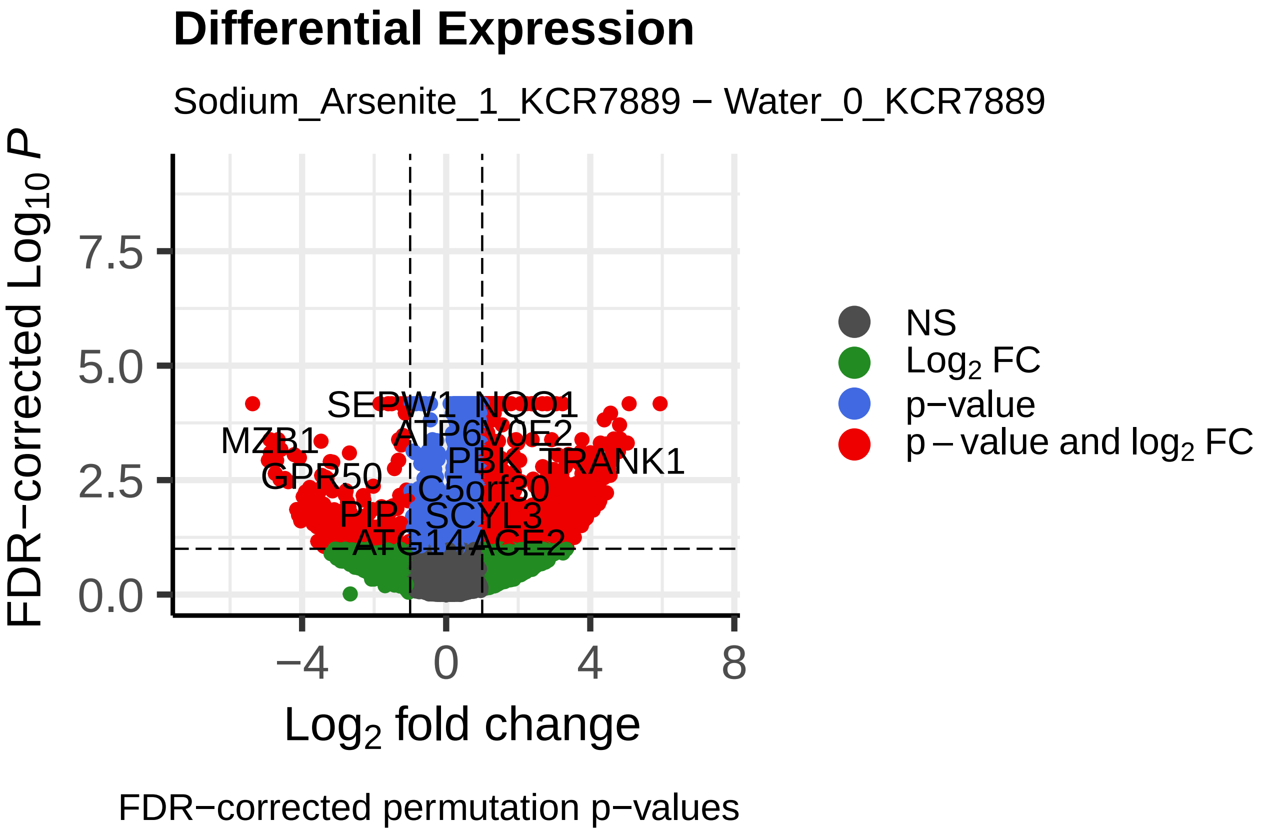
Differential Expression
Sodium_Arsenite_1_KCR7889 − Water_0_KCR7889
FDR−corrected Log10 P
Log2 fold change
SEPW1
NQO1
MZB1
ATP6V0E2
PBK
TRANK1
GPR50
C5orf30
PIP
SCYL3
ATG14
ACE2
NS
Log2 FC
p−value
p − value and log2 FC
FDR−corrected permutation p−values

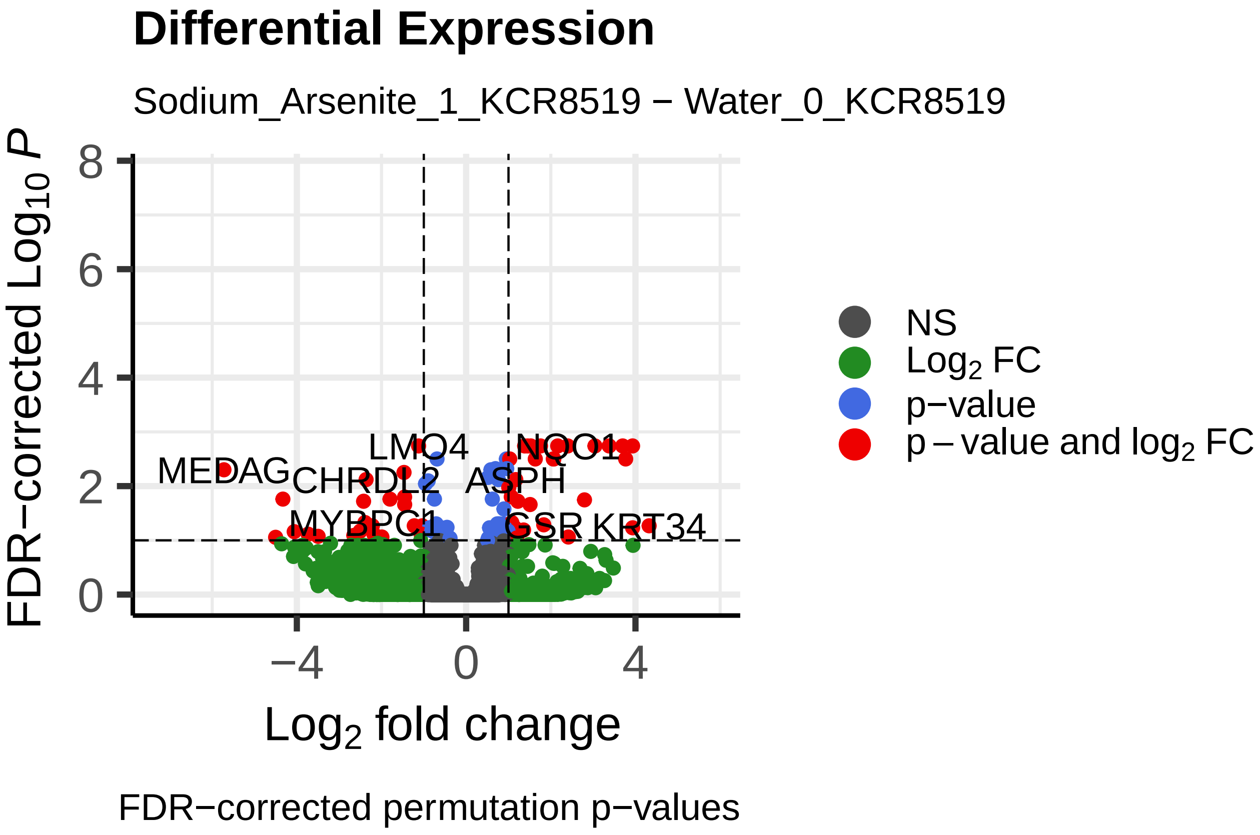
Differential Expression
Sodium_Arsenite_1_KCR8519 − Water_0_KCR8519
FDR−corrected Log10 P
Log2 fold change
LMO4
NQO1
MEDAG
CHRDL2
ASPH
MYBPC1
GSR
KRT34
NS
Log2 FC
p−value
p − value and log2 FC
FDR−corrected permutation p−values

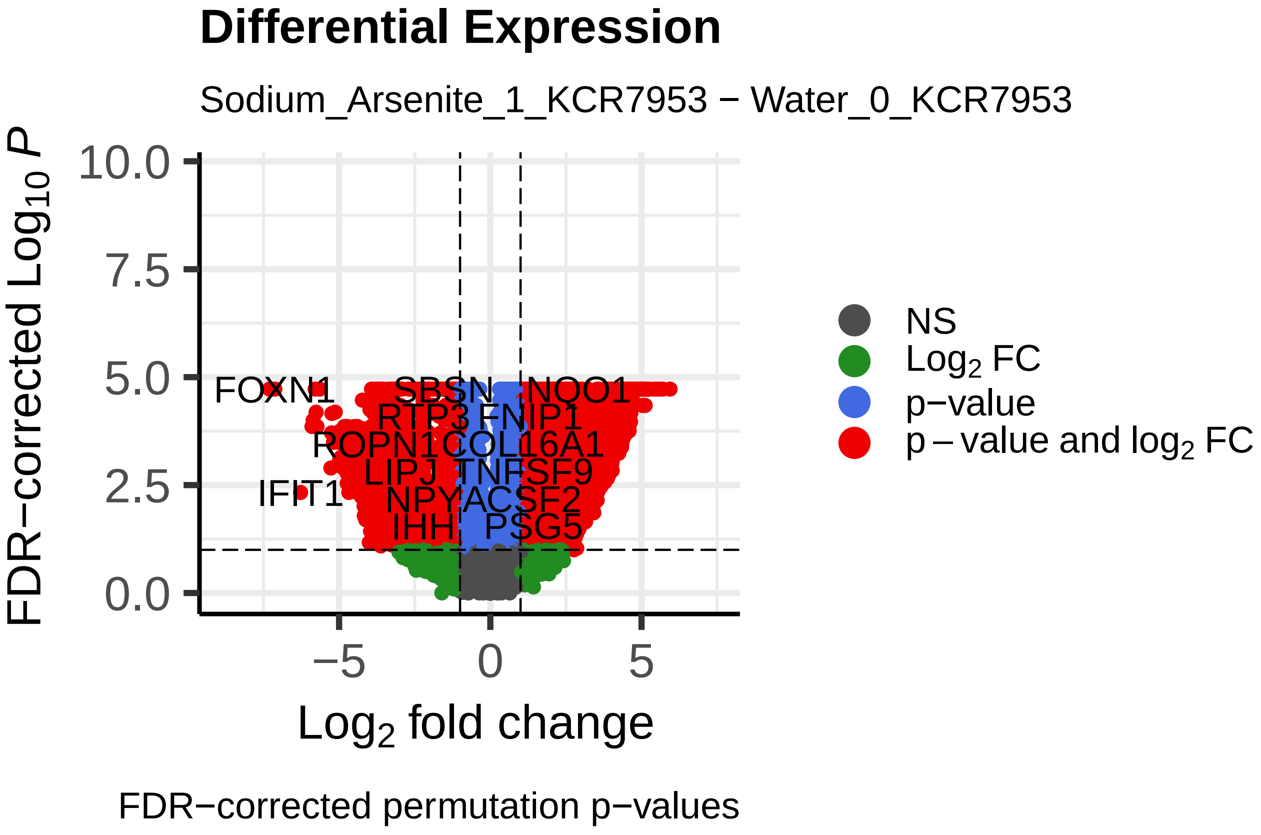
Differential Expression
Sodium_Arsenite_1_KCR7953 − Water_0_KCR7953
FDR−corrected Log10 P
Log2 fold change
FOXN1
SBSN
NQO1
RTP3
FNIP1
ROPN1
COL16A1
LIPJ
TNFSF9
IFIT1
NPY
ACSF2
IHH
PSG5
NS
Log2 FC
p−value
p − value and log2 FC
FDR−corrected permutation p−values

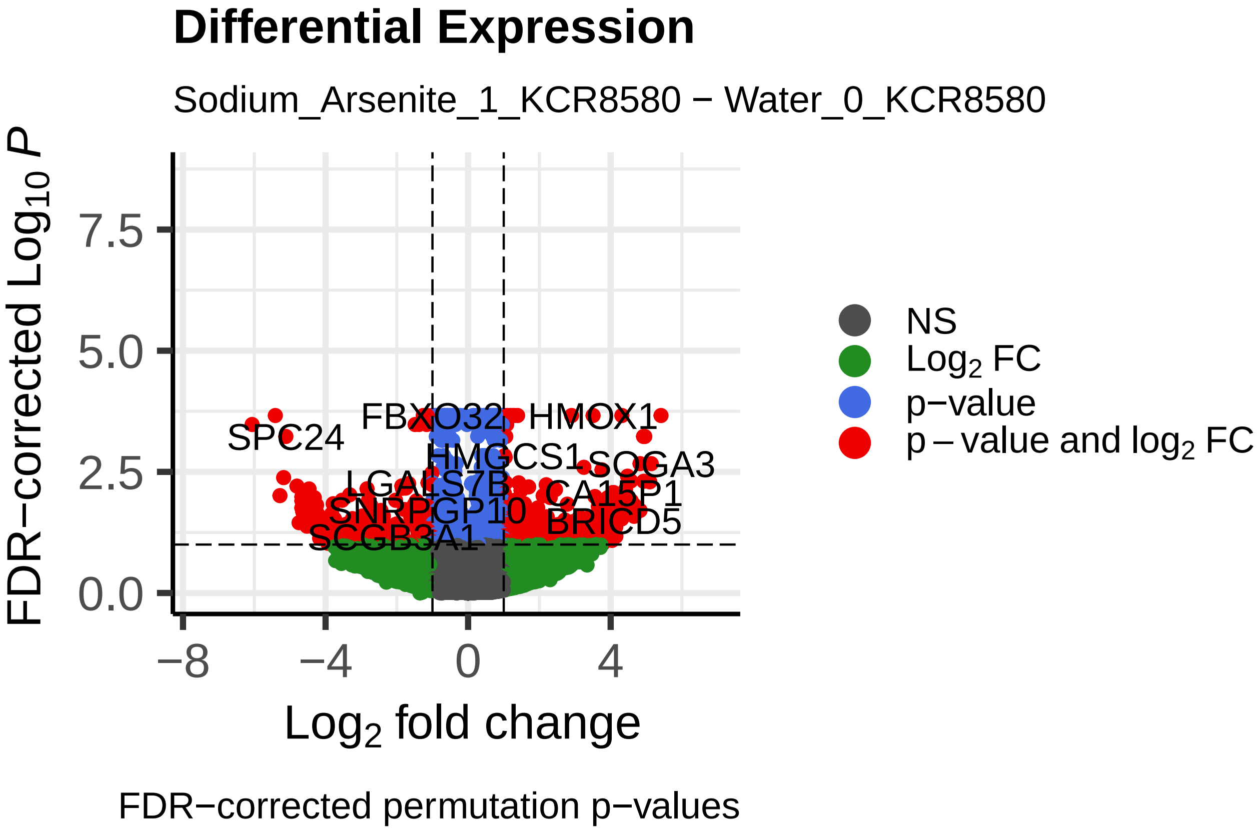
Differential Expression
Sodium_Arsenite_1_KCR8580 − Water_0_KCR8580
FDR−corrected Log10 P
Log2 fold change
FBXO32
HMOX1
SPC24
HMGCS1
SOGA3
LGALS7B
CA15P1
SNRPGP10
BRICD5
SCGB3A1
NS
Log2 FC
p−value
p − value and log2 FC
FDR−corrected permutation p−values

## Differential Expression

Sodium_Arsenite_10_KCR7518 − Water_0_KCR7518

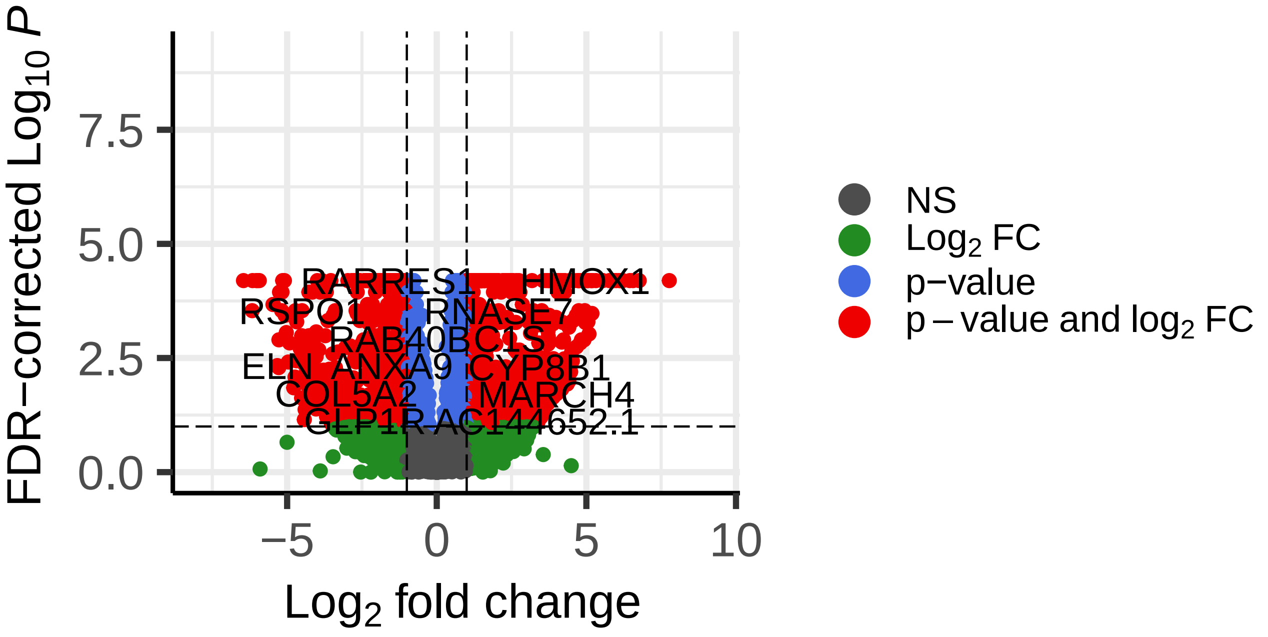


FDR−corrected permutation p−values

## Differential Expression

Sodium_Arsenite_10_KCR8195 − Water_0_KCR8195

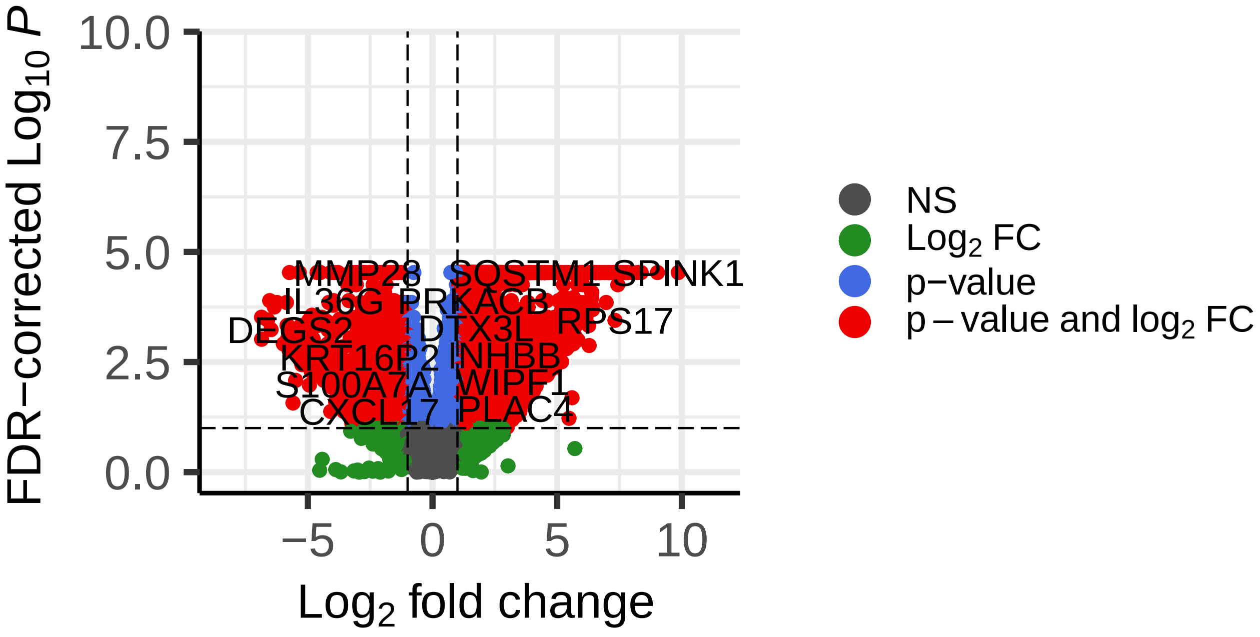


FDR−corrected permutation p−values

## Differential Expression

Sodium_Arsenite_10_KCR7889 − Water_0_KCR7889

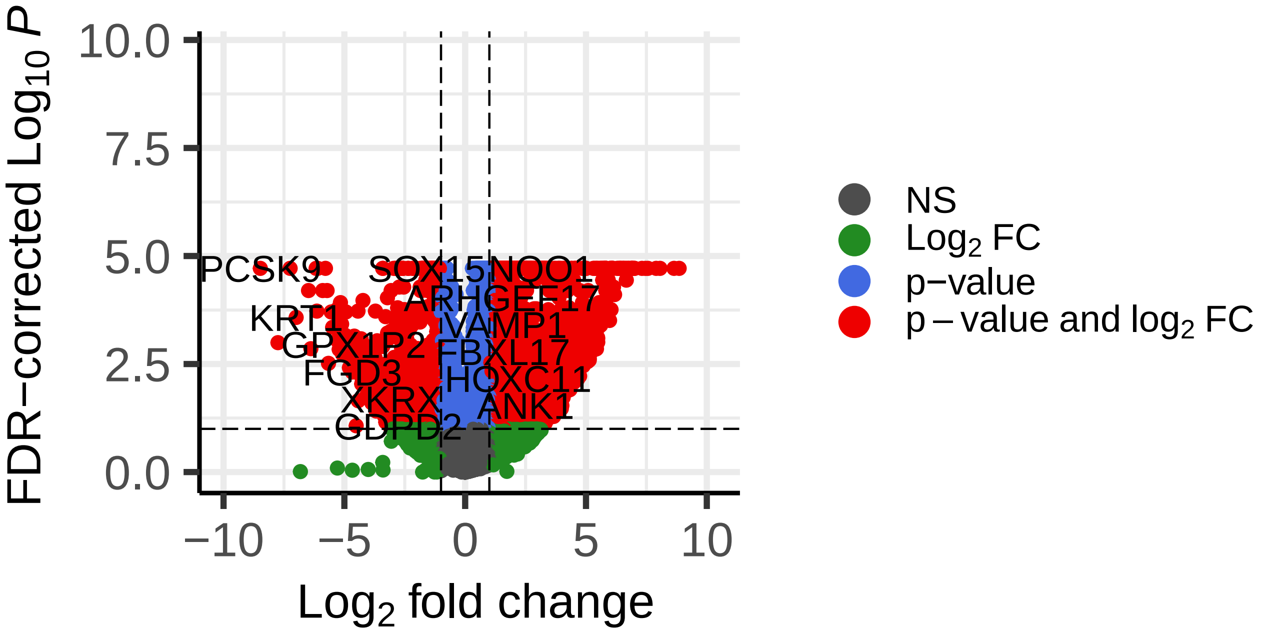


FDR−corrected permutation p−values

## Differential Expression

Sodium_Arsenite_10_KCR8519 − Water_0_KCR8519

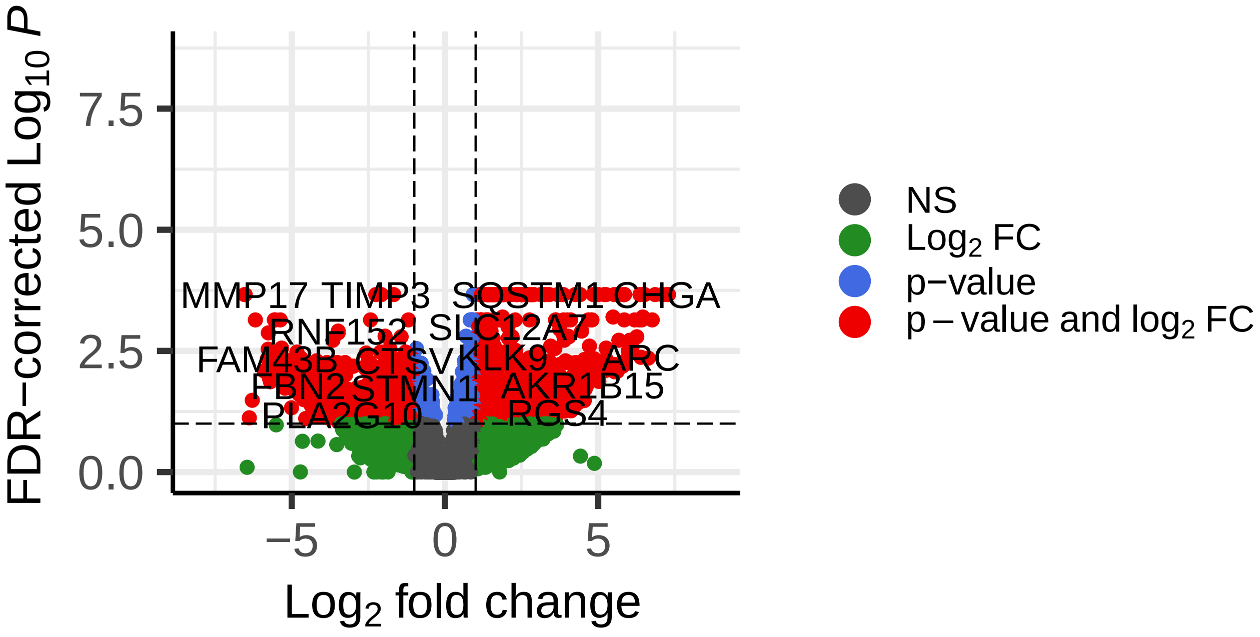


FDR−corrected permutation p−values

## Differential Expression

Sodium_Arsenite_10_KCR7953 − Water_0_KCR7953

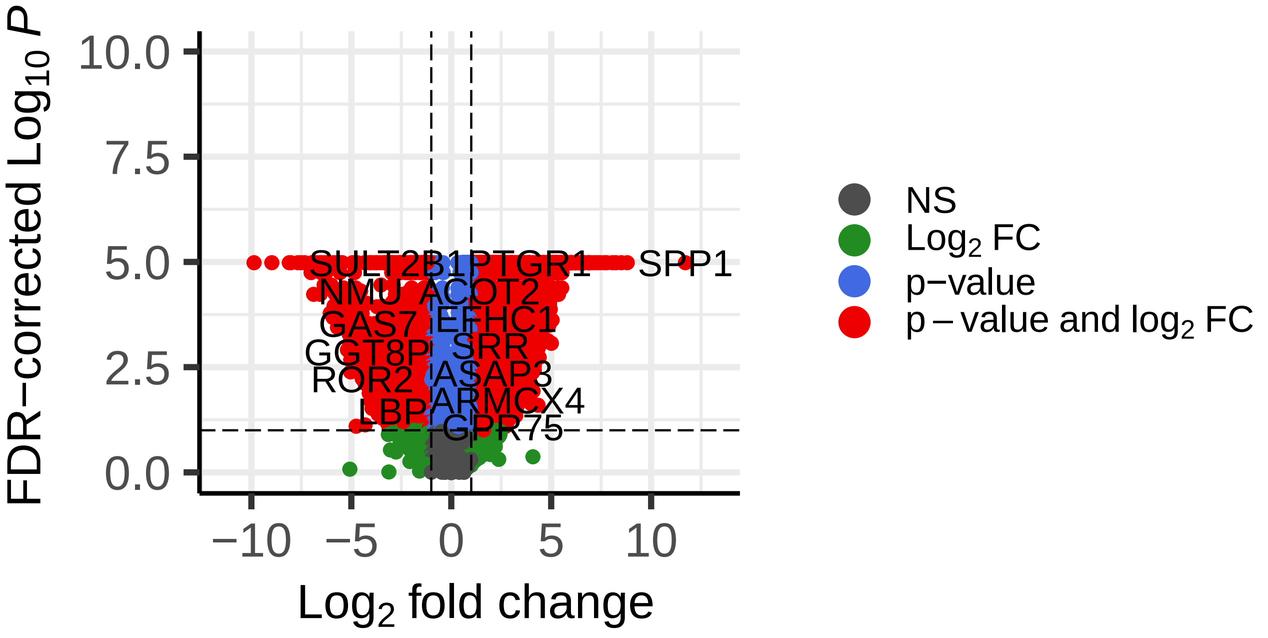


FDR−corrected permutation p−values

## Differential Expression

Sodium_Arsenite_10_KCR8580 − Water_0_KCR8580

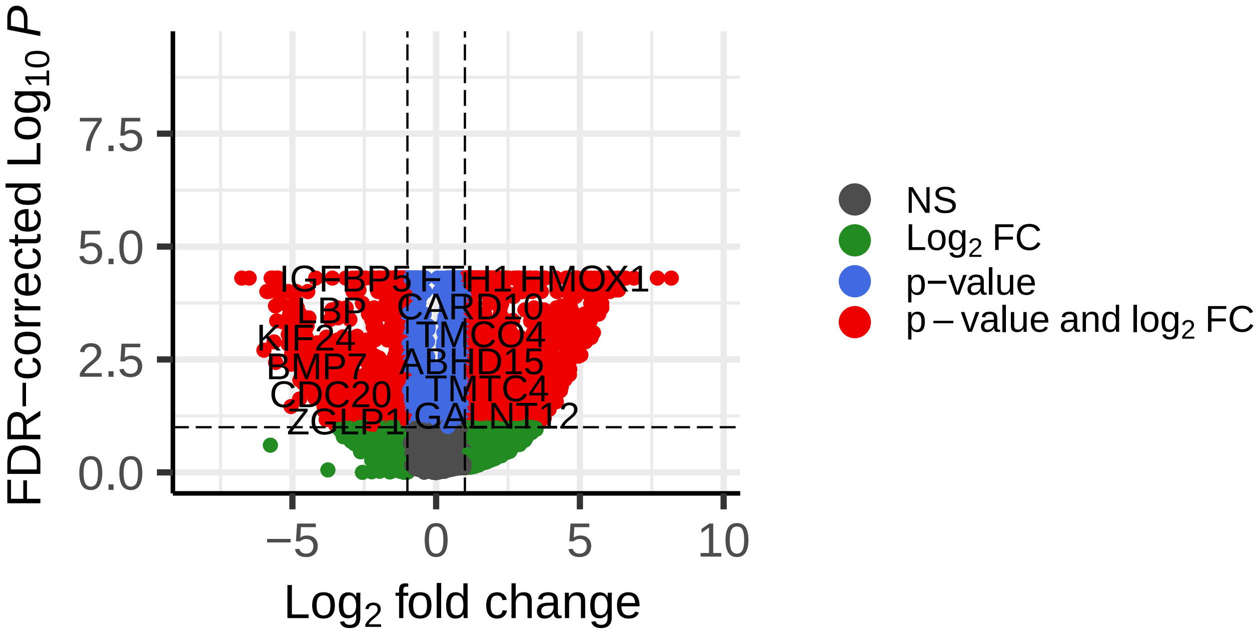


FDR−corrected permutation p−values

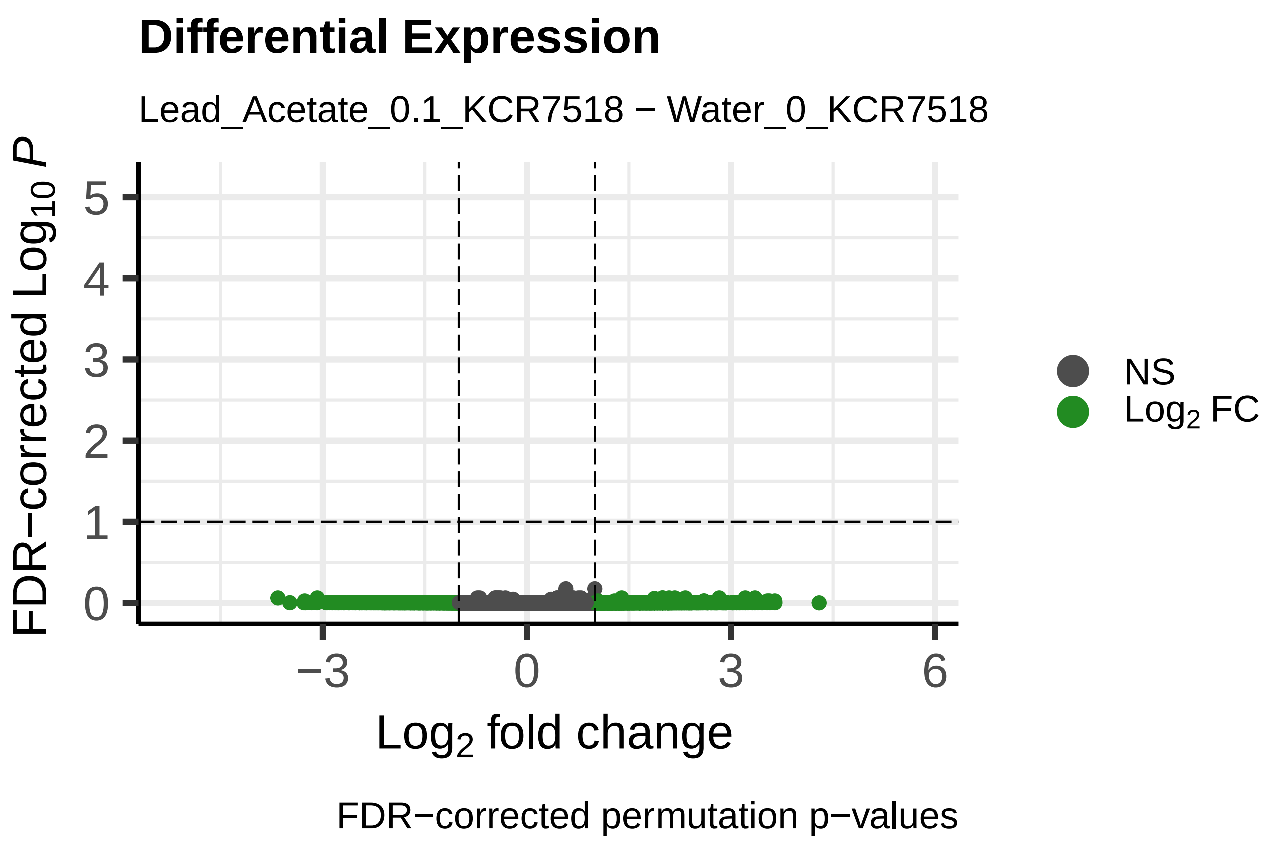
Differential Expression
Lead_Acetate_0.1_KCR7518 − Water_0_KCR7518
FDR−corrected $Log_{10}$ P
$Log_2$ fold change
NS
$Log_2$ FC
FDR−corrected permutation p−values

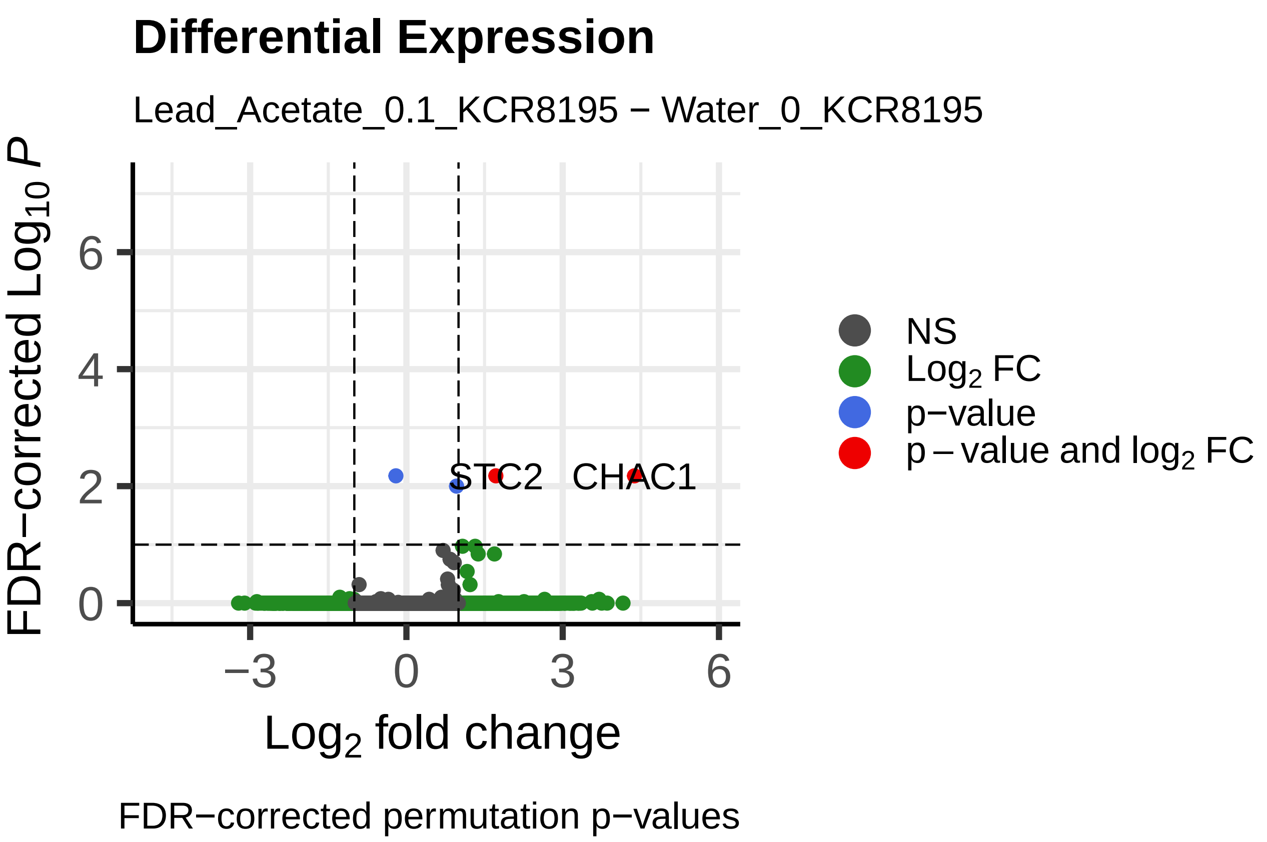
Differential Expression
Lead_Acetate_0.1_KCR8195 − Water_0_KCR8195
FDR−corrected $Log_{10}$ P
$Log_2$ fold change
STC2
CHAC1
NS
$Log_2$ FC
p−value
p − value and $log_2$ FC
FDR−corrected permutation p−values

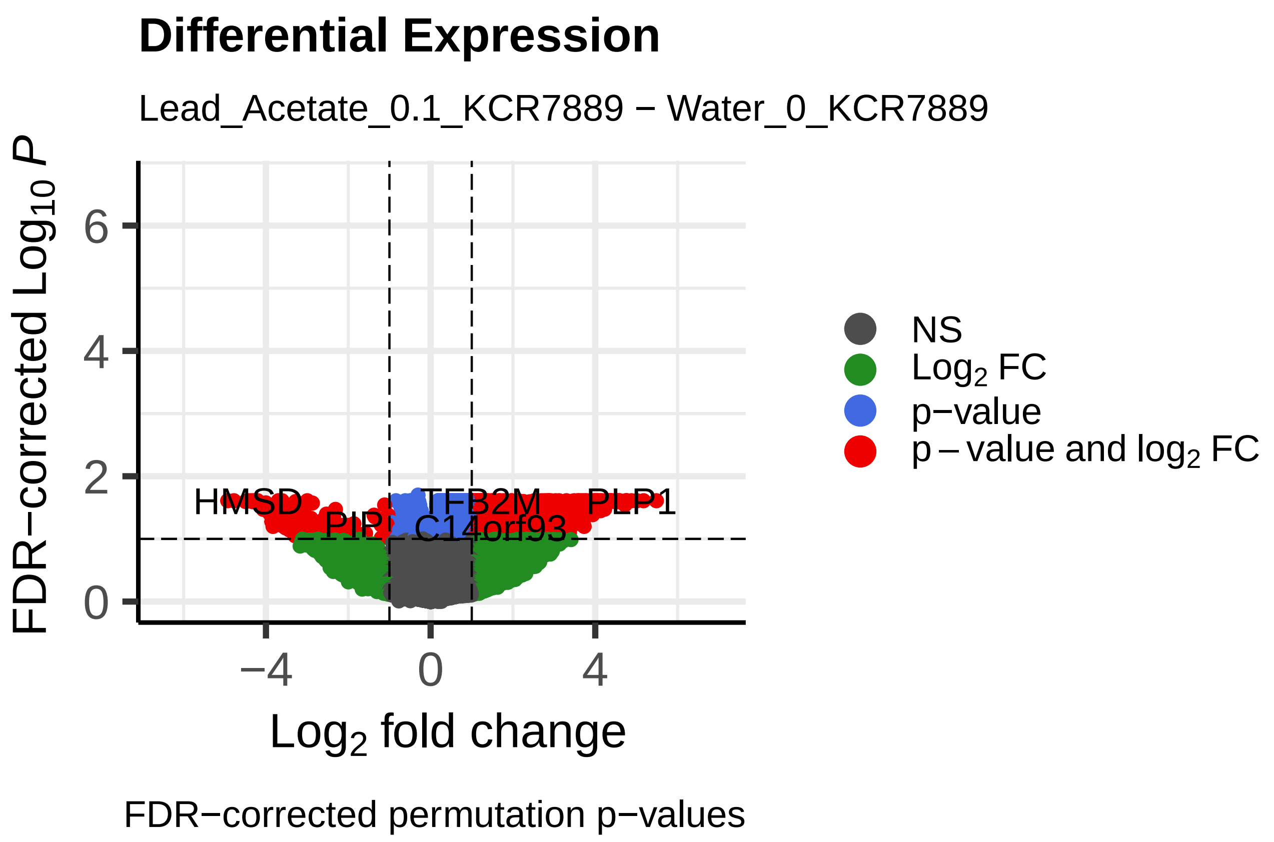
Differential Expression
Lead_Acetate_0.1_KCR7889 − Water_0_KCR7889
FDR−corrected $Log_{10}$ P
$Log_2$ fold change
HMSD
TFB2M
PLP1
PIP
C14orf93
NS
$Log_2$ FC
p−value
p − value and $log_2$ FC
FDR−corrected permutation p−values

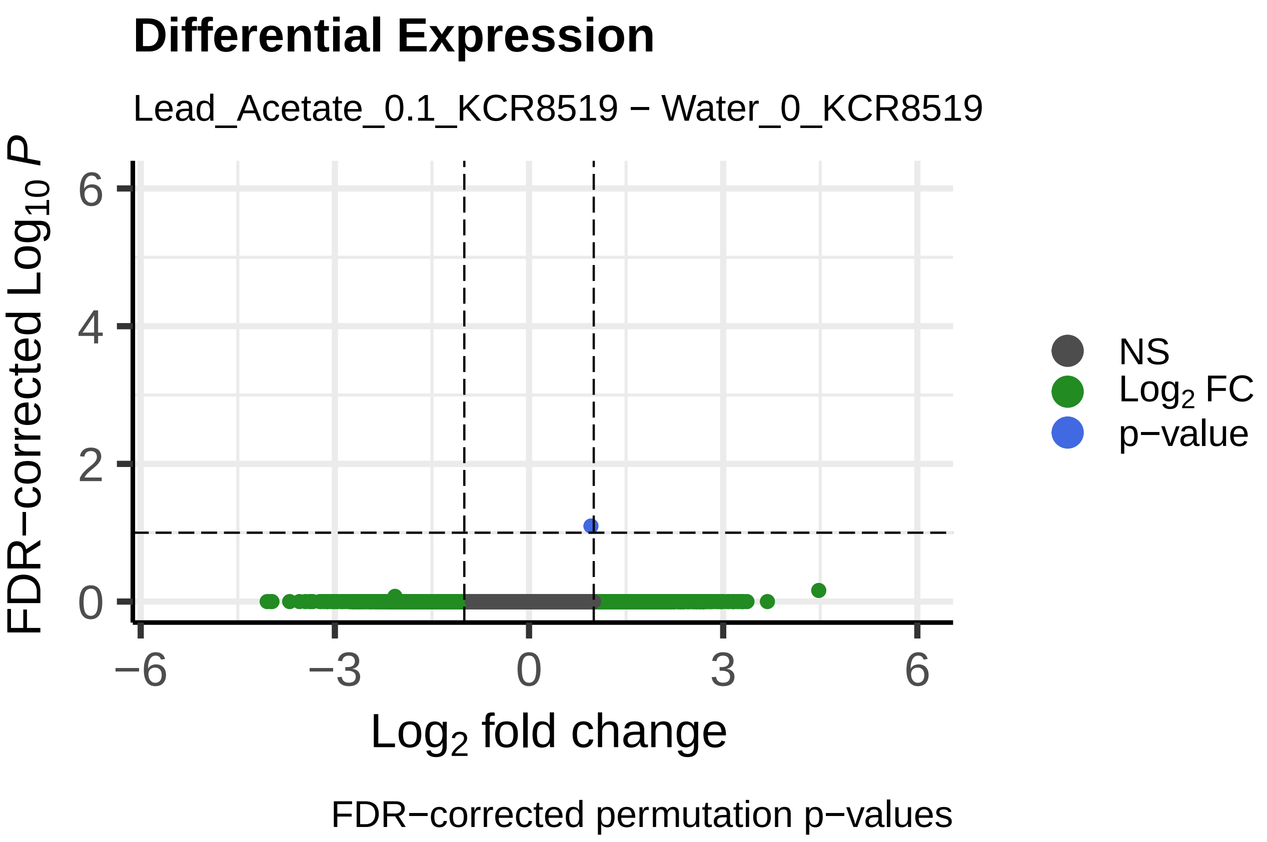
Differential Expression
Lead_Acetate_0.1_KCR8519 − Water_0_KCR8519
FDR−corrected $Log_{10}$ P
$Log_2$ fold change
NS
$Log_2$ FC
p−value
FDR−corrected permutation p−values

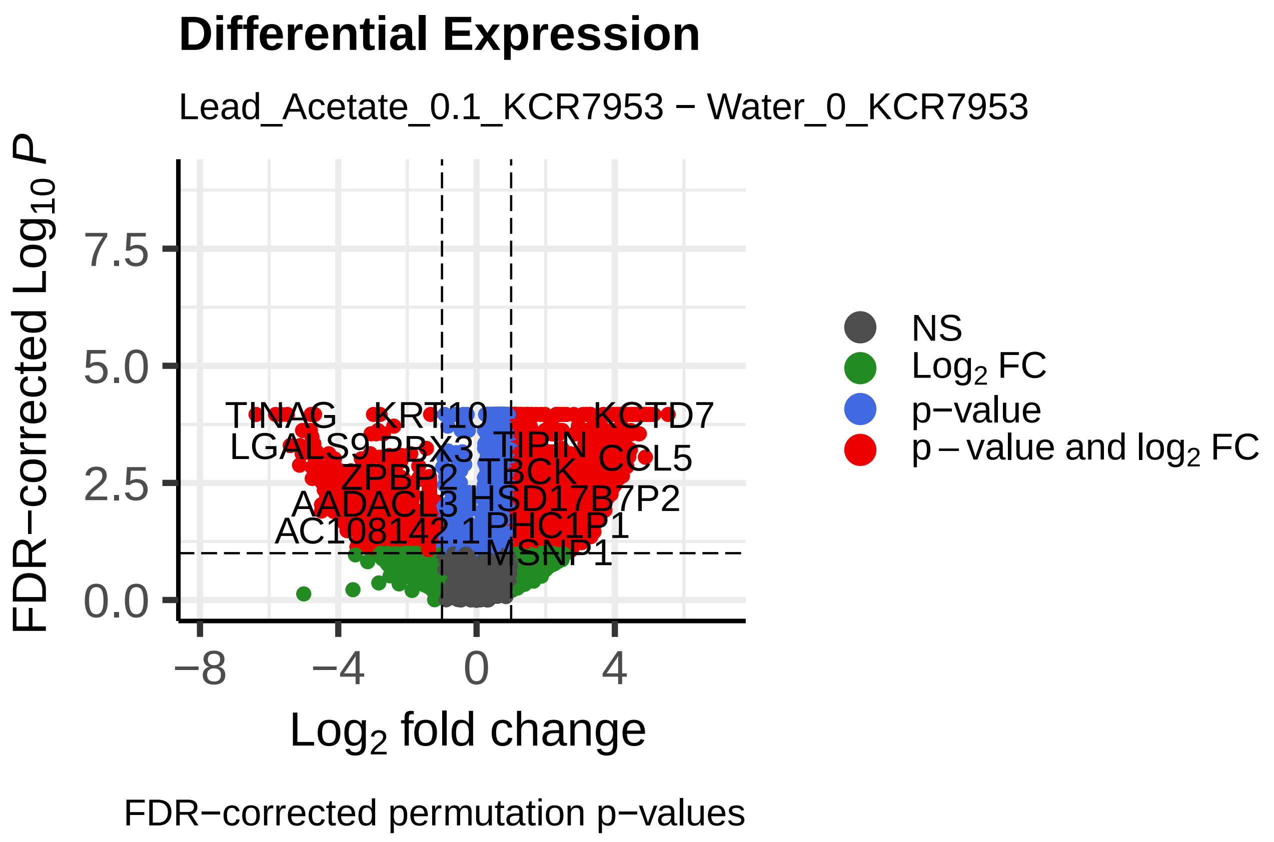
Differential Expression
Lead_Acetate_0.1_KCR7953 − Water_0_KCR7953
FDR−corrected $Log_{10}$ P
$Log_2$ fold change
TINAG
KRT10
KCTD7
LGALS9
PRX3
TIPIN
COL5
ZPBP2
TBCK
AADACL3
HSD17B7P2
AC108142.1
PHC1P1
MSNP1
NS
$Log_2$ FC
p−value
p − value and $log_2$ FC
FDR−corrected permutation p−values

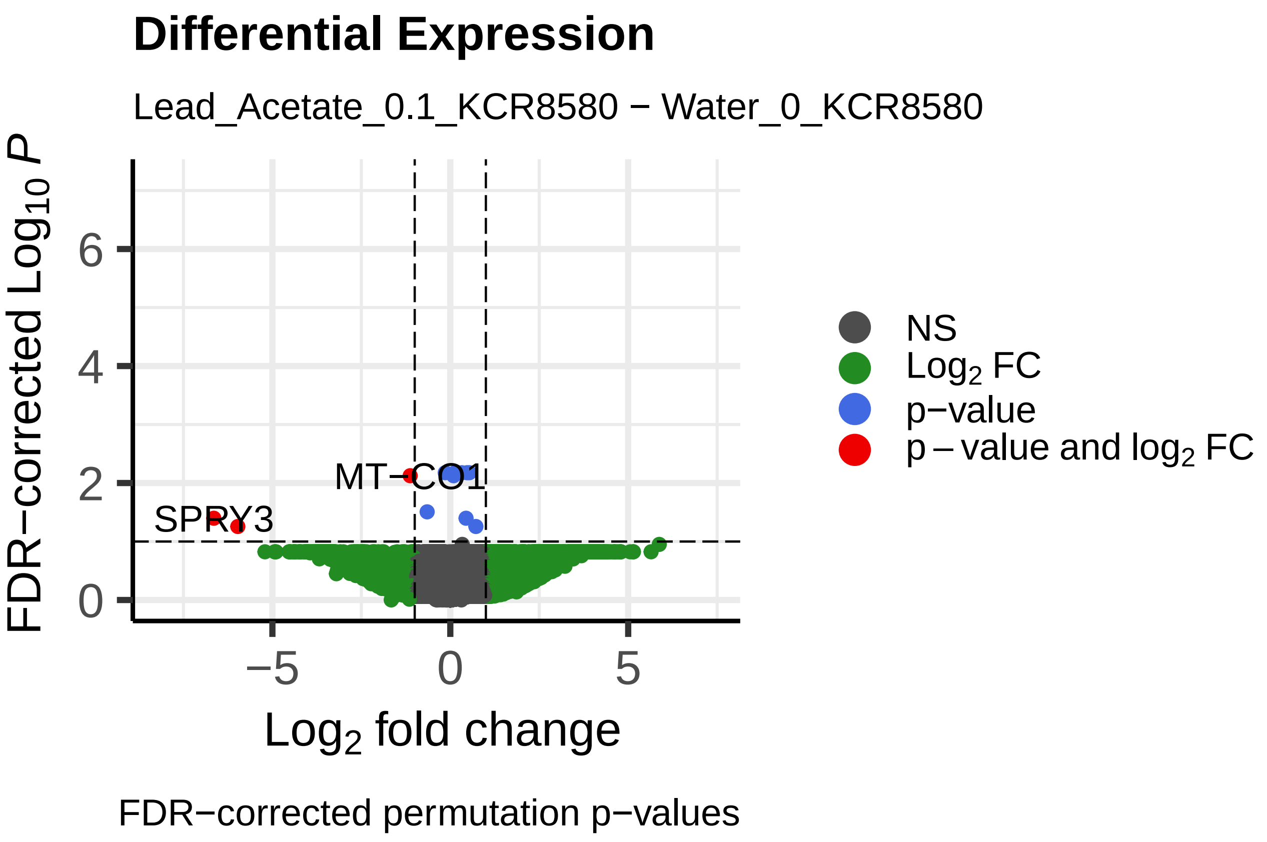
Differential Expression
Lead_Acetate_0.1_KCR8580 − Water_0_KCR8580
FDR−corrected $Log_{10}$ P
$Log_2$ fold change
MT−CO1
SPRY3
NS
$Log_2$ FC
p−value
p − value and $log_2$ FC
FDR−corrected permutation p−values

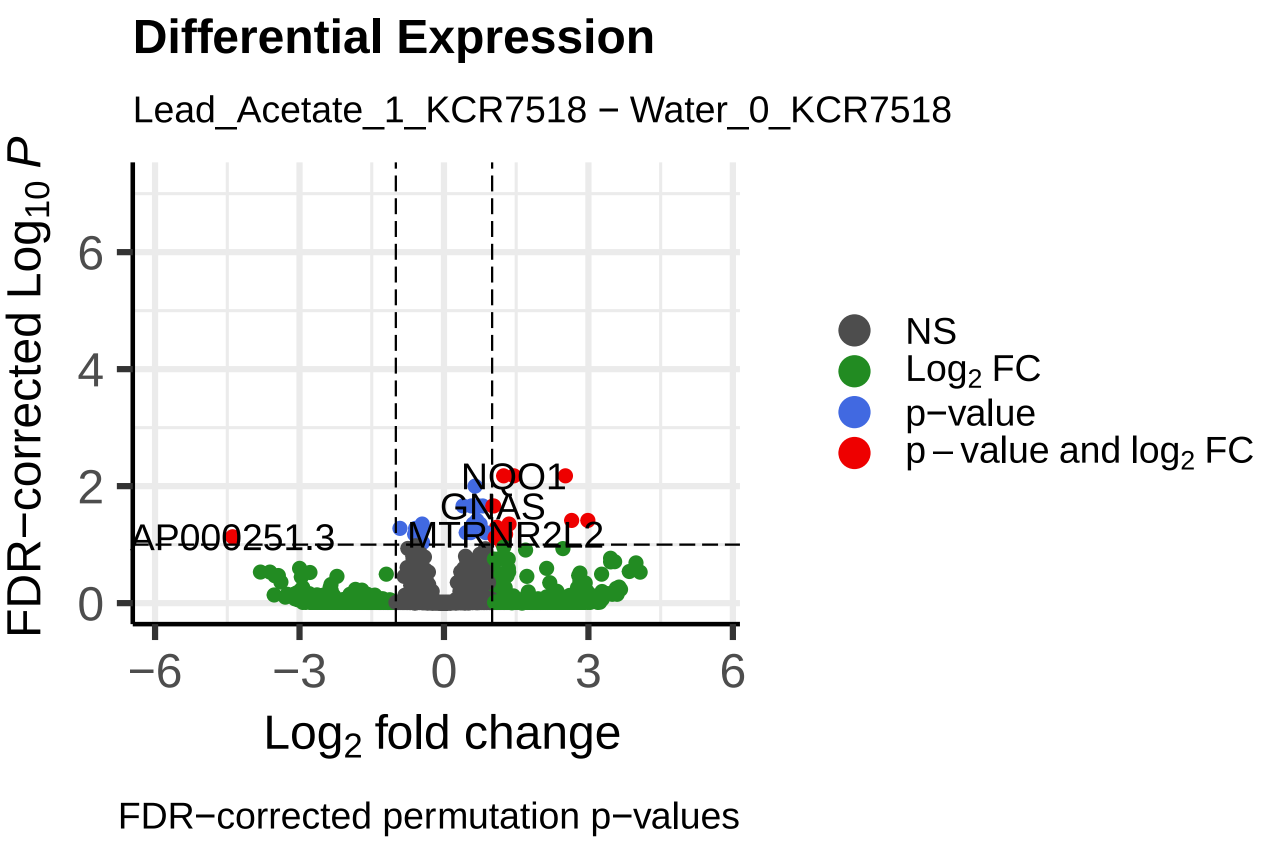
Differential Expression
Lead_Acetate_1_KCR7518 − Water_0_KCR7518
FDR−corrected $Log_{10}$ $P$
Log2 fold change
NQO1
GNAS
AP000251.3
MTRNR2L2
NS
Log2 FC
p−value
p − value and log2 FC
FDR−corrected permutation p−values

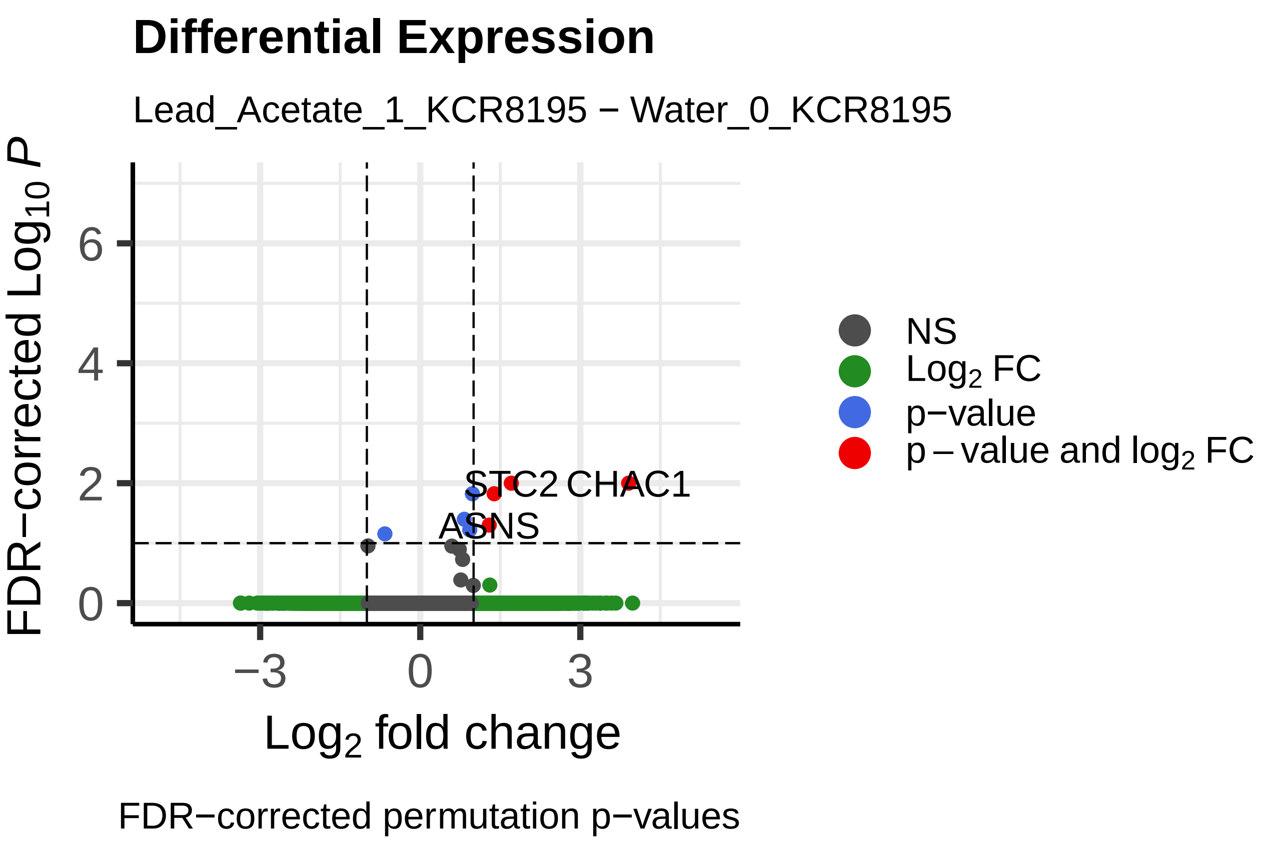
Differential Expression
Lead_Acetate_1_KCR8195 − Water_0_KCR8195
FDR−corrected $Log_{10}$ $P$
Log2 fold change
STC2
CHAC1
ASNS
NS
Log2 FC
p−value
p − value and log2 FC
FDR−corrected permutation p−values

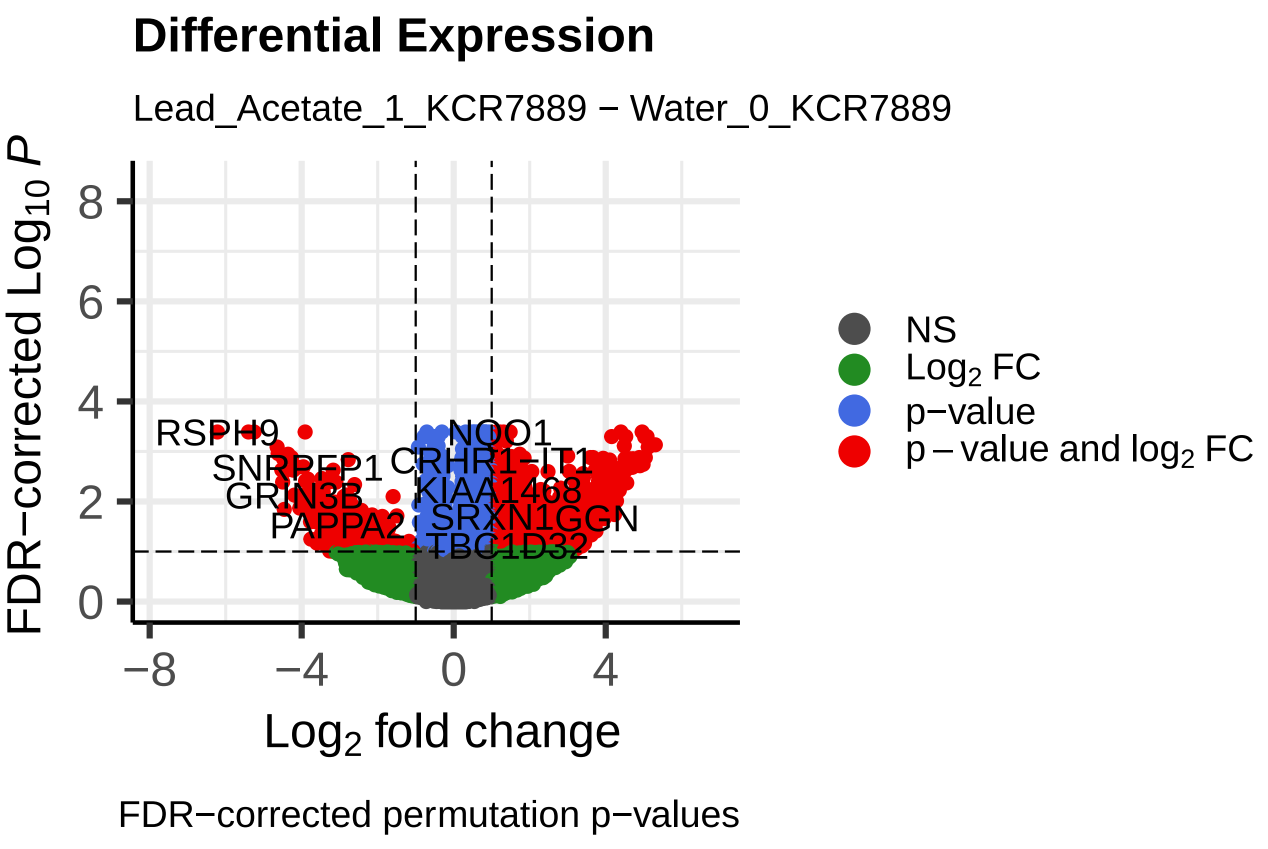
Differential Expression
Lead_Acetate_1_KCR7889 − Water_0_KCR7889
FDR−corrected $Log_{10}$ $P$
Log2 fold change
RSPH9
NQO1
SNRPEP1
CRHR1−IT1
GRIN3B
KIAA1468
PAPPA2
SRXN1
GGN
TBC1D32
NS
Log2 FC
p−value
p − value and log2 FC
FDR−corrected permutation p−values

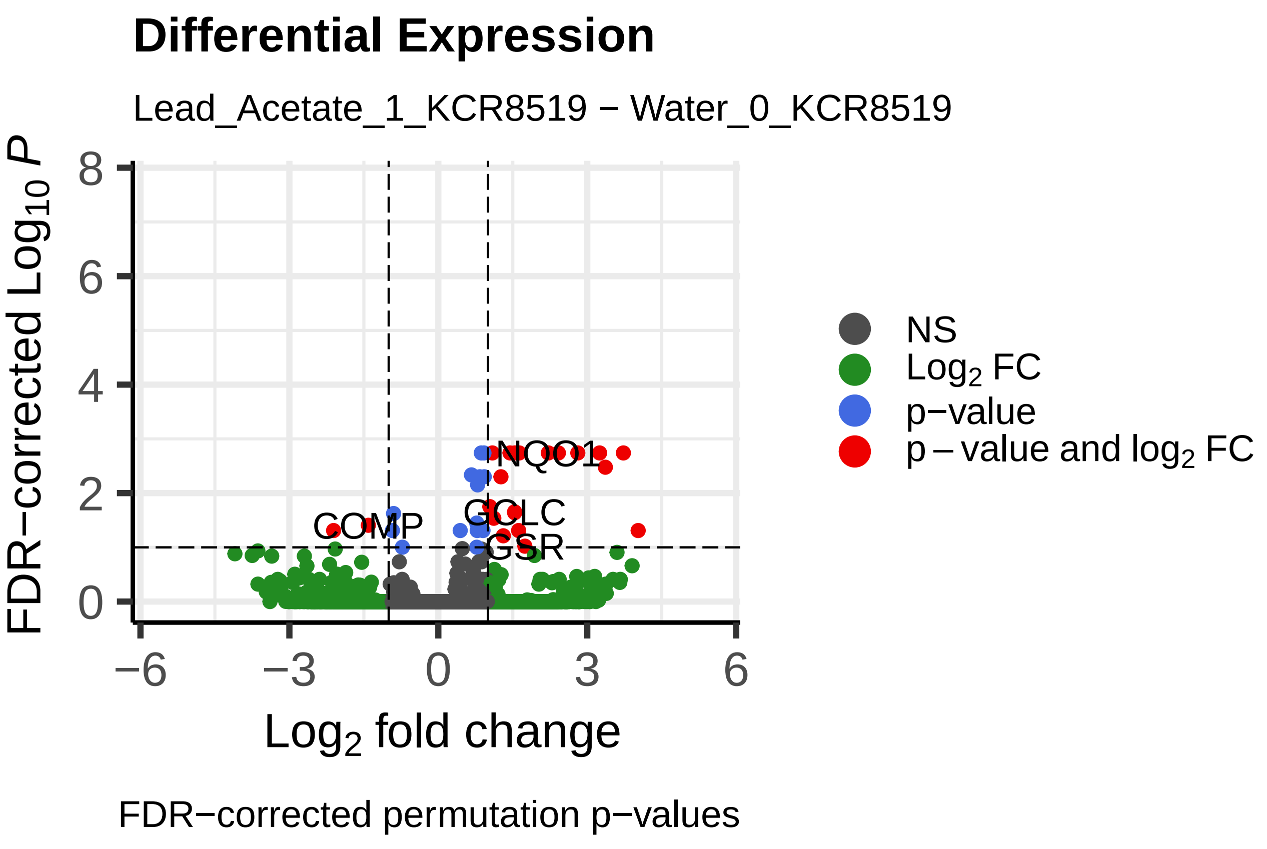
Differential Expression
Lead_Acetate_1_KCR8519 − Water_0_KCR8519
FDR−corrected $Log_{10}$ $P$
Log2 fold change
NQO1
COMP
GCLC
GSR
NS
Log2 FC
p−value
p − value and log2 FC
FDR−corrected permutation p−values

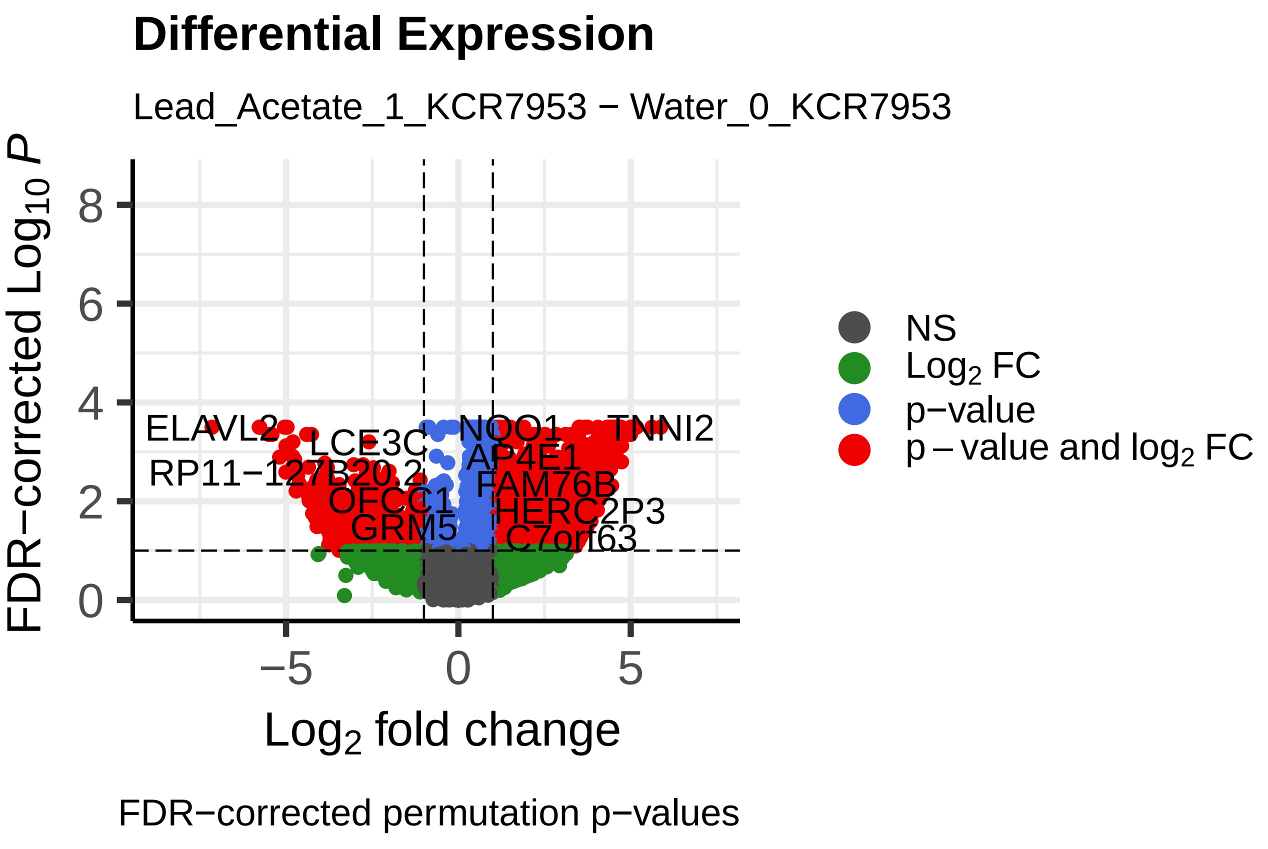
Differential Expression
Lead_Acetate_1_KCR7953 − Water_0_KCR7953
FDR−corrected $Log_{10}$ $P$
Log2 fold change
ELAVL2
NQO1
TNNI2
LCE3C
AP4E1
RP11−127B20.2
FAM76B
OFCC1
HERC2P3
GRM5
C7orf63
NS
Log2 FC
p−value
p − value and log2 FC
FDR−corrected permutation p−values

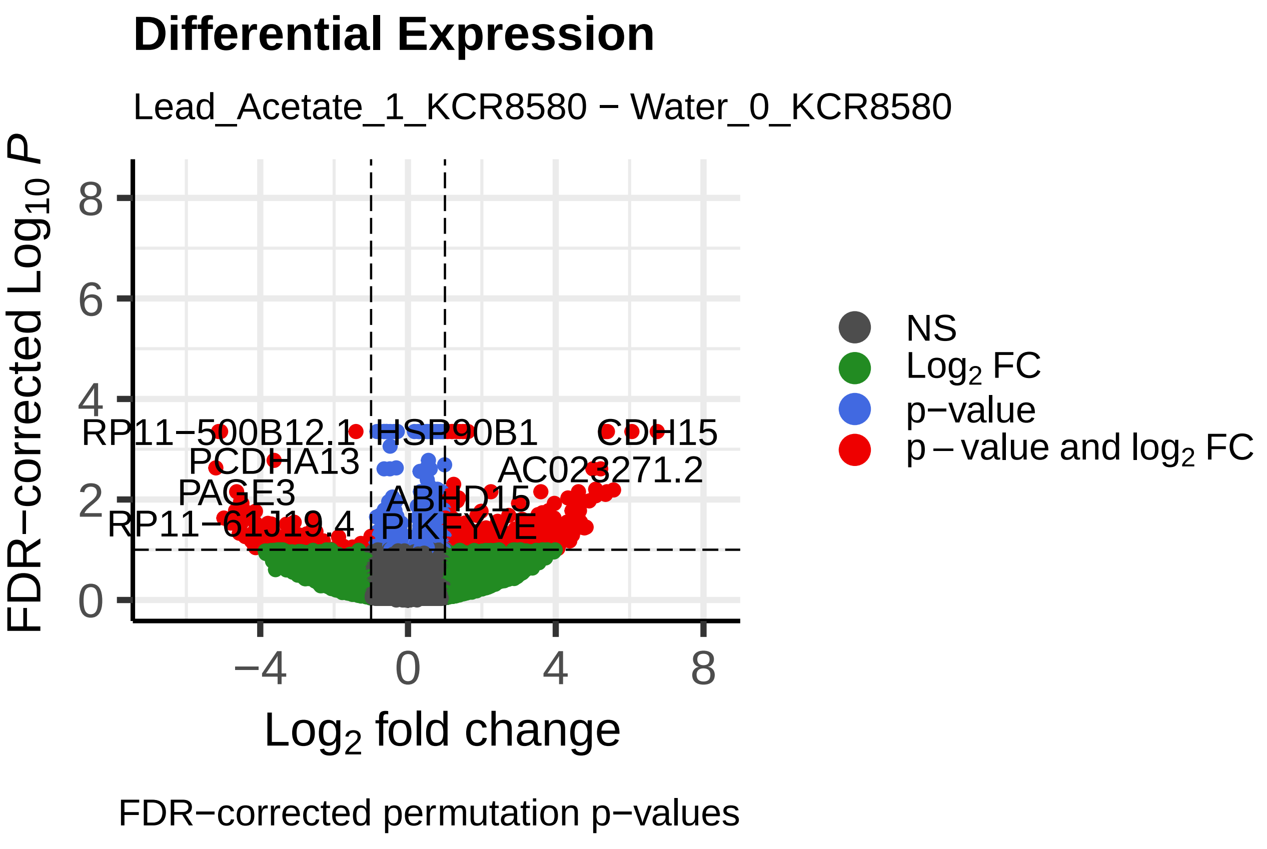
Differential Expression
Lead_Acetate_1_KCR8580 − Water_0_KCR8580
FDR−corrected $Log_{10}$ $P$
Log2 fold change
RP11−500B12.1
HSP90B1
CDH15
PCDHA13
AC023271.2
PAGE3
ABHD15
RP11−61J19.4
PIKFYVE
NS
Log2 FC
p−value
p − value and log2 FC
FDR−corrected permutation p−values

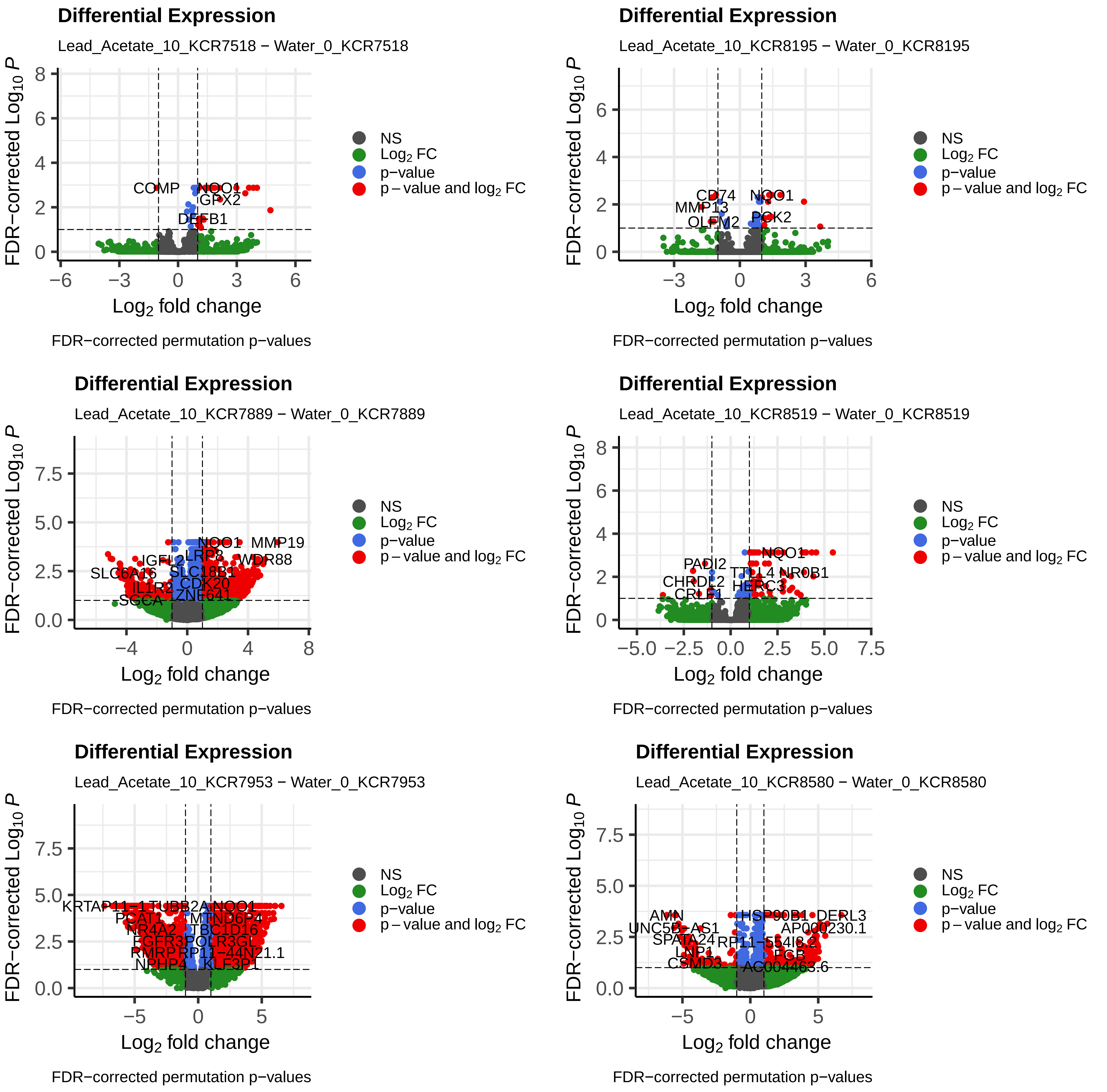
Differential Expression
Lead_Acetate_10_KCR7518 − Water_0_KCR7518
FDR−corrected Log10 P
Log2 fold change
NS
Log2 FC
p−value
p − value and log2 FC
FDR−corrected permutation p−values
Differential Expression
Lead_Acetate_10_KCR8195 − Water_0_KCR8195
FDR−corrected Log10 P
Log2 fold change
NS
Log2 FC
p−value
p − value and log2 FC
FDR−corrected permutation p−values
Differential Expression
Lead_Acetate_10_KCR7889 − Water_0_KCR7889
FDR−corrected Log10 P
Log2 fold change
NS
Log2 FC
p−value
p − value and log2 FC
FDR−corrected permutation p−values
Differential Expression
Lead_Acetate_10_KCR8519 − Water_0_KCR8519
FDR−corrected Log10 P
Log2 fold change
NS
Log2 FC
p−value
p − value and log2 FC
FDR−corrected permutation p−values
Differential Expression
Lead_Acetate_10_KCR7953 − Water_0_KCR7953
FDR−corrected Log10 P
Log2 fold change
NS
Log2 FC
p−value
p − value and log2 FC
FDR−corrected permutation p−values
Differential Expression
Lead_Acetate_10_KCR8580 − Water_0_KCR8580
FDR−corrected Log10 P
Log2 fold change
NS
Log2 FC
p−value
p − value and log2 FC
FDR−corrected permutation p−values

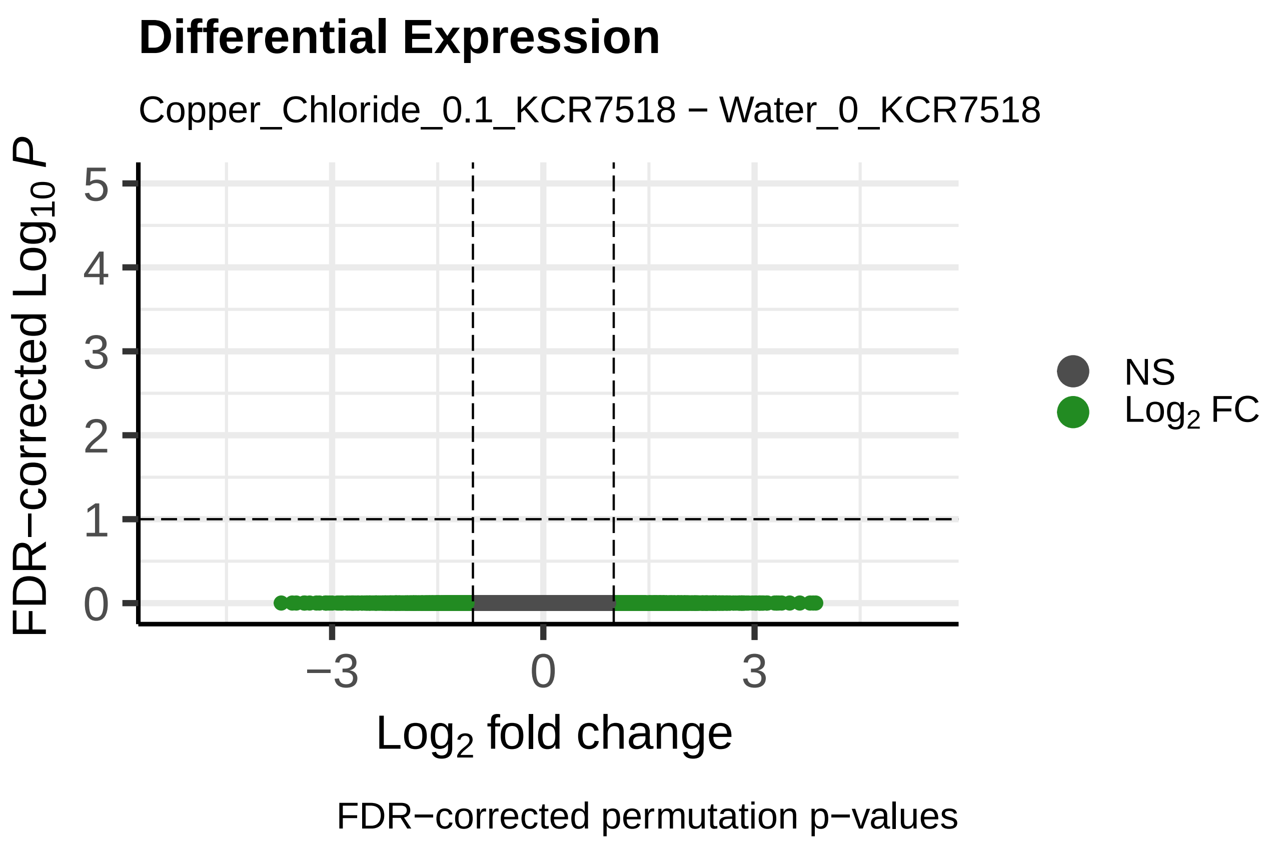
Differential Expression
Copper_Chloride_0.1_KCR7518 − Water_0_KCR7518
FDR−corrected Log10 P
Log2 fold change
NS
Log2 FC
FDR−corrected permutation p−values

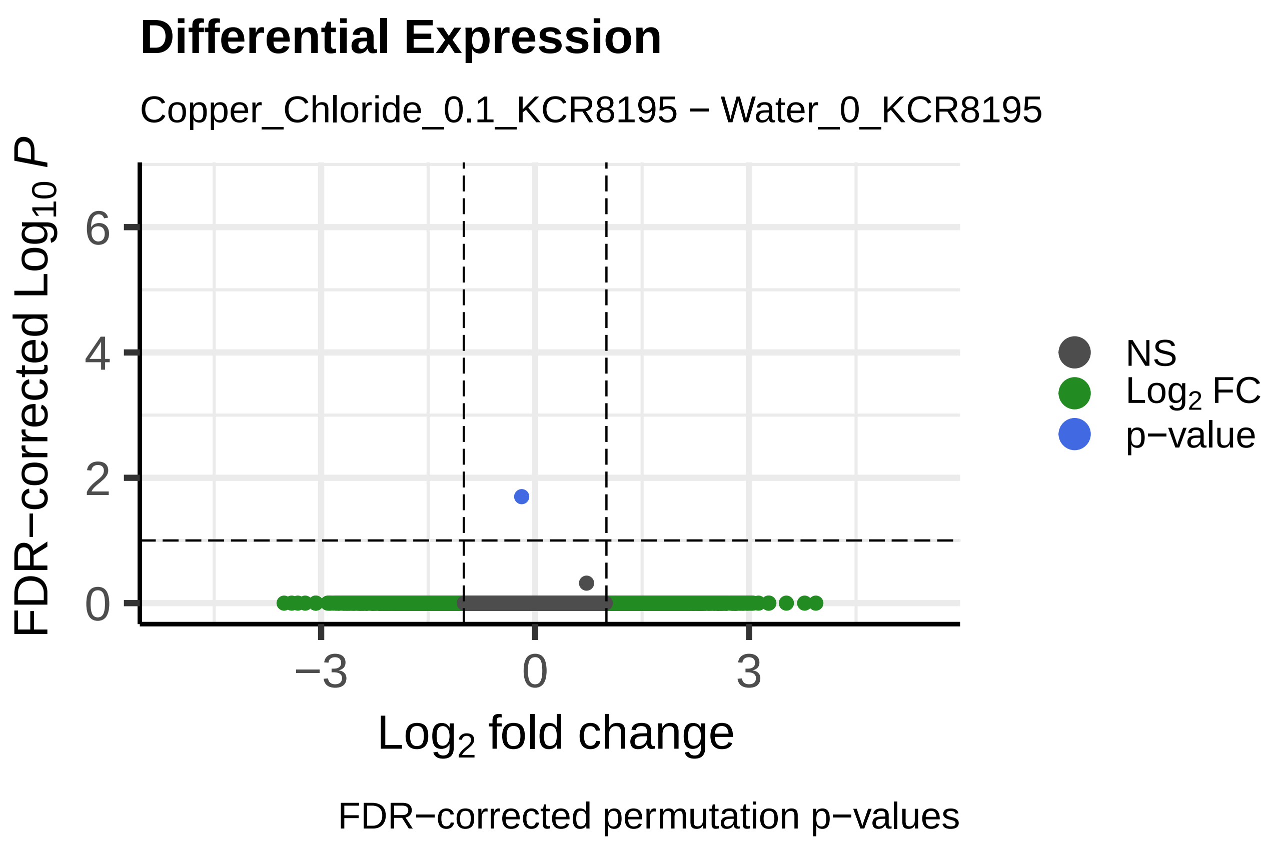
Differential Expression
Copper_Chloride_0.1_KCR8195 − Water_0_KCR8195
FDR−corrected Log10 P
Log2 fold change
NS
Log2 FC
p−value
FDR−corrected permutation p−values

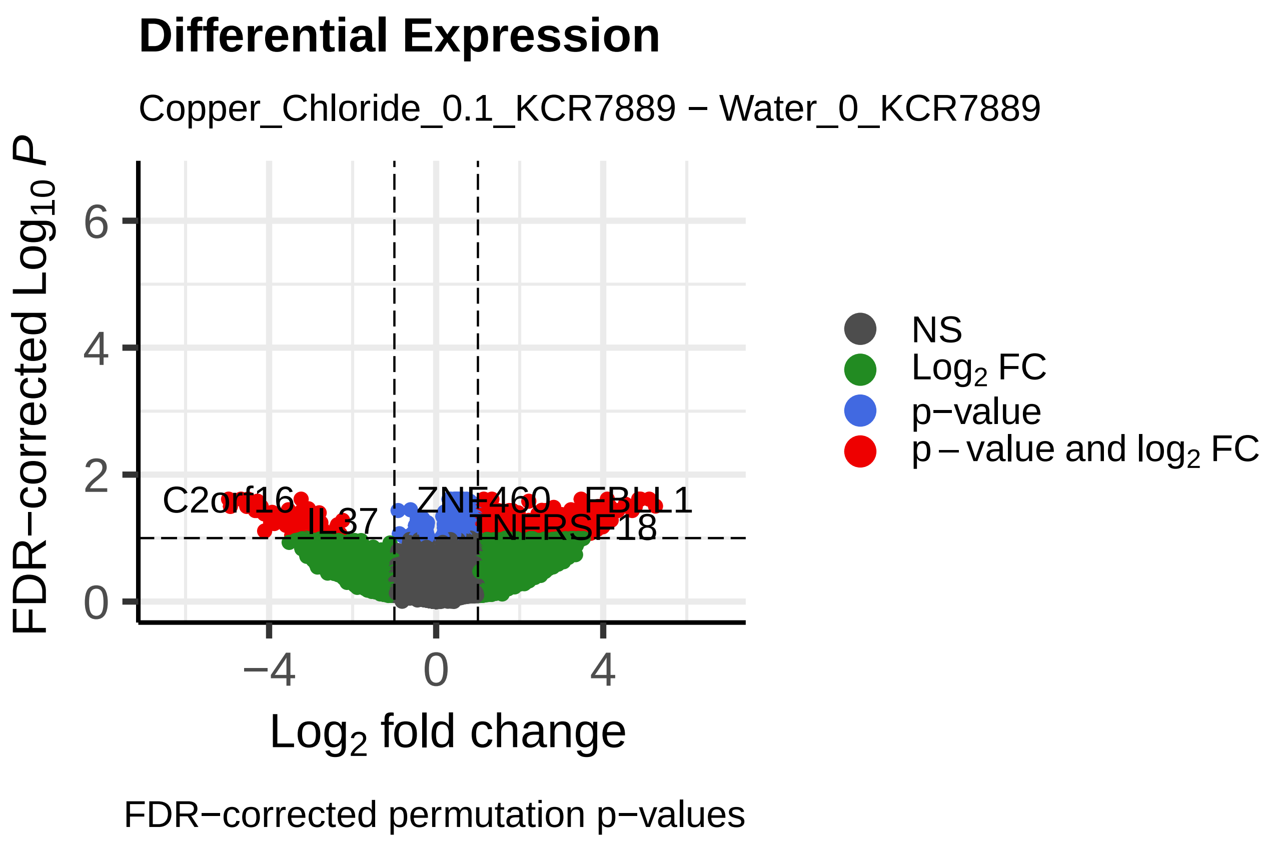
Differential Expression
Copper_Chloride_0.1_KCR7889 − Water_0_KCR7889
FDR−corrected Log10 P
Log2 fold change
C2orf16
IL37
FBLL1
TNFRSF18
NS
Log2 FC
p−value
p − value and log2 FC
FDR−corrected permutation p−values

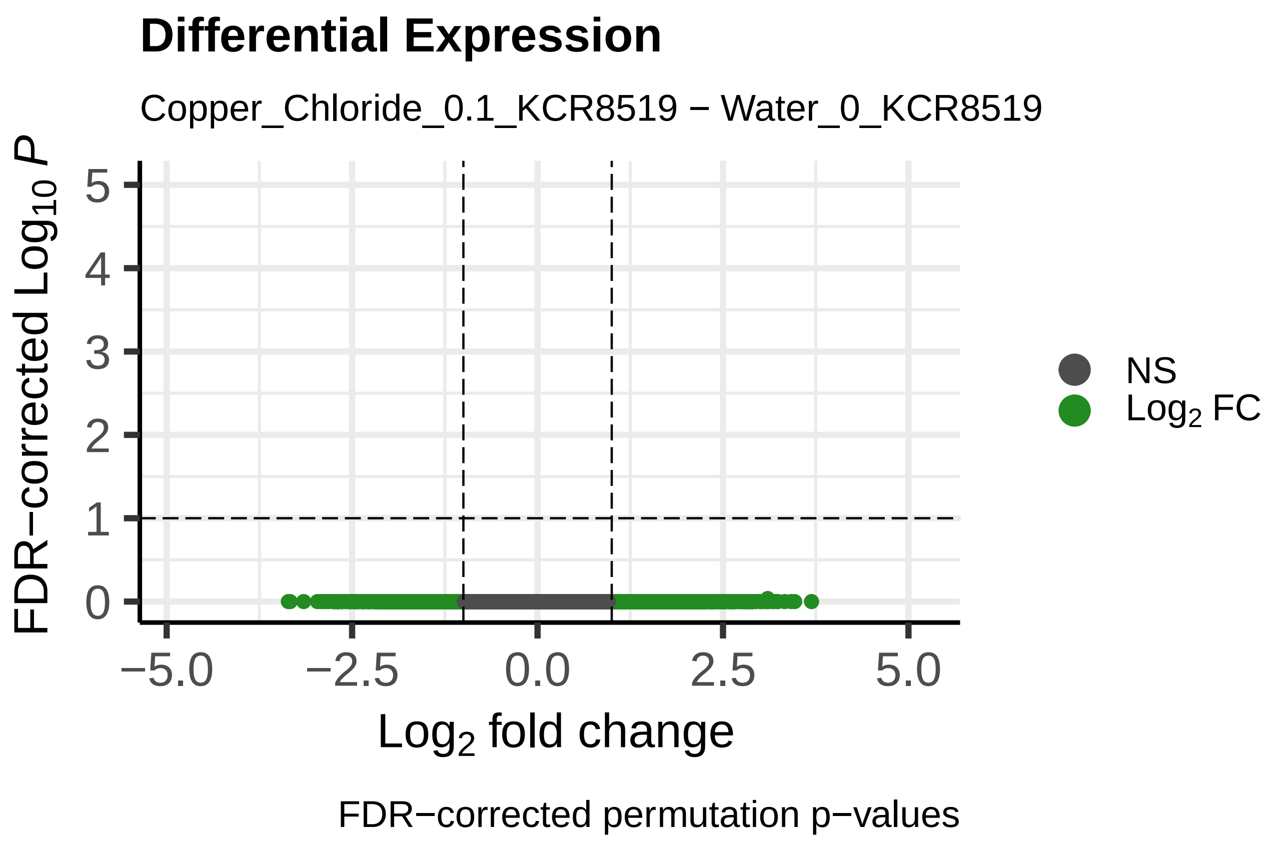
Differential Expression
Copper_Chloride_0.1_KCR8519 − Water_0_KCR8519
FDR−corrected Log10 P
Log2 fold change
NS
Log2 FC
FDR−corrected permutation p−values

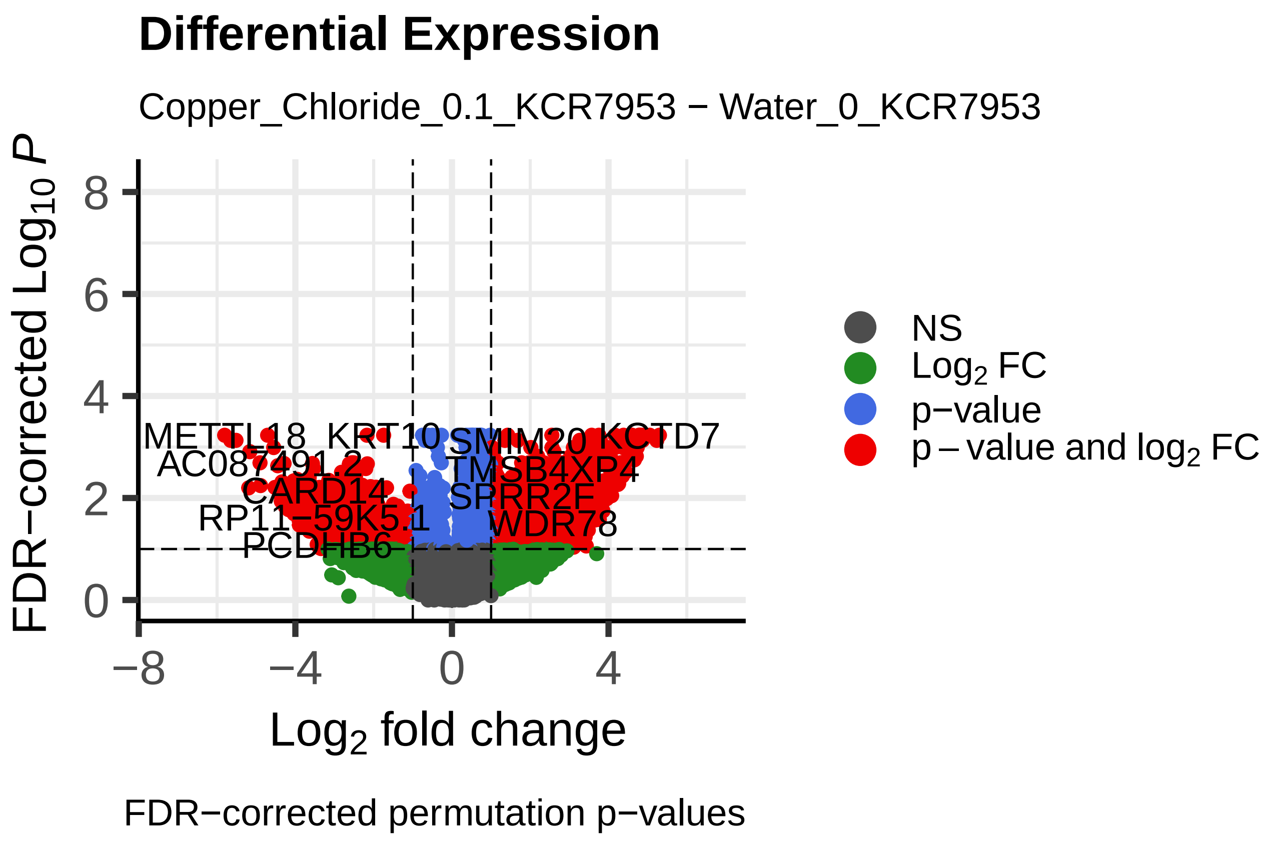
Differential Expression
Copper_Chloride_0.1_KCR7953 − Water_0_KCR7953
FDR−corrected Log10 P
Log2 fold change
KCTD7
TMSB4XP4
WDR78
PCDHB6
NS
Log2 FC
p−value
p − value and log2 FC
FDR−corrected permutation p−values

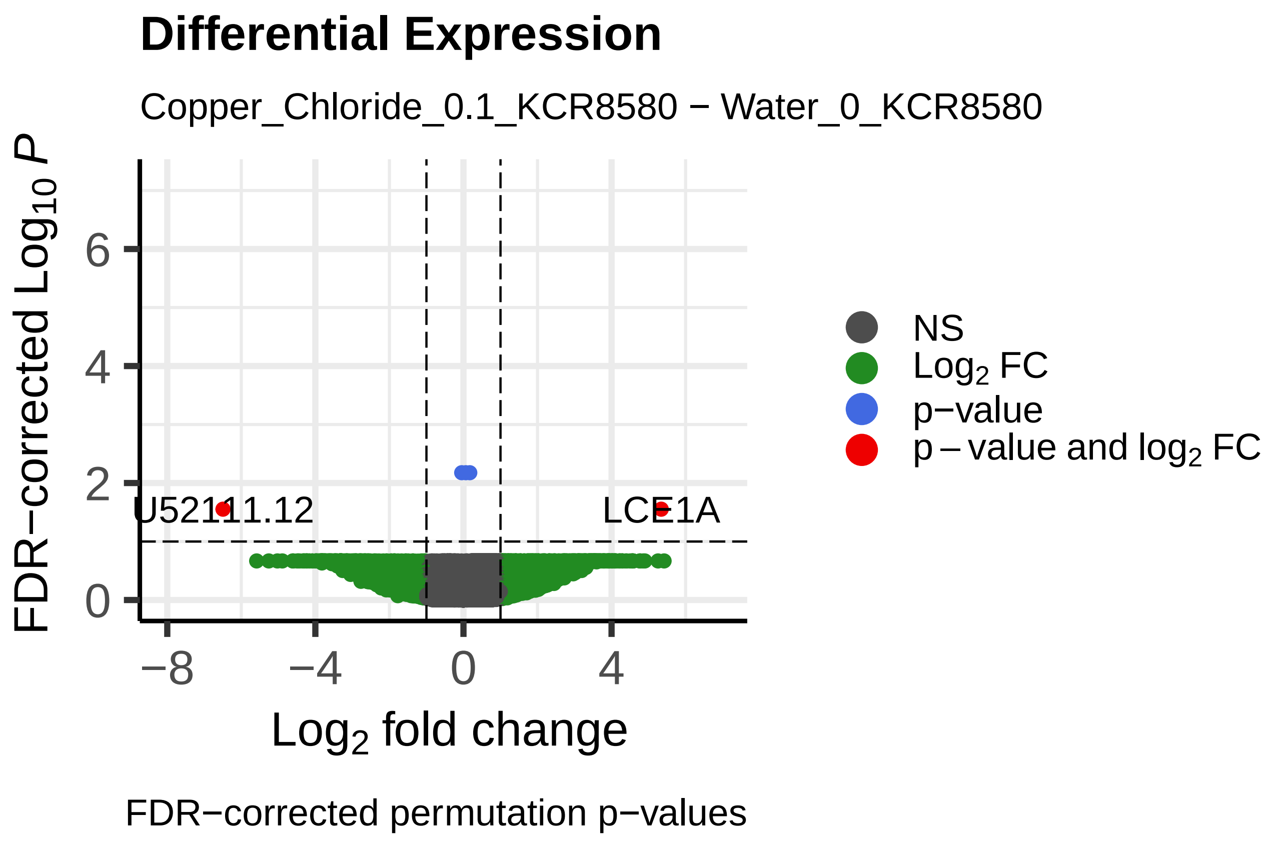
Differential Expression
Copper_Chloride_0.1_KCR8580 − Water_0_KCR8580
FDR−corrected Log10 P
Log2 fold change
U52111.12
LCE1A
NS
Log2 FC
p−value
p − value and log2 FC
FDR−corrected permutation p−values

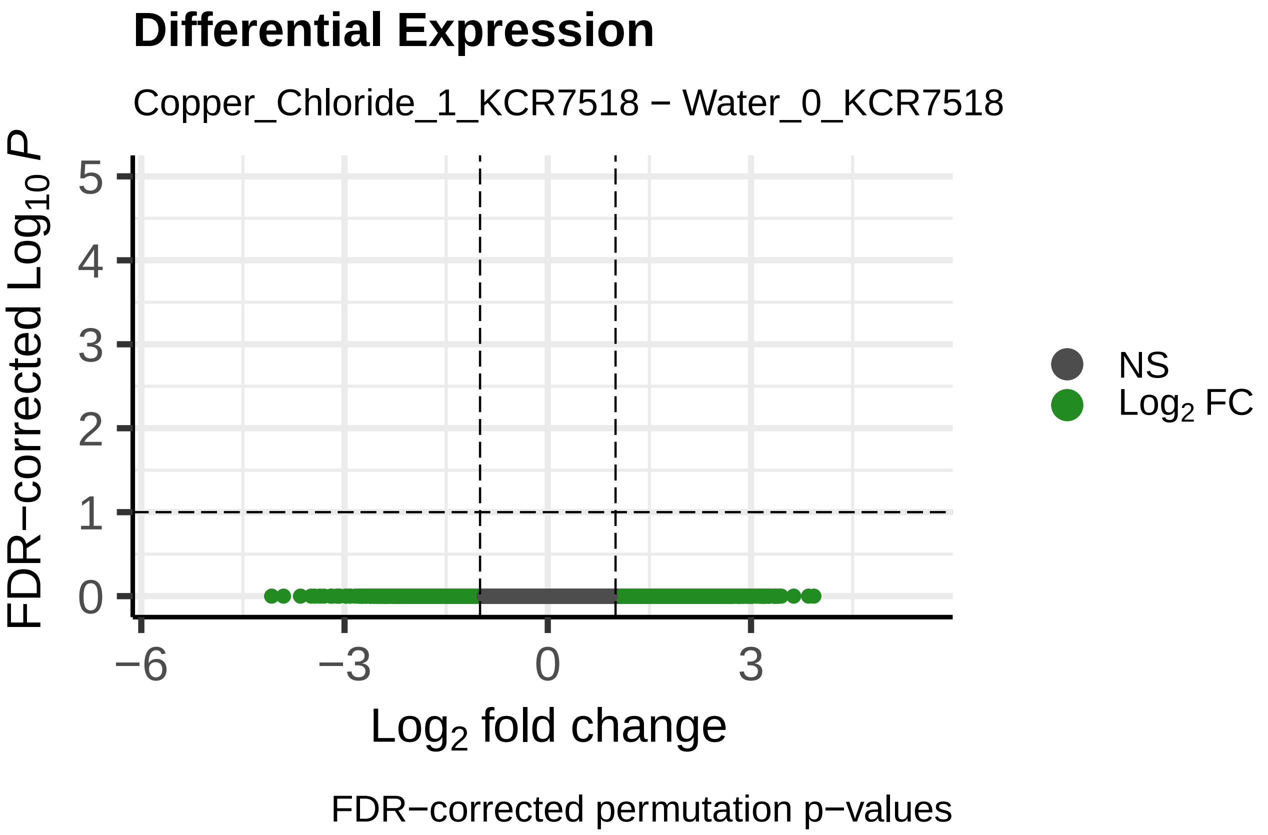
Differential Expression
Copper_Chloride_1_KCR7518 − Water_0_KCR7518
FDR−corrected $Log_{10}$ P
Log₂ fold change
NS
$Log_2$ FC
FDR−corrected permutation p−values

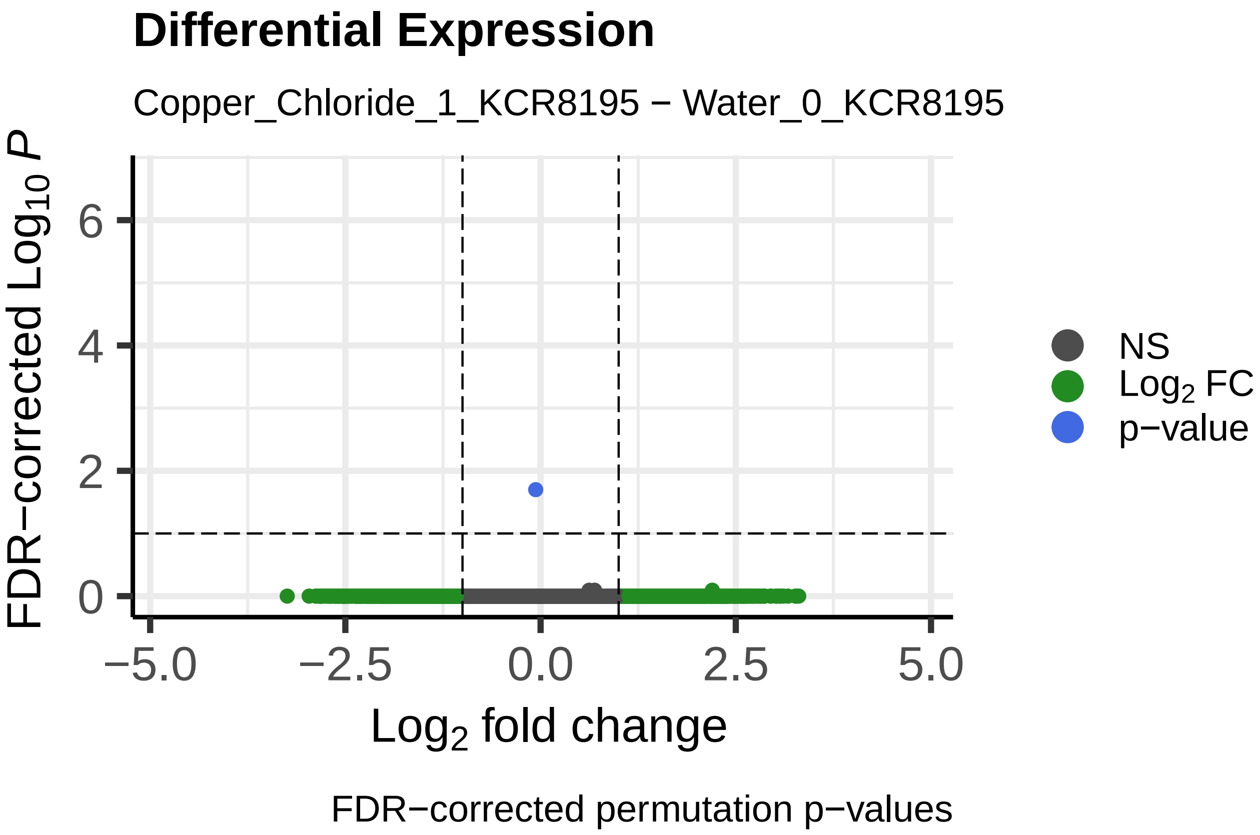
Differential Expression
Copper_Chloride_1_KCR8195 − Water_0_KCR8195
FDR−corrected $Log_{10}$ P
$Log_2$ fold change
NS
$Log_2$ FC
p−value
FDR−corrected permutation p−values

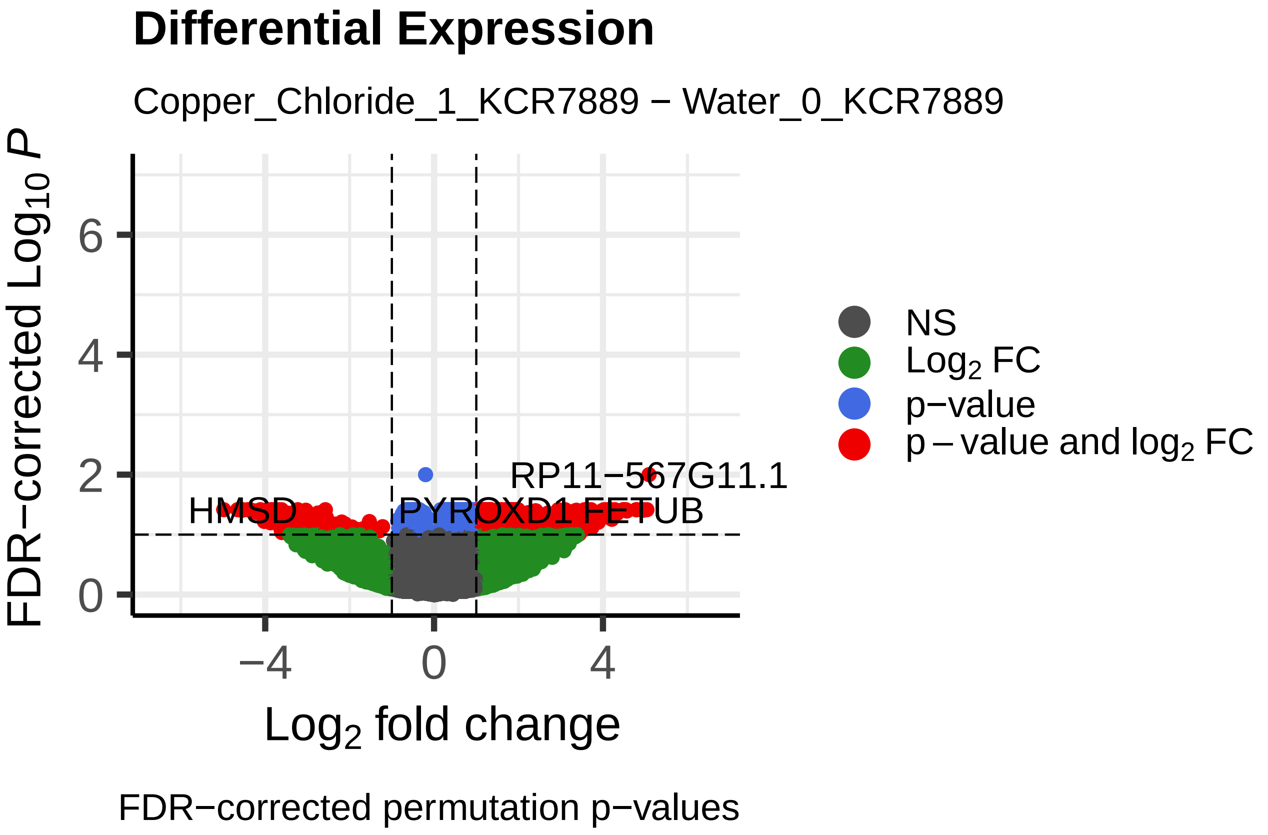
Differential Expression
Copper_Chloride_1_KCR7889 − Water_0_KCR7889
FDR−corrected $Log_{10}$ P
$Log_2$ fold change
RP11−567G11.1
HMSD
PYROXD1
FETUB
NS
$Log_2$ FC
p−value
p − value and $log_2$ FC
FDR−corrected permutation p−values

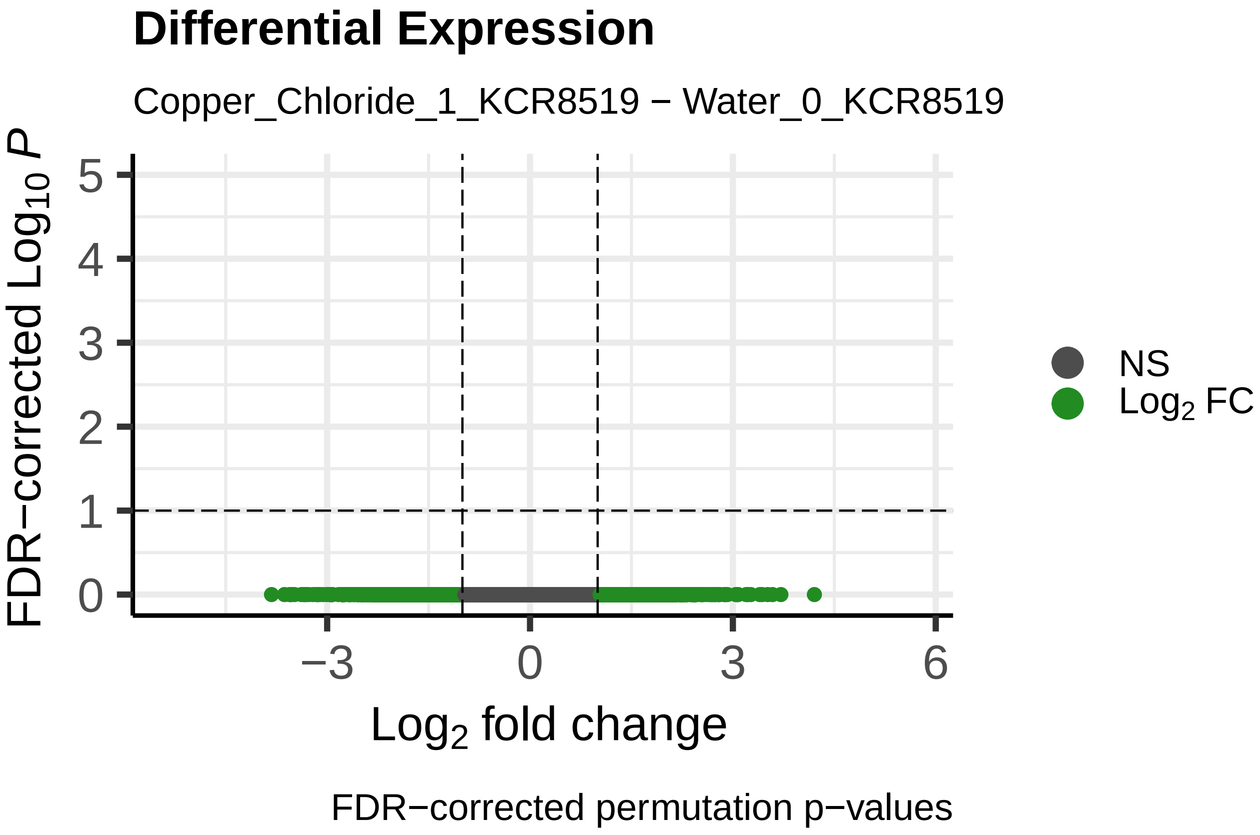
Differential Expression
Copper_Chloride_1_KCR8519 − Water_0_KCR8519
FDR−corrected $Log_{10}$ P
$Log_2$ fold change
NS
$Log_2$ FC
FDR−corrected permutation p−values

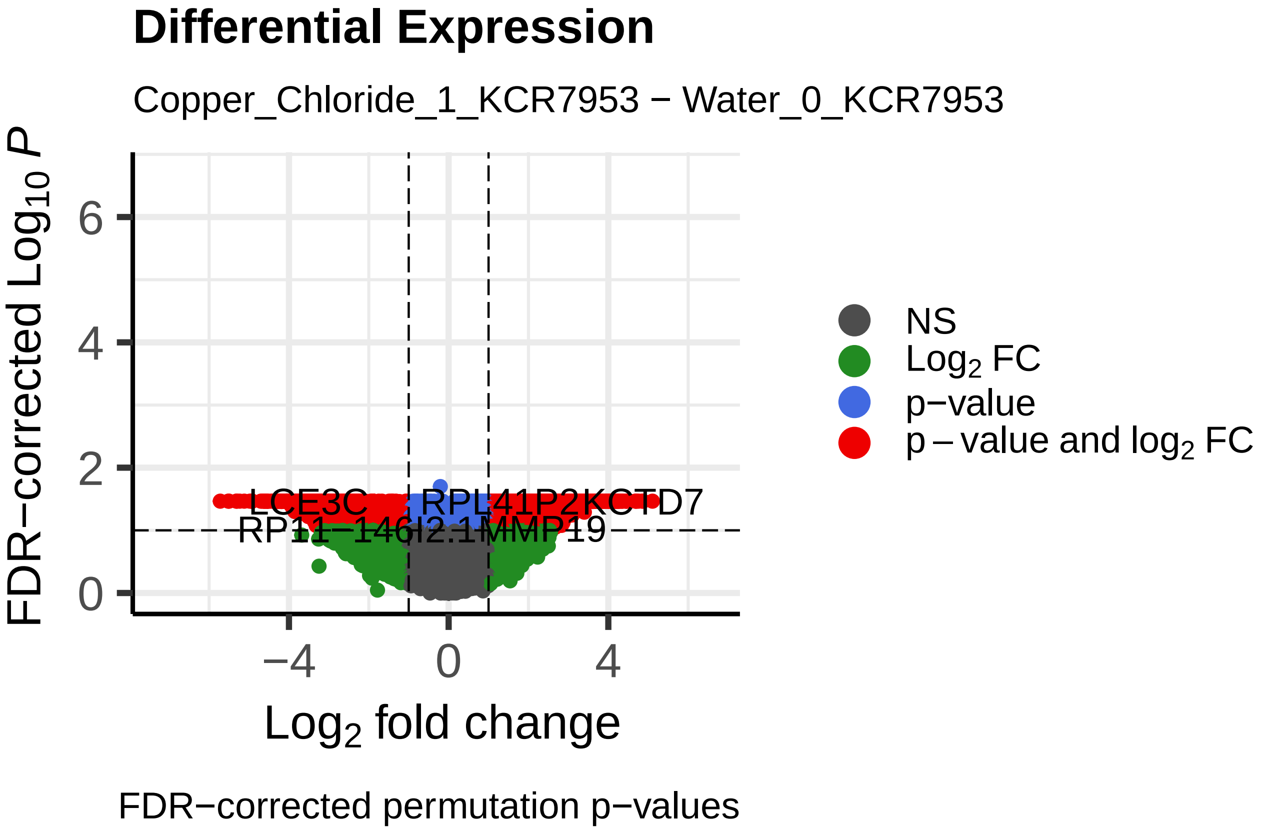
Differential Expression
Copper_Chloride_1_KCR7953 − Water_0_KCR7953
FDR−corrected $Log_{10}$ P
$Log_2$ fold change
LCE3C
RPL41P2
KCTD7
RP11−146I2.1
MMP19
NS
$Log_2$ FC
p−value
p − value and $log_2$ FC
FDR−corrected permutation p−values

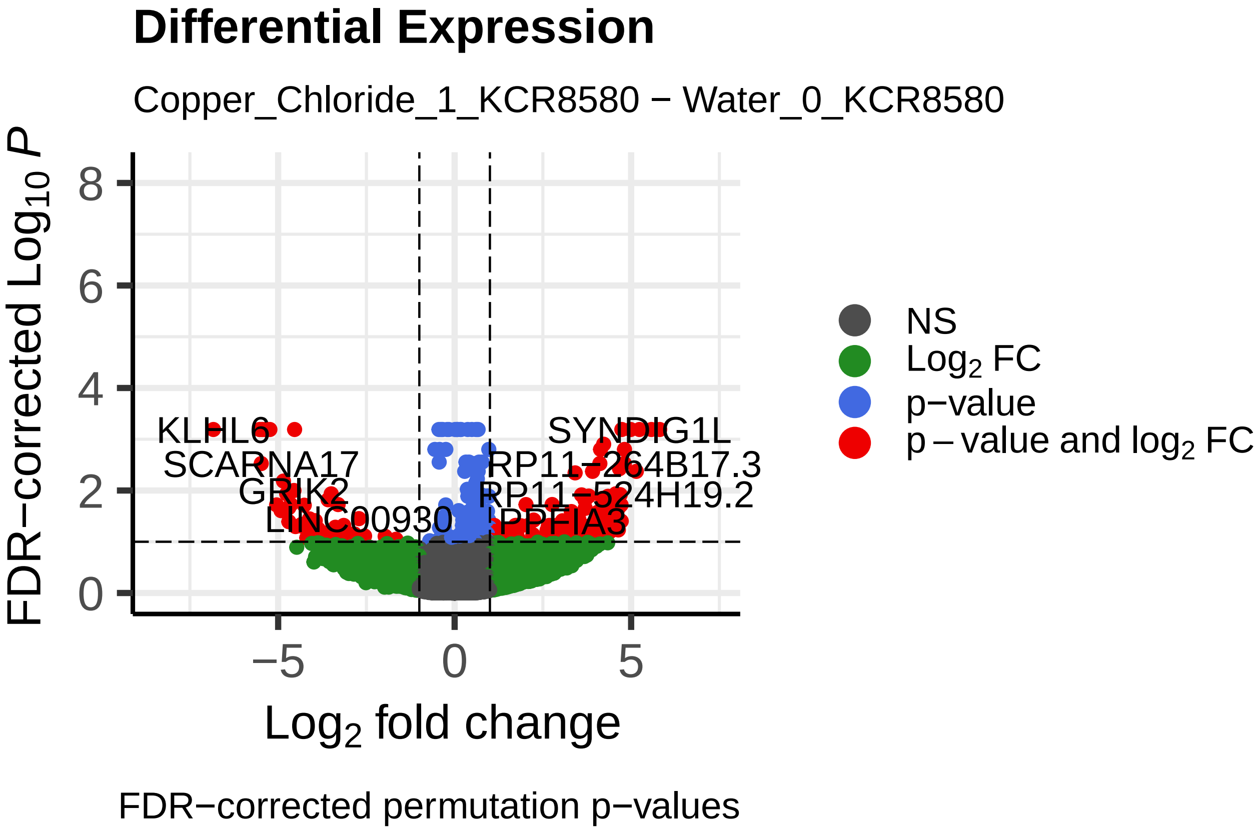
Differential Expression
Copper_Chloride_1_KCR8580 − Water_0_KCR8580
FDR−corrected $Log_{10}$ P
$Log_2$ fold change
KLHL6
SCARNA17
SYNDIG1L
RP11−524H19.2
PPFIA3
NS
$Log_2$ FC
p−value
p − value and $log_2$ FC
FDR−corrected permutation p−values

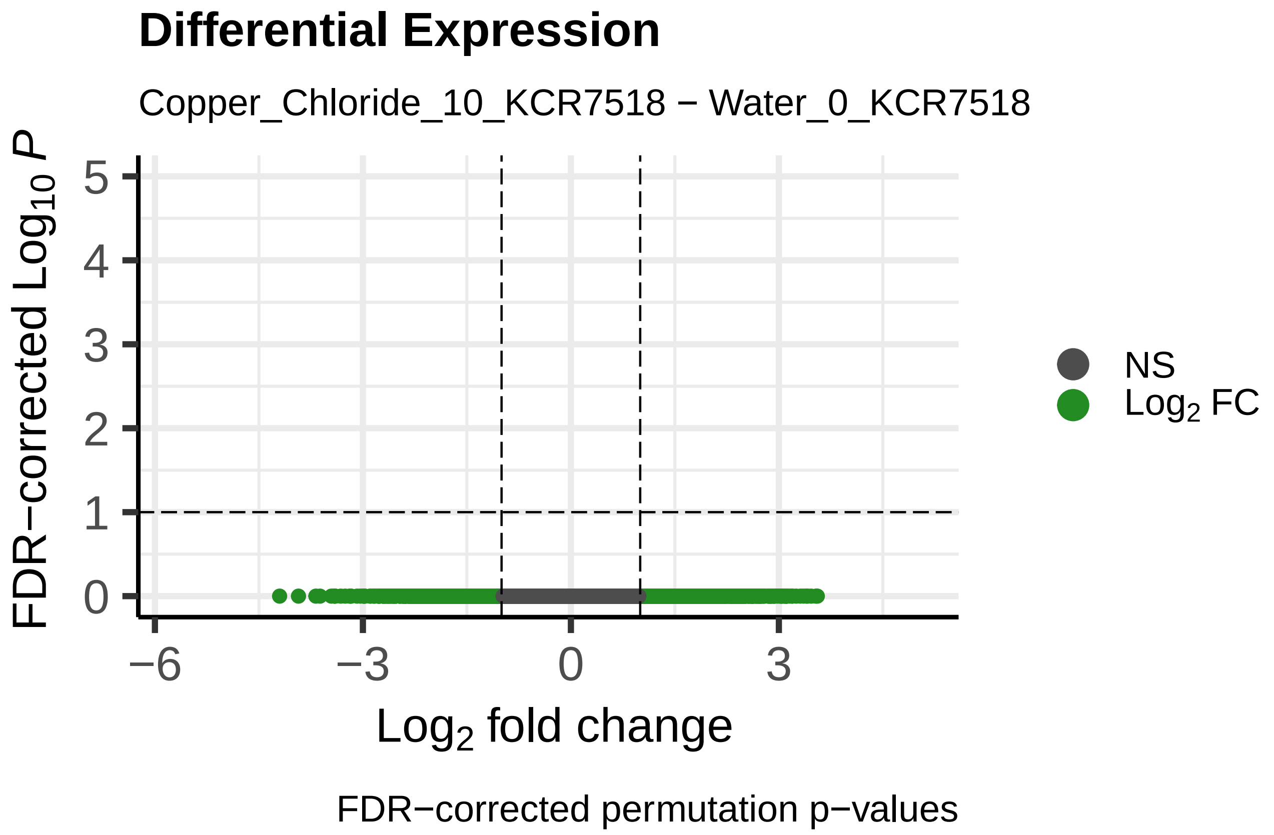
Differential Expression
Copper_Chloride_10_KCR7518 − Water_0_KCR7518
FDR−corrected Log10 P
Log2 fold change
NS
Log2 FC
FDR−corrected permutation p−values

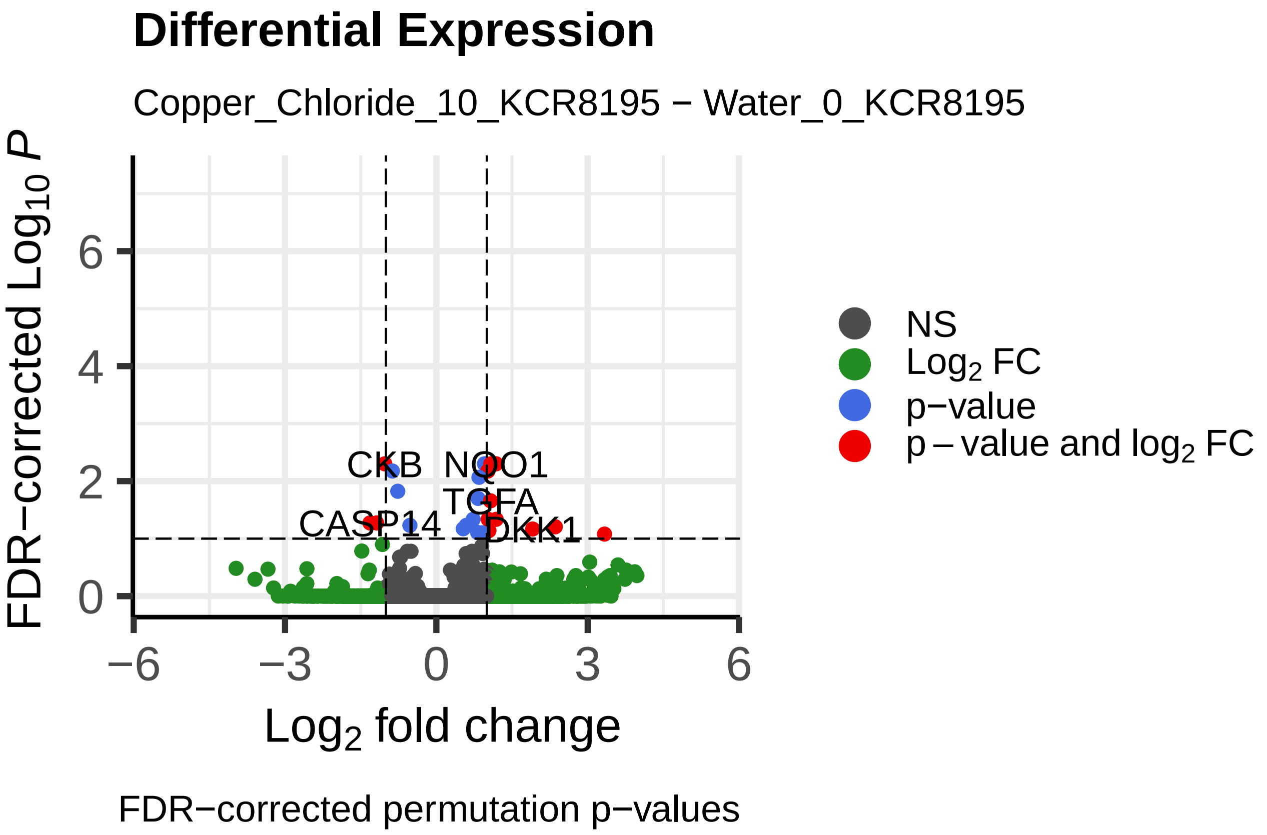
Differential Expression
Copper_Chloride_10_KCR8195 − Water_0_KCR8195
FDR−corrected Log10 P
Log2 fold change
CKB
NQO1
TGFA
CASP14
DKK1
NS
Log2 FC
p−value
p − value and log2 FC
FDR−corrected permutation p−values

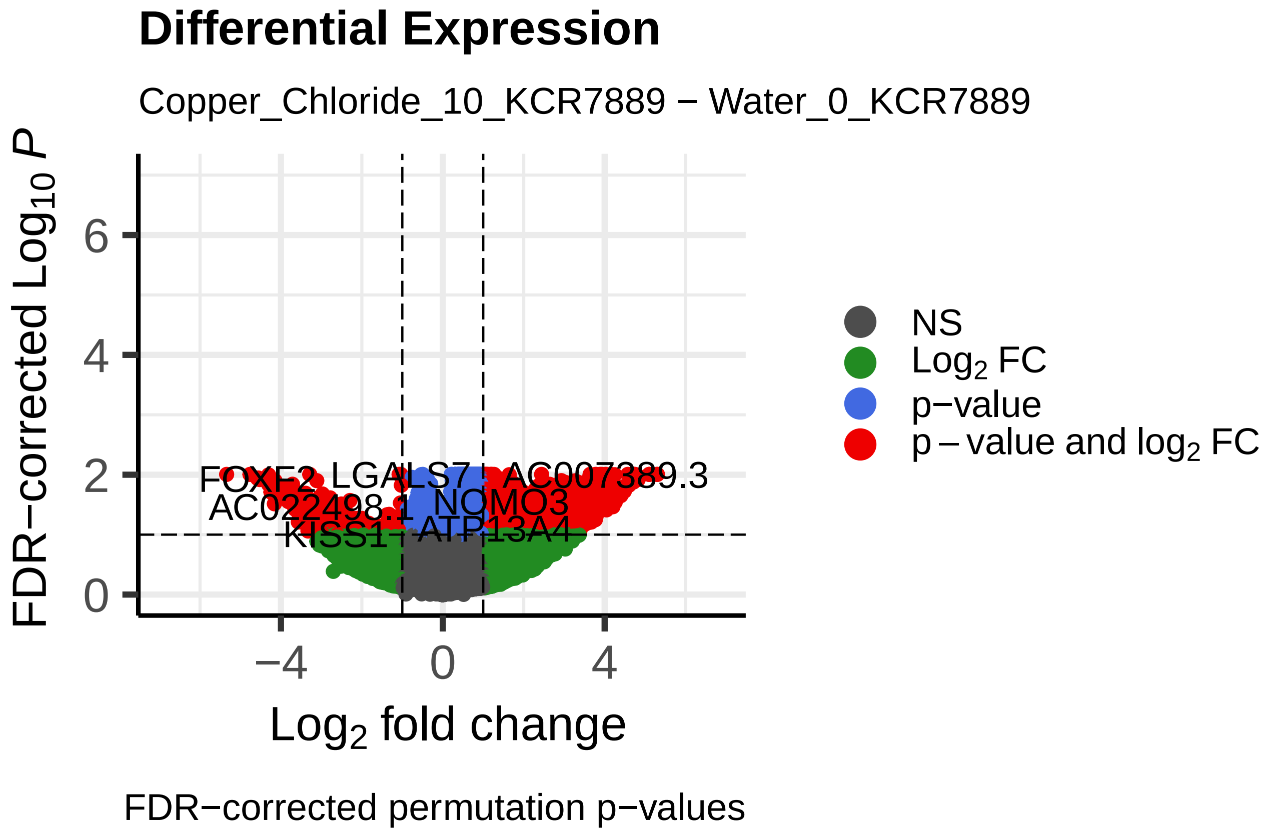
Differential Expression
Copper_Chloride_10_KCR7889 − Water_0_KCR7889
FDR−corrected Log10 P
Log2 fold change
FOXF2
LGALS7
AC007389.3
AC022498.1
NOMO3
KISS1
ATP13A4
NS
Log2 FC
p−value
p − value and log2 FC
FDR−corrected permutation p−values

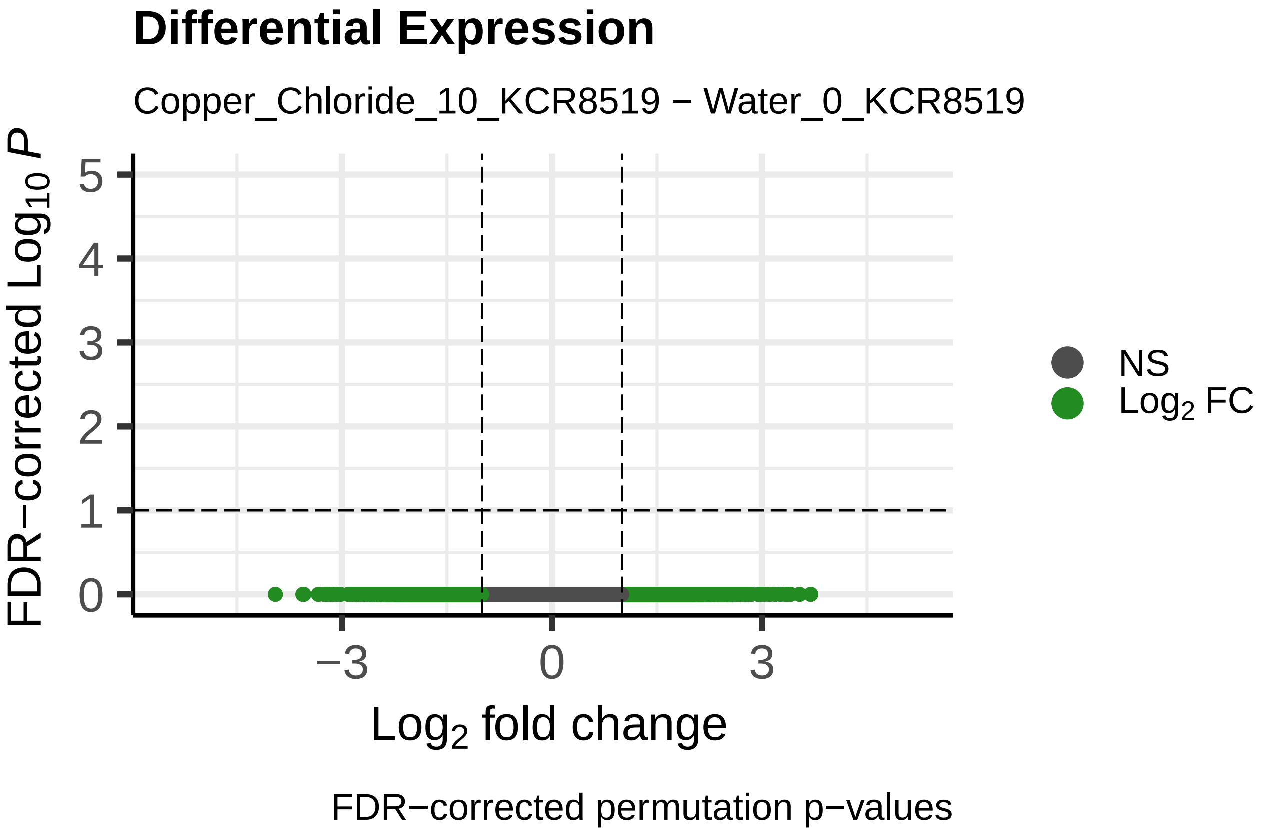
Differential Expression
Copper_Chloride_10_KCR8519 − Water_0_KCR8519
FDR−corrected Log10 P
Log2 fold change
NS
Log2 FC
FDR−corrected permutation p−values

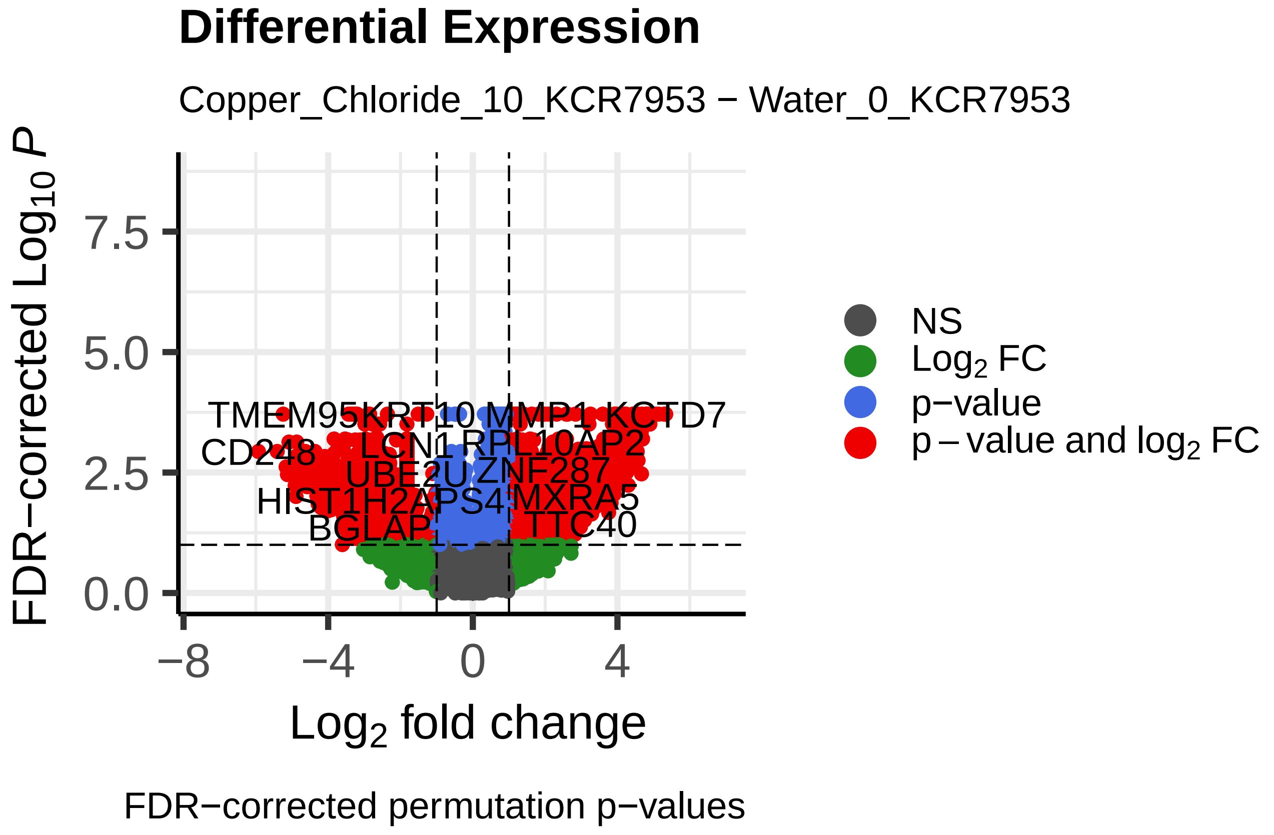
Differential Expression
Copper_Chloride_10_KCR7953 − Water_0_KCR7953
FDR−corrected Log10 P
Log2 fold change
TMEM95
KRT10
MMP1
KCTD7
CD248
LCN1
RPL10AP2
UBE2U
ZNF287
HIST1H2APS4
MXRA5
BGLAP
TTC40
NS
Log2 FC
p−value
p − value and log2 FC
FDR−corrected permutation p−values

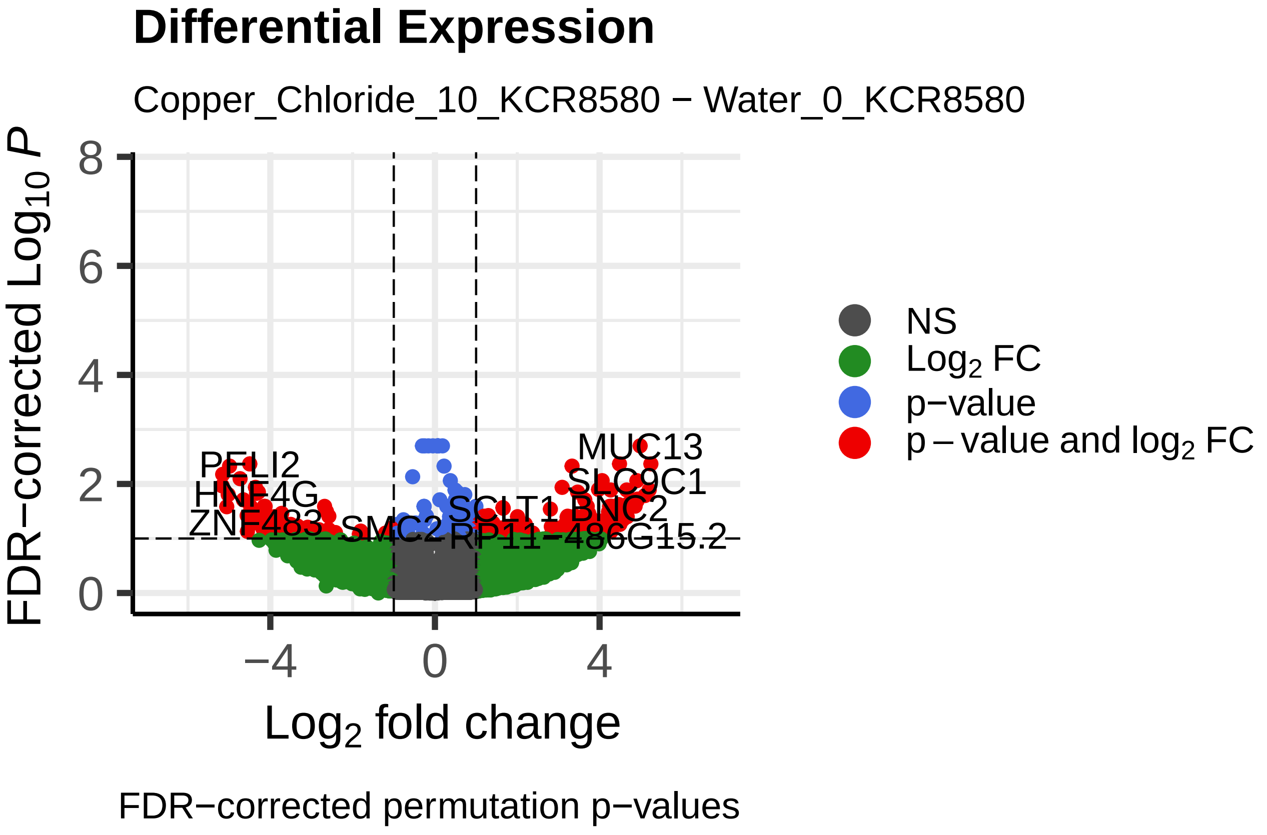
Differential Expression
Copper_Chloride_10_KCR8580 − Water_0_KCR8580
FDR−corrected Log10 P
Log2 fold change
PELI2
MUC13
HNF4G
SLC9C1
ZNF483
SCLT1
BNC2
SMC2
RP11−486G15.2
NS
Log2 FC
p−value
p − value and log2 FC
FDR−corrected permutation p−values

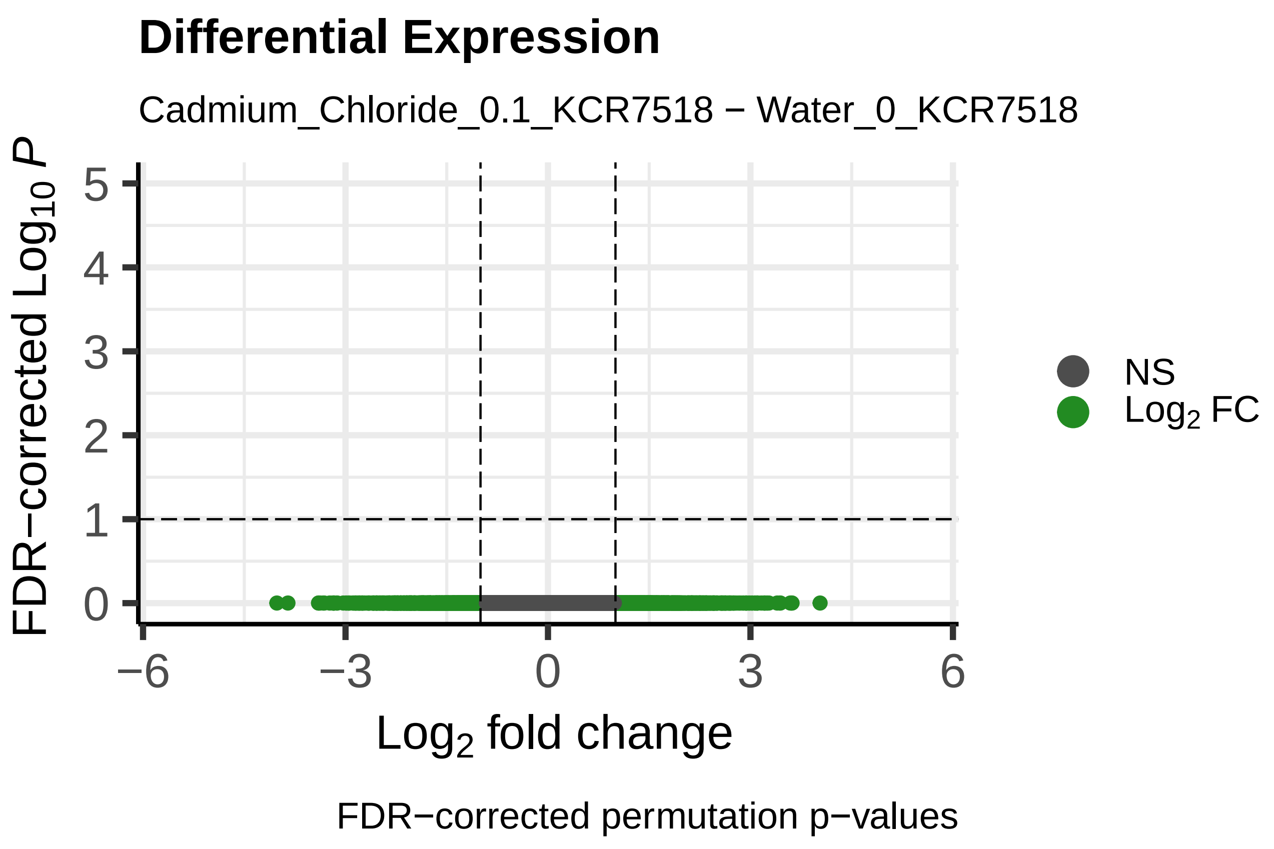
Differential Expression
Cadmium_Chloride_0.1_KCR7518 − Water_0_KCR7518
FDR−corrected Log10 P
Log2 fold change
NS
Log2 FC
FDR−corrected permutation p−values

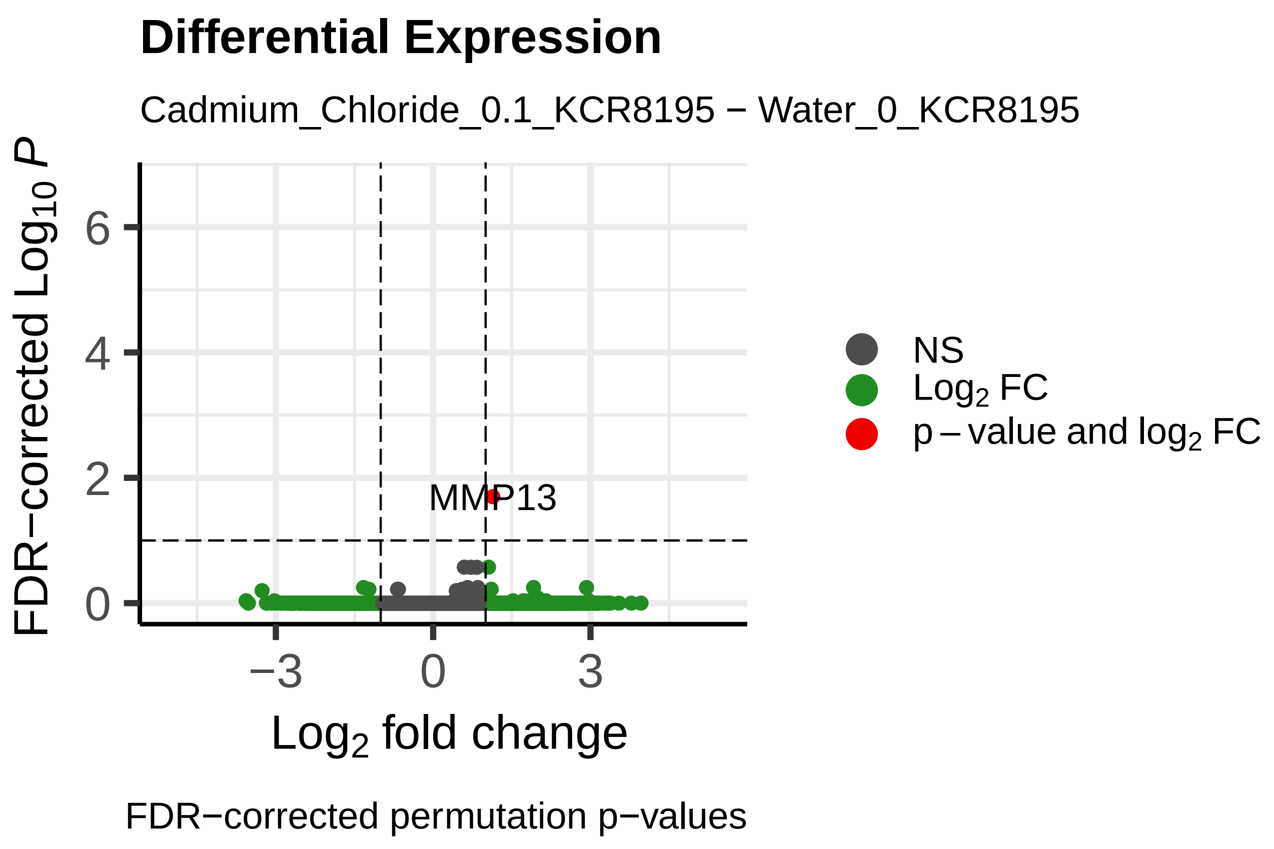
Differential Expression
Cadmium_Chloride_0.1_KCR8195 − Water_0_KCR8195
FDR−corrected Log10 P
Log2 fold change
MMP13
NS
Log2 FC
p − value and log2 FC
FDR−corrected permutation p−values

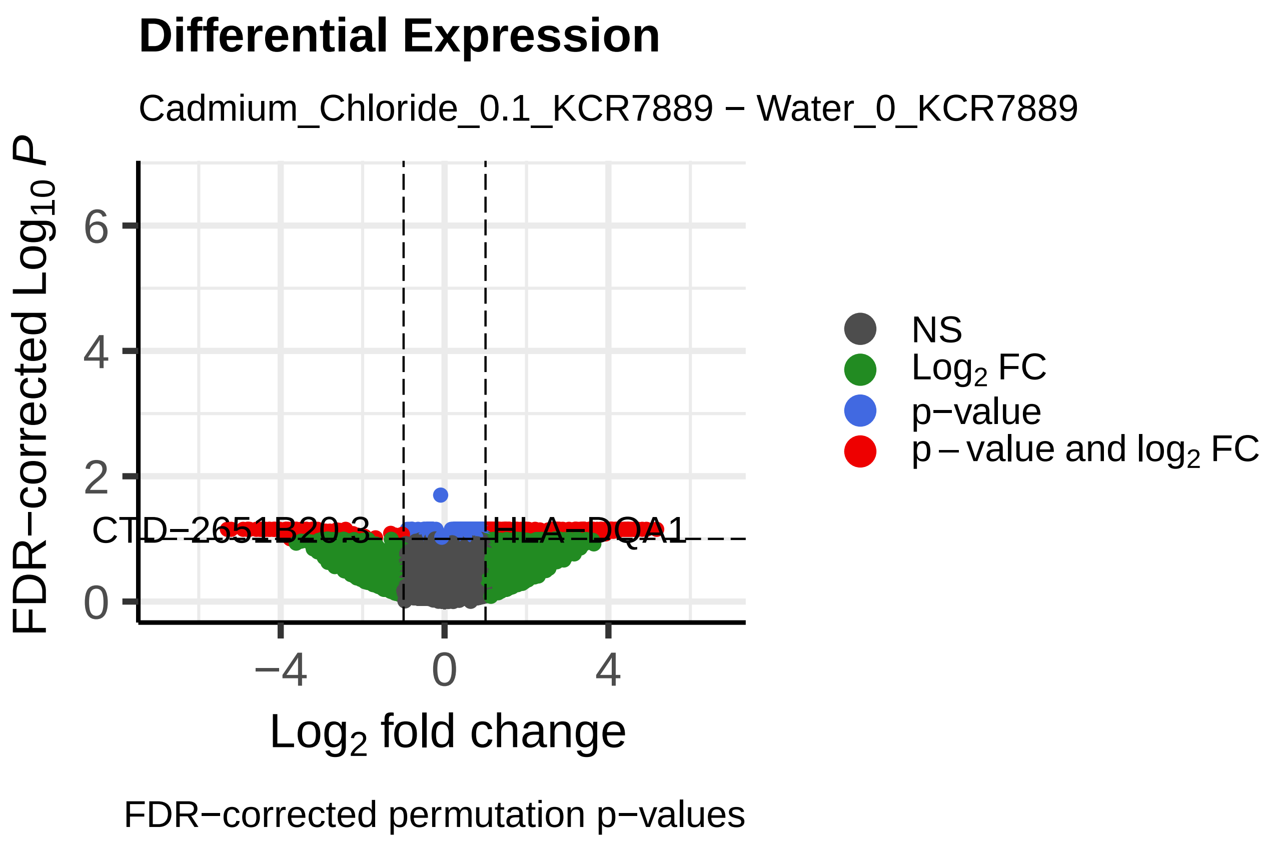
Differential Expression
Cadmium_Chloride_0.1_KCR7889 − Water_0_KCR7889
FDR−corrected Log10 P
Log2 fold change
CTD−2651B20.3
HLA−DQA1
NS
Log2 FC
p−value
p − value and log2 FC
FDR−corrected permutation p−values

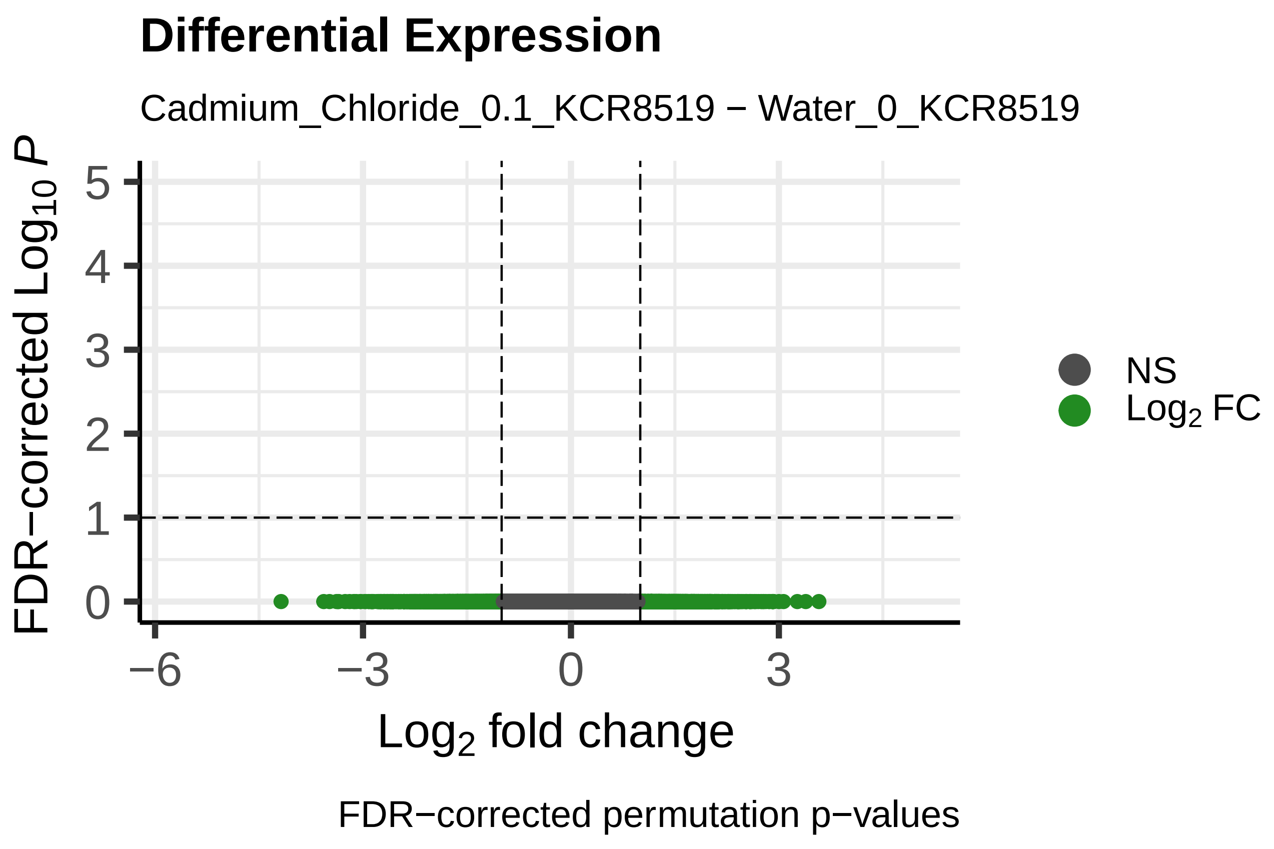
Differential Expression
Cadmium_Chloride_0.1_KCR8519 − Water_0_KCR8519
FDR−corrected Log10 P
Log2 fold change
NS
Log2 FC
FDR−corrected permutation p−values

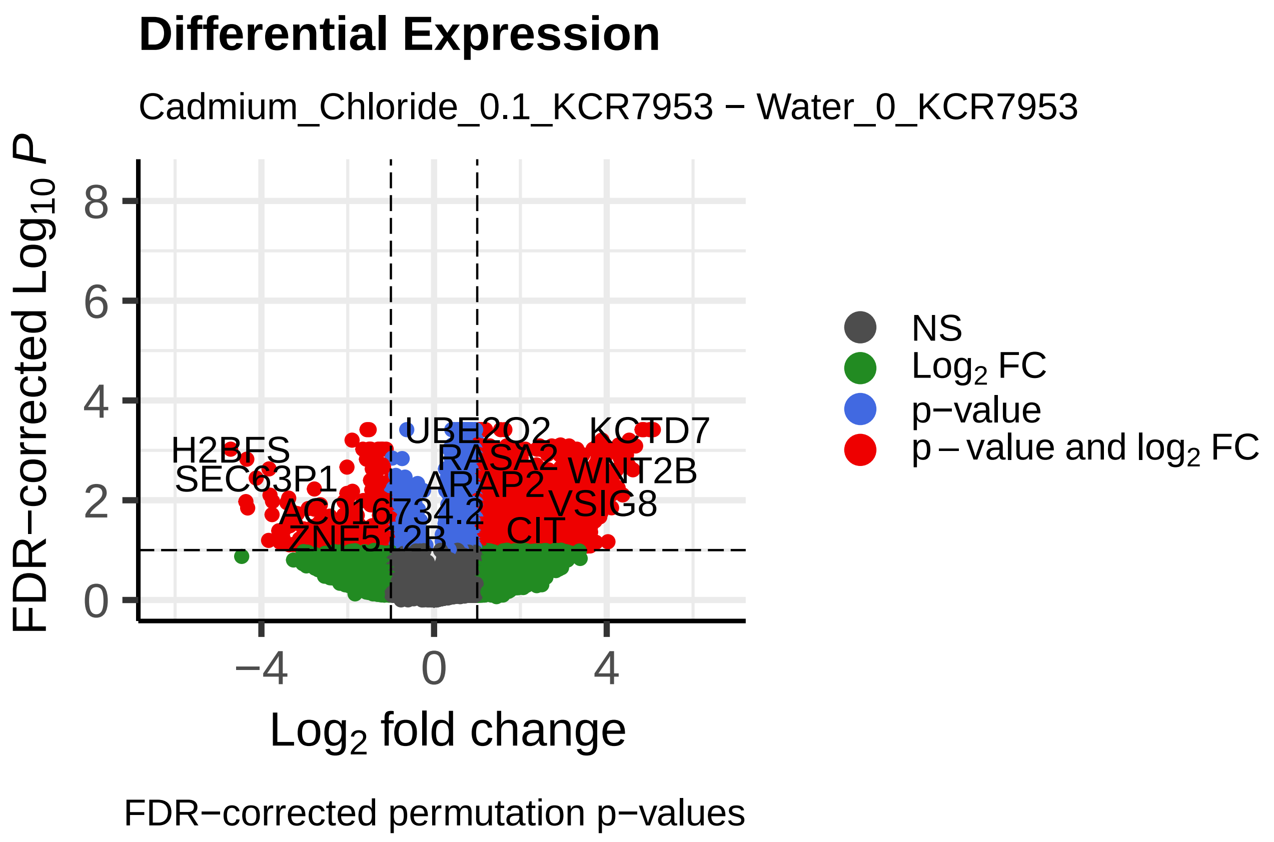
Differential Expression
Cadmium_Chloride_0.1_KCR7953 − Water_0_KCR7953
FDR−corrected Log10 P
Log2 fold change
H2BFS
SEC63P1
UBE2Q2
KCTD7
RASA2
WNT2B
ARAP2
VSIG8
AC016734.2
CIT
ZNF512B
NS
Log2 FC
p−value
p − value and log2 FC
FDR−corrected permutation p−values

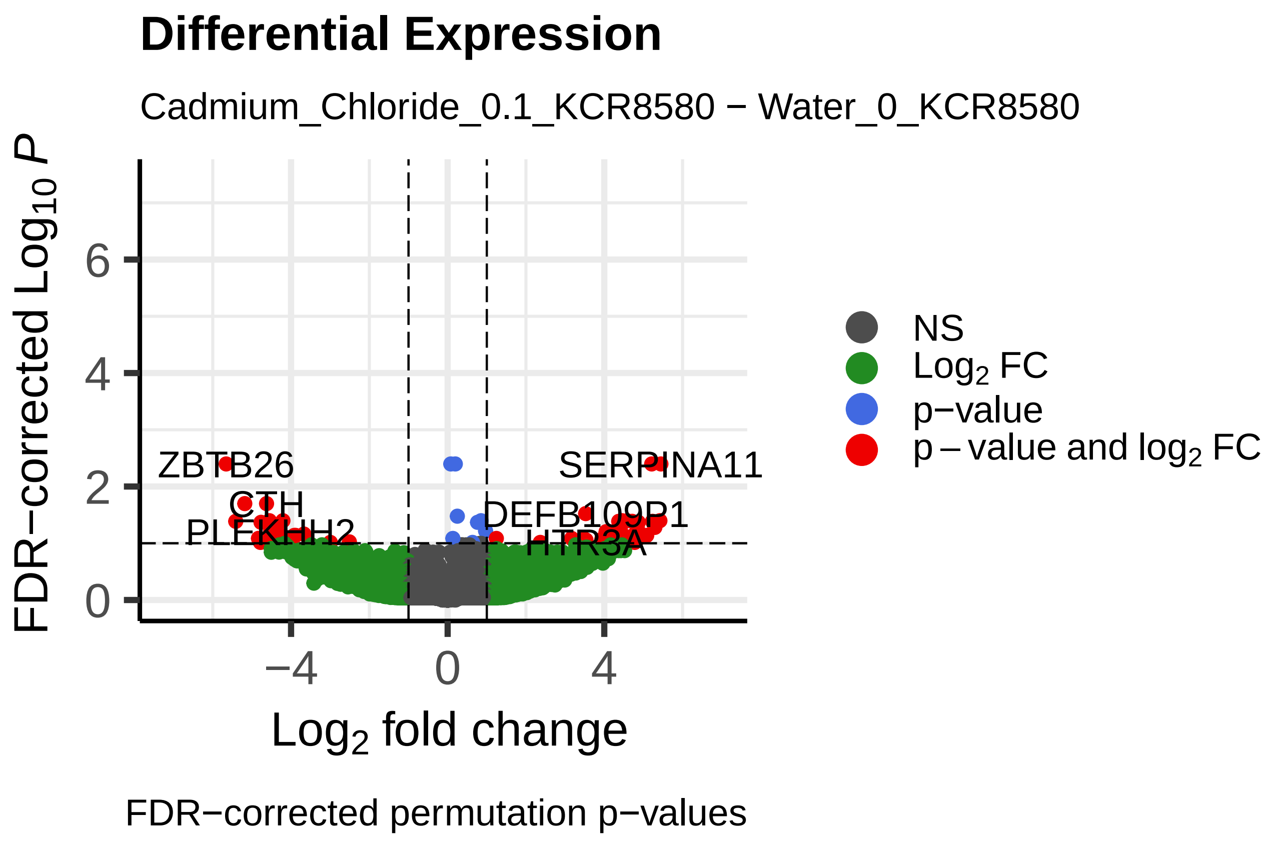
Differential Expression
Cadmium_Chloride_0.1_KCR8580 − Water_0_KCR8580
FDR−corrected Log10 P
Log2 fold change
ZBTB26
SERPINA11
CTH
DEFB109P1
PLEKHH2
HTR3A
NS
Log2 FC
p−value
p − value and log2 FC
FDR−corrected permutation p−values

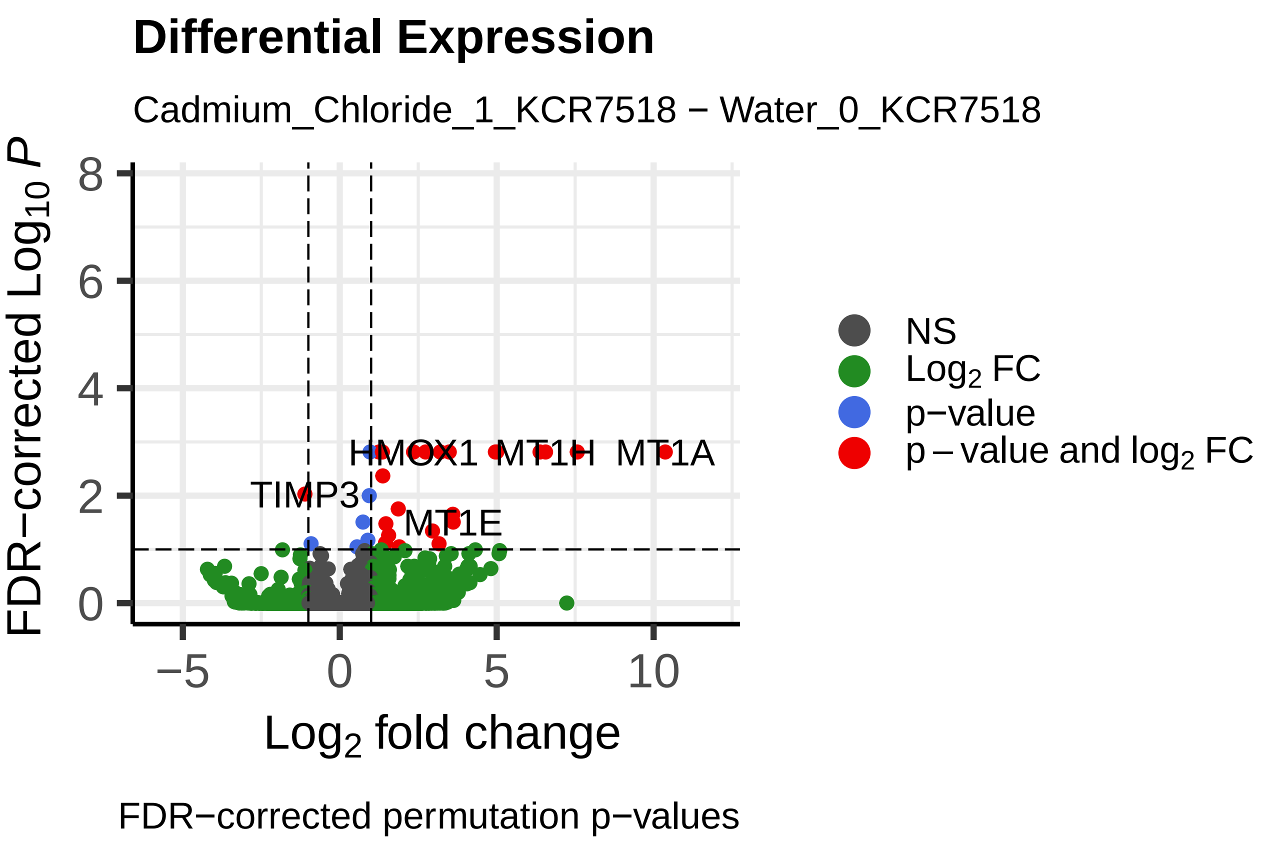
Differential Expression
Cadmium_Chloride_1_KCR7518 − Water_0_KCR7518
FDR−corrected Log10 P
Log2 fold change
HMOX1
MT1H
MT1A
TIMP3
MT1E
NS
Log2 FC
p−value
p − value and log2 FC
FDR−corrected permutation p−values

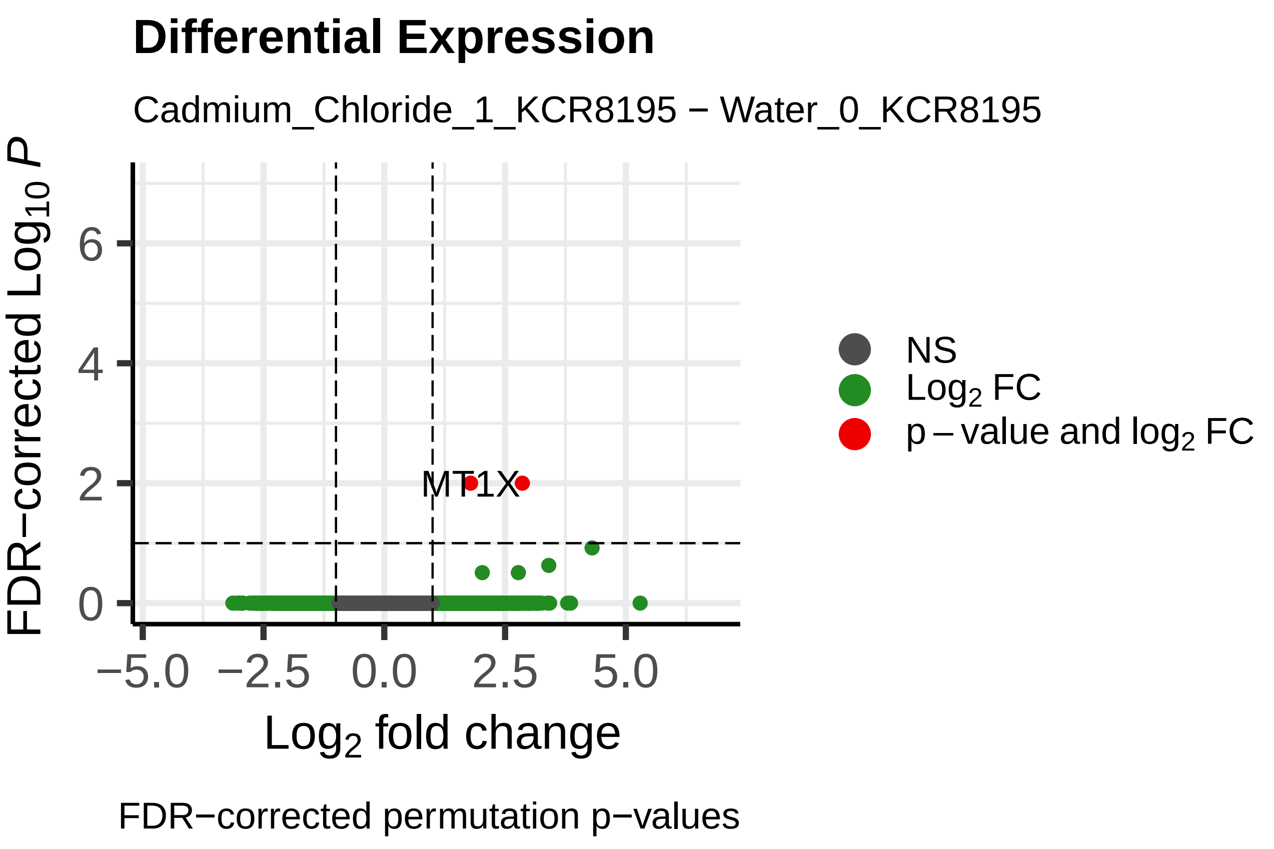
Differential Expression
Cadmium_Chloride_1_KCR8195 − Water_0_KCR8195
FDR−corrected Log10 P
Log2 fold change
MT1X
NS
Log2 FC
p − value and log2 FC
FDR−corrected permutation p−values

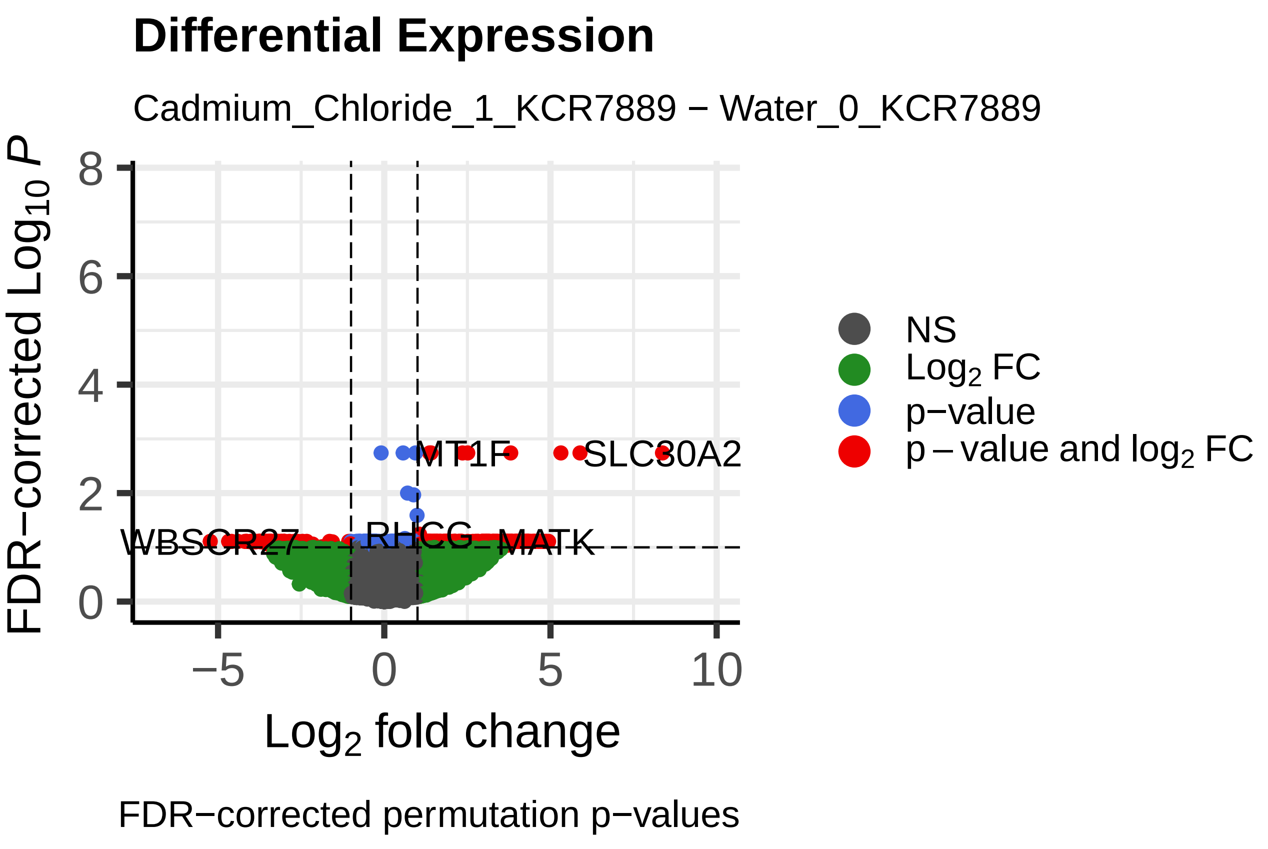
Differential Expression
Cadmium_Chloride_1_KCR7889 − Water_0_KCR7889
FDR−corrected Log10 P
Log2 fold change
MT1F
SLC30A2
WBSCR27
RHCG
MATK
NS
Log2 FC
p−value
p − value and log2 FC
FDR−corrected permutation p−values

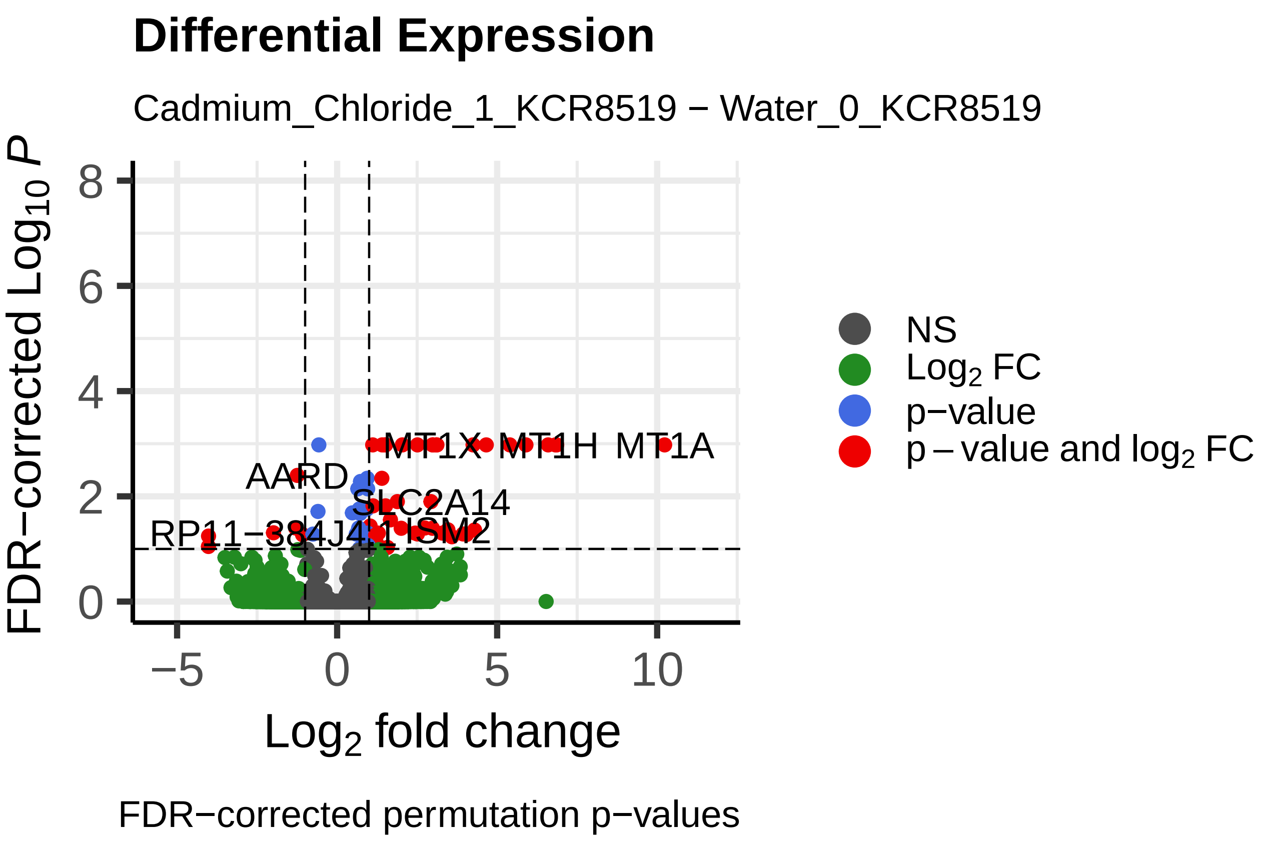
Differential Expression
Cadmium_Chloride_1_KCR8519 − Water_0_KCR8519
FDR−corrected Log10 P
Log2 fold change
MT1X
MT1H
MT1A
AARD
SLC2A14
RP11−384J4
ISM2
NS
Log2 FC
p−value
p − value and log2 FC
FDR−corrected permutation p−values

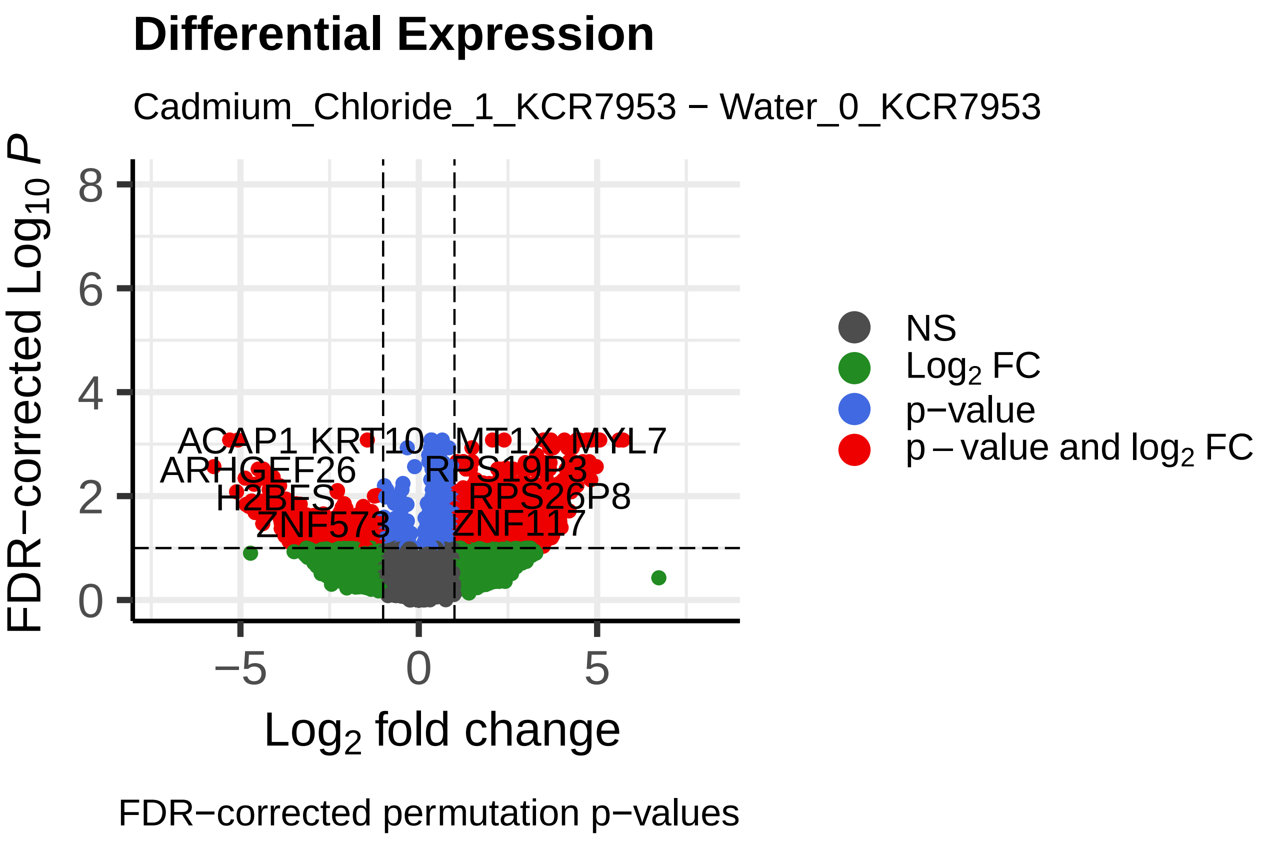
Differential Expression
Cadmium_Chloride_1_KCR7953 − Water_0_KCR7953
FDR−corrected Log10 P
Log2 fold change
ACAP1
KRT10
MT1X
MYL7
ARHGEF26
RPS19P3
H2BFS
RPS26P8
ZNF573
ZNF117
NS
Log2 FC
p−value
p − value and log2 FC
FDR−corrected permutation p−values

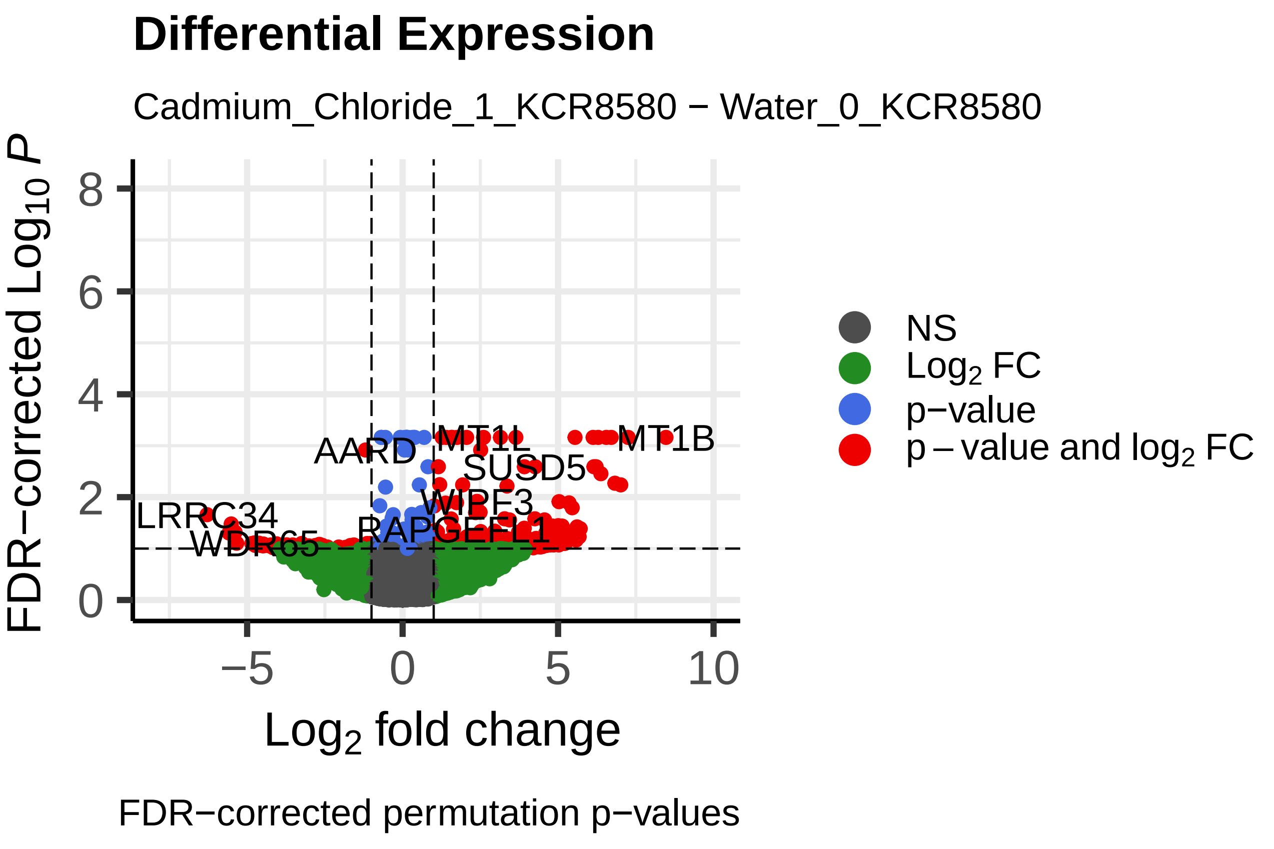
Differential Expression
Cadmium_Chloride_1_KCR8580 − Water_0_KCR8580
FDR−corrected Log10 P
Log2 fold change
MT1L
MT1B
AARD
SUSD5
LRRC34
WIPF3
WDR65
RAPGEFL1
NS
Log2 FC
p−value
p − value and log2 FC
FDR−corrected permutation p−values

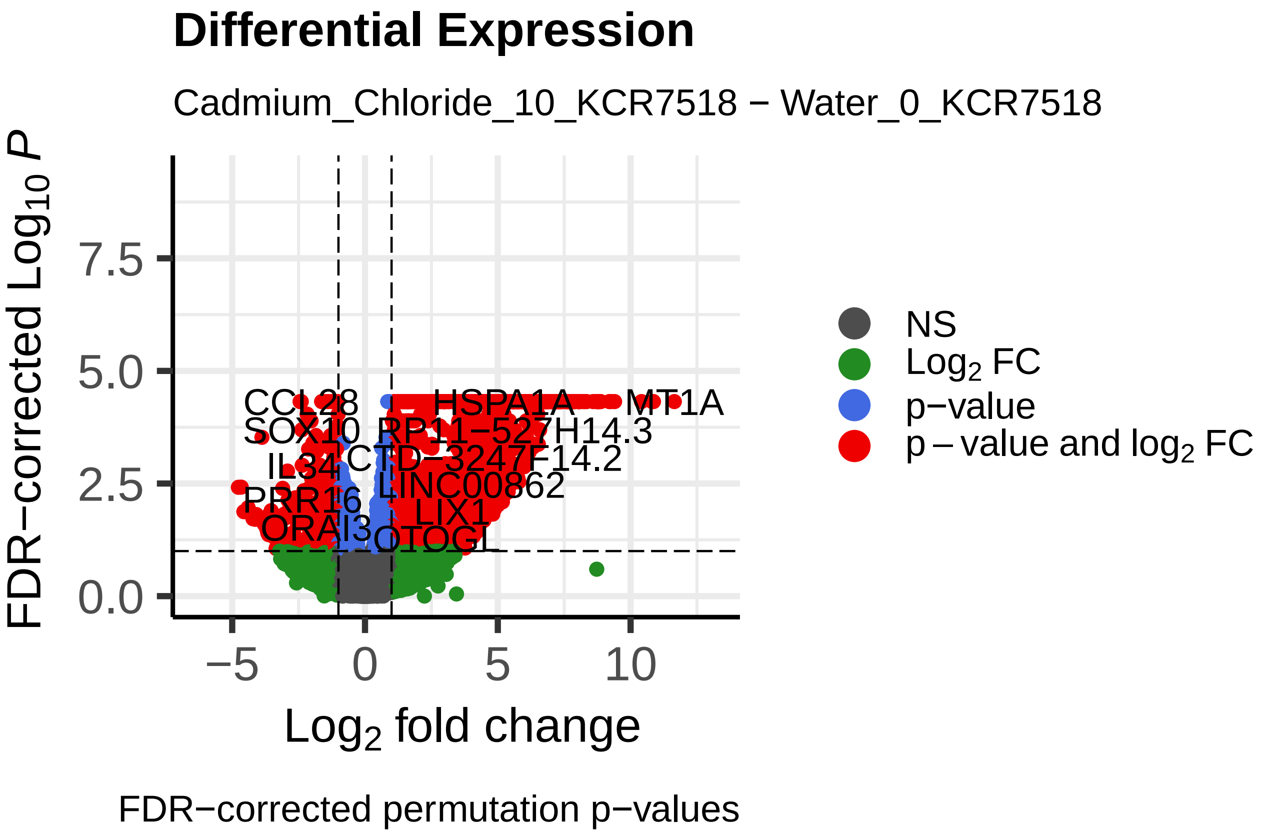
Differential Expression
Cadmium_Chloride_10_KCR7518 − Water_0_KCR7518
FDR−corrected Log10 P
Log2 fold change
NS
Log2 FC
p−value
p − value and log2 FC
FDR−corrected permutation p−values

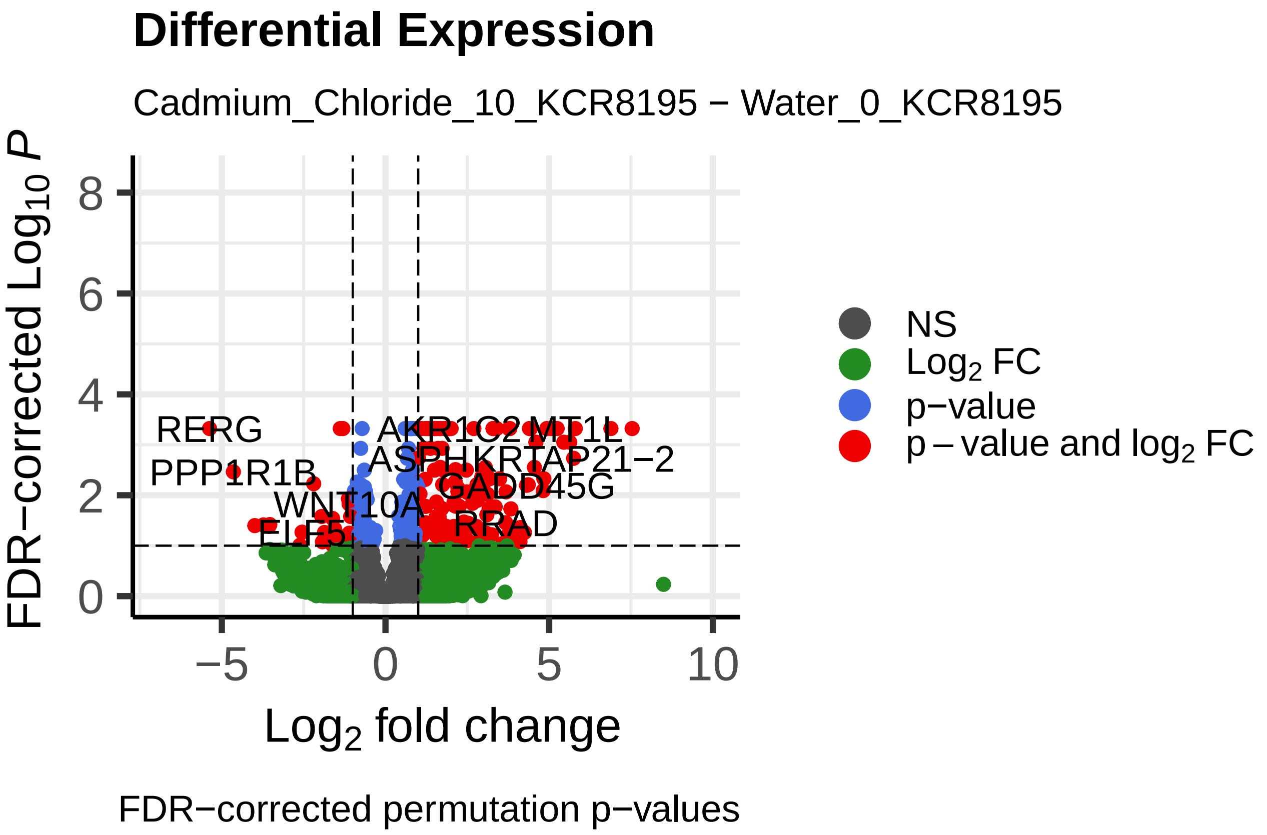
Differential Expression
Cadmium_Chloride_10_KCR8195 − Water_0_KCR8195
FDR−corrected Log10 P
Log2 fold change
NS
Log2 FC
p−value
p − value and log2 FC
FDR−corrected permutation p−values

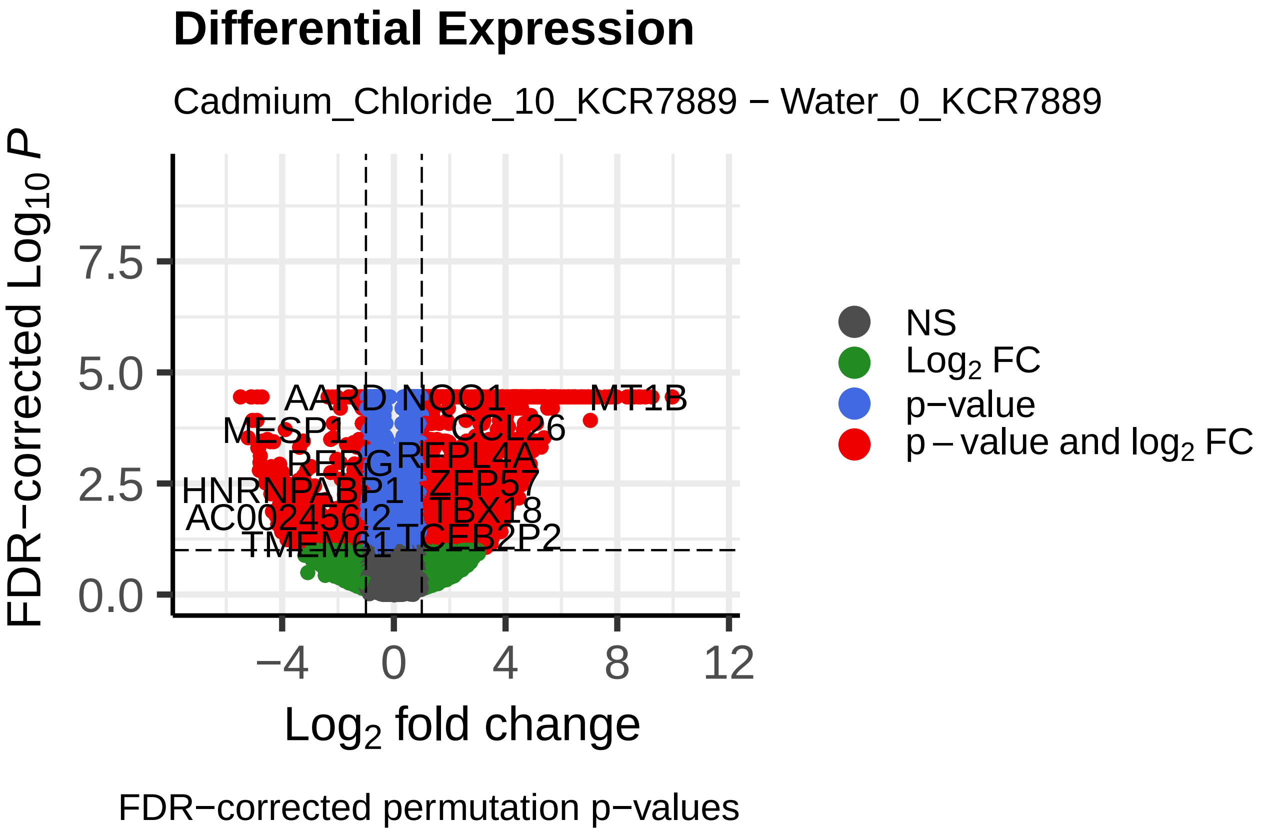
Differential Expression
Cadmium_Chloride_10_KCR7889 − Water_0_KCR7889
FDR−corrected Log10 P
Log2 fold change
NS
Log2 FC
p−value
p − value and log2 FC
FDR−corrected permutation p−values

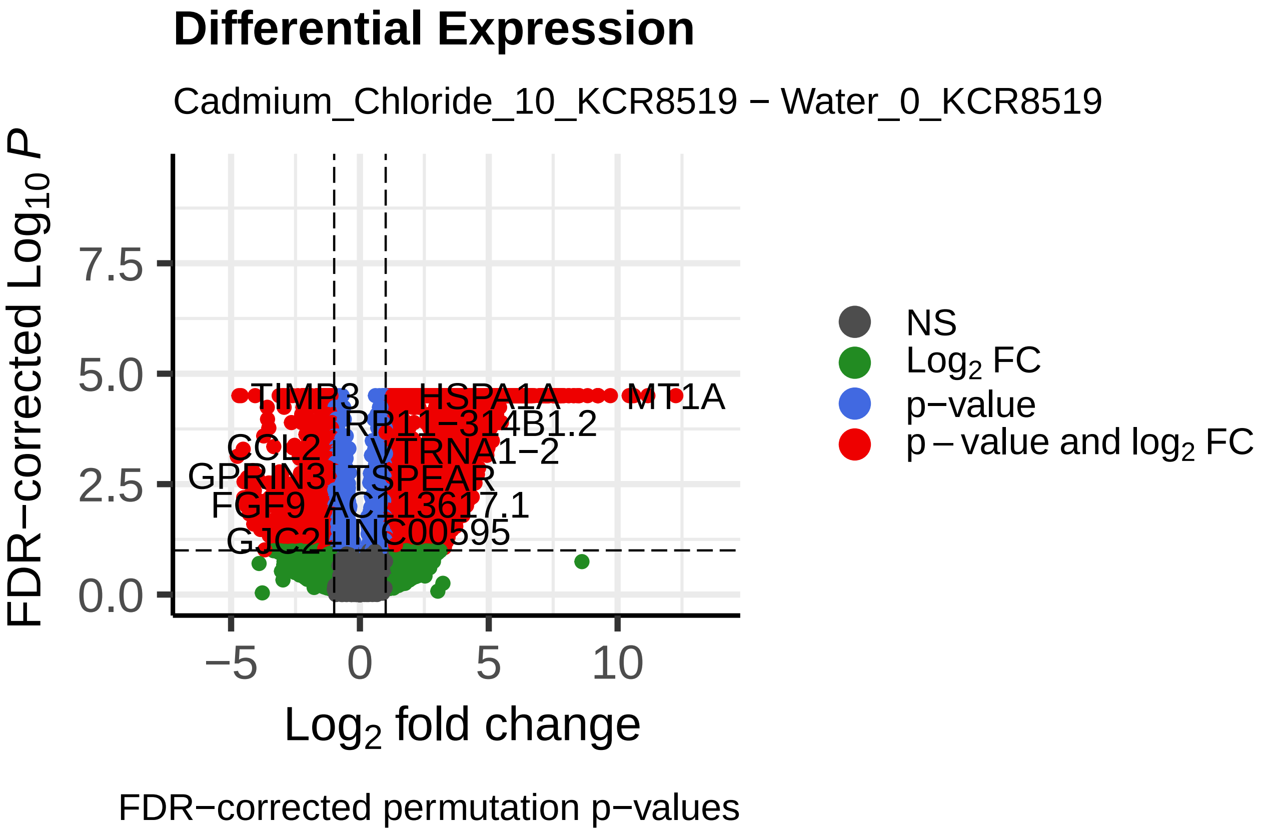
Differential Expression
Cadmium_Chloride_10_KCR8519 − Water_0_KCR8519
FDR−corrected Log10 P
Log2 fold change
NS
Log2 FC
p−value
p − value and log2 FC
FDR−corrected permutation p−values

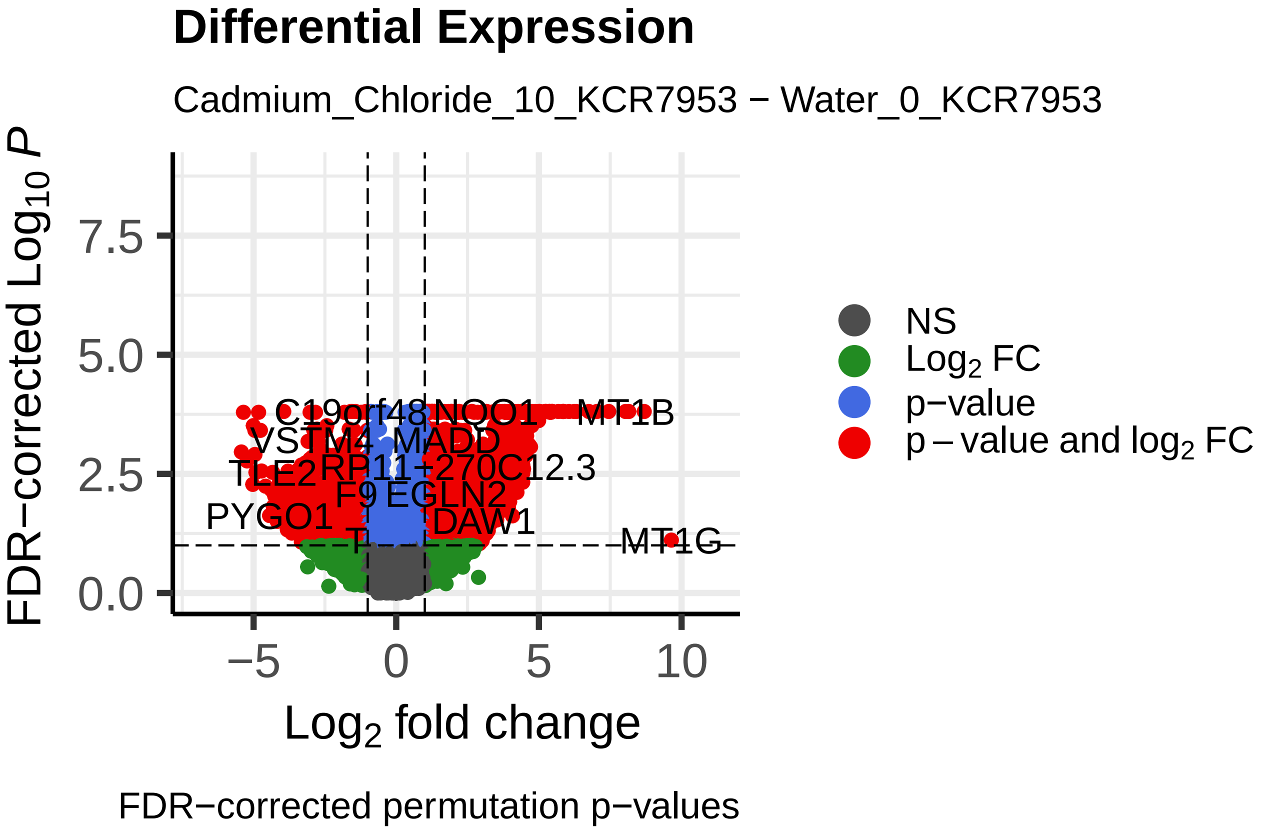
Differential Expression
Cadmium_Chloride_10_KCR7953 − Water_0_KCR7953
FDR−corrected Log10 P
Log2 fold change
NS
Log2 FC
p−value
p − value and log2 FC
FDR−corrected permutation p−values

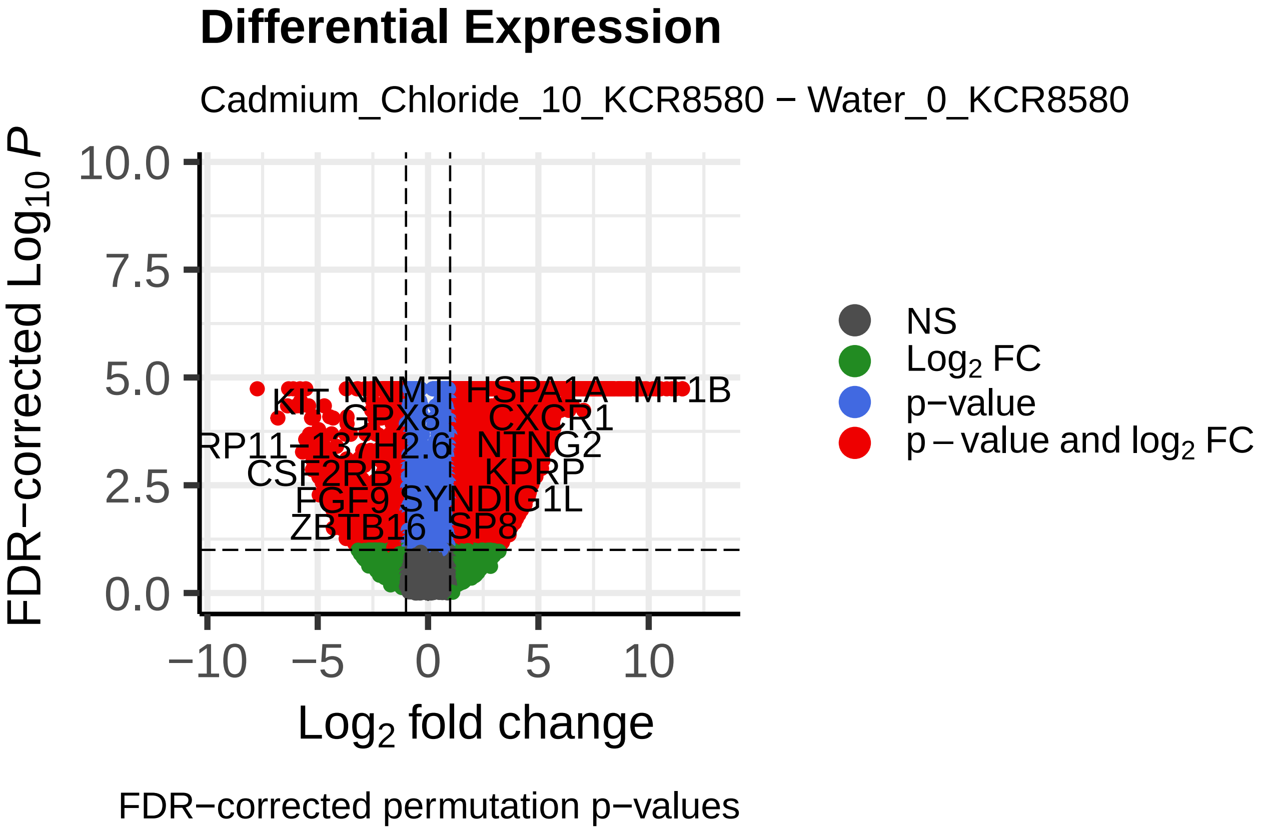
Differential Expression
Cadmium_Chloride_10_KCR8580 − Water_0_KCR8580
FDR−corrected Log10 P
Log2 fold change
NS
Log2 FC
p−value
p − value and log2 FC
FDR−corrected permutation p−values
